# Clinical and safety outcomes in unresectable, very early and early-stage hepatocellular carcinoma following Irreversible Electroporation (IRE) and Transarterial Chemoembolization (TACE): A systematic literature review and meta-analysis

**DOI:** 10.1371/journal.pone.0322113

**Published:** 2025-04-29

**Authors:** Kristen A. Cribbs, Wesley T. Baisley, Betsy J. Lahue, Praveen Peddu

**Affiliations:** 1 Alkemi LLC, Manchester Center, Vermont, United States of America; 2 Department of Clinical and Diagnostic Services, King’s College London, London, United Kingdom; Institute of Medical Sciences, Mongolian National University of Medical Sciences, MONGOLIA

## Abstract

**Background:**

Locoregional treatments for early-stage unresectable hepatocellular carcinoma (HCC) are widely used, with irreversible electroporation (IRE) and transarterial chemoembolization (TACE) representing two non-thermal treatment options. However, to date, no systematic evaluations of these technologies have been conducted. This study sought to comparatively assess the safety and effectiveness of IRE and TACE for the treatment of very early and early-stage, inoperable HCC via systematic literature reviews (SLRs) and meta-analyses.

**Methodology:**

Searches were conducted targeting English-language publications and congress proceedings of clinical trials and observational studies from January 1, 2012 to December 21, 2023 that reported effectiveness and safety outcomes (tumor response, progression-free survival (PFS), adverse events (AE)) for IRE and TACE. Two reviewers independently assessed eligibility and abstracted data. For each procedure, meta-analyses were conducted to assess tumor response by follow-up time point, as data permitted, and other outcomes were descriptively analyzed; Quality and risk of bias assessments were performed.

**Results:**

12 IRE publications (195 patients) and 33 TACE publications (6,899 patients) met eligibility criteria. During 0 to < 3 month follow-up, complete response was achieved in 84% of IRE patients vs. 68% for TACE (all results at 1-month); a proportion that increased at 3 to < 6 months (91% IRE vs. 41% TACE). Median PFS was 10.4 months for IRE and 19–30 months for TACE. Serious AEs (SAEs) were experienced by 4% vs. 5% of IRE and TACE patients, respectively.

**Conclusion:**

Both IRE and TACE are safe and effective non-thermal treatments for unresectable, very early and early-stage HCC. The high rate of short-term complete response observed for IRE, coupled with a low SAE rate, may support the broader adoption of this procedure in this patient population.

## Introduction

Liver cancer is the fourth leading cause of cancer-related death globally and is associated with significant patient burden and mortality, with an incidence rate of 9.5 per 100,000 individuals in the United States (US) and a less than 20% 5-year survival rate [1–3]. The most common form of primary liver cancer is hepatocellular carcinoma (HCC), comprising more than 80% of liver cancer cases worldwide [1]. Treatment paradigms for HCC are complex and depend on factors such as disease stage, liver function, and underlying patient comorbidities [1]. Approximately 40% of patients with very early or early-stage HCC are eligible for therapies that are potentially curative, including resection, transplantation, and local ablation [4]. These treatment approaches have previously been found to nearly double median survival from 36 months to 60 months compared to a natural history cohort [4]. Due to advancements in screening, more patients are being diagnosed with HCC at earlier stages of the disease [5]. However, for many patients, their cancer is considered unresectable due to underlying conditions or proximity of the lesions to critical structures (e.g., blood vessels) [4,5].

Among patients with early-stage HCC who are not candidates for surgical resection or transplantation, ablative modalities have become an accepted option in HCC treatment guidelines [6]. Two such modalities include irreversible electroporation (IRE) and transarterial chemoembolization (TACE) [7,8]. IRE is an image-guided tissue ablation technology that induces cell death via very short, strong, pulsed electric fields [9]. The NanoKnife^®^ System (AngioDynamics, Inc., Latham, NY, USA) is a commercially available IRE system that received 510(k) premarket clearance from the Food and Drug Administration (FDA) in 2019 [10]. Due to its non-thermal nature, IRE preserves vessels, nerves, and the extracellular matrix near the treatment zone, making it an effective treatment modality for solid tumors, including those that have permeated surrounding tissue. Side effects resulting from necrosis that are common among typical thermal HCC ablation methods, such as radiofrequency ablation (RFA) and microwave ablation (MWA), are not seen with IRE [11].

TACE is a non-surgical, image-guided, locoregional treatment that is recommended as a first-line treatment in unresectable, intermediate-stage HCC, but is also often used in earlier disease stages [12,13]. TACE causes tumor death by two primary mechanisms of action – intra-arterial infusion of cytotoxic chemotherapy agents and embolization of the tumor-feeding artery via gelatin sponge or solid embolic agents [1,8]. The chemotherapy is delivered in a highly concentrated manner to the cancer cells, and the lack of blood flow due to embolization allows the chemotherapy to remain concentrated around the cancer cells [14].

As ablative technologies are more frequently used to treat HCC, evidence gaps have emerged around their efficacy, real-world effectiveness, and safety, specifically in early-stage HCC [7,8]. While several studies have demonstrated the positive impact IRE has on HCC outcomes, including improving local recurrence free survival [15] and mean overall survival time [16], no systematic literature reviews (SLRs) or meta-analyses have been conducted to date to synthesize the body of evidence surrounding the efficacy of IRE for use specifically in unresectable, early-stage HCC [11,16]. Similarly, studies have shown that TACE improves overall survival for patients with early-stage HCC [17,18], but no SLRs or meta-analyses have been conducted to comprehensively assess the efficacy of TACE alone for use in unresectable, early-stage HCC. The goal of the present research was to systematically evaluate and synthesize the global body of evidence on the IRE and TACE technologies in liver ablation, specifically for patients with inoperable tumors.

## Methodology

Detailed protocols were developed for the IRE and TACE SLRs and meta-analyses a priori, in alignment with best practice guidelines for systematic reviews on health interventions, including Preferred Reporting Items for Systematic Reviews and Meta-Analyses for Protocols (PRISMA-P) and A MeaSurement Tool to Assess systematic Reviews 2 (AMSTAR 2). [19,20]. Additionally, the protocols were registered in the International Prospective Register of Systematic Reviews (PROSPERO) to facilitate transparency and minimize bias in the review process (IRE CRD: CRD42024498540; TACE CRD: CRD42024498549). The SLRs were conducted by the study authors, who are health services researchers with technical and subject-matter expertise in medical devices, liver cancer, health technology assessment, and SLR and meta-analysis methodologies.

### Search strategy

In alignment with SLR objectives and research questions, search strategies queried key effectiveness outcomes of interest, progression free survival (PFS) and tumor response, as well as adverse event (AE) outcomes related to the use of IRE and TACE for treatment of early-stage, unresectable HCC. For searches conducted in PubMed, medical subject headings (MeSH) and free-text searches were used to identify relevant literature. Hand-searching was done in Google Scholar using free text searches that included controlled vocabulary and keywords aligned to the research questions. Use of Google Scholar in SLRs has been recommended to capture unique references and ensure review completeness [21]. Search strategies for the IRE and TACE reviews are detailed in S1 Table.

### Study selection

Eligibility criteria for the reviews were defined according to Population, Intervention, Comparator, Outcomes, and Study Design (PICOS) criteria. Specific eligibility criteria for the IRE and TACE reviews are outlined in Table 1.

**Table 1:** PICOS study selection criteria, IRE and TACE reviews.

PICOS Criteria	IRE		TACE	
	Inclusion Criteria	Exclusion Criteria	Inclusion Criteria	Exclusion Criteria
Population	Patients with unresectable (due to tumor location), very early and early-stage HCC	Resectable HCC Other stage HCC Other liver cancer types Other cancer types	Patients with unresectable (due to tumor location), early-stage HCC	Resectable HCC Other stage HCC Other liver cancer types Other cancer types
Intervention	IRE	Non-IRE modalities Combined IRE with other locoregional modalities (e.g., chemotherapy)	All TACE including: DEB-TACE, OEM-TACE, B-TACE, DEM-TACE, DSM-TACE, DEE-TACE, PE-TACE	Non-TACE modalities Combined TACE with other locoregional modalities (e.g., chemotherapy)
Comparator	Non-surgical HCC treatment modalities, such as radiofrequency ablation, microwave ablation, cryotherapy, brachytherapy, TACE	Resection Transplant	Non-surgical HCC treatment modalities, such as radiofrequency ablation, microwave ablation, cryotherapy, brachytherapy, IRE	Resection Transplant
Outcome	PFS, Tumor Response, AEs	Other clinical and safety outcomes not noted, Economic outcomes, Health system outcomes, Patient outcomes	PFS, Tumor Response, AEs	Other clinical and safety outcomes not noted, Economic outcomes, Health system outcomes, Patient outcomes
Study Design	RCTs, clinical trials, observational studies (retrospective and prospective)	Animal studies, Case reports, Meta-analyses, SLRs, other reviews	RCTs, clinical trials, observational studies (retrospective and prospective)	Animal studies, Case reports, Meta-analyses, SLRs, other reviews
Publication Types	Peer reviewed full text articles and conference proceedings (abstracts, posters, presentations)	Opinion pieces, Editorials, Grey literature	Peer reviewed full text articles and conference proceedings (abstracts, posters, presentations)	Opinion pieces, Editorials, Grey literature
Publication Date	January 1, 2012-December 31, 2023	Prior to January 1, 2012	January 1, 2012-December 31, 2023	Prior to January 1, 2012
Language	English	Non-English language	English	Non-English language

Abbreviations: HCC, hepatocellular carcinoma; IRE, irreversible electroporation; TACE, transarterial chemoembolization; PICOS, Population, Intervention, Comparator, Outcomes, and Study Design; SLR, systematic literature review; RCT, randomized controlled trial; PFS, progression free survival; AE, adverse event; OEM-TACE, oxaliplatin-eluting microspheres transarterial chemoembolization; DEB-TACE, drug-eluting bead transarterial chemoembolization; B-TACE, balloon occluded transarterial chemoembolization; DEM-TACE, drug-eluting-microsphere transarterial chemoembolization; DSM-TACE, transarterial chemoembolization with degradable starch microspheres; DEE-TACE, drug-eluting embolic transarterial chemoembolization; PE-TACE, pirarubin-eluting transarterial chemoembolization.

In regard to the population of interest, early-stage disease was defined by the Barcelona Clinic Liver Cancer (BCLC) classification system, which utilizes the performance status (PS) scale and Child-Pugh system to evaluate health state and liver function, respectively [22]. Articles that included patients with BCLC Stages 0 or A were considered for inclusion in the IRE and TACE reviews. If an article did not report BCLC stage, criteria corresponding to BCLC Stages 0 and A were applied by the reviewers to ascertain whether the population met inclusion criteria for both reviews. Outcomes of interest for the IRE and TACE SLRs included clinical effectiveness and safety endpoints. A summary of BCLC classifications and outcomes of interest, including definitions, can be found in S2 and S3 Tables.

Following the literature searches, all records identified were imported into a citation management software program, where results were deduplicated. Unique citation records were then imported into an SLR software program for screening. Article eligibility was assessed by two reviewers through appraisal of unique publication titles and abstracts. To narrow the focus on outcomes of interest following preliminary screening, secondary screening was conducted, after consultation with the research team, to identify articles that included patients with only very early or early-stage, unresectable HCC. Publications that were determined to meet disease stage inclusion criteria were then extracted in full-text and independently re-screened by two reviewers for inclusion in the final review. Disagreements throughout the screening process were adjudicated by a third reviewer. None of the reviewers were blind to the study authors and their affiliated institutions.

### Data abstraction

Following final article selection, one reviewer used a customized template within the SLR software platform to abstract data on key domains and items (Table 2). A comma-separated values (CSV) file containing fully abstracted final review records was subsequently exported and saved on a secure, restricted access platform and referenced during literature synthesis. A citation library containing all final review records and portable document format (PDF) files was also created.

**Table 2:** Data abstraction elements, IRE and TACE reviews.

Publication Information	Study Design and Methodology	Patient Baseline Demographic and Clinical Characteristics	Effectiveness and Safety Outcomes
Full Citation Publication Type	Study Design Intervention & Comparator Location of intervention Study Location, Country, and Site Number of sites Sample Size Imaging Modality Used Methodology Description Criteria Used to Assess Response to Therapy AE Grading System	Age HCC Disease Stage Number of Tumors at Baseline Tumor Size at Baseline	Time to PFS PFS Rate at Follow-up Tumor Response Rate at Follow-up AEs/SAEs by Grade AEs/SAEs by Severity Total AEs/SAEs

Abbreviations: HCC, hepatocellular carcinoma; PFS, progression free survival; AE, adverse event; SAE, serious adverse event

### Assessment of methodological quality

The quality of studies included in the SLRs was assessed through risk of bias and quality assessments. Two independent reviewers assessed risk of bias for each publication using the National Institutes of Health (NIH) Study Quality Assessment Tools [23]. These tools encapsulate various dimensions of study design and implementation, which reviewers utilized to assess whether a publication was “good,” “fair,” or “poor” quality. Disagreements in bias assessments were resolved by a third reviewer.

Final review publications were also subject to a quality assessment using the Grades of Recommendation, Assessment, Development, and Evaluation (GRADE) methodology, a systematic approach to rating the certainty of evidence from published literature [24]. In accordance with GRADE criteria, evidence from randomized controlled trials (RCTs) started at high quality and, due to residual confounding, evidence from observational studies started at low quality. Reviewers assessed risk of bias, imprecision, inconsistency, indirectness, and publication bias, increasing or decreasing the quality rating as necessary to arrive at a final quality grade assignment (very low, low, moderate, or high).

### Analysis

Both quantitative and qualitative analysis techniques were employed to synthesize SLR data. Study and baseline patient characteristics were summarized descriptively, using counts, proportions, means, and standard deviations, as well as using 2-tailed t-tests (*p*-value=0.05) to assess statistically significant differences between groups, as appropriate.

The primary analysis involved a quantitative meta-analysis of tumor response to calculate the impact of IRE and TACE on very early and early-stage HCC tumors. Due to potential differences between study populations for each treatment, since individual studies did not uniformly incorporate randomization of treatments, analysis was performed separately by treatment. Analysis was performed using R software for Windows, version 4.2. Ninety-five percent confidence intervals (CIs) for the estimated proportions were computed using the Wilson Score approach for proportions with a continuity correction to account for the discrete nature of percentages based on small numbers of subjects for some studies and outcomes. A restricted maximum likelihood random effects meta-analysis technique was used to combine the estimated proportions for RECIST/mRECIST outcomes [25] from individual studies and to estimate additional variation in study proportion estimates beyond what would be expected due to sampling error, known as the heterogeneity. The *I*^2^ measure was used to estimate the percentage of variance in the study proportion estimates that is due to heterogeneity (see S4 Table).

Estimated proportions were analyzed with both fixed effect and random effect models, and both outputs were included in each forest plot. For TACE studies whose interventions and comparators included multiple types of TACE that met inclusion criteria (see Table 1), data from all relevant TACE treatments were included in meta-analyses. Per exclusion criteria, a TACE treatment was not included in meta-analysis if combined with other locoregional modalities (e.g., chemotherapy). To appropriately ascertain treatment impact, primary analyses were conducted by time point following treatment (0 to < 3 months, 3 to < 6 months, 6–12 months, 12+ months), as data allowed. For TACE, a sub-analysis was conducted to explore the impact of TACE type on tumor response. Studies not reporting a discrete follow-up time point that fell within the groupings noted above (e.g., studies reporting mean, median, or follow-up range), as well as those not reporting follow-up time point, were excluded from meta-analyses to minimize bias.

For tumor response, PFS, and AE outcomes that were deemed not suitable for meta-analyses, qualitative analysis methods were employed to summarize findings. Key inferences were contextualized by assessments of the strength of evidence and study quality across reviews. For all analyses, in cases of actual or perceived missing data, articles or data points were omitted to minimize bias.

## Results

### Study and sample characteristics

**Fig 1:**
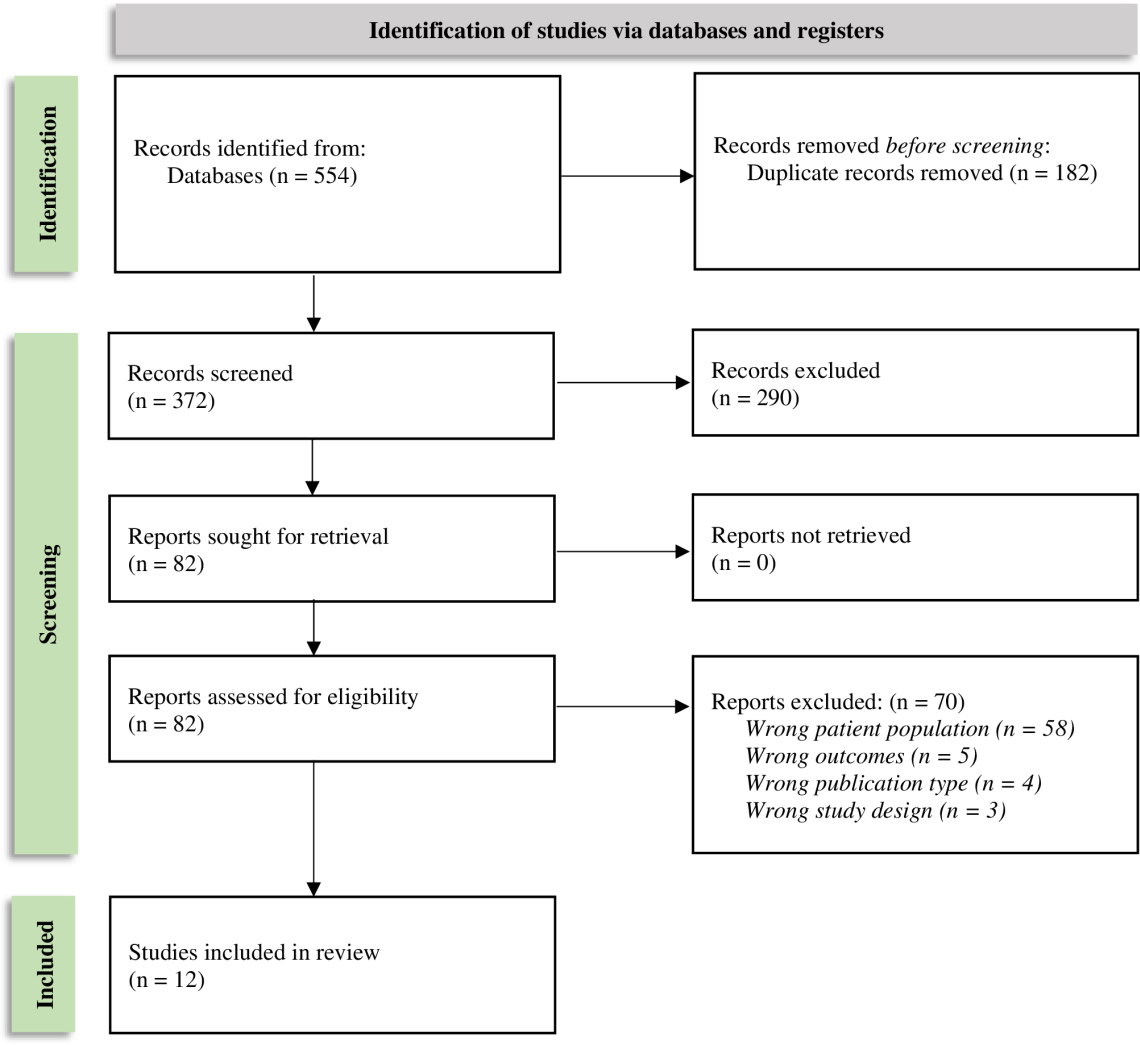
IRE SLR PRISMA flow diagram.

**Fig 2:**
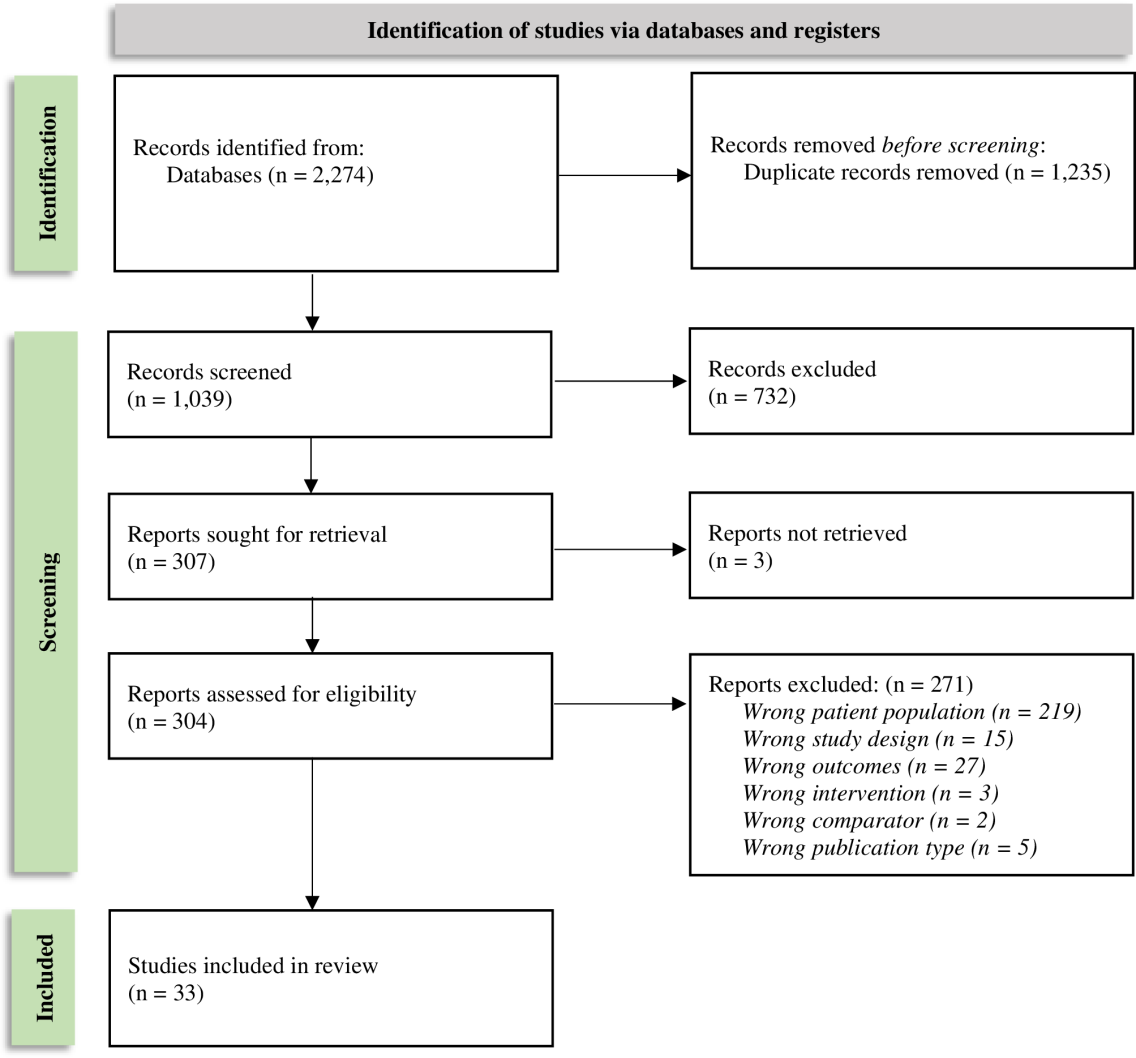
TACE SLR PRISMA flow diagram.

Following screening, 12 IRE publications and 33 TACE publications were determined to meet eligibility criteria and were included in the final reviews (Figs 1 and 2). See S18 and S19 Tables for full details on study selection.

Most IRE (n=8, 67%) studies were observational, with sample sizes ranging from 5 to 26 patients [26,27] and a total of 195 patients across included studies. Similarly, TACE studies were predominantly observational (n=28, 85%), with sample sizes ranging from 28 to 1,002 patients [12,13] and a total of 6,899 patients across included studies.All studies reporting on the use of IRE utilized the NanoKnife System. With the exception of Lencioni et al. [28], which was only available as an abstract, IRE was noted in all other publications to be used for patients contraindicated for thermal ablation or transplant based on tumor proximity to organs/other structures or other vascular criteria. Freeman et al. [15] required IRE to be the most suitable treatment based on the proximity of tumors to solid organs or vascular structures, which made thermal ablation unsuitable due to risk of thermal injury or decreased efficacy due to the heat-sink effect.

Most studies were single site (IRE: n=9, 90%; TACE: n=24, 71%) and took place in either Europe or Asia (IRE: n=9, 75%; TACE: n=31, 91%). Comparator treatments were assessed in 2 (17%) IRE studies and 18 (55%) TACE studies. Comparators in the 2 IRE studies included RFA (n=2) [29,30] and MWA (n=1) [29]. TACE studies commonly compared conventional TACE (cTACE) and drug-eluting bead TACE (DEB-TACE) (n=7 studies) [13,30–35].

While TACE was repeated in some studies due to tumor recurrence, the specific number of procedures was not reported. Chen et al. [36] noted the TACE was repeated when there was evidence of a residual viable tumor or intrahepatic recurrence on follow-up imaging. Kudo et al. [37] reported that tumors were assessed by dynamic computed tomography (CT) or magnetic resonance imaging (MRI) and tumor marker tests every 8 weeks, beginning 4 weeks after initial TACE treatment. TACE was continued until untreatable progression, progression to meet the TACE refractoriness criteria, unacceptable toxicity or withdrawal of consent.

The mean age of patients treated with TACE was slightly lower than those treated with IRE (63.0 ± 5.3 vs. 67.5 ± 4.4 years, respectively). Patients treated with TACE and IRE had the same mean number of tumors at baseline: 1.2 ± 0.2. Tumor size at baseline was mean 3.3 ± 1.3 cm for TACE versus 1.9 ± 0.4 for IRE. No statistically significant differences were observed among IRE and TACE cohorts for mean age, mean number of tumors, or mean tumor size. Additional details on study and baseline sample characteristics for both reviews can be found in Tables 3 and 4 and S5 and S6 Tables.

**Table 3:** Study characteristics, IRE and TACE reviews.

	IRE		TACE	
**Studies (n)**	**Patients (n)**	**Studies (n)**	**Patients (n)**
**Totals**	12	195	33	6,899
**Minimum study sample size**	–	5	–	28
**Maximum study sample size**	–	26	–	1,002
**Study design**	**Studies (n)**	**Patients (n)**	**Studies (n)**	**Patients (n)**
Prospective observational	1	20	5	810
Retrospective observational	7	125	23	5,477
Non-randomized controlled trial	4	50	0	–
Randomized controlled trial	0	–	5	612
**Publication type**	**Studies (n)**	**Patients (n)**	**Studies (n)**	**Patients (n)**
Original research	11	169	33	6,899
Conference proceeding	1	26	0	–
**Study location**	**Studies (n)**	**Patients (n)**	**Studies (n)**	**Patients (n)**
Europe	5	87	10	894
North America	1	20	0	–
Australia	2	34	1	51
Other^a^	4	54	22	5,954
**Institutional setting**	**Studies (n)**	**Patients (n)**	**Studies (n)**	**Patients (n)**
Single site	11	169	24	4,181
Multisite	1	26	8	2,668
Not specified	0	–	1	50
**Imaging modality used**	**Studies (n)**	**Patients (n)**	**Studies (n)**	**Patients (n)**
CT scan^b^	11	–	33	–
MRI	8	–	28	–
Not reported	1	–	1	–
Other^c^	3	–	1	–
**Response to Therapy Criteria Used**	**Studies (n)**	**Patients (n)**	**Studies (n)**	**Patients (n)**
mRECIST	4	–	25	–
RECIST	1	–	2	–
Not reported	7	–	3	–
Other^d^^,e^	0	–	4	–
**AE Grading Criteria Used**	**Studies (n)**	**Patients (n)**	**Studies (n)**	**Patients (n)**
SIR	4	–	3	–
CTCAE	1	–	16	–
Clavien-Dindo	1	–	0	–
CIRSE	1	–	2	–
Not reported	5	–	12	–

Abbreviations: IRE, irreversible electroporation; TACE, transarterial chemoembolization; CIRSE, Cardiovascular and Interventional Radiological Society of Europe; cm, centimeters; CT, computed tomography; CTCAE, Common Terminology Criteria for Adverse Events; MRI, magnetic resonance imaging; mRECIST, modified Response Evaluation Criteria in Solid Tumors; RECIST, Response Evaluation Criteria in Solid Tumors; SIR, Society of Interventional Radiology

^a^Includes South America, Asia, and Middle East

^b^Includes contrast enhanced CT [38,39] and multidetector CT [30]

^c^Includes positron emission tomography (PET) scan [40], contrast enhanced ultrasound (CEUS) [41,42] and bone scintigraph [43]

^d^Includes Liver Cancer Study Group of Japan [44], Response Evaluation Criteria in Cancer of the Liver (RECICL) [37], and European Association for the Study of the Liver (EASL) [30,45]

**Table 4:** Very early and early-stage patient characteristics, IRE and TACE reviews.

Patient Characteristics	IRE		TACE		*p*-value
**Studies (n)**	**Mean ± SD**	**Studies (n)**	**Mean ± SD**	
Age (years)	8	67.5 ± 4.4	8	63.0 ± 5.3	0.09
Tumors (n)	9	1.2 ± 0.2	8	1.2 ± 0.2	0.87
Tumor size (cm)	8	1.9 ± 0.4	5	3.3 ± 1.3	0.07

Abbreviations: SD, standard deviation; cm, centimeters; IRE, irreversible electroporation; TACE, transarterial chemoembolization

### Meta-analysis results

#### Tumor response.

Tumor response outcomes analyzed during IRE and TACE meta-analyses included objective response (OR), complete response (CR), partial response (PR), progressive disease (PD), and stable disease (SD) for IRE and TACE at pre-defined follow-up time periods, as data allowed.

**Fig 3:**
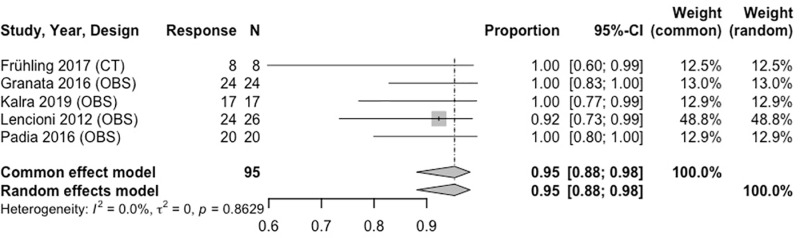
Forest plot, IRE OR results at 1 month.

**Fig 4:**
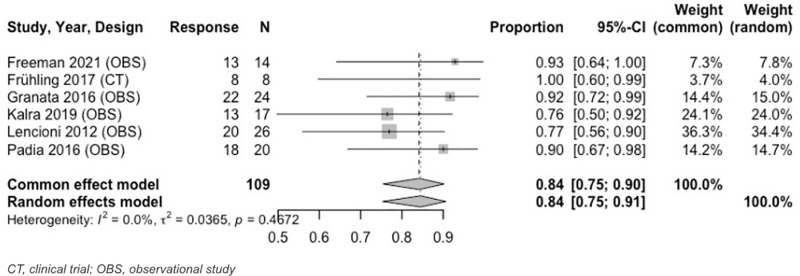
Forest plot, IRE CR results at 1 month.

Seven unique IRE studies were included in tumor response meta-analysis. For 0 to < 3-month analyses, all IRE studies reported tumor response at 1 month. At 1 month, the combined estimated OR rate was 95% (95% CI: 88%–98%) (Fig 3), and CR rate was 84% (95% CI: 75%–90%) (Fig 4). The PR rate was 14% (95% CI: 8%–23%) and SD rate was 4% (95% CI: 1%–11%). PD, an indicator of disease progression, was 3% (95% CI: 1%–9%).

**Fig 5:**
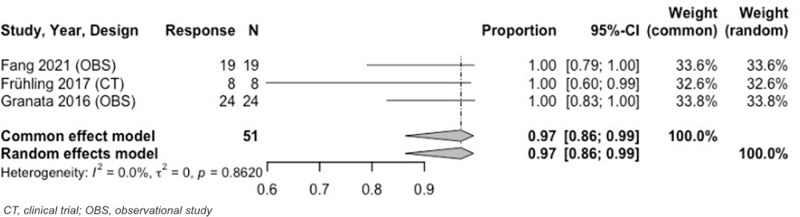
Forest plot, IRE OR results at 3 months.

**Fig 6:**
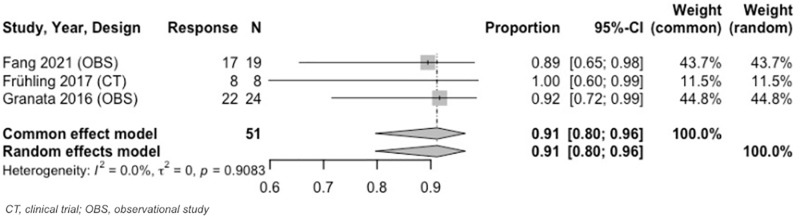
Forest plot, IRE CR results at 3 months.

Analysis from 3 to < 6–months revealed an OR of 97% (95% CI: 86%–99%) and CR of 91% (95% CI: 80%–96%) (Figs 5, 6). Both results were at 3 months. During this same time period (3 months), the estimated PR was 9% (95% CI: 4%–20%). Heterogeneity across IRE MAs was low. Table 5 and S1–S4 Figs include further details on IRE MA results.

**Table 5:** Tumor response meta-analysis results, IRE and TACE reviews.

Response	Studies (n)	Fixed Effect Percent [95% CI]	Random Effects Percent [95% CI]	*I* ^ 2^
IRE Tumor Response by Time Period^a^				
0 to < 3 Months				
OR	5	0.95 [0.88; 0.98]	0.95 [0.88; 0.98]	0.0%
CR	6	0.84 [0.75; 0.90]	0.84 [0.75; 0.91]	0.0%
PR	5	0.14 [0.08; 0.23]	0.14 [0.08; 0.23]	0.0%
SD	4	0.04 [0.01; 0.11]	0.04 [0.01; 0.11]	0.0%
PD	5	0.03 [0.01; 0.09]	0.03 [0.01; 0.09]	0.0%
3 to < 6 Months				
OR	3	0.97 [0.86; 0.99]	0.97 [0.86; 0.99]	0.0%
CR	3	0.91 [0.80; 0.96]	0.91 [0.80; 0.96]	0.0%
PR	3	0.09 [0.04; 0.20]	0.09 [0.04; 0.20]	0.0%
TACE Tumor Response by Time Period^b^				
0 to < 3 Months				
OR	13	0.87 [0.85; 0.89]	0.88 [0.83; 0.92]	65%
CR	14	0.68 [0.65; 0.70]	0.67 [0.60; 0.73]	72.2%
PR	12	0.29 [0.26; 0.32]	0.31 [0.25; 0.37]	73.8%
SD	10	0.10 [0.08; 0.13]	0.08 [0.04; 0.14]	59%
PD	11	0.02 [0.01; 0.03]	0.02 [0.01; 0.03]	0.0%
3 to < 6 Months				
OR	4	0.67 [0.63; 0.71]	0.73 [0.63; 0.81]	68.4%
CR	8	0.41 [0.37; 0.44]	0.48 [0.37; 0.59]	86.6%
PR	3	0.30 [0.24; 0.37]	0.30 [0.24; 0.37]	13.9%
SD	3	0.13 [0.09; 0.18]	0.13 [0.09; 0.18]	0.0%
PD	3	0.09 [0.06; 0.14]	0.10 [0.05; 0.17]	50.0%
6-12 Months				
OR	3	0.59 [0.51; 0.66]	0.63 [0.47; 0.76]	71.0%
CR	3	0.59 [0.51; 0.66]	0.64 [0.47; 0.77]	72.7%
OR by TACE Type				
DEB-TACE	4	0.81 [0.73; 0.87]	0.84 [0.72; 0.91]	42.6%
cTACE	7	0.87 [0.84; 0.90]	0.91 [0.84; 0.96]	76.7%
CR by TACE Type				
DEB-TACE	5	0.58 [0.50; 0.65]	0.58 [0.42; 0.72]	71.4%
cTACE	10	0.72 [0.69; 0.75]	0.72 [0.66; 0.78]	60.7%

Abbreviations: **OR, objective response; CR, complete response; PR, partial response; SD, stable disease; PD, progressive disease**

^a^ **For IRE time point analyses, all 0 to < 3-month analyses were reported at 1 month follow-up and 3 to <6-month analyses were reported at 3 months follow-up.**

^b^ **For TACE time point analyses, all 0 to < 3-month analyses were reported at 1 month follow-up.**

**Fig 7:**
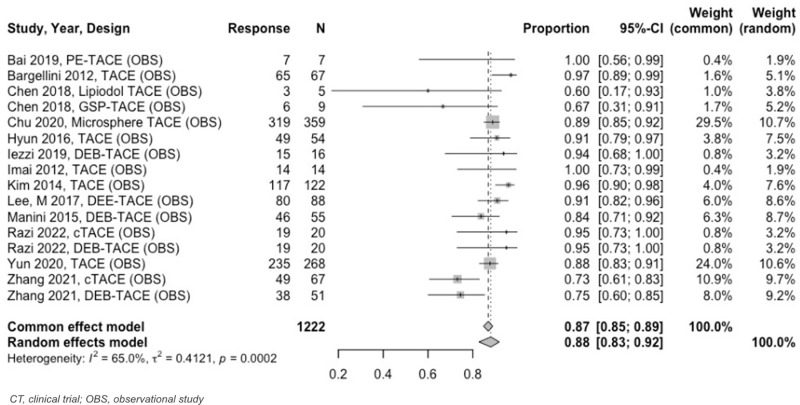
Forest plot, TACE OR results at 1 month.

**Fig 8:**
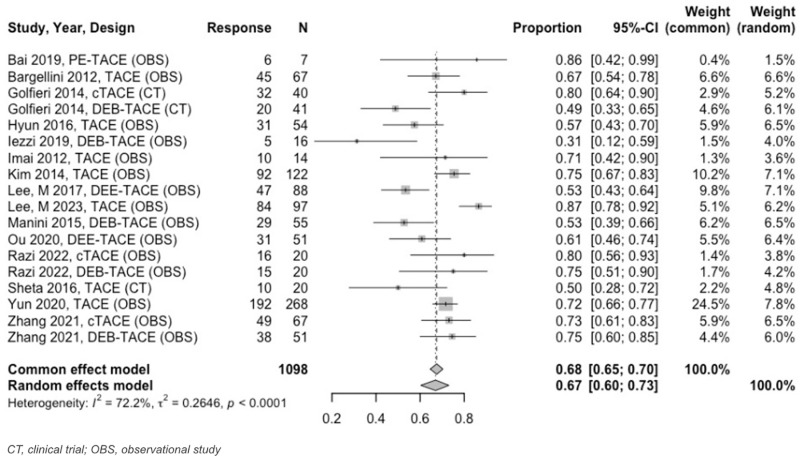
Forest plot, TACE CR results at 1 month.

Twenty–one unique TACE studies were included in tumor response meta–analysis. For 0 to < 3–month analyses, all TACE studies reported tumor response at 1 month. At 1 month follow–up, estimated OR was 87% (95% CI: 85%–89%) and CR was 68% (95% CI: 65%–70%) (Figs 7 and 8). PR and SD rates were 29% (95% CI: 26%–32%) and 10% (95% CI: 8%–13%), respectively, while PD was 2% (95% CI: 1%–3%).

**Fig 9:**
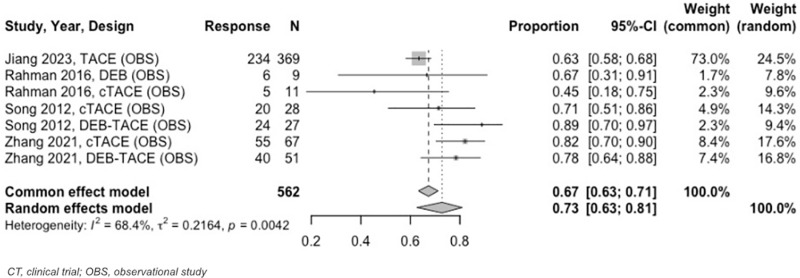
Forest plot, TACE OR results, 3 to < 6 months.

**Fig 10:**
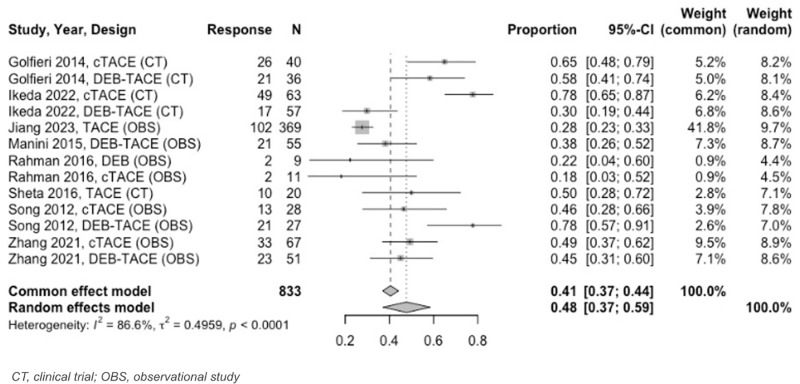
Forest plot, TACE CR results, 3 to < 6 months.

**Fig 11:**
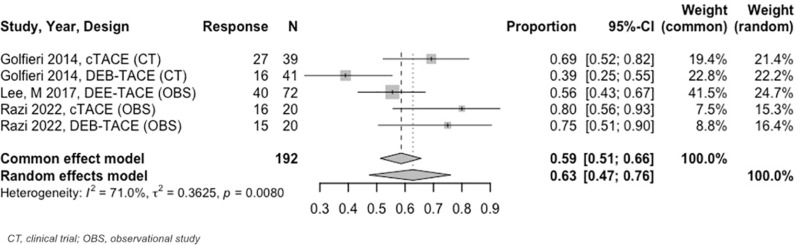
Forest plot, TACE OR results, 6 to 12 months.

**Fig 12:**
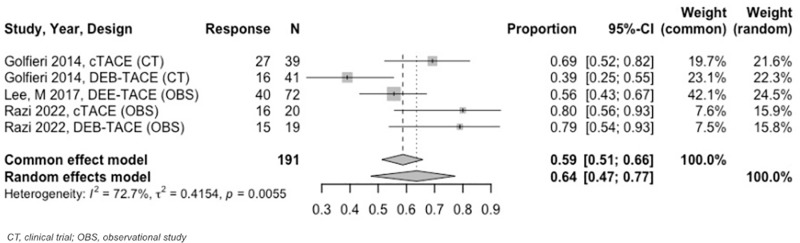
Forest plot, TACE CR results, 6 to 12 months.

At 3 to < 6–months, the estimated OR rate was 67% (95% CI: 63%–71%) and CR rate was 41% (95% CI: 37%–44%) (Figs 9 and 10). The PR was 30% (95% CI: 24%–37%), SD was 13% (95% CI: 9%–18%), and PD was 9% (95% CI: 6%–14%). Six–to–twelve–month analyses revealed an OR and CR of 59% (95% CI: 51%–66%) for both categories (Figs 11 and 12).

Sub-analysis of OR and CR by TACE type revealed that cTACE had a higher estimated tumor response rates compared to DEB-TACE for both OR and CR outcomes (87% vs. 81% OR; 72% vs. 58% CR). The amount of heterogeneity for these outcomes was high, with *I*^2^ values as high as 77%. Further details on TACE meta-analysis results can be found in Table 5 and S5–S10 Figs.

### Qualitative analysis results

#### Tumor response.

For both IRE and TACE, 2 studies each reported tumor response outcomes beyond 12 months follow up [26,46–48]; however, insufficient data precluded these studies from inclusion in meta-analyses. For IRE, one clinical trial reported a CR of 67% (8/12) during mean follow-up 18 ± 4 months (14–24 months) [46], and the other, also a clinical trial, reported a CR of 83% (5/6) during median follow up 244 ± 55 (Range 170–310) days [26]. For TACE, one study reported an OR of 46% (6/13), CR of 31% (4/13), PR of 15% (2/13), PD of 15% (2/13), and SD of 39% (5/13) at median 14 months (range: 1–77) [47]. The other reported a CR of 46% (109/237) at median 31.9 months (interquartile range: 15.9–44. [48]. Both were retrospective analyses. S7 and S8 Tables include descriptive tumor response results for the IRE and TACE reviews.

#### Progression free survival.

PFS outcomes were reported in 4 studies across both reviews (1 IRE, 3 TACE) [37,39,49,50]. In the only study reporting PFS among early-stage patients treated with IRE, PFS among 17 patients was a median 10 months (range: 3–30 months) [39]. Two TACE studies also reported median time to PFS, with results ranging from 19 months (95% CI 15.101–22.899) among 55 early-stage (BCLC Stage A) patients [50] to 30 months among 30 early-stage (BCLC Stage A) patients [49]. One TACE study reported mean time to PFS: mean 6.8 months (range: 1.1–27.5 months) among 52 early-stage DEB-TACE patients [51]. Full details on PFS results can be found in S9 and S10 Tables.

#### Adverse events.

Nine IRE studies (75%) and 12 TACE studies (36%) reported AEs and serious adverse events (SAEs) in early-stage HCC patients (Table 6 and S11 Table). While AE occurrence was assessed with various grading scales, including Common Terminology Criteria for Adverse Events (CTCAE), Society of Interventional Radiology (SIR), Cardiovascular and Interventional Radiological Society of Europe (CIRSE) guidelines, and Clavien-Dindo, some studies did not use an AE grading system. There was also variation in the way studies reported safety events with some reporting the number and proportion of patients experiencing an event and others reporting the absolute number of events.

**Table 6:** Summary of adverse event reporting, IRE and TACE reviews.

AE Grade/ Severity	IRE			TACE		
**N Studies (%)** ^a^	**N Patients (%)**	**N Events** ^b^	**N Studies (%)** ^a^	**N Patients (%)**	**N Events** ^b^
All-Grade AEs (I–IV)	9 (75)	21/172 (12)	14	13 (38)	144/1,350 (11)	210
Grade I/II or Mild	6 (50)	17/70 (24)	10	5 (15)	101/437 (23)	142
Grade III/IV/V or Moderate-Severe	8 (67)	4/102 (4)	4	10 (30)	42/864 (5)	68

Abbreviations: IRE, irreversible electroporation; TACE, transarterial chemoembolization; AE, adverse event; SAE, serious adverse event

^a^Some studies reported both AEs and SAEs

^b^For studies that only reported number of AE/SAE events, it was not possible to ascertain the proportion of patients who experienced these events (e.g., the same patient may have experienced multiple events).

Most AEs were Grade I/II or mild and included increases in blood pressure [26], fevers [52], and post embolization syndrome [53,54]. AE and SAE rates were comparable between the IRE and TACE, with 24% of IRE and 23% of TACE patients experienced mild AEs, respectively. SAEs were experienced by 4% of IRE patients compared to 5% of TACE patients. Among studies that reported the number of SAE events, 4 SAEs were reported following IRE treatment, and 68 SAEs were reported following TACE treatment. No Grade V or Death events were reported among IRE patients, while 4 were reported among TACE patients (including 2 deaths).

#### Quality and risk of bias.

Overall, the quality and risk of bias in both SLRs was acceptable, as depicted in Table 7. The 2 TACE studies that were determined to have increased risk of bias and were assigned a poor rating were rated low due to a lack of methodological specificity that made it difficult to ascertain study methodology and rigor. Most studies across the reviews were found to be low or very low quality (IRE n=9, TACE n=18). This can be attributed to the prevalence of observational studies that lacked the random design necessary to control for complicating factors. Further details on ratings can be found in S12–S17 Tables.

**Table 7:** Risk of bias and study quality, IRE and TACE reviews.

	IRE	TACE
Quality Rating, n studies		
High	0	4
Moderate	3	11
Low	7	15
Very Low	2	3
Risk of Bias Rating, n studies		
Good	3	7
Fair	9	24
Poor	0	2

Abbreviations: IRE, irreversible electroporation; TACE, transarterial chemoembolization

## Discussion

Due to increased screening of at-risk patients, HCC is commonly being diagnosed at earlier stages [5]. When surgical resection or liver transplantation are not feasible due to the severity of underlying conditions or comorbidities [55], local ablative techniques have become the standard of care for these early-stage patients [56]. Findings from this research provide novel insight into the comparative clinical effectiveness and safety of IRE and TACE for the ablation of unresectable, very early and early-stage HCC.

Meta-analyses indicate that both IRE and TACE reliably elicit an objective tumor response short-term post-procedure in most patients. Both modalities were efficacious at achieving CR, that is, the disappearance of all target lesions, particularly during the first few months after treatment (0 to < 3 months), with higher rates observed among IRE versus TACE. IRE CR rates in the present study were similar to those observed in a comparative study in early HCC involving other ablation modalities, which reported CR rates of 87.5% for MWA and 84.1% for RFA [57,58]. As follow-up periods approached 6 months for TACE, disease recurrence rates began to rise; however, notably, both OR and CR increased for IRE at 3 to <6 months follow-up, suggesting a robust, short-term duration of effect. A slight rebound effect was observed for TACE CR between 6 and 12 months (>50%), however, since observations for longer follow-up time points were based on a small number of studies (with data limitation precluding 6+ month meta-analysis for IRE), caution should be used when drawing inferences. Only a small fraction of patients (<10%) experienced disease progression after treatment with either IRE or TACE in the short-term (0 to < 6 months). Sub-analyses suggest potentially greater tumor response outcomes (OR, CR) among cTACE versus DEB-TACE, however, further research is needed to investigate TACE modality-specific benefits. Overall results demonstrate the short-term effectiveness of these modalities, especially IRE, for disease control and treatment. Descriptive analyses of 12+ month tumor response findings indicate that IRE may yield more robust, longer term treatment effects than TACE, however, results were based on few studies, most of which had small samples, so additional research is required.

Among the 3 studies that reported median PFS, patients treated for early-stage HCC with TACE experienced much longer median PFS times compared to IRE; however, the limited data available on PFS makes it difficult to draw inferences from these findings.

AEs were reported inconsistently across trials and could not be quantitatively analyzed due to variation in measurement and assessment. While incidence reporting for safety events was inconsistent across both SLRs, more IRE studies reported on early-stage AEs and SAEs than TACE, which may be due to TACE studies more infrequently delineating AE and SAE outcomes by disease stage. Additionally, IRE studies reported lower rates of AEs and SAEs compared to TACE studies. Generally, severe complications were uncommon, especially in IRE, while mild events related to post-operative discomfort including fever, abdominal pain, and minor bleeding were normative for both modalities. Documented IRE complications were consistent with device labeling [59]. Similar to findings in this study, AE rates have varied considerably across studies investigating techniques for local ablation, including IRE, TACE, TACE+MWA, transarterial radioembolization (TARE), and RFA/MWA. For example, recent studies have reported common AEs occurring in 3% to 35% of patients treated with TACE alone or TACE+MWA, and SAE rates have been documented to be as high as 53% in patients treated with DEB-TACE [60–62]. A comparative study between IRE and RFA/MWA found that IRE had higher low-grade AE rates compared to RFA/MWA, with 34% of IRE patients and 27% of RFA/MWA patients experiencing AEs, respectively, but similar SAE rates of 2.1% for IRE and 3.4% for RFA/MWA [63–65].

### Limitations

While the SLRs and meta-analyses conducted represent the current body of evidence available on the use of IRE and TACE technologies in very early and early-stage HCC, the process of reviewing and screening literature for inclusion in the final review inherently introduces subjectivity. Decisions on article eligibility and screening criteria reduce the number of full-text articles reviewed to those most salient, and thus, some key studies may have been omitted from the analysis. The use of dual reviewers helped mitigate accidental omissions, as each title/abstract was reviewed twice independently, with a third reviewer for adjudication. While publications that included patient cohorts with distinct overlap were eliminated to avoid double-counting, it is possible that the final reviews included publications with overlapping samples. Further, additional searches beyond the PubMed and Google Scholar databases (such as Embase or other medical databases) may have identified additional research. Despite these limitations, a robust set of articles was reviewed, and the SLRs were conducted in a systematic, rigorous manner in alignment with PRISMA and AMSTAR 2 guidelines and using independent reviewers to minimize bias.

There are also notable methodological limitations of published studies on IRE and TACE treatment for early-stage HCC, which is reflective of this emergent literature base. Most studies included in the SLRs and meta-analyses were observational, which may have introduced bias. Additionally, the small overall sample sizes for patients with early-stage HCC and limited or varied reporting on select outcomes, including PFS and AEs, restricted suitability for quantitative analyses. This limits the ability to generalize the results. Small sample sizes for select tumor response meta-analyses, such as IRE stable disease, where minimal events were observed, similarly may limit generalizability of findings. There was significant heterogeneity among the studies that were included in meta-analyses. This required the use of a random effects model for combined estimates. Nevertheless, the fixed and random effects estimates were generally similar for all outcomes that were analyzed. Meta-analysis of tumor response at 6+ month follow-up could also not be reliably reported due to the small number of studies that reported details from follow-up imaging. Furthermore, since patients were generally of an older age across studies included, results may not be generalizable to younger patient populations with early-stage disease. Additionally, since most studies were conducted in Europe and Asia, variation in treatment paradigms and patient populations limit generalizability to other settings and patient cohorts.

Given the paucity of PFS data among published studies, further investigation into disease progression after ablative treatment is recommended. Additionally, standardized assessment of AEs associated with these modalities would strengthen the evidence base surrounding their safety profile. Future studies should also prioritize longitudinal follow-up to examine key effectiveness outcomes over the medium-longer term in very early and early-stage, unresectable HCC to better ascertain the durability of the response to TACE and IRE.

## Conclusions

While TACE is a long-established standard in locoregional treatment for unresectable, early-stage HCC [66], IRE has emerged in recent years as a newer ablation option due to its minimally invasive properties and avoidance of thermal complications [67–69]. Findings from our SLRs and meta-analyses confirmed that both IRE and TACE are safe and effective non-thermal treatments for unresectable, very early and early-stage HCC. Notably, while results support the effectiveness of both therapies in eliciting a complete tumor response in this patient population, CR was more frequently achieved with IRE versus TACE. Although there are inherent risks and side effects to both procedures, each technique is generally safe, even in relatively older populations. Overall, findings may support the broader adoption of IRE as a procedural option into the standard of care for patients with early stage, unresectable HCC. While results are promising, additional research is recommended given the limited data available longer-term and for select outcomes important for the treatment of HCC.

## Supporting information

S1 Table Search terms. (DOCX)

S2 Table BCLC classification system. (DOCX)

S3 Table Outcomes of interest, IRE and TACE reviews. (DOCX)

S4 Table I^2^ Measurement interpretation. (DOCX)

S5 Table Select study and very early/early-stage patient characteristics, IRE SLR. (DOCX)

S6 Table Select study and very early/early-stage patient characteristics, TACE SLR. (DOCX)

S7 Table Very early/early-stage tumor response results, IRE SLR. (DOCX)

S8 Table Very early/early-stage tumor response results, TACE SLR. (DOCX)

S9 Table Very early/early-stage progression free survival results, IRE SLR. (DOCX)

S10 Table Very early/early-stage progression free survival results, TACE SLR. (DOCX)

S11 Table Adverse events reported, IRE and TACE reviews. (DOCX)

S12 Table IRE risk of bias assessment for observational studies (n=8). (DOCX)

S13 Table IRE risk of bias assessment for clinical trials (n=4). (DOCX)

S14 Table TACE risk of bias assessment for observational studies (n=28). (DOCX)

S15 Table TACE risk of bias assessment for clinical trials (n=5). (DOCX)

S16 Table IRE GRADE assessment. (DOCX)

S17 Table TACE GRADE assessment. (DOCX)

S18 Table All unique studies identified, IRE SLR. (DOCX)

S19 Table All unique studies identified, TACE SLR. (DOCX)

S1 Fig Forest plot, IRE PR results at 1 month. (PDF)

S2 Fig Forest plot, IRE PD results at 1 month. (PDF)

S3 Fig Forest plot, IRE SD results at 1 month. (PDF)

S4 Fig Forest plot, IRE PR results at 3 months. (PDF)

S5 Fig Forest plot, TACE PR results at 1 month. (PDF)

S6 Fig Forest plot, TACE PD results at 1 month. (PDF)

S7 Fig Forest plot, TACE SD results at 1 month. (PDF)

S8 Fig Forest plot, TACE PR results, 3 to < 6 months. (PDF)

S9 Fig Forest plot, TACE PD results, 3 to < 6 months. (PDF)

S10 Fig Forest plot, TACE SD results, 3 to < 6 months. (PDF)
