# Supplementary material for: Clinical and safety outcomes in unresectable, very early and early-stage hepatocellular carcinoma following Irreversible Electroporation (IRE) and Transarterial Chemoembolization (TACE): A systematic literature review and meta-analysis
Source: PLoS One. 2025 Apr 29;20(4):e0322113. doi: 10.1371/journal.pone.0322113 (PMC12083900; doi:10.1371/journal.pone.0322113)
Supplement: S1 Table — (DOCX) [file pone.0322113.s001.docx]

# S1 Table. Search Terms

| **Irreversible Electroporation (IRE) PubMed Search Terms** | 1. (((liver cancer) OR (liver neoplasms)) OR (hepatocellular carcinoma) AND (irreversible electroporation) 2. hepatocellular carcinoma AND irreversible electroporation 3. ((carcinoma, hepatocellular[MeSH Terms]) AND (electroporation[MeSH Terms]) |
| --- | --- |
| **Transarterial Chemoembolization (TACE) PubMed Search Terms** | 1. unresectable hepatocellular carcinoma AND ((transarterial chemoembolization) OR (TACE)) 2. (((unresectable) AND (Carcinoma, Hepatocellular[MeSH Terms]) OR (unresectable hepatocellular carcinoma)) AND ((transarterial chemoembolization) OR (TACE) OR Chemoembolization, Therapeutic [MeSH Terms])) AND (((treatment outcome[MeSH Terms]) OR (progression free survival)) OR (tumor response)) |
