# Supplementary material for: Clinical and safety outcomes in unresectable, very early and early-stage hepatocellular carcinoma following Irreversible Electroporation (IRE) and Transarterial Chemoembolization (TACE): A systematic literature review and meta-analysis
Source: PLoS One. 2025 Apr 29;20(4):e0322113. doi: 10.1371/journal.pone.0322113 (PMC12083900; doi:10.1371/journal.pone.0322113)
Supplement: S2 Table — (DOCX) [file pone.0322113.s002.docx]

# S2 Table. BCLC Classification System

| BCLC Stage | Criteria |
| --- | --- |
| Stage 0  (Very early stage)* | - Tumor is less than 2 cm - PS 0 - Liver is working normally (Child-Pugh A) |
| Stage A  (Early-stage)* | - Single tumor of any size, or up to 3 tumors all less than 3 cm - PS 0 - Liver is working normally or mild damage (Child-Pugh A or B). |
| Stage B  (Intermediate stage) | - Many tumors in the liver - PS 0 - Liver is working normally or mild damage (Child-Pugh A or B) |
| Stage C  (Advanced stage) | - Cancer has spread into the blood vessels, lymph nodes or other body organs - PS 1 or 2 - Liver is working normally or mild damage (Child-Pugh A or B) |
| Stage D  (End-stage) | - Many tumors - PS 3 or PS 4 - Severe liver damage (Child-Pugh C) |
| Abbreviations: BCLC, Barcelona Clinic Liver Cancer; cm, centimeters; PS, Performance Status  *BCLC stage met inclusion criteria for IRE and TACE reviews | |
