# Supplementary material for: Clinical and safety outcomes in unresectable, very early and early-stage hepatocellular carcinoma following Irreversible Electroporation (IRE) and Transarterial Chemoembolization (TACE): A systematic literature review and meta-analysis
Source: PLoS One. 2025 Apr 29;20(4):e0322113. doi: 10.1371/journal.pone.0322113 (PMC12083900; doi:10.1371/journal.pone.0322113)
Supplement: S3 Table — (DOCX) [file pone.0322113.s003.docx]

# S3 Table. Outcomes of Interest, IRE and TACE Reviews

| Outcome | Operational Definition |
| --- | --- |
| Progression Free Survival | Interval between treatment initiation and death or the date of local tumor progression at post-therapeutic imaging. |
| Tumor Response | Tumor response was evaluated using either the Response Evaluation Criteria in Solid Tumors (RECIST) or modified RECIST (mRECIST) criteria. Tumor responses evaluated via RECIST are categorized as follows [25]:   - **Complete Response (CR):** the disappearance of all target lesions - **Partial Response (PR)**: >30% decrease in the sum of the longest diameter of the target lesion compared to baseline - **Objective Response (OR)**: either PR or CR - **Progressive Disease (PD)**: the appearance of 1 or more new lesions or >20% size increase - **Stable Disease (SD)**: neither PR nor PD |
| Adverse Events | AEs were evaluated by established grading systems such as the Common Terminology Criteria for Adverse Events (CTCAE) or the Clavien-Dindo classification system. These systems assign grades, typically I-V, based on severity of event. Less serious AEs are lower grades (grades I and II) and more serious adverse events (SAEs) with life-threatening consequences are graded higher (grades III and higher).  When a classification system was not used, AEs were categorized as mild (e.g., low-grade fever, nausea, dizziness, etc.) or severe (e.g., hemorrhaging, organ failure, death). |
