# Supplementary material for: Clinical and safety outcomes in unresectable, very early and early-stage hepatocellular carcinoma following Irreversible Electroporation (IRE) and Transarterial Chemoembolization (TACE): A systematic literature review and meta-analysis
Source: PLoS One. 2025 Apr 29;20(4):e0322113. doi: 10.1371/journal.pone.0322113 (PMC12083900; doi:10.1371/journal.pone.0322113)
Supplement: S4 Table — (DOCX) [file pone.0322113.s004.docx]

# S4 Table. I^2^ Measurement Interpretation

| $\boldsymbol{I}^{\boldsymbol{2}}$ | Significance |
| --- | --- |
| 0%-40% | Might not be important |
| 30%-60% | May represent moderate heterogeneity |
| 50%-90% | May represent substantial heterogeneity |
| 75%-100% | Considerable heterogeneity |
