# Supplementary material for: Clinical and safety outcomes in unresectable, very early and early-stage hepatocellular carcinoma following Irreversible Electroporation (IRE) and Transarterial Chemoembolization (TACE): A systematic literature review and meta-analysis
Source: PLoS One. 2025 Apr 29;20(4):e0322113. doi: 10.1371/journal.pone.0322113 (PMC12083900; doi:10.1371/journal.pone.0322113)
Supplement: S5 Table — (DOCX) [file pone.0322113.s005.docx]

# S5 Table. Select Study and Very Early/Early-Stage Patient Characteristics, IRE SLR

| **First Author, Year** | **Intervention** | **Comparator** | **Study Design** | **Study Location** | **Sample Size (n)** | **Mean Age (years)** | **Mean Number of Tumors** | **Mean Tumor Size (cm)** |
| --- | --- | --- | --- | --- | --- | --- | --- | --- |
| Cheung W, 2013 | IRE | None | Clinical Trial (non-randomized) | Monash University, Australia | 9 | 72 | 1.3 | 2.4 |
| Fang C, 2021 | IRE | None | Retrospective Observational | King’s College Hospital, United Kingdom | 19 | 59.5 | NR | 2.4 |
| Freeman E, 2021 | IRE | None | Retrospective Observational | Alfred Hospital, Australia | 28 tumors (4 BCLC 0, 24 BCLC A) | NR | NR | NR |
| Frühling P, 2017 | IRE | None | Clinical Trial (non-randomized) | Uppsala University Hospital, Sweden | 8 (8 tumors) | 66.13 | 1.0 | 2.0 |
| Granata V, 2016 | IRE | None | Prospective Observational | National Cancer Institute, Italy | 20 (24 tumors) | 65.45 | 1.2 | 2.0 |
| Kalra N, 2019 | IRE | None | Retrospective Observational | India | 17 | NR | NR | NR |
| Lencioni R, 2012 | IRE | None | Clinical Trial (non-randomized) | L’institut de cancerologie Gustave Roussy, Hopital Beaujon, France,  Universitätsklinikum Magdeburg AoR Klinik für Radiologie und Nuklearmedizin, Germany,  University of Pisa School of Medicine, Istituto Nazionale Tumori - Fondazione Pascale, Italy,  Barcelona Clinic Liver Cancer Group Hospital Clinic i Provincial de Barcelona, Spain | 26 (29 tumors) | NR | 1.1 | NR |
| Padia SA, 2016 | IRE | None | Retrospective Observational | University of Washington, United States | 20 | NR | 1.0 | NR |
| Pan F, 2021 | IRE | None | Retrospective Observational | University Hospital Heidelberg, Germany | 9 (11 tumors) | 67 | 1.2 | 1.5 |
| Sugimoto K, 2015 | IRE | None | Clinical Trial (non-randomized) | Tokyo Medical University, Japan | 5 | 66.6 | 1.2 | 1.8 |
| Thamtorawat S, 2022 | IRE | RFA | Retrospective Observational | Thailand | 13 | 69.2 | 1.0 | 1.7 |
| Wada T, 2023 | IRE | RFA, MWA | Retrospective Observational | Japan (Tokyo Medical University) | 15 | 73.8 | 1.5 | 1.4 |

Abbreviations: RFA, radiofrequency ablation; MWA, microwave ablation; SLR, systematic literature review; BCLC, Barcelona Clinic Liver Cancer; cm, centimeters; NR, not reported
