# Supplementary material for: Clinical and safety outcomes in unresectable, very early and early-stage hepatocellular carcinoma following Irreversible Electroporation (IRE) and Transarterial Chemoembolization (TACE): A systematic literature review and meta-analysis
Source: PLoS One. 2025 Apr 29;20(4):e0322113. doi: 10.1371/journal.pone.0322113 (PMC12083900; doi:10.1371/journal.pone.0322113)
Supplement: S6 Table — (DOCX) [file pone.0322113.s006.docx]

# S6 Table. Select Study and Very Early/Early-Stage Patient Characteristics, TACE SLR

| First Author, Year | Intervention | Comparator | Study Design | Study Location | Sample Size (n) | Mean Age (years) | Mean Number of Tumors | Mean Tumor Size (cm) |
| --- | --- | --- | --- | --- | --- | --- | --- | --- |
| Alan A, 2023 | DEB-TACE | None | Retrospective Observational | Turkey (Marmara University) | 16 | NR | NR | NR |
| Bai M, 2019 | PE-TACE | None | Retrospective Observational | The Third Affiliated Hospital, Sun Yat-Sen University; Ling-nan Hospital, Sun Yat-Sen University, China | 7 | NR | NR | NR |
| Bargellini I, 2012 | TACE | None | Prospective Observational | University of Pisa, Italy | 67 | 70 | 1.5 | 3.7 |
| Cathomas M, 2023 | DEB-TACE | TAE | Retrospective Observational | Switzerland (Bern University Hospital) | 94 (52 DEB-TACE, 42 TAE) | NR | 1.3 | NR |
| Chen RX, 2016 | TACE | RFA | Retrospective Observational | Zhongshan Hospital, Fudan University, China | 443 (208 TACE, 235 RFA) | NR | NR | NR |
| Chen S, 2018 | Lipiodol TACE | GSP-TACE | Retrospective Observational | Fujian Cancer Hospital & Fujian Medical University Cancer Hospital, China | 14 (9 lipiodol, 5 GSP) | NR | NR | NR |
| Chu HH, 2020 | Microsphere TACE | Cisplatin TACE | Retrospective Observational | South Korea | 359 | NR | NR | NR |
| Golfieri R, 2014 | cTACE | DEB-TACE | RCT | Italy | 82 | NR | NR | NR |
| Hashem E, 2023 | TACE | None | Retrospective Observational | UK (King’s College Hospital) | 237 | 63 | NR | NR |
| Haubold J, 2020 | DSM-TACE | None | Retrospective Observational | University Hospital Essen, Germany | 8 | NR | NR | NR |
| Huo YR, 2019 | DEB-TACE | None | Retrospective Observational | Australia (Concord Repatriation General Hospital) | 30 (19 70-150um, 11 100-300um) | NR | NR | NR |
| Hyun D, 2016 | TACE | TACE+RFA | Retrospective Observational | South Korea (Samsung Medical Center) | 91 (54 TACE, 37 TACE + RFA) | 59.5 (TACE), 57.7 (TACE + RFA) | NR | NR |
| Iezzi R, 2019 | DEB-TACE | DEB-TACE+RFA | Prospective Observational | Italy (The Gemelli University Hospital) | 159 (16 DEB-TACE, 143 DEB-TACE + RFA) | 63.1 (DEB-TACE), 69.3 (DEB-TACE + RFA) | 1 | 4.2 |
| Ikeda M, 2022 | cTACE | DEB-TACE | RCT | Japan (multiple institutions) | Total: 120 (DEB-TACE: 57, cTACE: 63) | NR | NR | NR |
| Imai Y, 2012 | TACE | None | Prospective Observational | Japan (Saitama Medical University Hospital) | 27 | NR | NR | NR |
| Jiang JQ, 2023 | TACE | None | Retrospective Observational | China (multiple hospitals) | 369 | NR | NR | NR |
| Kim JW, 2014 | TACE | RFA | Retrospective Observational | South Korea (Asan Medical Center) | 287 (122 TACE, 165 RFA) | 56.3 | 1 | 1.6 |
| Kudo M, 2020 | TACE | TACE + sorafenib | RCT | Japan | 60 (33 TACE, 27 TACE + sorafenib) | NR | NR | NR |
| Lee M, 2017 | DEE-TACE | None | Retrospective Observational | South Korea (university hospitals) | 88 | NR | NR | NR |
| Lee M, 2023 | TACE | None | Retrospective Observational | Republic of Korea (Kyung Hee University Hospital at Gangdong) | 97 | 63.47 | NR | NR |
| Lee YK, 2017 | cTACE | DEB-TACE | Prospective Observational | South Korea (Yonsei University College of Medicine) | 69 (49 cTACE, 20 DEB-TACE) | NR | NR | NR |
| Manini MA, 2015 | DEB-TACE | None | Retrospective Observational | Italy (Ca' Granda Ospedale Maggiore Policlinico) | 55 (79 tumors) | NR | 1 | NR |
| Mendez Romero A, 2023 | DEB-TACE | Stereotactic Body Radiation Therapy (SBRT) | RCT | Netherlands and Belgium | 16 | NR | NR | NR |
| Ou HY, 2020 | DEE-TACE | None | Prospective Observational | Taiwan (Kaohsiung Chang Gung Memorial Hospital) | 51 | NR | NR | NR |
| Rahman A, 2016 | cTACE | DEB-TACE | Retrospective Observational | Malaysia (National University of Malaysia Medical Center) | 20 (11 cTACE, 9 DEB-TACE) | NR | NR | NR |
| Razi M, 2022 | cTACE | DEB-TACE | Retrospective Observational | China (Nanjing First Hospital) | 40 (20 cTACE, 20 DEB-TACE) | 57.43 (cTACE), 56.4 (DEB-TACE) | 1 | NR |
| Sheta E, 2016 | TACE | TACE + RFA | RCT | Egypt | 50 | NR | 1 | 4.8 |
| Song MJ, 2012 | cTACE | DEB-TACE | Retrospective Observational | South Korea (Catholic University Medical Center of Korea) | 55 (27 DEB-TACE, 28 cTACE) | NR | NR | NR |
| Tay B, 2022 | TACE | RFA | Retrospective Observational | Singapore (National University Health System) | 62 | NR | NR | NR |
| Tovar-Felice G, 2021 | DEM-TACE | None | Retrospective Observational | Spain (German Trias I Pujol University Hospital) | 12 | NR | NR | NR |
| Yun BY, 2020 | TACE | RFA | Retrospective Observational | Korea (Severance Hospital) | 404 (268 TACE, 136 RFA) | 69.3 TACE, 69.1 RFA | NR | 2.8 TACE, 2.0 RFA |
| Zhang L, 2021 | cTACE | DEB-TACE | Retrospective Observational | China (Soochow University, Zhejiang University School of Medicine, Wenzhou Medical University) | 118 (67 cTACE, 51 DEB-TACE) | NR | NR | NR |
| Zhang L, 2023 | TACE | None | Retrospective Observational | China (Tongji Medical College) | 55 | NR | NR | NR |
| Abbreviations: TACE, transarterial chemoembolization; SLR, systematic literature review; PE-TACE; pirarubin-eluting transarterial chemoembolization; cTACE, conventional transarterial chemoembolization; DSM-TACE, degradable starch microspheres transarterial chemoembolization; DEB-TACE, drug-eluting bead transarterial chemoembolization; DEE-TACE, drug-eluting embolic transarterial chemoembolization; DEM-TACE, drug-eluting microsphere transarterial chemoembolization; RFA, radiofrequency ablation; GSP-TACE, gelatin sponge particle transarterial chemoembolization; MWA, microwave ablation; NR, not reported | | | | | | | |  |
