# Supplementary material for: Clinical and safety outcomes in unresectable, very early and early-stage hepatocellular carcinoma following Irreversible Electroporation (IRE) and Transarterial Chemoembolization (TACE): A systematic literature review and meta-analysis
Source: PLoS One. 2025 Apr 29;20(4):e0322113. doi: 10.1371/journal.pone.0322113 (PMC12083900; doi:10.1371/journal.pone.0322113)
Supplement: S7 Table — (DOCX) [file pone.0322113.s007.docx]

# S7 Table. Very Early/Early-Stage Tumor Response Results, IRE SLR

| First Author, Year | Study Design | Follow-Up 1 Time Point | Follow-Up 1 Rate | Follow-Up 2 Time Point | Follow-Up 2 Rate | Follow-Up 3 Time Point | Follow-Up 3 Rate |
| --- | --- | --- | --- | --- | --- | --- | --- |
| Cheung W, 2013 | Clinical Trial (non-randomized) | Mean 18 ± 4 months (14-24 months) | CR: 8/12, 67% | NR | NR | NR | NR |
| Fang C, 2021 | Retrospective Observational | 3 months | OR: 19/19, 100%  CR: 17/19, 90%  PR: 2/19, 10%  PD: 0  SD: 0 | NR | NR | NR | NR |
| Freeman E, 2021 | Retrospective Observational | 1 month | CR: 13/14, 93% | NR | NR | NR | NR |
| Frühling P, 2017 | Clinical Trial (non-randomized) | 1 month | OR: 8/8, 100%  CR: 8/8, 100%  PR: 0  PD: 0  SD: 0 | 3 months | OR: 8/8, 100%  CR: 8/8, 100%  PR: 0  PD: 0  SD: 0 | NR | NR |
| Granata V, 2016 | Prospective Observational | 1 month | OR: 24/24, 100%  CR: 22/24, 92%  PR: 2/24, 8%  PD: 0 | 3 months | OR: 22/24, 92%  CR: 22/24, 92%  PR: 0  PD: 2/24,8% | 6 months | OR: 22/24, 92%  CR: 22/24, 92%  PR: 0  PD: 2/24, 8% |
| Kalra N, 2019 | Retrospective Observational | 1 month | OR: 17/17, 100%  CR: 13/17, 76%  PR: 4/17, 24%  PD: 0  SD: 0 | NR | NR | NR | NR |
| Lencioni R, 2012 | Clinical Trial (non-randomized) | 1 month | OR: 24/26, 92%  CR: 20/26, 77%  PR: 4/26, 15%  PD: 1/26, 4%  SD: 1/26, 4% | NR | NR | NR | NR |
| Padia SA, 2016 | Retrospective Observational | 1 month | OR: 20/20, 100%  CR: 18/20, 90%  PR: 2/20, 10%  PD: 0  SD: 0 | NR | NR | NR | NR |
| Sugimoto K, 2015 | Clinical Trial (non-randomized) | Median 244 ± 55 (Range 170-310) days | CR: 5/6, 83% | NR | NR | NR | NR |
| Abbreviations: IRE, irreversible electroporation; SLR, systematic literature review; CR, complete response; OR, objective response; PR, partial response; PD, progressive disease; SD, stable disease; NR, not reported | | | | | | | |
|  | | | | | | | |
