# Supplementary material for: Clinical and safety outcomes in unresectable, very early and early-stage hepatocellular carcinoma following Irreversible Electroporation (IRE) and Transarterial Chemoembolization (TACE): A systematic literature review and meta-analysis
Source: PLoS One. 2025 Apr 29;20(4):e0322113. doi: 10.1371/journal.pone.0322113 (PMC12083900; doi:10.1371/journal.pone.0322113)
Supplement: S8 Table — (DOCX) [file pone.0322113.s008.docx]

# S8 Table. Very Early/Early-Stage Tumor Response Results, TACE SLR

| First Author | Study Design | Follow-Up 1 Time Point | Follow-Up 1 Rate | Follow-Up 2 Time Point | Follow-Up 2 Rate | Follow-Up 3 Time Point | Follow-Up 3 Rate |
| --- | --- | --- | --- | --- | --- | --- | --- |
| Alan A, 2023 | Retrospective Observational | Median 14 months (range: 1-77) | OR: 6/13, 46%  CR: 4/13, 31%  PR: 2/13, 15%  PD: 2/13, 15%  SD: 5/13, 39% | NR | NR | NR | NR |
| Bai M, 2019 | Retrospective Observational | 1 month | OR: 7/7, 100%  CR: 6/7, 86%  PR: 1/7, 14%  PD: 0  SD: 0 | NR | NR | NR | NR |
| Bargellini I, 2012 | Prospective Observational | 1 month | OR: 65/67, 97%  CR: 45/67, 67%  PR: 20/67, 30%  PD: 2/67, 3% | NR | NR | NR | NR |
| Cathomas M, 2023 | Retrospective Observational | 1-3 months | OR: 70/72, 97%  CR: 33/72, 46%  PR: 37/72, 50%  PD: 1/72, 1.4%  SD: 2/72, 2.8% | NR | NR | NR | NR |
| Chen S, 2018 | Retrospective Observational | 1 month | GSP: OR 3/5, 60%  Lipiodol: OR: 6/9, 66.7% | NA | NR | NR | NR |
| Chu HH, 2020 | Retrospective Observational | 1 month | OR: 319/359, 89% | NR | NR | NR | NR |
| Golfieri R, 2014 | Retrospective Observational | 1 month | cTACE: CR: 32/40, 80%  DEB-TACE: CR: 20/41, 49% | 3 months | cTACE: CR 26/40, 65%  DEB-TACE: CR 21/36, 58% | 6 months | cTACE: CR 27/39, 69%  DEB-TACE: CR 16/31, 52% |
| Hashem E, 2023 | RCT | Median 31.9 months (interquartile range: 15.9-44.7) | CR: 109/237 (45.6%) | NR | NR | NR | NR |
| Haubold J, 2020 | Retrospective Observational | NR | OR: 4/8, 50%  CR: 2/8, 25%  PR: 2/8, 25%  PD: 1/8, 13%  SD: 3/8, 38% | NR | NR | NR | NR |
| Hyun D, 2016 | Retrospective Observational | 1 month | OR: 49/54, 91%  CR: 31/54, 57%  PR: 18/54, 33%  PD: 0  SD: 5/54, 9% | NR | NR | NR | NR |
| Iezzi R, 2019 | Retrospective Observational | 1 month | OR: 15/16, 94%  CR: 5/16, 31%  PR: 10/16, 63%  PD: 0  SD: 1/16, 6% | NR | NR | NR | NR |
| Ikeda M, 2022 | Retrospective Observational | 3 months | DEB-TACE: CR 17/57, 29.7%  cTACE: CR 49/63, 78.1% | NR | NR | NR | NR |
| Imai Y, 2012 | Prospective Observational | 1 month | OR: 14/14, 100%  CR: 10/14, 71%  PR: 4/14, 29%  PD: 0  SD: 0 | NR | NR | NR | NR |
| Jiang JQ, 2023 | RCT | 3-4 months | OR: 234/369, 63.4%  CR: 102/369, 27.8% | NR | NR | NR | NR |
| Kim JW, 2014 | Prospective Observational | 1 month | OR: 117/122, 96%  CR: 92/122, 75%  PR: 25/122, 20%  PD: 1/122, 1%  SD: 4/122, 3% | NR | NR | NR | NR |
| Lee M, 2017 | Retrospective Observational | 1 month | OR: 80/88, 91%  CR: 47/88, 53%  PR: 33/88, 38%  PD: 1/88, 1%  SD: 7/88, 8% | 6 months | OR: 48/72, 67%  CR: 40/72, 56%  PR: 8/72, 11%  PD: 23/72, 32% | NR | NR |
| Lee M, 2023 | Retrospective Observational | 1 month | CR: 84/97, 86.6% | NR | NR | NR | NR |
| Lee YK, 2017 | RCT | NR | cTACE:  OR: 43/49, 88%  CR: 31/49, 63%  PR: 12/49, 25%  DEB-TACE:  OR: 15/20, 75%  CR: 12/20, 60%  PR: 3/20, 15% | NR | NR | NR | NR |
| Manini MA, 2015 | Retrospective Observational | 1 month | OR: 46/55, 84%  CR: 29/55, 53%  PR: 17/55, 31%  PD: 4/55, 7%  SD: 5/55, 9% | 4 months | CR: 21/55, 38% | 7 months | CR: 17/55, 31% |
| Mendez Romero A, 2023 | Retrospective Observational | NR | OR: 13/16, 81%  CR: 9/16, 56%  PR: 4/16, 25% | NR | NR | NR | NR |
| Ou HY, 2020 | Prospective Observational | 1 month | CR: 31/51, 61% | NR | NR | NR | NR |
| Rahman A, 2016 | Retrospective Observational | 3 months | OR: DEB 6/9, 67%  cTACE 5/11, 45%  CR: DEB 2/9, 22%  cTACE 2/11, 18%  PR: DEB 4/9, 44%  cTACE 3/11, 27%  PD: DEB 1/9, 11%  cTACE 4/11, 36%  SD: DEB 2/9, 22%  cTACE 2/11, 18% | NR | NR | NR | NR |
| Razi M, 2022 | RCT | Immediate | cTACE:  OR: 19/20, 95%  CR: 16/20, 81.5%  PR: 3/20, 13%  PD: 1/20, 5.5%  SD: 0/20, 0  DEB-TACE:  OR: 19/20, 96.5%  CR: 15/20, 77.5%  PR: 4/20, 18.5%  PD: 1/20, 4%  SD: 0/20, 0 | 1 year | cTACE:  OR: 17/20, 85.2%  CR: 16/20, 79.5%  PR: 1/20, 5.5%  PD: 2/20, 10%  SD: 1/20, 5.5%  DEB-TACE:  OR: 16/20, 81.5%  CR: 15/20, 77.3%  PR: 1/20, 3.5%  PD: 2/20, 12%  SD: 1/20, 7% | NR | NR |
| Sheta E, 2016 | Prospective Observational | 1 month | CR: 10/20, 50% | 3 months | CR: 10/20, 50% | 6 months | CR: 10/20, 50% |
| Song MJ, 2012 | Retrospective Observational | 3 months | cTACE: OR: 20/28, 71% CR: 13/28, 46%  PR: 7/28, 25%  SD: 5/28, 18% PD: 3/28, 11%   DEB-TACE: OR: 24/27, 89% CR: 21/27, 78%  PR: 3/27, 11%  SD: 2/27, 7%  PD: 1/27, 4% | NR | NR | NR | NR |
| Tay B, 2022 | Retrospective Observational | NR | CR: 35/62, 55.7% | NR | NR | NR | NR |
| Tovar-Felice G, 2021 | RCT | NR | OR: 11/12, 92%  CR: 7/12, 58%  PR: 4/12, 33%  PD: 1/12, 8%  SD: 0 | NR | OR: 12/12, 100%  CR: 10/12, 83%  PR: 2/12, 17%  PD: 0  SD: 0 | NR | NR |
| Yun BY, 2020 | Retrospective Observational | 4 weeks | OR: 235/268, 88%  CR: 192/268, 72%  PR: 43/268, 16%  PD: 4/268, 1%  SD: 29/268, 11% | NR | NR | NR | NR |
| Zhang L, 2021 | Retrospective Observational | 1 month | cTACE:  OR: 49/67, 73%  CR: 32/67, 48%  PR: 17/67, 25%  PD: 1/67, 2%  SD: 17/67, 25%  DEB-TACE:  OR: 38/51, 75%  CR: 18/51, 35%  PR: 20/51, 39%  PD: 1/51, 2%  SD: 12/51, 24% | 3 months | cTACE:  OR: 55/67, 82%  CR: 33/67, 49%  PR: 22/67, 33%  PD: 3/67, 4.5%  SD: 9/67, 13%  DEB-TACE:  OR: 40/51, 78%  CR: 23/51, 45%  PR: 17/51, 33%  PD: 6/51, 12%  SD: 5/51, 10% | NR | NR |
| Abbreviations: TACE, transarterial chemoembolization; SLR, systematic literature review; CR, complete response; OR, objective response; PR, partial response; PD, progressive disease; SD, stable disease; NR, not reported | | | | | | | |
