# Supplementary material for: Clinical and safety outcomes in unresectable, very early and early-stage hepatocellular carcinoma following Irreversible Electroporation (IRE) and Transarterial Chemoembolization (TACE): A systematic literature review and meta-analysis
Source: PLoS One. 2025 Apr 29;20(4):e0322113. doi: 10.1371/journal.pone.0322113 (PMC12083900; doi:10.1371/journal.pone.0322113)
Supplement: S9 Table — (DOCX) [file pone.0322113.s009.docx]

# S9 Table. Very Early/Early-Stage Progression Free Survival Results, IRE SLR

| First Author, Year | Study Design | Median Time to PFS | Range Time to PFS |
| --- | --- | --- | --- |
| Kalra N, 2019 | Retrospective Observational | 10 Months | 3 – 30 Months |

Abbreviations: PFS, progression free survival
