# Supplementary material for: Clinical and safety outcomes in unresectable, very early and early-stage hepatocellular carcinoma following Irreversible Electroporation (IRE) and Transarterial Chemoembolization (TACE): A systematic literature review and meta-analysis
Source: PLoS One. 2025 Apr 29;20(4):e0322113. doi: 10.1371/journal.pone.0322113 (PMC12083900; doi:10.1371/journal.pone.0322113)
Supplement: S10 Table — (DOCX) [file pone.0322113.s010.docx]

# S10 Table. Very Early/Early-Stage Progression Free Survival Results, TACE SLR

| First Author, Year | Study Design | Median Time to PFS | Range Time to PFS | Hazard Ratio | Rate of PFS |
| --- | --- | --- | --- | --- | --- |
| Huo YR, 2019 | Retrospective Observational | 30 | NR | 30 months | NR |
| Cathomas M, 2023 | Retrospective Observational | 52 | 6.8 months | NR | 1.1-27.5 months |
| Zhang L, 2023 | Retrospective Observational | 55 | NR | 19 months (95% CI 15.101–22.899) | NR |

Abbreviations: PFS, progression free survival; CI, confidence interval; NR, not reported
