# Supplementary material for: Clinical and safety outcomes in unresectable, very early and early-stage hepatocellular carcinoma following Irreversible Electroporation (IRE) and Transarterial Chemoembolization (TACE): A systematic literature review and meta-analysis
Source: PLoS One. 2025 Apr 29;20(4):e0322113. doi: 10.1371/journal.pone.0322113 (PMC12083900; doi:10.1371/journal.pone.0322113)
Supplement: S11 Table — (DOCX) [file pone.0322113.s011.docx]

# S11 Table. Adverse Events Reported, IRE and TACE Reviews

| **Event** | **IRE** | **TACE** |
| --- | --- | --- |
| **Grade I/II or**  **mild AEs**  (n events) | - Pain (2) [37] - Increased alanine aminotransferase/aspartate aminotransferases (ALAT/ASAT) (5) [37] - Peripheral arteriovenous shunt (1) [41] - Segmental dilation of the intrahepatic biliary duct (1) [41] - Increases in systolic and diastolic blood pressure (5) [24] - Elevated serum liver transaminase and creatine phosphokinase on day 1 (5) [24] | - Fever ≥37 °C (33) [48] - Abdominal pain (21) [48] - Nausea and/or vomiting (17) [48] - Catheterization-site bleeding (6) [48] - Transient renal insufficiency (4) [48] - Groin hematoma (2) [48] - Liver decompensation (2) [48] - Post embolization syndrome (39) [49] - Post-embolization syndrome (3) [50] - Transient cholecystitis (1) [50] - Vascular access complications (VAC) and severe post-embolization syndrome (7) [55] |
| **Grade III/IV or moderate-severe AEs**  (n events) | - Hemothorax due to needle puncture of an intercostal artery requiring drainage (1) [25] - Transient hepatic decompensation undergoing spontaneous resolution (1) [25] - Vascular injury (1) [27] - Bile duct injury (1) [27] | - Liver failure (2) [49] - Variceal hemorrhage (1) [34] - Acute cholecystitis (2) [55,63] - Hepatic failure manifested by jaundice and newly formed ascites (1) [63] - Acute Azotemia (1) [63] - Liver failure (2) [31] - Localized bile duct dilation (8) [31] - Peri tumoral parenchymal ischemia (9) [31] - Liver abscess (2) [31] - Liver infraction (2) [31] - Acute cholecystitis (2) [31] - Biliary tree necrosis (2) [31] - Decompensation (4) [65] - Tumor rupture (1) [65] - Renal impairment (1) [65] - Worsening of ascites (2) [65] - New ascites (2) [65] - VAC, temporary hepatic decompensation and ischemic cholangiopathy (12) [55] |
| **Grade V or**  **Death**  (n events) | None | - Stroke and permanent hepatic decompensation (2) [55] - Death (2) [31] |
| Abbreviations: IRE, irreversible electroporation; TACE, transarterial chemoembolization; AE, adverse event; ALAT, alanine aminotransferase; ASAT, aspartate aminotransferase; VAC, vascular access complication | | |
