# Supplementary material for: Clinical and safety outcomes in unresectable, very early and early-stage hepatocellular carcinoma following Irreversible Electroporation (IRE) and Transarterial Chemoembolization (TACE): A systematic literature review and meta-analysis
Source: PLoS One. 2025 Apr 29;20(4):e0322113. doi: 10.1371/journal.pone.0322113 (PMC12083900; doi:10.1371/journal.pone.0322113)
Supplement: S16 Table — (DOCX) [file pone.0322113.s016.docx]

# S16 Table. IRE GRADE Assessment

|  | **First Author** | **Quality Rating** | **Rationale** |
| --- | --- | --- | --- |
| 1 | Cheung W, 2013 | Moderate | Study effectively controls for bias in patient selection, imprecision/inconsistency, and directly observes tumor response via established imaging criteria. The magnitude of effect is relatively large (67% had complete response at first ablation, no major complications), and while the number of patients (n=11) is limiting, the true effect is likely close to the estimated effect. |
| 2 | Fang C, 2021 | Low | Two cohorts compared different ablation techniques. Cohorts were differentiated by time sequence, not randomly. This introduced a risk of bias, as more advanced patients may have been treated earlier. Protocol also states "most" for certain techniques, masking possible confounding factors. Reliance on retrospective data also undermines the precision of results. |
| 3 | Freeman E, 2021 | Low | Frequent censorship of survival data suggests incomplete source documentation (study was retrospective). Downgrade. Results strongly suggested IRE treatment was associated with favorable tumor response. Results were complete, confidence intervals documents for local-recurrence free survival, and complications included. However, no large magnitude of effect was observed. By the end of data collection only 12 of 23 patients were still alive (despite most being early stage at treatment). Complications were relatively frequent, including kidney injury and subscapular hematoma. Reliance on retrospective data also undermines the precision of results. |
| 4 | Frühling P, 2017 | Low | The magnitude of the effect was large, however, patients were not randomized and the sample size was small (n=8), undermining reliability and precision of results. |
| 5 | Granata V, 2016 | Moderate | Strong study design. Single site, shared medical personnel, and evaluation of tumors and response via validated instruments ensured adequate control for variables and unknowns. Treatment was safe and effective, resulting in complete response after 1 treatment in 91.7% and 100% after 2 treatments. The large magnitude of effect and statistical rigor warrant the conclusion that true effect is likely similar to observed effect. |
| 6 | Kalra N, 2019 | Low | Study controlled for confound variables, shared medical personnel, and had complete data. Despite a majority of patient sample being early stage (17/21 were BCLC A), eight deaths occurred in the cohort with a median time to death of 5 months. The conclusion that IRE is an effective ablative treatment for small HCCs may or may not be accurate. Reliance on retrospective data also undermines the precision of results. |
| 7 | Lencioni R, 2012 | Very Low | Abstract details methods, clinical trial number, and outcome. However, format does not allow specificity, precision, or auxiliary statistics to effectively measure potential sources of bias or undue influence. |
| 8 | Padia SA, 2016 | Low | Outcomes are detailed in some areas, but vague in others (definitions of complete/partial response, for example). Few 95% CI, p-values, and other statistical measures on outcomes of importance. Complications are also not reported, suggest bias in outcomes included. Reliance on retrospective data also undermines the precision of results. |
| 9 | Pan F, 2021 | Low | Large magnitude of effect not observed. Patient sample size is small and procedure was conducted by multiple medical personnel, suggesting possible source of bias. Reliance on retrospective data also undermines the precision of results. |
| 10 | Sugimoto K, 2015 | Very Low | Small patient sample size and complicating events (issue applying additional pulses) suggest results may be anachronistic. Outcomes reported are largely without statistical details and standard outcomes (CR, PR, etc.). |
| 11 | Thamtorawat S, 2022 | Low | Moderate sample size, univariate and multivariate analysis, truncated outcomes, undermining the precision and reliability of effect estimates. |
| 12 | Wada T, 2023 | Moderate | Different modalities assessing therapy, large sample, detailed follow-up, large magnitude of effect |

Abbreviations: CR, complete response; PR, partial response; CI, confidence interval; HCC, hepatocellular carcinoma; IRE, irreversible electroporation; BCLC, Barcelona Clinic Liver Cancer
