# Supplementary material for: Clinical and safety outcomes in unresectable, very early and early-stage hepatocellular carcinoma following Irreversible Electroporation (IRE) and Transarterial Chemoembolization (TACE): A systematic literature review and meta-analysis
Source: PLoS One. 2025 Apr 29;20(4):e0322113. doi: 10.1371/journal.pone.0322113 (PMC12083900; doi:10.1371/journal.pone.0322113)
Supplement: S17 Table — (DOCX) [file pone.0322113.s017.docx]

# S17 Table. TACE GRADE Assessment

|  | **First Author** | **Quality Rating** | **Rationale** |
| --- | --- | --- | --- |
| 1 | Alan A, 2023 | Low | Reasonable sample size and methods detailed, however, reliance on retrospective data undermines the precision of results. |
| 2 | Bai M, 2019 | Moderate | Rigorous study methods, comprehensive outcomes reports (including CI, p-values), and multi-variate analysis support effect estimate and conclusions. |
| 3 | Bargellini I, 2012 | Low | Results suggested efficacy of TACE is mixed, and statistical measures such as CI and p-values were only reported for biomarkers. |
| 4 | Cathomas M, 2023 | Low | Adequate methods, appropriate outcomes, satisfactory sample size; however, reliance on retrospective data undermines the precision of results. |
| 5 | Chen RX, 2016 | Low | Findings are limited by imprecision in survival outcomes and indirectness. Inconsistencies between unadjusted and propensity-matched results raise questions about the robustness of the evidence. Additionally, reliance on retrospective data undermines the precision of results. |
| 6 | Chen S, 2018 | Low | Adequate methods and statistical approach, however, limited follow up compromises outcomes interpretation. |
| 7 | Chu HH, 2020 | Moderate | Magnitude of effect and rigorous study design suggest the true effect, that both DEM-TACE and cTACE are effective treatment with DEM-TACE being more effective for BCLC C and larger tumors is likely. |
| 8 | Golfieri R, 2014 | High | Large sample population and randomized across BCLC class and Child Pugh status. Outcomes were presented in full detail with a variety of results reported. Authors conclusion was that DEB-TACE did not demonstrate superiority to cTACE in efficacy, safety, and survival. It is likely that the true effect is similar to the observed effect. |
| 9 | Hashem E, 2022 | Moderate | Large sample size, detailed follow up and statistical methods controlling for risk factors and stratifying results provide a reasonable level of confidence that the true effect is close to the estimated effect. |
| 10 | Haubold J, 2020 | Very Low | P-value and confidence intervals are not included. As a result, there is a lack of precision, which casts doubt on the conclusion that DSM-TACE is an effective treatment (only 11% of patients showed complete response, further confounding conclusions). Reliance on retrospective data also undermines the precision of results. |
| 11 | Huo YR, 2019 | Very Low | Frequent data censorship in survival curves suggest true effect may be markedly different from author's conclusion that DEB-TACE w/70-150 nano-meter particles are an effective and safe treatment. Reliance on retrospective data also undermines the precision of results. |
| 12 | Hyun D, 2016 | Moderate | Strong study design and sizable sample population suggest true efficacy of TACE+RFA is likely close to observed effect. Outcomes are reported with statistical rigor including univariate and multivariate analyses. |
| 13 | Iezzi R, 2019 | Low | Some p-values were included but no confidence intervals. True effect may or may not be similar to observed effect. |
| 14 | Ikeda M, 2022 | High | Significant effect size, rigorous controls, detailed reporting enhances reliability of findings. |
| 15 | Imai Y, 2012 | Low | Medium sized study. Commonly reported outcomes are included, along with p-values, however, CI/RR/HR not included, resulting in imprecision of findings. |
| 16 | Jiang J, 2023 | Low | Despite propensity score matching, simplistic methods were used, resulting in imprecision of findings. Reliance on retrospective data also undermines the precision of results. |
| 17 | Kim JW, 2014 | Moderate | Large patient population. Outcomes reported were detailed and statistically comprehensive. Large magnitude of response was observed in tumor response rates between the two groups, with a p-value of <0.001 for CR in RFA vs TACE (RFA being superior at eliciting response). |
| 18 | Kudo M, 2020 | High | Large randomized trial comparing TACE alone to TACE + Sorafenib. A variety of outcomes were reported with rigorous statistical parameters (HR, CI, p-value). TACE + sorafenib had a statistically significant longer median PFS (p=0.006) and improved overall survival. Magnitude of effect and rigorous study design suggest true effect is close to observed effect. |
| 19 | Lee M, 2017 | Low | Variety of outcomes reported minimizes reporting bias, however, retrospective design and variable results (a large magnitude of effect was not observed, ~50% of responses were only partial), undermine the precision of effect estimates. Reliance on retrospective data also undermines the precision of results. |
| 20 | Lee YK, 2017 | Moderate | Large sample, detailed outcomes were included with appropriate statistical parameters. OS, TTP, and disease control rate were all reported by HR, CI, and p-value, with little differences observed. showed little difference. |
| 21 | Lee M, 2023 | Low | Moderate sample size, lack of clearly defined confidence intervals or measures of variability, as well as comparators, undermine the precision and reliability of effect estimates. Reliance on retrospective data also undermines the precision of results. |
| 22 | Manini MA, 2015 | Moderate | Thorough reporting of outcomes across a variety of variables. CI and p-values included for key outcomes to statistically assess impact. Likely that true effect is close to observed effect. |
| 23 | Mendez-Romero A, 2023 | Moderate | Small sample size (ended study early), appropriate comparisons |
| 24 | Ou HY, 2020 | Moderate | Comprehensive statistical reporting and detailed sub-group analysis that included p-values and other statistical measures of treatment effect. Treatment showed large magnitude of effect with complete response in 51% and effective downstaging in other patients. |
| 25 | Rahman A, 2016 | Low | Limited number of outcomes were reported and statistical detail is lacking, undermine the precision and reliability of effect estimates. Reliance on retrospective data also undermines the precision of results. |
| 26 | Razi M, 2022 | Very Low | Details are sparse and paper is brief in its presentation of methods and results. P-values included where appropriate but other statistical data is sparse. Reliance on retrospective data also undermines the precision of results. |
| 27 | Sheta E, 2016 | High | Rigorous methodology and laboratory profile of participants. Three randomized groups compared patients treated with TACE alone, TACE + RFA, and TACE + MWA. The difference in success between groups was markedly in favor of TACE + MWA. |
| 28 | Song MJ, 2012 | Moderate | Results/outcomes rigorously reported with appropriate statistical detail. Large magnitude of effect. |
| 29 | Tay B, 2022 | Low | Adequate methods, sufficient sub-analyses. Reliance on retrospective data also undermines the precision of results. |
| 30 | Tovar-Felice G, 2021 | Low | Small/medium retrospective study. Outcomes are sufficiently detailed with several sub-group analyses. Reliance on retrospective data also undermines the precision of results. |
| 31 | Yun BY, 2020 | Low | Large sample size and appropriate statistical rigor. Reliance on retrospective data also undermines the precision of results. |
| 32 | Zhang L, 2021 | Moderate | Large sample size with detailed statistical analysis and thorough results. |
| 33 | Zhang L, 2023 | Low | Detailed methods and statistical analysis. Reliance on retrospective data also undermines the precision of results. |

Abbreviations: CI, confidence interval; TACE, transarterial chemoembolization; DEM-TACE, drug-eluting microsphere transarterial chemoembolization; cTACE, conventional transarterial chemoembolization; BCLC, Barcelona Clinic Liver Cancer; DSM-TACE, degradable starch microspheres transarterial chemoembolization; DEB-TACE, drug-eluting bead transarterial chemoembolization; RR, relative risk; HR, hazard ratio; RFA, radiofrequency ablation; PFS, progression free survival; MWA, microwave ablation
