# Supplementary material for: Clinical and safety outcomes in unresectable, very early and early-stage hepatocellular carcinoma following Irreversible Electroporation (IRE) and Transarterial Chemoembolization (TACE): A systematic literature review and meta-analysis
Source: PLoS One. 2025 Apr 29;20(4):e0322113. doi: 10.1371/journal.pone.0322113 (PMC12083900; doi:10.1371/journal.pone.0322113)
Supplement: S18 Table — (DOCX) [file pone.0322113.s018.docx]

# S18 Table. All Unique Studies Identified, IRE SLR

| **Number** | **Authors** | **Title** | **Published Year** | **Published Month** | **Journal** | **Volume** | **Issue** | **Pages** | **DOI** | **Exclusion Reason (Full Text)** |
| --- | --- | --- | --- | --- | --- | --- | --- | --- | --- | --- |
| 1 | Cheung, W.; Kavnoudias, H.; Roberts, S.; Szkandera, B.; Kemp, W.; Thomson, K. R. | Irreversible electroporation for unresectable hepatocellular carcinoma: initial experience and review of safety and outcomes | 2013 | Jun | Technol Cancer Res Treat | 12 | 3 | 233-41 | 10.7785/tcrt.2012.500317 |  |
| 2 | Fang, C.; Kibriya, N.; Heaton, N. D.; Prachalias, A.; Srinivasan, P.; Menon, K.; Peddu, P. | Safety and efficacy of irreversible electroporation treatment in hepatobiliary and pancreatic tumours: a single-centre experience | 2021 | Aug | Clin Radiol | 76 | 8 | 599-606 | 10.1016/j.crad.2021.03.020 |  |
| 3 | Frühling, P.; Nilsson, A.; Duraj, F.; Haglund, U.; Norén, A. | Single-center nonrandomized clinical trial to assess the safety and efficacy of irreversible electroporation (IRE) ablation of liver tumors in humans: Short to mid-term results | 2017 | Apr | Eur J Surg Oncol | 43 | 4 | 751-757 | 10.1016/j.ejso.2016.12.004 |  |
| 4 | Freeman, E.; Cheung, W.; Kavnoudias, H.; Majeed, A.; Kemp, W.; Roberts, S. K. | Irreversible Electroporation For Hepatocellular Carcinoma: Longer-Term Outcomes At A Single Centre | 2021 | Feb | Cardiovasc Intervent Radiol | 44 | 2 | 247-253 | 10.1007/s00270-020-02666-4 |  |
| 5 | Granata, V.; de Lutio di Castelguidone, E.; Fusco, R.; Catalano, O.; Piccirillo, M.; Palaia, R.; Izzo, F.; Gallipoli, A. D.; Petrillo, A. | Irreversible electroporation of hepatocellular carcinoma: preliminary report on the diagnostic accuracy of magnetic resonance, computer tomography, and contrast-enhanced ultrasound in evaluation of the ablated area | 2016 | Feb | Radiol Med | 121 | 2 | 122-31 | 10.1007/s11547-015-0582-5 |  |
| 6 | Kalra, N.; Gupta, P.; Gorsi, U.; Bhujade, H.; Chaluvashetty, S. B.; Duseja, A.; Singh, V.; Dhiman, R. K.; Chawla, Y. K.; Khandelwal, N. | Irreversible Electroporation for Unresectable Hepatocellular Carcinoma: Initial Experience | 2019 | Apr | Cardiovasc Intervent Radiol | 42 | 4 | 584-590 | 10.1007/s00270-019-02164-2 |  |
| 7 | Lencioni, R.; Izzo, F.; Crocetti, L.; Vilgrain, V.; Abdel-Rehim, M.; Bianchi, L.; Ricke, J.; Pech, M.; Bruix, J. | Abstract No LB12: A prospective, multicenter phase II clinical trial using irreversible electroporation for the treatment of early stage HCC | 2012 |  | Journal Of Vascular And Interventional Radiology | 23 | 8 | 1114 | 10.1016/j.jvir.2012.05.018 |  |
| 8 | Padia, S. A.; Johnson, G. E.; Yeung, R. S.; Park, J. O.; Hippe, D. S.; Kogut, M. J. | Irreversible Electroporation in Patients with Hepatocellular Carcinoma: Immediate versus Delayed Findings at MR Imaging | 2016 | Jan | Radiology | 278 | 1 | 285-94 | 10.1148/radiol.2015150031 |  |
| 9 | Pan, F.; Do, T. D.; Vollherbst, D. F.; Pereira, P. L.; Richter, G. M.; Faerber, M.; Weiss, K. H.; Mehrabi, A.; Kauczor, H. U.; Sommer, C. M. | Percutaneous Irreversible Electroporation for Treatment of Small Hepatocellular Carcinoma Invisible on Unenhanced CT: A Novel Combined Strategy with Prior Transarterial Tumor Marking | 2021 | 22-Apr | Cancers (Basel) | 13 | 9 |  | 10.3390/cancers13092021 |  |
| 10 | Sugimoto, K.; Moriyasu, F.; Kobayashi, Y.; Saito, K.; Takeuchi, H.; Ogawa, S.; Ando, M.; Sano, T.; Mori, T.; Furuichi, Y.; Nakamura, I. | Irreversible electroporation for nonthermal tumor ablation in patients with hepatocellular carcinoma: initial clinical experience in Japan | 2015 | Jul | Jpn J Radiol | 33 | 7 | 424-32 | 10.1007/s11604-015-0442-1 |  |
| 11 | Thamtorawat, S.; Patanawanitkul, R.; Rojwatcharapibarn, S.; Chaiyasoot, W.; Tongdee, T.; Yodying, J.; Sorotpinya, S. | Biliary complications and efficacy after ablation of peribiliary tumors using irreversible electroporation (IRE) or radiofrequency ablation (RFA) | 2022 |  | Int J Hyperthermia | 39 | 1 | 751-757 | 10.1080/02656736.2022.2079733 |  |
| 12 | Wada, T.; Sugimoto, K.; Sakamaki, K.; Takahashi, H.; Kakegawa, T.; Tomita, Y.; Abe, M.; Yoshimasu, Y.; Takeuchi, H.; Itoi, T. | Comparisons of Radiofrequency Ablation, Microwave Ablation, and Irreversible Electroporation by Using Propensity Score Analysis for Early Stage Hepatocellular Carcinoma | 2023 | 25-Jan | Cancers (Basel) | 15 | 3 |  | 10.3390/cancers15030732 |  |
| 13 | Šilkūnas, M.; Bavirša, M.; Saulaė, R.; Batiuškaitė, D.; Saulis, G. | To breathe or not to breathe? Hypoxia after pulsed-electric field treatment reduces the effectiveness of electrochemotherapy in vitro | 2021 | Feb | Bioelectrochemistry | 137 |  | 107636 | 10.1016/j.bioelechem.2020.107636 |  |
| 14 | Adeyanju, O. O.; Al-Angari, H. M.; Sahakian, A. V. | The optimization of needle electrode number and placement for irreversible electroporation of hepatocellular carcinoma | 2012 | Jun | Radiol Oncol | 46 | 2 | 126-35 | 10.2478/v10019-012-0026-y |  |
| 15 | Agnass, P.; van Veldhuisen, E.; Vogel, J. A.; Kok, H. P.; De Keijzer, M. J.; Schooneveldt, G.; De Haan, L. R.; Klaessens, J. H.; Scheffer, H. J.; Meijerink, M. R.; van Lienden, K. P.; van Gulik, T. M.; Heger, M.; Crezee, J.; Besselink, M. G. | Thermodynamic profiling during irreversible electroporation in porcine liver and pancreas: a case study series | 2020 | 13-Apr | J Clin Transl Res | 5 | 3 | 109-132 |  |  |
| 16 | Akhan, O.; Sarıkaya, Y.; Köksal, A.; Ünal, E.; Çiftçi, T.; Akıncı, D. | Irreversible Electroporation of Recurrent Hepatocellular Carcinoma After Liver Transplantation: Report of Two Cases | 2021 | May | Cardiovasc Intervent Radiol | 44 | 5 | 807-811 | 10.1007/s00270-021-02784-7 |  |
| 17 | Alkış, M. E.; Buldurun, K.; Turan, N.; Alan, Y.; Yılmaz Ü, K.; Mantarcı, A. | Synthesis, characterization, antiproliferative of pyrimidine based ligand and its Ni(II) and Pd(II) complexes and effectiveness of electroporation | 2022 | Jun | J Biomol Struct Dyn | 40 | 9 | 4073-4083 | 10.1080/07391102.2020.1852965 |  |
| 18 | Allemailem, K. S.; Alsahli, M. A.; Almatroudi, A.; Alrumaihi, F.; Al Abdulmonem, W.; Moawad, A. A.; Alwanian, W. M.; Almansour, N. M.; Rahmani, A. H.; Khan, A. A. | Innovative Strategies of Reprogramming Immune System Cells by Targeting CRISPR/Cas9-Based Genome-Editing Tools: A New Era of Cancer Management | 2023 |  | Int J Nanomedicine | 18 |  | 5531-5559 | 10.2147/ijn.S424872 |  |
| 19 | Alnaggar, M.; Lin, M.; Mesmar, A.; Liang, S.; Qaid, A.; Xu, K.; Chen, J.; Niu, L.; Yin, Z. | Allogenic Natural Killer Cell Immunotherapy Combined with Irreversible Electroporation for Stage IV Hepatocellular Carcinoma: Survival Outcome | 2018 |  | Cell Physiol Biochem | 48 | 5 | 1882-1893 | 10.1159/000492509 |  |
| 20 | Alnaggar, M.; Qaid, A. M.; Chen, J.; Niu, L.; Xu, K. | Irreversible electroporation of malignant liver tumors: Effect on laboratory values | 2018 | Sep | Oncol Lett | 16 | 3 | 3881-3888 | 10.3892/ol.2018.9058 |  |
| 21 | Amygdalos, I.; Hitpass, L.; Schmidt, F.; Josephs, G.; Bednarsch, J.; Berres, M. L.; Lüdde, T.; Olde Damink, S. W. M.; Ulmer, T. F.; Neumann, U. P.; Bruners, P.; Lang, S. A. | Survival after combined resection and ablation is not inferior to that after resection alone, in patients with four or more colorectal liver metastases | 2023 | 29-Aug | Langenbecks Arch Surg | 408 | 1 | 343 | 10.1007/s00423-023-03082-1 |  |
| 22 | Anaganti, N.; Chattopadhyay, A.; Poirier, J. T.; Hussain, M. M. | Generation of hepatoma cell lines deficient in microsomal triglyceride transfer protein | 2022 | Sep | J Lipid Res | 63 | 9 | 100257 | 10.1016/j.jlr.2022.100257 |  |
| 23 | Arellano, R. S. | What's New in Percutaneous Ablative Strategies for Hepatocellular Carcinoma and Colorectal Hepatic Metastases? 2020 Update | 2020 | 28-Jul | Curr Oncol Rep | 22 | 10 | 105 | 10.1007/s11912-020-00967-y |  |
| 24 | Asham, E. H.; Kaseb, A.; Ghobrial, R. M. | Management of hepatocellular carcinoma | 2013 | Dec | Surg Clin North Am | 93 | 6 | 1423-50 | 10.1016/j.suc.2013.08.008 |  |
| 25 | Au, J. T.; Kingham, T. P.; Jun, K.; Haddad, D.; Gholami, S.; Mojica, K.; Monette, S.; Ezell, P.; Fong, Y. | Irreversible electroporation ablation of the liver can be detected with ultrasound B-mode and elastography | 2013 | Jun | Surgery | 153 | 6 | 787-93 | 10.1016/j.surg.2012.11.022 |  |
| 26 | Au, J. T.; Mittra, A.; Song, T. J.; Cavnar, M.; Jun, K.; Carson, J.; Gholami, S.; Haddad, D.; Gaujoux, S.; Monette, S.; Ezell, P.; Wolchok, J.; Fong, Y. | Irreversible electroporation facilitates gene transfer of a GM-CSF plasmid with a local and systemic response | 2013 | Sep | Surgery | 154 | 3 | 496-503 | 10.1016/j.surg.2013.06.005 |  |
| 27 | Bäumler, W.; Beyer, L. P.; Lürken, L.; Wiggermann, P.; Stroszczynski, C.; Dollinger, M.; Schicho, A. | Detection of Incomplete Irreversible Electroporation (IRE) and Microwave Ablation (MWA) of Hepatocellular Carcinoma (HCC) Using Iodine Quantification in Dual Energy Computed Tomography (DECT) | 2022 | 14-Apr | Diagnostics (Basel) | 12 | 4 |  | 10.3390/diagnostics12040986 | Wrong outcomes |
| 28 | Bäumler, W.; Schicho, A.; Schaible, J.; Verloh, N.; Senk, K.; Wiggermann, P.; Stroszczynski, C.; Beyer, L. P. | Changes in gadoxetic-acid-enhanced MR imaging during the first year after irreversible electroporation of malignant hepatic tumors | 2020 |  | Plos One | 15 | 11 | e0242093 | 10.1371/journal.pone.0242093 |  |
| 29 | Bäumler, W.; Sebald, M.; Einspieler, I.; Wiggermann, P.; Schicho, A.; Schaible, J.; Lürken, L.; Dollinger, M.; Stroszczynski, C.; Beyer, L. P. | Incidence and evolution of venous thrombosis during the first 3 months after irreversible electroporation of malignant hepatic tumours | 2019 | 27-Dec | Sci Rep | 9 | 1 | 19876 | 10.1038/s41598-019-56324-y | Wrong patient population |
| 30 | Bäumler, W.; Wiggermann, P.; Lürken, L.; Dollinger, M.; Stroszczynski, C.; Beyer, L. P.; Schicho, A. | Early Detection of Local Tumor Progression after Irreversible Electroporation (IRE) of a Hepatocellular Carcinoma Using Gd-EOB-DTPA-Based MR Imaging at 3T | 2021 | 30-Mar | Cancers (Basel) | 13 | 7 |  | 10.3390/cancers13071595 |  |
| 31 | Bagla, S.; Papadouris, D. | Percutaneous irreversible electroporation of surgically unresectable pancreatic cancer: a case report | 2012 | Jan | J Vasc Interv Radiol | 23 | 1 | 142-5 | 10.1016/j.jvir.2011.10.002 |  |
| 32 | Barabasch, A.; Distelmaier, M.; Heil, P.; Krämer, N. A.; Kuhl, C. K.; Bruners, P. | Magnetic Resonance Imaging Findings After Percutaneous Irreversible Electroporation of Liver Metastases: A Systematic Longitudinal Study | 2017 | Jan | Invest Radiol | 52 | 1 | 23-29 | 10.1097/rli.0000000000000301 |  |
| 33 | Beermann, M.; Lindeberg, J.; Engstrand, J.; Galmén, K.; Karlgren, S.; Stillström, D.; Nilsson, H.; Harbut, P.; Freedman, J. | 1000 consecutive ablation sessions in the era of computer assisted image guidance - Lessons learned | 2019 |  | Eur J Radiol Open | 6 |  | 8-Jan | 10.1016/j.ejro.2018.11.002 | Wrong outcomes |
| 34 | Beitel-White, N.; Bhonsle, S.; Martin, R. C. G.; Davalos, R. V. | Electrical Characterization of Human Biological Tissue for Irreversible Electroporation Treatments | 2018 | Jul | Annu Int Conf Ieee Eng Med Biol Soc | 2018 |  | 4170-4173 | 10.1109/embc.2018.8513341 |  |
| 35 | Belfiore, M. P.; De Chiara, M.; Reginelli, A.; Clemente, A.; Urraro, F.; Grassi, R.; Belfiore, G.; Cappabianca, S. | An overview of the irreversible electroporation for the treatment of liver metastases: When to use it | 2022 |  | Front Oncol | 12 |  | 943176 | 10.3389/fonc.2022.943176 |  |
| 36 | Beyer, L. P.; Pregler, B.; Michalik, K.; Niessen, C.; Dollinger, M.; Müller, M.; Schlitt, H. J.; Stroszczynski, C.; Wiggermann, P. | Evaluation of a robotic system for irreversible electroporation (IRE) of malignant liver tumors: initial results | 2017 | May | Int J Comput Assist Radiol Surg | 12 | 5 | 803-809 | 10.1007/s11548-016-1485-1 | Wrong patient population |
| 37 | Bhutiani, N.; Agle, S.; Li, Y.; Li, S.; Martin, R. C., 2nd | Irreversible electroporation enhances delivery of gemcitabine to pancreatic adenocarcinoma | 2016 | Aug | J Surg Oncol | 114 | 2 | 181-6 | 10.1002/jso.24288 |  |
| 38 | Bhutiani, N.; Davidyuk, V.; Mortensen, G. F.; Brown, A. N.; Bahr, M. H.; Martin, R. C. G., 2nd; Vitale, G. C. | Safety, Efficacy, and Technical Details of Endoscopic Retrograde Cholangiopancreatography After Irreversible Electroporation for Locally Advanced Pancreatic Cancer | 2020 | May | J Gastrointest Surg | 24 | 5 | 1077-1081 | 10.1007/s11605-019-04223-y |  |
| 39 | Bhutiani, N.; Philips, P.; Scoggins, C. R.; Mcmasters, K. M.; Potts, M. H.; Martin, R. C. | Evaluation of tolerability and efficacy of irreversible electroporation (IRE) in treatment of Child-Pugh B (7/8) hepatocellular carcinoma (HCC) | 2016 | Jul | Hpb (Oxford) | 18 | 7 | 593-9 | 10.1016/j.hpb.2016.03.609 | Wrong patient population |
| 40 | Bibok, A.; Kim, D. W.; Malafa, M.; Kis, B. | Minimally invasive image-guided therapy of primary and metastatic pancreatic cancer | 2021 | 21-Jul | World J Gastroenterol | 27 | 27 | 4322-4341 | 10.3748/wjg.v27.i27.4322 |  |
| 41 | Blaise, L.; Pereira, H.; Vilgrain, V.; Sutter, O.; Gigante, E.; Walter, A.; Ganne-Carrié, N.; Nahon, P.; Bouattour, M.; Dioguardi Burgio, M.; Grando, V.; Nkontchou, G.; Seror, O.; Nault, J. C. | Percutaneous ablation for locally advanced hepatocellular carcinoma with tumor portal invasion | 2021 | Nov | Clin Res Hepatol Gastroenterol | 45 | 6 | 101731 | 10.1016/j.clinre.2021.101731 |  |
| 42 | Boyvat, F. | Local ablation for hepatocellular carcinoma | 2014 | Mar | Exp Clin Transplant | 12 Suppl 1 |  | 55-9 | 10.6002/ect.25liver.l52 |  |
| 43 | Bréhier, G.; Besnier, L.; Delagnes, A.; Oberti, F.; Lebigot, J.; Aubé, C.; Paisant, A. | Imaging after percutaneous thermal and non-thermal ablation of hepatic tumour: normal appearances, progression and complications | 2021 | 1-Jul | Br J Radiol | 94 | 1123 | 20201327 | 10.1259/bjr.20201327 |  |
| 44 | Bruners, P. | [CT-guided local ablative interventions] | 2023 | Jul | Radiologie (Heidelb) | 63 | 7 | 490-496 | 10.1007/s00117-023-01164-1 |  |
| 45 | Bulvik, B. E.; Rozenblum, N.; Gourevich, S.; Ahmed, M.; Andriyanov, A. V.; Galun, E.; Goldberg, S. N. | Irreversible Electroporation versus Radiofrequency Ablation: A Comparison of Local and Systemic Effects in a Small-Animal Model | 2016 | Aug | Radiology | 280 | 2 | 413-24 | 10.1148/radiol.2015151166 |  |
| 46 | Cadth | CADTH Rapid Response Reports | 2016 |  | Irreversible Electroporation For Tumors Of The Pancreas Or Liver: A Review Of Clinical And Cost-Effectiveness |  |  |  |  | Wrong publication type |
| 47 | Campelo, S.; Valerio, M.; Ahmed, H. U.; Hu, Y.; Arena, S. L.; Neal, R. E., 2Nd; Emberton, M.; Arena, C. B. | An evaluation of irreversible electroporation thresholds in human prostate cancer and potential correlations to physiological measurements | 2017 | Dec | Apl Bioeng | 1 | 1 | 16101 | 10.1063/1.5005828 |  |
| 48 | Cannon, R. M.; Bolus, D. N.; White, J. A. | Irreversible Electroporation as a Bridge to Liver Transplantation | 2019 | 1-Jan | Am Surg | 85 | 1 | 103-110 |  | Wrong patient population |
| 49 | Cannon, R.; Ellis, S.; Hayes, D.; Narayanan, G.; Martin, R. C., 2nd | Safety and early efficacy of irreversible electroporation for hepatic tumors in proximity to vital structures | 2013 | Apr | J Surg Oncol | 107 | 5 | 544-9 | 10.1002/jso.23280 | Wrong patient population |
| 50 | Cano, D.; Lasarte, J. J.; Vivas, I. | [Irreversible electroporation: present and future in the treatment of hepatocellular carcinoma] | 2022 | 21-Nov | An Sist Sanit Navar | 45 | 3 |  | 10.23938/assn.1019 |  |
| 51 | Chai, W.; Tian, G.; Jiang, T. | Percutaneous Irreversible Electroporation for Portal Vein Tumor Thrombus: A Case Report | 2017 | Dec | Ultrasound Q | 33 | 4 | 296-299 | 10.1097/ruq.0000000000000305 |  |
| 52 | Chai, W.; Xie, L.; Zhao, Q.; Cheng, C.; Tian, G.; Jiang, T.; Wu, P. | Ultrasound and Contrast-enhanced Ultrasound Findings after Percutaneous Irreversible Electroporation of Hepatic Malignant Tumors | 2020 | Mar | Ultrasound Med Biol | 46 | 3 | 620-629 | 10.1016/j.ultrasmedbio.2019.12.012 |  |
| 53 | Chaiyadet, S.; Tangkawattana, S.; Smout, M. J.; Ittiprasert, W.; Mann, V. H.; Deenonpoe, R.; Arunsan, P.; Loukas, A.; Brindley, P. J.; Laha, T. | Knockout of liver fluke granulin, Ov-grn-1, impedes malignant transformation during chronic infection with Opisthorchis viverrini | 2022 | Sep | Plos Pathog | 18 | 9 | e1010839 | 10.1371/journal.ppat.1010839 |  |
| 54 | Chan, P.; Mclean, C.; Chan, S.; Goh, G. S. | The interaction between irreversible electroporation therapy (IRE) and embolization material using a validated vegetal model: an experimental study | 2019 | Jul | Diagnostic And Interventional Radiology | 25 | 4 | 304-309 | 10.5152/dir.2019.18361 |  |
| 55 | Chapiro, J.; Geschwind, J. F. | Science to Practice: Systemic Implications of Ablative Tumor Therapies-Reality Uncovered and Myths Exposed? | 2016 | Aug | Radiology | 280 | 2 | 329-31 | 10.1148/radiol.2016160505 |  |
| 56 | Charalampopoulos, G.; Iezzi, R.; Tsitskari, M.; Mazioti, A.; Papakonstantinou, O.; Kelekis, A.; Kelekis, N.; Filippiadis, D. | Role of Percutaneous Ablation in the Management of Intrahepatic Cholangiocarcinoma | 2023 | 22-Jun | Medicina (Kaunas) | 59 | 7 |  | 10.3390/medicina59071186 |  |
| 57 | Charpentier, K. P. | Irreversible electroporation for the ablation of liver tumors: are we there yet? | 2012 | Nov | Arch Surg | 147 | 11 | 1053-61 | 10.1001/2013.jamasurg.100 | Wrong study design |
| 58 | Chen, J. B.; Pan, Z. B.; Du, D. M.; Qian, W.; Ma, Y. Y.; Mu, F.; Xu, K. C. | Hydrogen gas therapy induced shrinkage of metastatic gallbladder cancer: A case report | 2019 | 6-Aug | World J Clin Cases | 7 | 15 | 2065-2074 | 10.12998/wjcc.v7.i15.2065 |  |
| 59 | Chen, S.; Zeng, X.; Su, T.; Xiao, H.; Lin, M.; Peng, Z.; Peng, S.; Kuang, M. | Combinatory local ablation and immunotherapies for hepatocellular carcinoma: Rationale, efficacy, and perspective | 2022 |  | Front Immunol | 13 |  | 1033000 | 10.3389/fimmu.2022.1033000 |  |
| 60 | Chen, T. C.; Hsieh, C. H.; Sarnow, P. | Supporting Role for GTPase Rab27a in Hepatitis C Virus RNA Replication through a Novel miR-122-Mediated Effect | 2015 | Aug | Plos Pathog | 11 | 8 | e1005116 | 10.1371/journal.ppat.1005116 |  |
| 61 | Chen, X. H.; Beebe, S. J.; Zheng, S. S. | Tumor ablation with nanosecond pulsed electric fields | 2012 | Apr | Hepatobiliary Pancreat Dis Int | 11 | 2 | 122-4 | 10.1016/s1499-3872(12)60135-0 |  |
| 62 | Chen, X.; Ren, Z.; Yin, S.; Xu, Y.; Guo, D.; Xie, H.; Zhou, L.; Wu, L.; Jiang, J.; Li, H.; Sun, J.; Zheng, S. | The local liver ablation with pulsed electric field stimulate systemic immune reaction against hepatocellular carcinoma (HCC) with time-dependent cytokine profile | 2017 | May | Cytokine | 93 |  | 44-50 | 10.1016/j.cyto.2017.05.003 |  |
| 63 | Cheng, R. G.; Bhattacharya, R.; Yeh, M. M.; Padia, S. A. | Irreversible Electroporation Can Effectively Ablate Hepatocellular Carcinoma to Complete Pathologic Necrosis | 2015 | Aug | J Vasc Interv Radiol | 26 | 8 | 1184-8 | 10.1016/j.jvir.2015.05.014 | Wrong patient population |
| 64 | Chua, T.; Faigel, D. O. | Endoscopic Ultrasound-Guided Ablation of Liver Tumors | 2019 | Apr | Gastrointest Endosc Clin N Am | 29 | 2 | 369-379 | 10.1016/j.giec.2018.11.007 |  |
| 65 | Chuong, M. D.; Herrera, R.; Ucar, A.; Aparo, S.; De Zarraga, F.; Asbun, H.; Jimenez, R.; Asbun, D.; Narayanan, G.; Joseph, S.; Kotecha, R.; Hall, M. D.; Mittauer, K. M.; Alvarez, D.; Mcculloch, J.; Romaguera, T.; Gutierrez, A.; Kaiser, A. | Causes of Death Among Patients With Initially Inoperable Pancreas Cancer After Induction Chemotherapy and Ablative 5-fraction Stereotactic Magnetic Resonance Image Guided Adaptive Radiation Therapy | 2023 | Jan-Feb | Adv Radiat Oncol | 8 | 1 | 101084 | 10.1016/j.adro.2022.101084 |  |
| 66 | Cindric, H.; Gasljevic, G.; Edhemovic, I.; Brecelj, E.; Zmuc, J.; Cemazar, M.; Seliskar, A.; Miklavcic, D.; Kos, B. | Numerical mesoscale tissue model of electrochemotherapy in liver based on histological findings | 2022 | 20-Apr | Sci Rep | 12 | 1 | 6476 | 10.1038/s41598-022-10426-2 |  |
| 67 | Cindric, H.; Mariappan, P.; Beyer, L.; Wiggermann, P.; Moche, M.; Miklavcic, D.; Kos, B. | Retrospective Study for Validation and Improvement of Numerical Treatment Planning of Irreversible Electroporation Ablation for Treatment of Liver Tumors | 2021 | Dec | Ieee Trans Biomed Eng | 68 | 12 | 3513-3524 | 10.1109/tbme.2021.3075772 |  |
| 68 | Coelen, R. J. S.; Vogel, J. A.; Vroomen, L. G. P. H.; Roos, E.; Busch, O. R. C.; van Delden, O. M.; Delft, F. V.; Heger, M.; van Hooft, J. E.; Kazemier, G.; Klümpen, H. J.; van Lienden, K. P.; Rauws, E. A. J.; Scheffer, H. J.; Verheul, H. M.; Vries, J.; Wilmink, J. W.; Zonderhuis, B. M.; Besselink, M. G.; van Gulik, T. M.; Meijerink, M. R. | Ablation with irreversible electroporation in patients with advanced perihilar cholangiocarcinoma (ALPACA): a multicentre phase I/II feasibility study protocol | 2017 | 1-Sep | Bmj Open | 7 | 9 | e015810 | 10.1136/bmjopen-2016-015810 |  |
| 69 | Cohen, E. I.; Field, D.; Lynskey, G. E.; Kim, A. Y. | Technology of irreversible electroporation and review of its clinical data on liver cancers | 2018 | Feb | Expert Rev Med Devices | 15 | 2 | 99-106 | 10.1080/17434440.2018.1425612 | Wrong patient population |
| 70 | Colquhoun, S. D. | Another club in the bag: comment on "Irreversible electroporation for the ablation of liver tumors" | 2012 | Nov | Arch Surg | 147 | 11 | 1061 | 10.1001/jamasurg.2013.493 |  |
| 71 | Cornelis, F. H.; Cindrič, H.; Kos, B.; Fujimori, M.; Petre, E. N.; Miklavčič, D.; Solomon, S. B.; Srimathveeravalli, G. | Peri-tumoral Metallic Implants Reduce the Efficacy of Irreversible Electroporation for the Ablation of Colorectal Liver Metastases | 2020 | Jan | Cardiovasc Intervent Radiol | 43 | 1 | 84-93 | 10.1007/s00270-019-02300-y |  |
| 72 | Cornelis, F. H.; Durack, J. C.; Kimm, S. Y.; Wimmer, T.; Coleman, J. A.; Solomon, S. B.; Srimathveeravalli, G. | A Comparative Study of Ablation Boundary Sharpness After Percutaneous Radiofrequency, Cryo-, Microwave, and Irreversible Electroporation Ablation in Normal Swine Liver and Kidneys | 2017 | Oct | Cardiovasc Intervent Radiol | 40 | 10 | 1600-1608 | 10.1007/s00270-017-1692-3 |  |
| 73 | Corovic, S.; Lackovic, I.; Sustaric, P.; Sustar, T.; Rodic, T.; Miklavcic, D. | Modeling of electric field distribution in tissues during electroporation | 2013 | 21-Feb | Biomed Eng Online | 12 |  | 16 | 10.1186/1475-925x-12-16 |  |
| 74 | Da Fonseca, L. G.; Araujo, R. L. C. | Combination approaches in hepatocellular carcinoma: How systemic treatment can benefit candidates to locoregional modalities | 2022 | 28-Jul | World J Gastroenterol | 28 | 28 | 3573-3585 | 10.3748/wjg.v28.i28.3573 |  |
| 75 | Dai, Z.; Wang, Z.; Lei, K.; Liao, J.; Peng, Z.; Lin, M.; Liang, P.; Yu, J.; Peng, S.; Chen, S.; Kuang, M. | Irreversible electroporation induces CD8(+) T cell immune response against post-ablation hepatocellular carcinoma growth | 2021 | 10-Apr | Cancer Lett | 503 |  | 10-Jan | 10.1016/j.canlet.2021.01.001 |  |
| 76 | Das, R.; Langou, S.; Le, T. T.; Prasad, P.; Lin, F.; Nguyen, T. D. | Electrical Stimulation for Immune Modulation in Cancer Treatments | 2021 |  | Front Bioeng Biotechnol | 9 |  | 795300 | 10.3389/fbioe.2021.795300 |  |
| 77 | de Herder, W. W.; Hofland, J.; Feingold, K. R.; Anawalt, B.; Blackman, M. R.; Boyce, A.; Chrousos, G.; Corpas, E.; De Herder, W. W.; Dhatariya, K.; Dungan, K.; Hofland, J.; Kalra, S.; Kaltsas, G.; Kapoor, N.; Koch, C.; Kopp, P.; Korbonits, M.; Kovacs, C. S.; Kuohung, W.; Laferrère, B.; Levy, M.; Mcgee, E. A.; Mclachlan, R.; New, M.; Purnell, J.; Sahay, R.; Shah, A. S.; Singer, F.; Sperling, M. A.; Stratakis, C. A.; Trence, D. L.; Wilson, D. P. | Insulinoma | 2000 |  | Endotext |  |  |  |  |  |
| 78 | de Herder, W. W.; Zandee, W. T.; Hofland, J.; Feingold, K. R.; Anawalt, B.; Boyce, A.; Chrousos, G.; De Herder, W. W.; Dhatariya, K.; Dungan, K.; Hershman, J. M.; Hofland, J.; Kalra, S.; Kaltsas, G.; Koch, C.; Kopp, P.; Korbonits, M.; Kovacs, C. S.; Kuohung, W.; Laferrère, B.; Levy, M.; Mcgee, E. A.; Mclachlan, R.; Morley, J. E.; New, M.; Purnell, J.; Sahay, R.; Singer, F.; Sperling, M. A.; Stratakis, C. A.; Trence, D. L.; Wilson, D. P. | Insulinoma | 2000 |  | Endotext |  |  |  |  |  |
| 79 | De Re, V.; Rossetto, A.; Rosignoli, A.; Muraro, E.; Racanelli, V.; Tornesello, M. L.; Zompicchiatti, A.; Uzzau, A. | Hepatocellular Carcinoma Intrinsic Cell Death Regulates Immune Response and Prognosis | 2022 |  | Front Oncol | 12 |  | 897703 | 10.3389/fonc.2022.897703 |  |
| 80 | Diaz-Nieto, R.; Fenwick, S.; Malik, H.; Poston, G. | Defining the Optimal Use of Ablation for Metastatic Colorectal Cancer to the Liver Without High-Level Evidence | 2017 | Feb | Curr Treat Options Oncol | 18 | 2 | 8 | 10.1007/s11864-017-0452-6 |  |
| 81 | Distelmaier, M.; Barabasch, A.; Heil, P.; Kraemer, N. A.; Isfort, P.; Keil, S.; Kuhl, C. K.; Bruners, P. | Midterm Safety and Efficacy of Irreversible Electroporation of Malignant Liver Tumors Located Close to Major Portal or Hepatic Veins | 2017 | Dec | Radiology | 285 | 3 | 1023-1031 | 10.1148/radiol.2017161561 | Wrong patient population |
| 82 | Djokic, M.; Cemazar, M.; Popovic, P.; Kos, B.; Dezman, R.; Bosnjak, M.; Zakelj, M. N.; Miklavcic, D.; Potrc, S.; Stabuc, B.; Tomazic, A.; Sersa, G.; Trotovsek, B. | Electrochemotherapy as treatment option for hepatocellular carcinoma, a prospective pilot study | 2018 | May | Eur J Surg Oncol | 44 | 5 | 651-657 | 10.1016/j.ejso.2018.01.090 |  |
| 83 | Djokic, M.; Dezman, R.; Cemazar, M.; Stabuc, M.; Petric, M.; Smid, L. M.; Jansa, R.; Plesnik, B.; Bosnjak, M.; Tratar, U. L.; Trotovsek, B.; Kos, B.; Miklavcic, D.; Sersa, G.; Popovic, P. | Percutaneous image guided electrochemotherapy of hepatocellular carcinoma: technological advancement | 2020 | 20-Jun | Radiol Oncol | 54 | 3 | 347-352 | 10.2478/raon-2020-0038 |  |
| 84 | Dollinger, M.; Beyer, L. P.; Haimerl, M.; Niessen, C.; Jung, E. M.; Zeman, F.; Stroszczynski, C.; Wiggermann, P. | Adverse effects of irreversible electroporation of malignant liver tumors under CT fluoroscopic guidance: a single-center experience | 2015 | Nov-Dec | Diagnostic And Interventional Radiology | 21 | 6 | 471-5 | 10.5152/dir.2015.14442 | Wrong patient population |
| 85 | Dollinger, M.; Jung, E. M.; Beyer, L.; Niessen, C.; Scheer, F.; Müller-Wille, R.; Stroszczynski, C.; Wiggermann, P. | Irreversible electroporation ablation of malignant hepatic tumors: subacute and follow-up CT appearance of ablation zones | 2014 | Oct | J Vasc Interv Radiol | 25 | 10 | 1589-94 | 10.1016/j.jvir.2014.06.026 |  |
| 86 | Dollinger, M.; Müller-Wille, R.; Zeman, F.; Haimerl, M.; Niessen, C.; Beyer, L. P.; Lang, S. A.; Teufel, A.; Stroszczynski, C.; Wiggermann, P. | Irreversible Electroporation of Malignant Hepatic Tumors--Alterations in Venous Structures at Subacute Follow-Up and Evolution at Mid-Term Follow-Up | 2015 |  | Plos One | 10 | 8 | e0135773 | 10.1371/journal.pone.0135773 |  |
| 87 | Dollinger, M.; Zeman, F.; Niessen, C.; Lang, S. A.; Beyer, L. P.; Müller, M.; Stroszczynski, C.; Wiggermann, P. | Bile Duct Injury after Irreversible Electroporation of Hepatic Malignancies: Evaluation of MR Imaging Findings and Laboratory Values | 2016 | Jan | J Vasc Interv Radiol | 27 | 1 | 96-103 | 10.1016/j.jvir.2015.10.002 |  |
| 88 | Donadon, M.; Solbiati, L.; Dawson, L.; Barry, A.; Sapisochin, G.; Greig, P. D.; Shiina, S.; Fontana, A.; Torzilli, G. | Hepatocellular Carcinoma: The Role of Interventional Oncology | 2016 | Nov | Liver Cancer | 6 | 1 | 34-43 | 10.1159/000449346 |  |
| 89 | Dong, L.; Feng, M.; Qiao, Y.; Liu, C.; Zhou, Y.; Xing, S.; Zhang, K.; Cai, Z.; Wu, H.; Wu, J.; Yu, X.; Zhang, H.; Kong, W. | Preclinical Safety and Biodistribution in Mice Following Single-Dose Intramuscular Inoculation of Tumor DNA Vaccine by Electroporation | 2022 | Jul | Hum Gene Ther | 33 | 13-14 | 757-764 | 10.1089/hum.2022.038 |  |
| 90 | Doreille, A.; N'Kontchou, G.; Halimi, A.; Bouhafs, F.; Coderc, E.; Sellier, N.; Seror, O. | Percutaneous treatment of extrahepatic recurrence of hepatocellular carcinoma | 2016 | Nov | Diagn Interv Imaging | 97 | 11 | 1117-1123 | 10.1016/j.diii.2015.11.020 | Wrong patient population |
| 91 | Dos Santos Da Luz, J. C.; Claudia Voges, A.; Guariglia D'Agostino, L. | Adjuvant electrochemotherapy of malignant ocular melanoma in a dog | 2023 |  | Vet Res Forum | 14 | 10 | 575-578 | 10.30466/vrf.2023.1983391.3721 |  |
| 92 | Eisele, R. M.; Chopra, S. S.; Glanemann, M.; Gebauer, B. | Risk of local failure after ultrasound guided irreversible electroporation of malignant liver tumors | 2014 | Dec | Interv Med Appl Sci | 6 | 4 | 147-53 | 10.1556/imas.6.2014.4.2 | Wrong patient population |
| 93 | Elboraey, M.; Devcic, Z.; Lewis, A. R.; Ritchie, C. A.; Frey, G. T.; Paz-Fumagalli, R.; Mckinney, J. M.; Toskich, B. B. | Transchondral access for irreversible electroporation of hepatocellular carcinoma | 2020 | May | Radiol Case Rep | 15 | 5 | 531-533 | 10.1016/j.radcr.2020.01.032 |  |
| 94 | Elias, D.; Viganò, L.; Orsi, F.; Scorsetti, M.; Comito, T.; Lerut, J.; Cosola, D.; Torzilli, G. | New Perspectives in the Treatment of Colorectal Metastases | 2016 | Nov | Liver Cancer | 6 | 1 | 90-98 | 10.1159/000449492 |  |
| 95 | Eller, A.; Schmid, A.; Schmidt, J.; May, M.; Brand, M.; Saake, M.; Uder, M.; Lell, M. | Local control of perivascular malignant liver lesions using percutaneous irreversible electroporation: initial experiences | 2015 | Feb | Cardiovasc Intervent Radiol | 38 | 1 | 152-9 | 10.1007/s00270-014-0898-x |  |
| 96 | Enjuto, D. T.; Herrera Merino, N.; Abadal Villandrade, J. M.; Gálvez González, E.; Llorente Lázaro, R.; Díaz Peña, P.; Álvarez Pérez , M. J.; Pérez González, M. | Irreversible Electroporation in Locally Advanced Pancreatic Adenocarcinoma: Aiming to Improve Overall Survival | 2020 | Sep | J Gastrointest Cancer | 51 | 3 | 1084-1087 | 10.1007/s12029-020-00425-9 |  |
| 97 | Eresen, A.; Sun, C.; Zhou, K.; Shangguan, J.; Wang, B.; Pan, L.; Hu, S.; Ma, Q.; Yang, J.; Zhang, Z.; Yaghmai, V. | Early Differentiation of Irreversible Electroporation Ablation Regions With Radiomics Features of Conventional MRI | 2021 | 18-Dec | Acad Radiol |  |  |  | 10.1016/j.acra.2021.11.020 |  |
| 98 | Eresen, A.; Sun, C.; Zhou, K.; Shangguan, J.; Wang, B.; Pan, L.; Hu, S.; Ma, Q.; Yang, J.; Zhang, Z.; Yaghmai, V. | Early Differentiation of Irreversible Electroporation Ablation Regions With Radiomics Features of Conventional MRI | 2022 | Sep | Acad Radiol | 29 | 9 | 1378-1386 | 10.1016/j.acra.2021.11.020 |  |
| 99 | Eresen, A.; Yang, J.; Scotti, A.; Cai, K.; Yaghmai, V.; Zhang, Z. | Combination of natural killer cell-based immunotherapy and irreversible electroporation for the treatment of hepatocellular carcinoma | 2021 | Jul | Ann Transl Med | 9 | 13 | 1089 | 10.21037/atm-21-539 |  |
| 100 | Eresen, A.; Zhou, K.; Sun, C.; Shangguan, J.; Wang, B.; Pan, L.; Hu, S.; Pang, Y.; Zhang, Z.; Tran, R. M. N.; Bhatia, A. P.; Nouizi, F.; Abi-Jaoudeh, N.; Yaghmai, V.; Zhang, Z. | Early assessment of irreversible electroporation ablation outcomes by analyzing MRI texture: preclinical study in an animal model of liver tumor | 2022 |  | Am J Transl Res | 14 | 8 | 5541-5551 |  |  |
| 101 | Fang, G.; Niu, L.; Chen, J. | Prevention of Procedural Hypertension in the Irreversible Electroporation Ablation of Liver and Pancreatic Tumors Based on Distance from the Adrenal Gland | 2020 |  | Cancer Manag Res | 12 |  | 71-78 | 10.2147/cmar.S235227 |  |
| 102 | Fang, Z.; Mao, H.; Moser, M. A. J.; Zhang, W.; Qian, Z.; Zhang, B. | Irreversible Electroporation Enhanced by Radiofrequency Ablation: An In Vitro and Computational Study in a 3D Liver Tumor Model | 2021 | Sep | Ann Biomed Eng | 49 | 9 | 2126-2138 | 10.1007/s10439-021-02734-x |  |
| 103 | Field, W.; Rostas, J. W.; Martin, R. C. G. | Quality of life assessment for patients undergoing irreversible electroporation (IRE) for treatment of locally advanced pancreatic cancer (LAPC) | 2019 | Sep | Am J Surg | 218 | 3 | 571-578 | 10.1016/j.amjsurg.2019.03.020 |  |
| 104 | Figini, M.; Wang, X.; Lyu, T.; Su, Z.; Procissi, D.; Yaghmai, V.; Larson, A. C.; Zhang, Z. | Preclinical and clinical evaluation of the liver tumor irreversible electroporation by magnetic resonance imaging | 2017 |  | Am J Transl Res | 9 | 2 | 580-590 |  |  |
| 105 | Figini, M.; Zhou, K.; Pan, L.; Sun, C.; Wang, B.; Hu, S.; Yang, J.; Shangguan, J.; Eresen, A.; Velichko, Y.; Yaghmai, V.; Zhang, Z. | Transcatheter intra-arterial perfusion (TRIP)-MRI biomarkers help detect immediate response to irreversible electroporation of rabbit VX2 liver tumor | 2020 | Jul | Magn Reson Med | 84 | 1 | 365-374 | 10.1002/mrm.28104 |  |
| 106 | Frühling, P.; Stillström, D.; Holmquist, F.; Nilsson, A.; Freedman, J. | Irreversible electroporation of hepatocellular carcinoma and colorectal cancer liver metastases: A nationwide multicenter study with short- and long-term follow-up | 2023 | Nov | Eur J Surg Oncol | 49 | 11 | 107046 | 10.1016/j.ejso.2023.107046 | Wrong patient population |
| 107 | Fredericks, C.; Arslan, B.; Mullane, M.; Firfer, B. L.; Chan, E. Y. | Needle Tract Seeding Following Irreversible Electroporation (IRE) of Metastatic Colorectal Carcinoma to the Liver | 2015 | Oct | Cardiovasc Intervent Radiol | 38 | 5 | 1349-51 | 10.1007/s00270-015-1124-1 |  |
| 108 | Freedman, J.; Nilsson, H.; Jonas, E. | New horizons in ablation therapy for hepatocellular carcinoma | 2015 | Oct | Hepat Oncol | 2 | 4 | 349-358 | 10.2217/hep.15.28 |  |
| 109 | Freeman, E.; Cheung, W.; Ferdousi, S.; Kavnoudias, H.; Majeed, A.; Kemp, W.; Roberts, S. K. | Irreversible electroporation versus radiofrequency ablation for hepatocellular carcinoma: a single centre propensity-matched comparison | 2021 | Aug | Scand J Gastroenterol | 56 | 8 | 942-947 | 10.1080/00365521.2021.1930145 | Wrong patient population |
| 110 | Freeman, E.; Cheung, W.; Kavnoudias, H.; Majeed, A.; Kemp, W.; Roberts, S. K. | Correction to: Irreversible Electroporation For Hepatocellular Carcinoma: Longer-Term Outcomes At A Single Centre | 2021 | Feb | Cardiovasc Intervent Radiol | 44 | 2 | 359 | 10.1007/s00270-020-02698-w |  |
| 111 | Froud, T.; Venkat, S. R.; Barbery, K. J.; Gunjan, A.; Narayanan, G. | Liver Function Tests Following Irreversible Electroporation of Liver Tumors: Experience in 174 Procedures | 2015 | Sep | Tech Vasc Interv Radiol | 18 | 3 | 140-6 | 10.1053/j.tvir.2015.06.004 |  |
| 112 | Fuhrmann, I.; Probst, U.; Wiggermann, P.; Beyer, L. | Navigation Systems for Treatment Planning and Execution of Percutaneous Irreversible Electroporation | 2018 | 1-Jan | Technol Cancer Res Treat | 17 |  | 1.53E+15 | 10.1177/1533033818791792 |  |
| 113 | Gómez, F. M.; Patel, P. A.; Stuart, S.; Roebuck, D. J. | Systematic review of ablation techniques for the treatment of malignant or aggressive benign lesions in children | 2014 | Oct | Pediatr Radiol | 44 | 10 | 1281-9 | 10.1007/s00247-014-3001-5 |  |
| 114 | Galati, G.; Dell'Unto, C.; Vespasiani-Gentilucci, U.; Vincentis, A.; Gallo, P.; Guidi, A.; Picardi, A. | Hepatocellular Carcinoma in Alcoholic Liver Disease: Current Management and Recent Advances | 2016 |  | Rev Recent Clin Trials | 11 | 3 | 238-252 | 10.2174/1574887111999160701091605 |  |
| 115 | Gallinato, O.; De Senneville, B. D.; Seror, O.; Poignard, C. | Numerical workflow of irreversible electroporation for deep-seated tumor | 2019 | 7-Mar | Phys Med Biol | 64 | 5 | 55016 | 10.1088/1361-6560/ab00c4 |  |
| 116 | Galmén, K.; Jakobsson, J. G.; Freedman, J.; Harbut, P. | Post-operative hypertension during early recovery following liver tumour ablation: A retrospective study | 2021 | Oct | Acta Anaesthesiol Scand | 65 | 9 | 1248-1253 | 10.1111/aas.13930 |  |
| 117 | Garnon, J.; Auloge, P.; Dalili, D.; Cazzato, R. L.; Gangi, A. | Percutaneous irreversible electroporation of porta hepatis lymph node metastasis | 2021 | Jan | Diagn Interv Imaging | 102 | 1 | 53-54 | 10.1016/j.diii.2020.11.005 |  |
| 118 | Geboers, B.; Scheffer, H. J.; Graybill, P. M.; Ruarus, A. H.; Nieuwenhuizen, S.; Puijk, R. S.; van den Tol, P. M.; Davalos, R. V.; Rubinsky, B.; De Gruijl, T. D.; Miklavčič, D.; Meijerink, M. R. | High-Voltage Electrical Pulses in Oncology: Irreversible Electroporation, Electrochemotherapy, Gene Electrotransfer, Electrofusion, and Electroimmunotherapy | 2020 | May | Radiology | 295 | 2 | 254-272 | 10.1148/radiol.2020192190 |  |
| 119 | Geboers, B.; van der Lei, S.; Kloppenborg, L. T.; Boon, R. M.; Timmer, F. E.; Puijk, R. S.; de Vries, J. J.; Scheffer, H. J.; Meijerink, M. R. | Transcatheter CT arteriography-guided irreversible electroporation of locally advanced pancreatic adenocarcinoma: A pictorial essay | 2023 | Jun | J Med Imaging Radiat Oncol | 67 | 4 | 428-434 | 10.1111/1754-9485.13535 |  |
| 120 | Ghidini, M.; Petrillo, A.; Salati, M.; Khakoo, S.; Varricchio, A.; Tomasello, G.; Grossi, F.; Petrelli, F. | Surgery or Locoregional Approaches for Hepatic Oligometastatic Pancreatic Cancer: Myth, Hope, or Reality? | 2019 | 1-Aug | Cancers (Basel) | 11 | 8 |  | 10.3390/cancers11081095 |  |
| 121 | Giorgio, A.; Amendola, F.; Calvanese, A.; Ingenito, E.; Santoro, B.; Gatti, P.; Ciracì, E.; Matteucci, P.; Giorgio, V. | Ultrasound-guided percutaneous irreversible electroporation of hepatic and abdominal tumors not eligible for surgery or thermal ablation: a western report on safety and efficacy | 2019 | Mar | J Ultrasound | 22 | 1 | 53-58 | 10.1007/s40477-019-00372-7 | Wrong patient population |
| 122 | Golberg, A.; Bruinsma, B. G.; Jaramillo, M.; Yarmush, M. L.; Uygun, B. E. | Rat liver regeneration following ablation with irreversible electroporation | 2016 |  | Peerj | 4 |  | e1571 | 10.7717/peerj.1571 |  |
| 123 | Golberg, A.; Bruinsma, B. G.; Uygun, B. E.; Yarmush, M. L. | Tissue heterogeneity in structure and conductivity contribute to cell survival during irreversible electroporation ablation by "electric field sinks" | 2015 | 16-Feb | Sci Rep | 5 |  | 8485 | 10.1038/srep08485 |  |
| 124 | Gong, Ju; Wang, Shunhong; Wang, Shuting; Li, Chaojie; Li, Wenhua; Chen, Yingjie; Xia, Ning; Wang, Zhong-Min | A Retrospective Study of the Feasibility and Safety of Irreversible Electroporation for Tumors in Special Sites of the Liver |  |  | Available At Ssrn 4204875 |  |  |  |  |  |
| 125 | Gonzalez-Beicos, A.; Venkat, S.; Songrug, T.; Poveda, J.; Garcia-Buitrago, M.; Poozhikunnath Mohan, P.; Narayanan, G. | Irreversible Electroporation of Hepatic and Pancreatic Malignancies: Radiologic-Pathologic Correlation | 2015 | Sep | Tech Vasc Interv Radiol | 18 | 3 | 176-82 | 10.1053/j.tvir.2015.06.009 | Wrong patient population |
| 126 | Granata, V.; Fusco, R.; Salati, S.; Petrillo, A.; Di Bernardo, E.; Grassi, R.; Palaia, R.; Danti, G.; La Porta, M.; Cadossi, M.; Gašljević, G.; Sersa, G.; Izzo, F. | A Systematic Review about Imaging and Histopathological Findings for Detecting and Evaluating Electroporation Based Treatments Response | 2021 | 24-May | Int J Environ Res Public Health | 18 | 11 |  | 10.3390/ijerph18115592 | Wrong publication type |
| 127 | Granata V, Fusco R, Catalano O, et al. | Percutaneous ablation therapy of hepatocellular carcinoma with irreversible electroporation: MRI findings. | 2015 |  | AJR Am J Roentgenol. | 204 | 5 | 1000-1007 | doi:10.2214/AJR.14.12509 | Wrong patient population |
| 128 | Graybill, P. M.; Davalos, R. V. | Cytoskeletal Disruption after Electroporation and Its Significance to Pulsed Electric Field Therapies | 2020 | 30-Apr | Cancers (Basel) | 12 | 5 |  | 10.3390/cancers12051132 |  |
| 129 | Green, B. L.; House, M. G. | Nonsurgical Approaches to Treat Biliary Tract and Liver Tumors | 2019 | Oct | Surg Oncol Clin N Am | 28 | 4 | 573-586 | 10.1016/j.soc.2019.06.013 |  |
| 130 | Guenther, E.; Klein, N.; Mikus, P.; Botea, F.; Pautov, M.; Lugnani, F.; Macchioro, M.; Popescu, I.; Stehling, M. K.; Rubinsky, B. | Toward a clinical real time tissue ablation technology: combining electroporation and electrolysis (E2) | 2020 |  | Peerj | 8 |  | e7985 | 10.7717/peerj.7985 |  |
| 131 | Guo, X.; Du, F.; Liu, Q.; Guo, Y.; Wang, Q.; Huang, W.; Wang, Z.; Ding, X.; Wu, Z. | Immunological effect of irreversible electroporation on hepatocellular carcinoma | 2021 | 21-Apr | Bmc Cancer | 21 | 1 | 443 | 10.1186/s12885-021-08176-x |  |
| 132 | Gupta, P.; Maralakunte, M.; Sagar, S.; Kumar, M. P.; Bhujade, H.; Chaluvashetty, S. B.; Kalra, N. | Efficacy and safety of irreversible electroporation for malignant liver tumors: a systematic review and meta-analysis | 2021 | Sep | Eur Radiol | 31 | 9 | 6511-6521 | 10.1007/s00330-021-07742-y | Wrong patient population |
| 133 | Gyftopoulos, A.; Ziogas, I. A.; Barbas, A. S.; Moris, D. | The Synergistic Role of Irreversible Electroporation and Chemotherapy for Locally Advanced Pancreatic Cancer | 2022 |  | Front Oncol | 12 |  | 843769 | 10.3389/fonc.2022.843769 |  |
| 134 | Habib, A.; Desai, K.; Hickey, R.; Thornburg, B.; Lewandowski, R.; Salem, R. | Locoregional therapy of hepatocellular carcinoma | 2015 | May | Clin Liver Dis | 19 | 2 | 401-20 | 10.1016/j.cld.2015.01.008 | Wrong patient population |
| 135 | He, F.; Li, W. N.; Li, X. X.; Yue, K. Y.; Duan, J. L.; Ruan, B.; Liu, J. J.; Song, P.; Yue, Z. S.; Tao, K. S.; Wang, L. | Exosome-mediated delivery of RBP-J decoy oligodeoxynucleotides ameliorates hepatic fibrosis in mice | 2022 |  | Theranostics | 12 | 4 | 1816-1828 | 10.7150/thno.69885 |  |
| 136 | Herwald, S. E.; Chen, J. H.; Arellano, R. S. | Irreversible Electroporation for Treatment of Hepatocellular Carcinoma Adjacent to the Gallbladder | 2016 | Jul | J Vasc Interv Radiol | 27 | 7 | 1093-4 | 10.1016/j.jvir.2016.03.008 | Wrong patient population |
| 137 | Hickey, R.; Vouche, M.; Sze, D. Y.; Hohlastos, E.; Collins, J.; Schirmang, T.; Memon, K.; Ryu, R. K.; Sato, K.; Chen, R.; Gupta, R.; Resnick, S.; Carr, J.; Chrisman, H. B.; Nemcek, A. A., Jr.; Vogelzang, R. L.; Lewandowski, R. J.; Salem, R. | Cancer concepts and principles: primer for the interventional oncologist-part II | 2013 | Aug | J Vasc Interv Radiol | 24 | 8 | 1167-88 | 10.1016/j.jvir.2013.04.023 |  |
| 138 | Hitpass, L.; Distelmaier, M.; Neumann, U. P.; Schöning, W.; Isfort, P.; Keil, S.; Kuhl, C. K.; Bruners, P.; Barabasch, A. | Recurrent Colorectal Liver Metastases in the Liver Remnant After Major Liver Surgery-IRE as a Salvage Local Treatment When Resection and Thermal Ablation are Unsuitable | 2022 | Feb | Cardiovasc Intervent Radiol | 45 | 2 | 182-189 | 10.1007/s00270-021-02981-4 |  |
| 139 | Hitpass, L.; Distelmaier, M.; Neumann, U. P.; Schöning, W.; Isfort, P.; Keil, S.; Kuhl, C. K.; Bruners, P.; Barabasch, A. | Recurrent Colorectal Liver Metastases in the Liver Remnant After Major Liver Surgery-IRE as a Salvage Local Treatment When Resection and Thermal Ablation are Unsuitable | 2022 | Feb | Cardiovasc Intervent Radiol | 45 | 2 | 182-189 | 10.1007/s00270-021-02981-4 |  |
| 140 | Hoffer, E. K.; Shelton, T. W.; Ring, N. Y. | Pulmonary Hemorrhage during Irreversible Electroporation of Hepatocellular Carcinoma | 2019 | Jun | J Vasc Interv Radiol | 30 | 6 | 970-972.e1 | 10.1016/j.jvir.2019.02.018 | Wrong patient population |
| 141 | Hong, Y.; Rice, J.; Sharma, D.; Martin, R. C. G., 2nd | The use of IRE in multi-modality treatment for oligometastatic pancreatic cancer | 2018 | Jul | Am J Surg | 216 | 1 | 106-110 | 10.1016/j.amjsurg.2018.01.037 |  |
| 142 | Hosein, P. J.; Echenique, A.; Loaiza-Bonilla, A.; Froud, T.; Barbery, K.; Rocha Lima, C. M.; Yrizarry, J. M.; Narayanan, G. | Percutaneous irreversible electroporation for the treatment of colorectal cancer liver metastases with a proposal for a new response evaluation system | 2014 | Aug | J Vasc Interv Radiol | 25 | 8 | 1233-1239.e2 | 10.1016/j.jvir.2014.04.007 |  |
| 143 | Hsiao, C. Y.; Yang, P. C.; Li, X.; Huang, K. W. | Clinical impact of irreversible electroporation ablation for unresectable hilar cholangiocarcinoma | 2020 | 2-Jul | Sci Rep | 10 | 1 | 10883 | 10.1038/s41598-020-67772-2 |  |
| 144 | Hu, S.; Sun, C.; Wang, B.; Zhou, K.; Pan, L.; Shangguan, J.; Yang, J.; Yaghmai, V.; Figini, M.; Zhang, Z. | Diffusion-Weighted MR Imaging to Evaluate Immediate Response to Irreversible Electroporation in a Rabbit VX2 Liver Tumor Model | 2019 | Nov | J Vasc Interv Radiol | 30 | 11 | 1863-1869 | 10.1016/j.jvir.2019.05.030 |  |
| 145 | Huang, K. W.; Yang, P. C.; Pua, U.; Kim, M. D.; Li, S. P.; Qiu, Y. D.; Song, T. Q.; Liang, P. C. | The efficacy of combination of induction chemotherapy and irreversible electroporation ablation for patients with locally advanced pancreatic adenocarcinoma | 2018 | Jul | J Surg Oncol | 118 | 1 | 31-36 | 10.1002/jso.25110 |  |
| 146 | Isfort, P.; Rauen, P.; Na, H. S.; Ito, N.; Von Stillfried, S.; Kuhl, C.; Bruners, P. | Does Drug-Eluting Bead TACE Enhance the Local Effect of IRE? Imaging and Histopathological Evaluation in a Porcine Model | 2019 | Jun | Cardiovasc Intervent Radiol | 42 | 6 | 880-885 | 10.1007/s00270-019-02181-1 |  |
| 147 | Jacobs, E. J. Th; Aycock, K. N.; Santos, P. P.; Tuohy, J. L.; Davalos, R. V. | Rapid estimation of electroporation-dependent tissue properties in canine lung tumors using a deep neural network | 2024 | 15-Jan | Biosens Bioelectron | 244 |  | 115777 | 10.1016/j.bios.2023.115777 |  |
| 148 | Jacobson, J. M.; Zahrieh, D.; Strand, C. A.; Cruz-Correa, M.; Pungpapong, S.; Roberts, L. R.; Mandrekar, S. J.; Rodriguez, L. M.; Boyer, J.; Marrero, I.; Kraynyak, K. A.; Morrow, M. P.; Sylvester, A. J.; Pawlicki, J. M.; Gillespie, E.; Barranco, E.; Richmond, E.; Umar, A.; Weiner, D. B.; Limburg, P. J. | Phase I Trial of a Therapeutic DNA Vaccine for Preventing Hepatocellular Carcinoma from Chronic Hepatitis C Virus (HCV) Infection | 2023 | 1-Mar | Cancer Prev Res (Phila) | 16 | 3 | 163-173 | 10.1158/1940-6207.Capr-22-0217 |  |
| 149 | Jeon, S. M.; Davaa, E.; Jiang, Y.; Jenjob, R.; Truong, N. T.; Shin, K. J.; Jeong, S.; Yang, S. G. | Assessment of Hepatic Lesions After non-Thermal Tumor Ablation by Irreversible Electroporation in a Pig Model | 2023 | Jan-Dec | Technol Cancer Res Treat | 22 |  | 1.53E+16 | 10.1177/15330338221147122 |  |
| 150 | Jiang, C.; Davalos, R. V.; Bischof, J. C. | A review of basic to clinical studies of irreversible electroporation therapy. | 2015 | Jan | Ieee Trans Biomed Eng | 62 | 1 | 20-Apr | 10.1109/TBME.2014.2367543 | Wrong patient population |
| 151 | Kah, J.; Koh, S.; Volz, T.; Ceccarello, E.; Allweiss, L.; Lütgehetmann, M.; Bertoletti, A.; Dandri, M. | Lymphocytes transiently expressing virus-specific T cell receptors reduce hepatitis B virus infection | 2017 | 1-Aug | J Clin Invest | 127 | 8 | 3177-3188 | 10.1172/jci93024 |  |
| 152 | Kalra, N.; Gupta, P.; Chawla, Y.; Khandelwal, N. | Locoregional treatment for hepatocellular carcinoma: The best is yet to come | 2015 | 28-Oct | World J Radiol | 7 | 10 | 306-18 | 10.4329/wjr.v7.i10.306 |  |
| 153 | Kang, T. W.; Lim, H. K.; Cha, D. I. | Percutaneous ablation for perivascular hepatocellular carcinoma: Refining the current status based on emerging evidence and future perspectives | 2018 | 21-Dec | World J Gastroenterol | 24 | 47 | 5331-5337 | 10.3748/wjg.v24.i47.5331 |  |
| 154 | Kang, T. W.; Rhim, H. | Recent Advances in Tumor Ablation for Hepatocellular Carcinoma | 2015 | Sep | Liver Cancer | 4 | 3 | 176-87 | 10.1159/000367740 |  |
| 155 | Kasivisvanathan, V.; Thapar, A.; Oskrochi, Y.; Picard, J.; Leen, E. L. | Irreversible electroporation for focal ablation at the porta hepatis | 2012 | Dec | Cardiovasc Intervent Radiol | 35 | 6 | 1531-4 | 10.1007/s00270-012-0363-7 |  |
| 156 | Keane, M. G.; Bramis, K.; Pereira, S. P.; Fusai, G. K. | Systematic review of novel ablative methods in locally advanced pancreatic cancer | 2014 | 7-Mar | World J Gastroenterol | 20 | 9 | 2267-78 | 10.3748/wjg.v20.i9.2267 |  |
| 157 | Khorasani, A. | Thermal damage map prediction during irreversible electroporation with U-Net | 2023 | 29-Dec | Electromagn Biol Med |  |  | 11-Jan | 10.1080/15368378.2023.2299212 |  |
| 158 | Kim, H. B.; Baik, K. Y.; Sung, C. K. | Histological Response to 5 kHz Irreversible Electroporation in a Porcine Liver Model | 2023 | Jan-Dec | Technol Cancer Res Treat | 22 |  | 1.53E+16 | 10.1177/15330338231171767 |  |
| 159 | Kingham, T. P.; Karkar, A. M.; D'Angelica, M. I.; Allen, P. J.; Dematteo, R. P.; Getrajdman, G. I.; Sofocleous, C. T.; Solomon, S. B.; Jarnagin, W. R.; Fong, Y. | Ablation of perivascular hepatic malignant tumors with irreversible electroporation | 2012 | Sep | J Am Coll Surg | 215 | 3 | 379-87 | 10.1016/j.jamcollsurg.2012.04.029 | Wrong patient population |
| 160 | Knab, L. M.; Salem, R.; Mahvi, D. M. | Minimally invasive therapies for hepatic malignancy | 2013 | Apr | Curr Probl Surg | 50 | 4 | 146-79 | 10.1067/j.cpsurg.2013.01.001 |  |
| 161 | Knavel, E. M.; Brace, C. L. | Tumor ablation: common modalities and general practices | 2013 | Dec | Tech Vasc Interv Radiol | 16 | 4 | 192-200 | 10.1053/j.tvir.2013.08.002 |  |
| 162 | Koethe, Y.; Wilson, N.; Narayanan, G. | Irreversible electroporation for colorectal cancer liver metastasis: a review | 2022 |  | Int J Hyperthermia | 39 | 1 | 682-687 | 10.1080/02656736.2021.2008025 |  |
| 163 | Kourounis, G.; Paul Tabet, P.; Moris, D.; Papalambros, A.; Felekouras, E.; Georgiades, F.; Astras, G.; Petrou, A. | Irreversible Electroporation (NanoKnife® Treatment) in the Field of Hepatobiliary Surgery: Current Status and Future Perspectives | 2017 | Jan-Feb | J Buon | 22 | 1 | 141-149 |  | Wrong patient population |
| 164 | Kovács, A.; Iezzi, R.; Cellini, F.; Lancellotta, V.; Bischoff, P.; Carchesio, F.; Tagliaferri, L.; Kovács, G.; Gambacorta, M. A. | Critical review of multidisciplinary non-surgical local interventional ablation techniques in primary or secondary liver malignancies | 2019 | Dec | J Contemp Brachytherapy | 11 | 6 | 589-600 | 10.5114/jcb.2019.90466 |  |
| 165 | Koza, A.; Bhogal, R. H.; Fotiadis, N.; Mavroeidis, V. K. | The Role of Ablative Techniques in the Management of Hepatocellular Carcinoma: Indications and Outcomes | 2023 | 31-Mar | Biomedicines | 11 | 4 |  | 10.3390/biomedicines11041062 |  |
| 166 | Kundalia, K.; Hakeem, A.; Papoulas, M.; Mcphail, M.; Reddy, S.; Peddu, P.; Kibriya, N.; Atkinson, S.; Prachalias, A.; Srinivasan, P.; Heaton, N.; Sarker, D.; Ross, P.; Zen, Y.; Menon, K. | Margin ACcentuation for resectable Pancreatic cancer using Irreversible Electroporation - Results from the MACPIE-I study | 2021 | Oct | Eur J Surg Oncol | 47 | 10 | 2571-2578 | 10.1016/j.ejso.2021.05.024 |  |
| 167 | Labarbera, N. | Uncertainty Quantification in Irreversible Electroporation Simulations | 2017 | 6-May | Bioengineering (Basel) | 4 | 2 |  | 10.3390/bioengineering4020041 |  |
| 168 | Laimer, G.; Jaschke, N.; Gottardis, M.; Schullian, P.; Putzer, D.; Sturm, W.; Bale, R. | Stereotactic Radiofrequency Ablation of an Unresectable Intrahepatic Cholangiocarcinoma (ICC): Transforming an Aggressive Disease into a Chronic Condition | 2020 | May | Cardiovasc Intervent Radiol | 43 | 5 | 791-796 | 10.1007/s00270-020-02443-3 |  |
| 169 | Lakshmi Narasimhan, P.; Tokoutsi, Z.; Baroli, D.; Baragona, M.; Veroy, K.; Maessen, R.; Ritter, A. | Global sensitivity study for irreversible electroporation: Towards treatment planning under uncertainty | 2023 | Mar | Med Phys | 50 | 3 | 1290-1304 | 10.1002/mp.16220 |  |
| 170 | Langan, R. C.; Goldman, D. A.; D'Angelica, M. I.; Dematteo, R. P.; Allen, P. J.; Balachandran, V. P.; Jarnagin, W. R.; Kingham, T. P. | Recurrence patterns following irreversible electroporation for hepatic malignancies | 2017 | May | J Surg Oncol | 115 | 6 | 704-710 | 10.1002/jso.24570 | Wrong patient population |
| 171 | Lasarte-Cia, A.; Lozano, T.; Cano, D.; Martín-Otal, C.; Navarro, F.; Gorraiz, M.; Casares, N.; Vivas, I.; Lasarte, J. J. | Intratumoral STING Agonist Injection Combined with Irreversible Electroporation Delays Tumor Growth in a Model of Hepatocarcinoma | 2021 |  | Biomed Res Int | 2021 |  | 8852233 | 10.1155/2021/8852233 |  |
| 172 | Lee, E. W.; Wong, D.; Tafti, B. A.; Prieto, V.; Totonchy, M.; Hilton, J.; Dry, S.; Cho, S.; Loh, C. T.; Kee, S. T. | Irreversible electroporation in eradication of rabbit VX2 liver tumor | 2012 | Jun | J Vasc Interv Radiol | 23 | 6 | 833-40 | 10.1016/j.jvir.2012.02.017 |  |
| 173 | Lee, S. W. L.; Adriani, G.; Ceccarello, E.; Pavesi, A.; Tan, A. T.; Bertoletti, A.; Kamm, R. D.; Wong, S. C. | Characterizing the Role of Monocytes in T Cell Cancer Immunotherapy Using a 3D Microfluidic Model | 2018 |  | Front Immunol | 9 |  | 416 | 10.3389/fimmu.2018.00416 |  |
| 174 | Lei, Y.; Liu, B.; Su, L.; Xie, X.; Kuang, M.; Chen, M.; Lan, H.; Zhao, H.; Lin, M. | Perioperative Nursing of Patients with Pancreatic Cancer Treated with a Nanoknife | 2020 | 1-Oct | J Nanosci Nanotechnol | 20 | 10 | 6584-6590 | 10.1166/jnn.2020.18603 |  |
| 175 | Lencioni, R.; Crocetti, L. | Local-regional treatment of hepatocellular carcinoma | 2012 | Jan | Radiology | 262 | 1 | 43-58 | 10.1148/radiol.11110144 |  |
| 176 | Lencioni, R.; Crocetti, L. | Image-guided ablation for hepatocellular carcinoma | 2013 |  | Recent Results Cancer Res | 190 |  | 181-94 | 10.1007/978-3-642-16037-0_12 |  |
| 177 | Lencioni, R.; Crocetti, L.; Narayanan, G. | Irreversible Electroporation in the Treatment of Hepatocellular Carcinoma | 2015 | Sep | Tech Vasc Interv Radiol | 18 | 3 | 135-9 | 10.1053/j.tvir.2015.06.003 | Wrong patient population |
| 178 | Lencioni, R.; De Baere, T.; Martin, R. C.; Nutting, C. W.; Narayanan, G. | Image-Guided Ablation of Malignant Liver Tumors: Recommendations for Clinical Validation of Novel Thermal and Non-Thermal Technologies - A Western Perspective | 2015 | Dec | Liver Cancer | 4 | 4 | 208-14 | 10.1159/000367747 |  |
| 179 | L'Huillier, R.; Dumortier, J.; Mastier, C.; Cayot, B.; Chambon, C.; Benech, N.; Stacoffe, N.; Valette, P. J.; Milot, L. | Robotic-assisted percutaneous irreversible electroporation for the treatment of hepatocellular carcinoma | 2023 | Dec | Diagn Interv Imaging | 104 | 12 | 615-617 | 10.1016/j.diii.2023.08.003 | Wrong publication type |
| 180 | Li, D.; Kang, J.; Golas, B. J.; Yeung, V. W.; Madoff, D. C. | Minimally invasive local therapies for liver cancer | 2014 | Dec | Cancer Biol Med | 11 | 4 | 217-36 | 10.7497/j.issn.2095-3941.2014.04.001 | Wrong patient population |
| 181 | Li, D.; Kang, J.; Madoff, D. C. | Locally ablative therapies for primary and metastatic liver cancer | 2014 | Aug | Expert Rev Anticancer Ther | 14 | 8 | 931-45 | 10.1586/14737140.2014.911091 |  |
| 182 | Li, J.; Zeng, J.; Chen, J.; Shi, J.; Luo, X.; Fang, G.; Chai, W.; Zhang, W.; Liu, T.; Niu, L. | Evaluation of the safety of irreversible electroporation on the stomach wall using a pig model | 2017 | Jul | Exp Ther Med | 14 | 1 | 696-702 | 10.3892/etm.2017.4559 |  |
| 183 | Li, Q.; Sun, X.; Fan, H.; Liu, Y.; Wang, S.; Huang, Y.; Li, J. | CT-Guided Irreversible Electroporation of Unresectable Pelvic Solitary Fibrous Tumor | 2023 | Feb | Cardiovasc Intervent Radiol | 46 | 2 | 289-291 | 10.1007/s00270-022-03307-8 |  |
| 184 | Li, T.; Huang, W.; Wu, Z.; Wang, Y.; Wang, Q.; Wang, Z.; Liu, Q.; Liu, J.; Wang, S.; Ding, X.; Wang, Z. | Percutaneous Ablation of Hepatic Tumors at the Hepatocaval Confluence Using Irreversible Electroporation: A Preliminary Study | 2022 | 31-May | Curr Oncol | 29 | 6 | 3950-3961 | 10.3390/curroncol29060316 |  |
| 185 | Lin, M.; Xie, X.; Xu, M.; Feng, S.; Tian, W.; Zhuang, B.; Su, L.; Ye, J.; Lin, J.; Liang, P.; Yu, J.; Kuang, M. | Non-enhanced Pattern on Contrast-Enhanced Ultrasound in the Local Efficacy Assessment of Irreversible Electroporation Ablation of Pancreatic Adenocarcinoma | 2018 | Sep | Ultrasound Med Biol | 44 | 9 | 1986-1995 | 10.1016/j.ultrasmedbio.2018.05.018 |  |
| 186 | Lin, Y. M.; Paolucci, I.; Brock, K. K.; Odisio, B. C. | Image-Guided Ablation for Colorectal Liver Metastasis: Principles, Current Evidence, and the Path Forward | 2021 | 4-Aug | Cancers (Basel) | 13 | 16 |  | 10.3390/cancers13163926 |  |
| 187 | Lindelauf, K. H. K.; Baragona, M.; Baumann, M.; Maessen, R. T. H.; Ritter, A. | Pulse Parameters and Thresholds for (ir)Reversible Electroporation on Hepatocellular Carcinoma Cells in Vitro | 2023 | Jan-Dec | Technol Cancer Res Treat | 22 |  | 1.53E+16 | 10.1177/15330338221136694 |  |
| 188 | Lindelauf, K. H. K.; Thomas, A.; Baragona, M.; Jouni, A.; Nolte, T.; Pedersoli, F.; Pfeffer, J.; Baumann, M.; Maessen, R. T. H.; Ritter, A. | Plant-based model for the visual evaluation of electroporated area after irreversible electroporation and its comparison to in-vivo animal data | 2023 | Jan-Mar | Sci Prog | 106 | 1 | 3.69E+14 | 10.1177/00368504231156294 |  |
| 189 | Liu, Boyu; Fu, Dianxun; Fan, Yong; Wang, Zhe; Lang, Xu | Irreversible electroporation versus radiofrequency ablation for malignant hepatic tumor: A prospective single-center double-arm trial | 2022 |  | Journal Of Interventional Medicine | 5 | 2 | 89-94 |  | Wrong patient population |
| 190 | Liu, Z. G.; Chen, X. H.; Yu, Z. J.; Lv, J.; Ren, Z. G. | Recent progress in pulsed electric field ablation for liver cancer | 2020 | 28-Jun | World J Gastroenterol | 26 | 24 | 3421-3431 | 10.3748/wjg.v26.i24.3421 | Wrong patient population |
| 191 | Llovet, J. M.; De Baere, T.; Kulik, L.; Haber, P. K.; Greten, T. F.; Meyer, T.; Lencioni, R. | Locoregional therapies in the era of molecular and immune treatments for hepatocellular carcinoma | 2021 | May | Nat Rev Gastroenterol Hepatol | 18 | 5 | 293-313 | 10.1038/s41575-020-00395-0 |  |
| 192 | Long, G.; Bakos, G.; Shires, P. K.; Gritter, L.; Crissman, J. W.; Harris, J. L.; Clymer, J. W. | Histological and finite element analysis of cell death due to irreversible electroporation | 2014 | Dec | Technol Cancer Res Treat | 13 | 6 | 561-9 | 10.7785/tcrtexpress.2013.600253 |  |
| 193 | Lu, D. S.; Kee, S. T.; Lee, E. W. | Irreversible electroporation: ready for prime time? | 2013 | Dec | Tech Vasc Interv Radiol | 16 | 4 | 277-86 | 10.1053/j.tvir.2013.08.010 | Wrong patient population |
| 194 | Luerken, L.; Doppler, M.; Brunner, S. M.; Schlitt, H. J.; Uller, W. | Stereotactic Percutaneous Electrochemotherapy as Primary Approach for Unresectable Large HCC at the Hepatic Hilum | 2021 | Sep | Cardiovasc Intervent Radiol | 44 | 9 | 1462-1466 | 10.1007/s00270-021-02841-1 |  |
| 195 | Luerken, L.; Haimerl, M.; Doppler, M.; Uller, W.; Beyer, L. P.; Stroszczynski, C.; Einspieler, I. | Update on Percutaneous Local Ablative Procedures for the Treatment of Hepatocellular Carcinoma | 2022 | Oct | Rofo | 194 | 10 | 1075-1086 | 10.1055/a-1768-0954 | Wrong outcomes |
| 196 | Lyu, T.; Wang, X.; Su, Z.; Shangguan, J.; Sun, C.; Figini, M.; Wang, J.; Yaghmai, V.; Larson, A. C.; Zhang, Z. | Irreversible electroporation in primary and metastatic hepatic malignancies: A review | 2017 | Apr | Medicine (Baltimore) | 96 | 17 | e6386 | 10.1097/md.0000000000006386 | Wrong patient population |
| 197 | Ma, Y. Y.; Shi, J. J.; Chen, J. B.; Xu, K. C.; Niu, L. Z. | Irreversible electroporation for liver metastasis from pancreatic cancer: A case report | 2020 | 26-Jan | World J Clin Cases | 8 | 2 | 390-397 | 10.12998/wjcc.v8.i2.390 |  |
| 198 | Ma, Y.; Chen, Z.; Liang, B.; Li, R.; Li, J.; Li, Z.; Lin, M.; Niu, L. | Irreversible Electroporation for Hepatocellular Carcinoma Abutting the Diaphragm: A Prospective Single-center Study | 2022 | 28-Apr | J Clin Transl Hepatol | 10 | 2 | 190-196 | 10.14218/jcth.2021.00019 | Wrong patient population |
| 199 | Mafeld, S.; Wong, J. J.; Kibriya, N.; Stenberg, B.; Manas, D.; Bassett, P.; Aslam, T.; Evans, J.; Littler, P. | Percutaneous Irreversible Electroporation (IRE) of Hepatic Malignancy: A Bi-institutional Analysis of Safety and Outcomes | 2019 | Apr | Cardiovasc Intervent Radiol | 42 | 4 | 577-583 | 10.1007/s00270-018-2120-z | Wrong patient population |
| 200 | Mannelli, L.; Padia, S. A.; Yeung, R. S.; Green, D. E. | Irreversible electroporation of a liver metastasis | 2013 | Jan | Liver Int | 33 | 1 | 104 | 10.1111/liv.12000 | Wrong patient population |
| 201 | Martin, C. H.; Martin, R. C. G. | Optimal Dosing and Patient Selection for Electrochemotherapy in Solid Abdominal Organ and Bone Tumors | 2023 | 18-Aug | Bioengineering (Basel) | 10 | 8 |  | 10.3390/bioengineering10080975 |  |
| 202 | Martin, E. K.; Bhutiani, N.; Egger, M. E.; Philips, P.; Scoggins, C. R.; Mcmasters, K. M.; Kelly, L. R.; Vitale, G. C.; Martin, R. C. G. | Safety and efficacy of irreversible electroporation in the treatment of obstructive jaundice in advanced hilar cholangiocarcinoma | 2018 | Nov | Hpb (Oxford) | 20 | 11 | 1092-1097 | 10.1016/j.hpb.2018.06.1806 |  |
| 203 | Martin, R. C., 2Nd; Durham, A. N.; Besselink, M. G.; Iannitti, D.; Weiss, M. J.; Wolfgang, C. L.; Huang, K. W. | Irreversible electroporation in locally advanced pancreatic cancer: A call for standardization of energy delivery | 2016 | Dec | J Surg Oncol | 114 | 7 | 865-871 | 10.1002/jso.24404 |  |
| 204 | Martin, R. C.; Philips, P.; Ellis, S.; Hayes, D.; Bagla, S. | Irreversible electroporation of unresectable soft tissue tumors with vascular invasion: effective palliation | 2014 | 26-Jul | Bmc Cancer | 14 |  | 540 | 10.1186/1471-2407-14-540 |  |
| 205 | Martin, R. C.; Schwartz, E.; Adams, J.; Farah, I.; Derhake, B. M. | Intra - operative Anesthesia Management in Patients Undergoing Surgical Irreversible Electroporation of the Pancreas, Liver, Kidney, and Retroperitoneal Tumors | 2015 | Jun | Anesth Pain Med | 5 | 3 | e22786 | 10.5812/aapm.22786 |  |
| 206 | Mathy, R. M.; Tinoush, P.; Da Florencia, R. D.; Braun, A.; Ghamarnejad, O.; Radeleff, B.; Kauczor, H. U.; Chang, D. H. | Impact of needle positioning on ablation success of irreversible electroporation: a unicentric retrospective analysis | 2020 | 14-Dec | Sci Rep | 10 | 1 | 21902 | 10.1038/s41598-020-78660-0 |  |
| 207 | Mauer, K.; O'Kelley, R.; Podda, N.; Flanagan, S.; Gadani, S. | New treatment modalities for hepatocellular cancer | 2015 | May | Curr Gastroenterol Rep | 17 | 5 | 442 | 10.1007/s11894-015-0442-4 |  |
| 208 | Mauri, G.; Monfardini, L.; Garnero, A.; Zampino, M. G.; Orsi, F.; Della Vigna, P.; Bonomo, G.; Varano, G. M.; Busso, M.; Gazzera, C.; Fonio, P.; Veltri, A.; Calandri, M. | Optimizing Loco Regional Management of Oligometastatic Colorectal Cancer: Technical Aspects and Biomarkers, Two Sides of the Same Coin | 2021 | 26-May | Cancers (Basel) | 13 | 11 |  | 10.3390/cancers13112617 |  |
| 209 | Mccaffer, C.; Wong, B. Y. W.; Murugan, C. S.; Muir, T.; Lester, S. | Electrochemotherapy for the palliative management of non-skin-origin head and neck cancer: case series and UK national survey | 2022 | Mar | J Laryngol Otol | 136 | 3 | 256-260 | 10.1017/s0022215121002942 |  |
| 210 | Mccarthy, C. J.; Kilcoyne, A.; Li, X.; Cahalane, A. M.; Liu, B.; Arellano, R. S.; Uppot, R. N.; Gee, M. S. | Radiation Dose and Risk Estimates of CT-Guided Percutaneous Liver Ablations and Factors Associated with Dose Reduction | 2018 | Dec | Cardiovasc Intervent Radiol | 41 | 12 | 1935-1942 | 10.1007/s00270-018-2066-1 |  |
| 211 | Mcdevitt, J. L.; Mouli, S. K.; Tyler, P. D.; Li, W.; Nicolai, J.; Procissi, D.; Ragin, A. B.; Wang, Y. A.; Lewandowski, R. J.; Salem, R.; Larson, A. C.; Omary, R. A. | MR imaging enables measurement of therapeutic nanoparticle uptake in rat N1-S1 liver tumors after nanoablation | 2014 | Aug | J Vasc Interv Radiol | 25 | 8 | 1288-94 | 10.1016/j.jvir.2014.03.033 |  |
| 212 | Meijerink, M. R.; Ruarus, A. H.; Vroomen, L. G. P. H.; Puijk, R. S.; Geboers, B.; Nieuwenhuizen, S.; van den Bemd, B. A. T.; Nielsen, K.; De Vries, J. J. J.; van Lienden, K. P.; Lissenberg-Witte, B. I.; van den Tol, M. P.; Scheffer, H. J. | Irreversible Electroporation to Treat Unresectable Colorectal Liver Metastases (COLDFIRE-2): A Phase II, Two-Center, Single-Arm Clinical Trial | 2021 | May | Radiology | 299 | 2 | 470-480 | 10.1148/radiol.2021203089 |  |
| 213 | Meyer, J.; Toomay, S. | Update on treatment of liver metastases: focus on ablation therapies | 2015 | Jan | Curr Oncol Rep | 17 | 1 | 420 | 10.1007/s11912-014-0420-2 |  |
| 214 | Mirzaei, S.; Gholami, M. H.; Aghdaei, H. A.; Hashemi, M.; Parivar, K.; Karamian, A.; Zarrabi, A.; Ashrafizadeh, M.; Lu, J. | Exosome-mediated miR-200a delivery into TGF-Î²-treated AGS cells abolished epithelial-mesenchymal transition with normalization of ZEB1, vimentin and Snail1 expression | 2023 | 15-Aug | Environ Res | 231 | Pt 1 | 116115 | 10.1016/j.envres.2023.116115 |  |
| 215 | Narayanan, G.; Bhatia, S.; Echenique, A.; Suthar, R.; Barbery, K.; Yrizarry, J. | Vessel patency post irreversible electroporation | 2014 | Dec | Cardiovasc Intervent Radiol | 37 | 6 | 1523-9 | 10.1007/s00270-014-0988-9 |  |
| 216 | Narayanan, G.; Froud, T.; Lo, K.; Barbery, K. J.; Perez-Rojas, E.; Yrizarry, J. | Pain analysis in patients with hepatocellular carcinoma: irreversible electroporation versus radiofrequency ablation-initial observations | 2013 | Feb | Cardiovasc Intervent Radiol | 36 | 1 | 176-82 | 10.1007/s00270-012-0426-9 | Wrong patient population |
| 217 | Narayanan, G.; Froud, T.; Suthar, R.; Barbery, K. | Irreversible electroporation of hepatic malignancy | 2013 | Mar | Semin Intervent Radiol | 30 | 1 | 67-73 | 10.1055/s-0033-1333655 | Wrong patient population |
| 218 | Nault, J. C.; Sutter, O.; Nahon, P.; Ganne-Carrié, N.; Séror, O. | Percutaneous treatment of hepatocellular carcinoma: State of the art and innovations | 2018 | Apr | J Hepatol | 68 | 4 | 783-797 | 10.1016/j.jhep.2017.10.004 |  |
| 219 | Neal, R. E., 2nd; Kavnoudias, H.; Cheung, W.; Golebiowski, B.; Mclean, C. A.; Thomson, K. R. | Hepatic epithelioid hemangioendothelioma treated with irreversible electroporation and antibiotics | 2013 | 20-Sep | J Clin Oncol | 31 | 27 | e422-6 | 10.1200/jco.2012.44.9736 |  |
| 220 | Nielsen, K.; Scheffer, H. J.; Vieveen, J. M.; van Tilborg, A. A.; Meijer, S.; van Kuijk, C.; van den Tol, M. P.; Meijerink, M. R.; Bouwman, R. A. | Anaesthetic management during open and percutaneous irreversible electroporation | 2014 | Dec | Br J Anaesth | 113 | 6 | 985-92 | 10.1093/bja/aeu256 | Wrong patient population |
| 221 | Niessen, C.; Beyer, L. P.; Haimerl, M.; Schicho, A.; Stroszczynski, C.; Wiggermann, P.; Jung, E. M. | Percutaneous irreversible electroporation of hepatocellular carcinoma: Contrast-enhanced ultrasound-findings during 1-year follow-up | 2019 |  | Clin Hemorheol Microcirc | 72 | 1 | 85-93 | 10.3233/ch-180449 | Wrong patient population |
| 222 | Niessen, C.; Beyer, L. P.; Pregler, B.; Dollinger, M.; Trabold, B.; Schlitt, H. J.; Jung, E. M.; Stroszczynski, C.; Wiggermann, P. | Percutaneous Ablation of Hepatic Tumors Using Irreversible Electroporation: A Prospective Safety and Midterm Efficacy Study in 34 Patients | 2016 | Apr | J Vasc Interv Radiol | 27 | 4 | 480-6 | 10.1016/j.jvir.2015.12.025 | Wrong patient population |
| 223 | Niessen, C.; Igl, J.; Pregler, B.; Beyer, L.; Noeva, E.; Dollinger, M.; Schreyer, A. G.; Jung, E. M.; Stroszczynski, C.; Wiggermann, P. | Factors associated with short-term local recurrence of liver cancer after percutaneous ablation using irreversible electroporation: a prospective single-center study | 2015 | May | J Vasc Interv Radiol | 26 | 5 | 694-702 | 10.1016/j.jvir.2015.02.001 | Wrong patient population |
| 224 | Niessen, C.; Jung, E. M.; Schreyer, A. G.; Wohlgemuth, W. A.; Trabold, B.; Hahn, J.; Rechenmacher, M.; Stroszczynski, C.; Wiggermann, P. | Palliative treatment of presacral recurrence of endometrial cancer using irreversible electroporation: a case report | 2013 | 13-May | J Med Case Rep | 7 |  | 128 | 10.1186/1752-1947-7-128 |  |
| 225 | Niessen, C.; Jung, E. M.; Wohlgemuth, W. A.; Trabold, B.; Haimerl, M.; Schreyer, A.; Stroszczynski, C.; Wiggermann, P. | Response | 2014 | Jan-Feb | Korean J Radiol | 15 | 1 | 182 |  |  |
| 226 | Niessen, C.; Jung, E. M.; Wohlgemuth, W. A.; Trabold, B.; Haimerl, M.; Schreyer, A.; Stroszczynski, C.; Wiggermann, P. | Irreversible electroporation of a hepatocellular carcinoma lesion adjacent to a transjugular intrahepatic portosystemic shunt stent graft | 2013 | Sep-Oct | Korean J Radiol | 14 | 5 | 797-800 | 10.3348/kjr.2013.14.5.797 |  |
| 227 | Niessen, C.; Jung, E. M.; Wohlgemuth, W. A.; Trabold, B.; Haimerl, M.; Schreyer, A.; Stroszczynski, C.; Wiggermann, P. | Response | 2014 | Jan-Feb | Korean J Radiol | 15 | 1 | 182 |  |  |
| 228 | Niessen, C.; Thumann, S.; Beyer, L.; Pregler, B.; Kramer, J.; Lang, S.; Teufel, A.; Jung, E. M.; Stroszczynski, C.; Wiggermann, P. | Percutaneous Irreversible Electroporation: Long-term survival analysis of 71 patients with inoperable malignant hepatic tumors | 2017 | 7-Mar | Sci Rep | 7 |  | 43687 | 10.1038/srep43687 | Wrong patient population |
| 229 | Nieuwenhuizen, S.; Dijkstra, M.; Puijk, R. S.; Geboers, B.; Ruarus, A. H.; Schouten, E. A.; Nielsen, K.; de Vries, J. J. J.; Bruynzeel, A. M. E.; Scheffer, H. J.; van den Tol, M. P.; Haasbeek, C. J. A.; Meijerink, M. R. | Microwave Ablation, Radiofrequency Ablation, Irreversible Electroporation, and Stereotactic Ablative Body Radiotherapy for Intermediate-Size (3-5 cm) Unresectable Colorectal Liver Metastases: A Systematic Review and Meta-analysis | 2022 | Jun | Curr Oncol Rep | 24 | 6 | 793-808 | 10.1007/s11912-022-01248-6 |  |
| 230 | Nieuwenhuizen, S.; Puijk, R. S.; van den Bemd, B.; Aldrighetti, L.; Arntz, M.; van den Boezem, P. B.; Bruynzeel, A. M. E.; Burgmans, M. C.; de Cobelli, F.; Coolsen, M. M. E.; Dejong, C. H. C.; Derks, S.; Diederik, A.; van Duijvendijk, P.; Eker, H. H.; Engelsman, A. F.; Erdmann, J. I.; Fütterer, J. J.; Geboers, B.; Groot, G.; Haasbeek, C. J. A.; Janssen, J. J.; de Jong, K. P.; Kater, G. M.; Kazemier, G.; Kruimer, J. W. H.; Leclercq, W. K. G.; van der Leij, C.; Manusama, E. R.; Meier, M. A. J.; van der Meijs, B. B.; Melenhorst, Mcam; Nielsen, K.; Nijkamp, M. W.; Potters, F. H.; Prevoo, W.; Rietema, F. J.; Ruarus, A. H.; Ruiter, S. J. S.; Schouten, E. A. C.; Serafino, G. P.; Sietses, C.; Swijnenburg, R. J.; Timmer, F. E. F.; Versteeg, K. S.; Vink, T.; de Vries, J. J. J.; de Wilt, J. H. W.; Zonderhuis, B. M.; Scheffer, H. J.; van den Tol, P. M. P.; Meijerink, M. R. | Resectability and Ablatability Criteria for the Treatment of Liver Only Colorectal Metastases: Multidisciplinary Consensus Document from the COLLISION Trial Group | 2020 | 3-Jul | Cancers (Basel) | 12 | 7 |  | 10.3390/cancers12071779 |  |
| 231 | Onafowokan, O. O.; de Liguori Carino, N. | Needle tract seeding following percutaneous irreversible electroporation for hepatocellular carcinoma | 2022 | 12-Oct | Bmj Case Rep | 15 | 10 |  | 10.1136/bcr-2022-251880 |  |
| 232 | O'Neill, C. H.; Martin, R. C. G., 2nd | Cardiac synchronization and arrhythmia during irreversible electroporation | 2020 | Sep | J Surg Oncol | 122 | 3 | 407-411 | 10.1002/jso.26041 |  |
| 233 | Owen, M.; Makary, M. S.; Beal, E. W. | Locoregional Therapy for Intrahepatic Cholangiocarcinoma | 2023 | 20-Apr | Cancers (Basel) | 15 | 8 |  | 10.3390/cancers15082384 |  |
| 234 | Pañella, C.; Castellví­, Q.; Moll, X.; Quesada, R.; Villanueva, A.; Iglesias, M.; Naranjo, D.; Sánchez-Velázquez, P.; Andaluz, A.; Grande, L.; Ivorra, A.; Burdío, F. | Focused Transhepatic Electroporation Mediated by Hypersaline Infusion through the Portal Vein in Rat Model. Preliminary Results on Differential Conductivity | 2017 | Dec | Radiol Oncol | 51 | 4 | 415-421 | 10.1515/raon-2017-0051 |  |
| 235 | Paiella, S.; Butturini, G.; Frigerio, I.; Salvia, R.; Armatura, G.; Bacchion, M.; Fontana, M.; D'Onofrio, M.; Martone, E.; Bassi, C. | Safety and feasibility of Irreversible Electroporation (IRE) in patients with locally advanced pancreatic cancer: results of a prospective study | 2015 |  | Dig Surg | 32 | 2 | 90-7 | 10.1159/000375323 |  |
| 236 | Pan, L.; Sun, C.; Zhou, K.; Figini, M.; Wang, B.; Shangguan, J.; Hu, S.; Yang, J.; Xing, W.; Wang, J.; Velichko, Y.; Yaghmai, V.; Zhang, Z. | Transcatheter Intraarterial Perfusion MRI Approaches to Differentiate Reversibly Electroporated Penumbra From Irreversibly Electroporated Zones in Rabbit Liver | 2020 | Dec | Acad Radiol | 27 | 12 | 1727-1733 | 10.1016/j.acra.2020.01.008 |  |
| 237 | Papalampros, A.; Vailas, M. G.; Deladetsima, I.; Moris, D.; Sotiropoulou, M.; Syllaios, A.; Petrou, A.; Felekouras, E. | Irreversible electroporation in a case of pancreatic leiomyosarcoma: a novel weapon versus a rare malignancy? | 2019 | 5-Jan | World J Surg Oncol | 17 | 1 | 6 | 10.1186/s12957-018-1553-9 |  |
| 238 | Papoulas, M.; Abdul-Hamid, S.; Peddu, P.; Cotoi, C.; Heaton, N.; Menon, K. | Irreversible electroporation in borderline resectable pancreatic adenocarcinoma for margin accentuation | 2018 | Jun | J Surg Case Rep | 2018 | 6 | rjy127 | 10.1093/jscr/rjy127 |  |
| 239 | Partridge, B. R.; O'Brien, T. J.; Lorenzo, M. F.; Coutermarsh-Ott, S. L.; Barry, S. L.; Stadler, K.; Muro, N.; Meyerhoeffer, M.; Allen, I. C.; Davalos, R. V.; Dervisis, N. G. | High-Frequency Irreversible Electroporation for Treatment of Primary Liver Cancer: A Proof-of-Principle Study in Canine Hepatocellular Carcinoma | 2020 | Mar | J Vasc Interv Radiol | 31 | 3 | 482-491.e4 | 10.1016/j.jvir.2019.10.015 |  |
| 240 | Pavliha, D.; Kos, B.; Marčan, M.; Županič, A.; Serša, G.; Miklavčič, D. | Planning of electroporation-based treatments using Web-based treatment-planning software | 2013 | Nov | J Membr Biol | 246 | 11 | 833-42 | 10.1007/s00232-013-9567-2 |  |
| 241 | Philips, P.; Hays, D.; Martin, R. C. | Irreversible electroporation ablation (IRE) of unresectable soft tissue tumors: learning curve evaluation in the first 150 patients treated | 2013 |  | Plos One | 8 | 11 | e76260 | 10.1371/journal.pone.0076260 | Wrong patient population |
| 242 | Phillips, M.; Rubinsky, L.; Meir, A.; Raju, N.; Rubinsky, B. | Combining Electrolysis and Electroporation for Tissue Ablation | 2015 | Aug | Technol Cancer Res Treat | 14 | 4 | 395-410 | 10.1177/1533034614560102 |  |
| 243 | Pintar, M.; Langus, J.; Edhemovič, I.; Brecelj, E.; Kranjc, M.; Sersa, G.; Šuštar, T.; Rodič, T.; Miklavčič, D.; Kotnik, T.; Kos, B. | Time-Dependent Finite Element Analysis of In Vivo Electrochemotherapy Treatment | 2018 | 1-Jan | Technol Cancer Res Treat | 17 |  | 1.53E+15 | 10.1177/1533033818790510 |  |
| 244 | Pompili, M.; Francica, G. | Irreversible electroporation for hepatic tumors | 2019 | Mar | J Ultrasound | 22 | 1 | 3-Jan | 10.1007/s40477-019-00367-4 | Wrong patient population |
| 245 | Probst, U.; Fuhrmann, I.; Beyer, L.; Wiggermann, P. | Electrochemotherapy as a New Modality in Interventional Oncology: A Review | 2018 | 1-Jan | Technol Cancer Res Treat | 17 |  | 1.53E+15 | 10.1177/1533033818785329 |  |
| 246 | Pua, U. | Re: irreversible electroporation of a hepatocellular carcinoma lesion adjacent to a transjugular intrahepatic portosystemic shunt stent graft | 2014 | Jan-Feb | Korean J Radiol | 15 | 1 | 181 | 10.3348/kjr.2014.15.1.181 |  |
| 247 | Puijk, R. S.; Ruarus, A. H.; Scheffer, H. J.; Vroomen, L. G. P. H.; van Tilborg, Aajm; de Vries, J. J. J.; Berger, F. H.; van den Tol, P. M. P.; Meijerink, M. R. | Percutaneous Liver Tumour Ablation: Image Guidance, Endpoint Assessment, and Quality Control | 2018 | Feb | Can Assoc Radiol J | 69 | 1 | 51-62 | 10.1016/j.carj.2017.11.001 |  |
| 248 | Qasrawi, R.; Silve, L.; Burdío, F.; Abdeen, Z.; Ivorra, A. | Anatomically Realistic Simulations of Liver Ablation by Irreversible Electroporation: Impact of Blood Vessels on Ablation Volumes and Undertreatment | 2017 | Dec | Technol Cancer Res Treat | 16 | 6 | 783-792 | 10.1177/1533034616687477 |  |
| 249 | Qian, K.; Zhang, F.; Allison, S. K.; Zheng, C.; Yang, X. | Image-guided locoregional non-intravascular interventional treatments for hepatocellular carcinoma: Current status | 2021 | Feb | J Interv Med | 4 | 1 | 7-Jan | 10.1016/j.jimed.2020.10.008 |  |
| 250 | Qu, Y. D.; Chen, Z.; Li, X. | Quantitative Review of Anesthesia in Liver Tumor Ablation: A Bibliometric Study from 1999 to 2022 | 2023 | 6-Jun | Med Sci Monit | 29 |  | e939607 | 10.12659/msm.939607 |  |
| 251 | Rajagopalan, N. R.; Munawar, T.; Sheehan, M. C.; Fujimori, M.; Vista, W. R.; Wimmer, T.; Gutta, N. B.; Solomon, S. B.; Srimathveeravalli, G. | Electrolysis products, reactive oxygen species and ATP loss contribute to cell death following irreversible electroporation with microsecond-long pulsed electric fields | 2024 | Feb | Bioelectrochemistry | 155 |  | 108579 | 10.1016/j.bioelechem.2023.108579 |  |
| 252 | Reataza, M.; Imagawa, D. K. | Advances in managing hepatocellular carcinoma | 2014 | Jun | Front Med | 8 | 2 | 175-89 | 10.1007/s11684-014-0332-4 |  |
| 253 | Rennert, J.; Wiesinger, I.; Schicho, A.; Wiggermann, P.; Stroszczynski, C.; Beyer, L. P.; Jung, E. M. | Color Coded Perfusion Imaging with Contrast Enhanced Ultrasound (CEUS) for Post-Interventional Success Control Following Irreversible Electroporation (IRE) of Primary and Secondary Malignant Liver Lesions | 2019 | 1-Sep | J Gastrointestin Liver Dis | 28 | 3 | 311-318 | 10.15403/jgld-254 |  |
| 254 | Ridouani, F.; Ghosn, M.; Cornelis, F.; Petre, E. N.; Hsu, M.; Moskowitz, C. S.; Kingham, P. T.; Solomon, S. B.; Srimathveeravalli, G. | Ablation Zone Involution of Liver Tumors Is Faster in Patients Treated with Irreversible Electroporation Than Microwave Ablation | 2021 | 26-Aug | Medicina (Kaunas) | 57 | 9 |  | 10.3390/medicina57090877 |  |
| 255 | Ritter, A.; Bruners, P.; Isfort, P.; Barabasch, A.; Pfeffer, J.; Schmitz, J.; Pedersoli, F.; Baumann, M. | Electroporation of the Liver: More Than 2 Concurrently Active, Curved Electrodes Allow New Concepts for Irreversible Electroporation and Electrochemotherapy | 2018 | 1-Jan | Technol Cancer Res Treat | 17 |  | 1.53E+15 | 10.1177/1533033818809994 |  |
| 256 | Ruarus, A. H.; Barabasch, A.; Catalano, O.; Leen, E.; Narayanan, G.; Nilsson, A.; Padia, S. A.; Wiggermann, P.; Scheffer, H. J.; Meijerink, M. R. | Irreversible Electroporation for Hepatic Tumors: Protocol Standardization Using the Modified Delphi Technique | 2020 | Nov | J Vasc Interv Radiol | 31 | 11 | 1765-1771.e15 | 10.1016/j.jvir.2020.02.030 |  |
| 257 | Ruarus, A. H.; Vroomen, L. G. P. H.; Puijk, R. S.; Scheffer, H. J.; Faes, T. J. C.; Meijerink, M. R. | Conductivity Rise During Irreversible Electroporation: True Permeabilization or Heat? | 2018 | Aug | Cardiovasc Intervent Radiol | 41 | 8 | 1257-1266 | 10.1007/s00270-018-1971-7 |  |
| 258 | Ruarus, A. H.; Vroomen, L. G. P. H.; Puijk, R. S.; Scheffer, H. J.; Zonderhuis, B. M.; Kazemier, G.; van den Tol, M. P.; Berger, F. H.; Meijerink, M. R. | Irreversible Electroporation in Hepatopancreaticobiliary Tumours | 2018 | Feb | Can Assoc Radiol J | 69 | 1 | 38-50 | 10.1016/j.carj.2017.10.005 |  |
| 259 | Rubinsky, L.; Guenther, E.; Mikus, P.; Stehling, M.; Rubinsky, B. | Electrolytic Effects During Tissue Ablation by Electroporation | 2016 | Oct | Technol Cancer Res Treat | 15 | 5 | Np95-np103 | 10.1177/1533034615601549 |  |
| 260 | Ryan, E. R.; Sofocleous, C. T.; Schöder, H.; Carrasquillo, J. A.; Nehmeh, S.; Larson, S. M.; Thornton, R.; Siegelbaum, R. H.; Erinjeri, J. P.; Solomon, S. B. | Split-dose technique for FDG PET/CT-guided percutaneous ablation: a method to facilitate lesion targeting and to provide immediate assessment of treatment effectiveness | 2013 | Jul | Radiology | 268 | 1 | 288-95 | 10.1148/radiol.13121462 |  |
| 261 | Ryan, M. J.; Willatt, J.; Majdalany, B. S.; Kielar, A. Z.; Chong, S.; Ruma, J. A.; Pandya, A. | Ablation techniques for primary and metastatic liver tumors | 2016 | 28-Jan | World J Hepatol | 8 | 3 | 191-9 | 10.4254/wjh.v8.i3.191 | Wrong patient population |
| 262 | Sánchez-Velázquez, P.; Castellví­, Q.; Villanueva, A.; Iglesias, M.; Quesada, R.; Pañella, C.; Cáceres, M.; Dorcaratto, D.; Andaluz, A.; Moll, X.; Burdío, J. M.; Grande, L.; Ivorra, A.; Burdío, F. | Long-term effectiveness of irreversible electroporation in a murine model of colorectal liver metastasis | 2017 | 22-Mar | Sci Rep | 7 |  | 44821 | 10.1038/srep44821 |  |
| 263 | Sánchez-Velázquez, P.; Castellví­, Q.; Villanueva, A.; Quesada, R.; Pañella, C.; Cáceres, M.; Dorcaratto, D.; Andaluz, A.; Moll, X.; Trujillo, M.; Burdío, J. M.; Berjano, E.; Grande, L.; Ivorra, A.; Burdío, F. | Irreversible electroporation of the liver: is there a safe limit to the ablation volume? | 2016 | 1-Apr | Sci Rep | 6 |  | 23781 | 10.1038/srep23781 |  |
| 264 | Sánchez-Velázquez, P.; Clavien, P. A. | The role of the irreversible electroporation in the hepato-pancreatico-biliary surgery | 2017 | Jun-Jul | Cir Esp | 95 | 6 | 307-312 | 10.1016/j.ciresp.2017.01.007 |  |
| 265 | Sahakian, A. V.; Al-Angari, H. M.; Adeyanju, O. O. | Electrode activation sequencing employing conductivity changes in irreversible electroporation tissue ablation | 2012 | Mar | Ieee Trans Biomed Eng | 59 | 3 | 604-7 | 10.1109/tbme.2011.2180722 |  |
| 266 | Saini, A.; Breen, I.; Alzubaidi, S.; Pershad, Y.; Sheth, R.; Naidu, S.; Knuttinen, M. G.; Albadawi, H.; Oklu, R. | Irreversible Electroporation in Liver Cancers and Whole Organ Engineering | 2018 | 25-Dec | J Clin Med | 8 | 1 |  | 10.3390/jcm8010022 |  |
| 267 | Salameh, Z. S.; Aycock, K. N.; Alinezhadbalalami, N.; Imran, K. M.; Mckillop, I. H.; Allen, I. C.; Davalos, R. V. | Harnessing the Electrochemical Effects of Electroporation-Based Therapies to Enhance Anti-tumor Immune Responses | 2023 | 21-Nov | Ann Biomed Eng |  |  |  | 10.1007/s10439-023-03403-x |  |
| 268 | Salati, U.; Barry, A.; Chou, F. Y.; Ma, R.; Liu, D. M. | State of the ablation nation: a review of ablative therapies for cure in the treatment of hepatocellular carcinoma | 2017 | Jul | Future Oncol | 13 | 16 | 1437-1448 | 10.2217/fon-2017-0061 |  |
| 269 | Sano, M. B.; Fesmire, C. C.; Petrella, R. A. | Electro-Thermal Therapy Algorithms and Active Internal Electrode Cooling Reduce Thermal Injury in High Frequency Pulsed Electric Field Cancer Therapies | 2021 | Jan | Ann Biomed Eng | 49 | 1 | 191-202 | 10.1007/s10439-020-02524-x |  |
| 270 | Sano, M. B.; Petrella, R. A.; Kaufman, J. D.; Fesmire, C. C.; Xing, L.; Gerber, D.; Fogle, C. A. | Electro-thermal therapy: Microsecond duration pulsed electric field tissue ablation with dynamic temperature control algorithms | 2020 | Jun | Comput Biol Med | 121 |  | 103807 | 10.1016/j.compbiomed.2020.103807 |  |
| 271 | Sarreshtehdari, A.; Burdio, F.; López-Alonso, B.; Lucía, Ó.; Burdio, J. M.; Villamonte, M.; Andaluz, A.; García-Arnas, F.; Berjano, E.; Moll, X. | Preliminary evaluation of the safety and efficacy of glucose solution infusion through the hepatic artery on irreversible electroporation focusing | 2023 | 2-May | Sci Rep | 13 | 1 | 7120 | 10.1038/s41598-023-33487-3 |  |
| 272 | Savic, L. J.; Chapiro, J.; Hamm, B.; Gebauer, B.; Collettini, F. | Irreversible Electroporation in Interventional Oncology: Where We Stand and Where We Go | 2016 | Aug | Rofo | 188 | 8 | 735-45 | 10.1055/s-0042-104203 | Wrong patient population |
| 273 | Schachtschneider, K. M. | The Promise of Improving Hepatocellular Carcinoma Treatment Responses through Translational Device Testing | 2020 | Mar | J Vasc Interv Radiol | 31 | 3 | 492-493 | 10.1016/j.jvir.2019.11.031 |  |
| 274 | Scheck, J.; Bruners, P.; Schindler, D.; Kuhl, C.; Isfort, P. | Comparison of Chronologic Change in the Size and Contrast-Enhancement of Ablation Zones on CT Images after Irreversible Electroporation and Radiofrequency Ablation | 2018 | Jul-Aug | Korean J Radiol | 19 | 4 | 560-567 | 10.3348/kjr.2018.19.4.560 |  |
| 275 | Scheffer, H. J.; Melenhorst, M. C.; Echenique, A. M.; Nielsen, K.; van Tilborg, A. A.; van den Bos, W.; Vroomen, L. G.; van den Tol, P. M.; Meijerink, M. R. | Irreversible Electroporation for Colorectal Liver Metastases | 2015 | Sep | Tech Vasc Interv Radiol | 18 | 3 | 159-69 | 10.1053/j.tvir.2015.06.007 |  |
| 276 | Scheffer, H. J.; Melenhorst, M. C.; van Tilborg, A. A.; Nielsen, K.; van Nieuwkerk, K. M.; de Vries, R. A.; van den Tol, P. M.; Meijerink, M. R. | Percutaneous Irreversible Electroporation of a Large Centrally Located Hepatocellular Adenoma in a Woman with a Pregnancy Wish | 2015 | Aug | Cardiovasc Intervent Radiol | 38 | 4 | 1031-5 | 10.1007/s00270-014-1041-8 |  |
| 277 | Scheffer, H. J.; Nielsen, K.; de Jong, M. C.; van Tilborg, A. A.; Vieveen, J. M.; Bouwman, A. R.; Meijer, S.; van Kuijk, C.; van den Tol, P. M.; Meijerink, M. R. | Irreversible electroporation for nonthermal tumor ablation in the clinical setting: a systematic review of safety and efficacy | 2014 | Jul | J Vasc Interv Radiol | 25 | 7 | 997-1011; quiz 1011 | 10.1016/j.jvir.2014.01.028 | Wrong patient population |
| 278 | Scheffer, H. J.; Nielsen, K.; van Tilborg, A. A.; Vieveen, J. M.; Bouwman, R. A.; Kazemier, G.; Niessen, H. W.; Meijer, S.; van Kuijk, C.; van den Tol, M. P.; Meijerink, M. R. | Ablation of colorectal liver metastases by irreversible electroporation: results of the COLDFIRE-I ablate-and-resect study | 2014 | Oct | Eur Radiol | 24 | 10 | 2467-75 | 10.1007/s00330-014-3259-x |  |
| 279 | Scheffer, H. J.; Vogel, J. A.; van den Bos, W.; Neal, R. E., 2nd; van Lienden, K. P.; Besselink, M. G.; van Gemert, M. J.; van der Geld, C. W.; Meijerink, M. R.; Klaessens, J. H.; Verdaasdonk, R. M. | The Influence of a Metal Stent on the Distribution of Thermal Energy during Irreversible Electroporation | 2016 |  | Plos One | 11 | 2 | e0148457 | 10.1371/journal.pone.0148457 |  |
| 280 | Scheffer, H. J.; Vroomen, L. G.; Nielsen, K.; van Tilborg, A. A.; Comans, E. F.; van Kuijk, C.; van der Meijs, B. B.; van den Bergh, J.; van den Tol, P. M.; Meijerink, M. R. | Colorectal liver metastatic disease: efficacy of irreversible electroporation--a single-arm phase II clinical trial (COLDFIRE-2 trial) | 2015 | 24-Oct | Bmc Cancer | 15 |  | 772 | 10.1186/s12885-015-1736-5 |  |
| 281 | Schembri, V.; Piron, L.; Le Roy, J.; Hermida, M.; Lonjon, J.; Escal, L.; Pierredon, M. A.; Belgour, A.; Cassinotto, C.; Guiu, B. | Percutaneous ablation of obscure hypovascular liver tumours in challenging locations using arterial CT-portography guidance | 2020 | Nov | Diagn Interv Imaging | 101 | 11 | 707-713 | 10.1016/j.diii.2020.09.005 |  |
| 282 | Schicho, A.; Niessen, C.; Haimerl, M.; Wiesinger, I.; Stroszczynski, C.; Beyer, L. P.; Wiggermann, P. | Long-term survival after percutaneous irreversible electroporation of inoperable colorectal liver metastases | 2019 |  | Cancer Manag Res | 11 |  | 317-322 | 10.2147/cmar.S182091 |  |
| 283 | Schoellhammer, H. F.; Goldner, B.; Merchant, S. J.; Kessler, J.; Fong, Y.; Gagandeep, S. | Colorectal liver metastases: making the unresectable resectable using irreversible electroporation for microscopic positive margins - a case report | 2015 | 12-Apr | Bmc Cancer | 15 |  | 271 | 10.1186/s12885-015-1279-9 |  |
| 284 | Serša, I.; Kranjc, M.; Miklavčič, D. | Current density imaging sequence for monitoring current distribution during delivery of electric pulses in irreversible electroporation | 2015 |  | Biomed Eng Online | 14 Suppl 3 | Suppl 3 | S6 | 10.1186/1475-925x-14-s3-s6 |  |
| 285 | Shangguan, A. J.; Zhou, K.; Yang, J.; Eresen, A.; Wang, B.; Sun, C.; Pan, L.; Hu, S.; Khan, A. T.; Mouli, S. K.; Yaghmai, V.; Zhang, Z. | Intraprocedural Transcatheter Intraarterial Perfusion (TRIP)-MRI for Evaluation of Irreversible Electroporation Therapy Response in a Rabbit Liver Tumor Model | 2020 |  | Clin Exp Gastroenterol | 13 |  | 543-553 | 10.2147/ceg.S269163 |  |
| 286 | Shankara Narayanan, J. S.; Hayashi, T.; Erdem, S.; Mcardle, S.; Tiriac, H.; Ray, P.; Pu, M.; Mikulski, Z.; Miller, A.; Messer, K.; Carson, D.; Schoenberger, S.; White, R. R. | Treatment of pancreatic cancer with irreversible electroporation and intratumoral CD40 antibody stimulates systemic immune responses that inhibit liver metastasis in an orthotopic model | 2023 | Jan | J Immunother Cancer | 11 | 1 |  | 10.1136/jitc-2022-006133 |  |
| 287 | Shi, X.; O'Neill, C.; Wang, X.; Chen, Y.; Yu, Y.; Tan, M.; Lv, G.; Li, Y.; Martin, R. C. | Irreversible electroporation enhances immunotherapeutic effect in the off-target tumor in a murine model of orthotopic HCC | 2021 |  | Am J Cancer Res | 11 | 6 | 3304-3319 |  |  |
| 288 | Shiina, S.; Sato, K.; Tateishi, R.; Shimizu, M.; Ohama, H.; Hatanaka, T.; Takawa, M.; Nagamatsu, H.; Imai, Y. | Percutaneous Ablation for Hepatocellular Carcinoma: Comparison of Various Ablation Techniques and Surgery | 2018 |  | Can J Gastroenterol Hepatol | 2018 |  | 4756147 | 10.1155/2018/4756147 |  |
| 289 | Siddiqui, I. A.; Kirks, R. C.; Latouche, E. L.; Dewitt, M. R.; Swet, J. H.; Baker, E. H.; Vrochides, D.; Iannitti, D. A.; Davalos, R. V.; Mckillop, I. H. | High-Frequency Irreversible Electroporation: Safety and Efficacy of Next-Generation Irreversible Electroporation Adjacent to Critical Hepatic Structures | 2017 | Jun | Surg Innov | 24 | 3 | 276-283 | 10.1177/1553350617692202 | Wrong patient population |
| 290 | Silk, M. T.; Wimmer, T.; Lee, K. S.; Srimathveeravalli, G.; Brown, K. T.; Kingham, P. T.; Fong, Y.; Durack, J. C.; Sofocleous, C. T.; Solomon, S. B. | Percutaneous ablation of peribiliary tumors with irreversible electroporation | 2014 | Jan | J Vasc Interv Radiol | 25 | 1 | 112-8 | 10.1016/j.jvir.2013.10.012 |  |
| 291 | Silk, M.; Tahour, D.; Srimathveeravalli, G.; Solomon, S. B.; Thornton, R. H. | The state of irreversible electroporation in interventional oncology | 2014 | Jun | Semin Intervent Radiol | 31 | 2 | 111-7 | 10.1055/s-0034-1373785 |  |
| 292 | Silva-Pilipich, N.; Lasarte-Cía, A.; Lozano, T.; Martín-Otal, C.; Lasarte, J. J.; Smerdou, C. | Intratumoral electroporation of a self-amplifying RNA expressing IL-12 induces antitumor effects in mouse models of cancer | 2022 | 13-Sep | Mol Ther Nucleic Acids | 29 |  | 387-399 | 10.1016/j.omtn.2022.07.020 |  |
| 293 | Simmerman, E.; Chung, J.; Lawson, A.; Kruse, E. | Application of Irreversible Electroporation Ablation as Adjunctive Treatment for Margin Enhancement: Safety and Efficacy | 2020 | Feb | J Surg Res | 246 |  | 260-268 | 10.1016/j.jss.2019.06.010 |  |
| 294 | Sofocleous, C. T.; Sideras, P.; Petre, E. N. | "How we do it" - a practical approach to hepatic metastases ablation techniques | 2013 | Dec | Tech Vasc Interv Radiol | 16 | 4 | 219-29 | 10.1053/j.tvir.2013.08.005 |  |
| 295 | Song, J.; Zhang, T.; Wang, J.; Liu, Y. | Ablation treatment of hepatocellular carcinoma: a bibliometric analysis | 2023 |  | Front Oncol | 13 |  | 1166775 | 10.3389/fonc.2023.1166775 |  |
| 296 | Spallek, H.; Bischoff, P.; Zhou, W.; de Terlizzi, F.; Jakob, F.; Kovács, A. | Percutaneous electrochemotherapy in primary and secondary liver malignancies - local tumor control and impact on overall survival | 2022 | 11-Feb | Radiol Oncol | 56 | 1 | 102-110 | 10.2478/raon-2022-0003 |  |
| 297 | Spiers, H. V. M.; Lancellotti, F.; de Liguori Carino, N.; Pandanaboyana, S.; Frampton, A. E.; Jegatheeswaran, S.; Nadarajah, V.; Siriwardena, A. K. | Irreversible Electroporation for Liver Metastases from Colorectal Cancer: A Systematic Review | 2023 | 24-Apr | Cancers (Basel) | 15 | 9 |  | 10.3390/cancers15092428 |  |
| 298 | Spiliotis, A. E.; Holländer, S.; Rudzitis-Auth, J.; Wagenpfeil, G.; Eisele, R.; Nika, S.; Mallis Kyriakides, O.; Laschke, M. W.; Menger, M. D.; Glanemann, M.; Gäbelein, G. | Evaluation of Electrochemotherapy with Bleomycin in the Treatment of Colorectal Hepatic Metastases in a Rat Model | 2023 | 4-Mar | Cancers (Basel) | 15 | 5 |  | 10.3390/cancers15051598 |  |
| 299 | Steinbrecher, K.; Arslan, B.; Nassin, M. L.; Kent, P. | Irreversible electroporation in the curative treatment of Ewing's sarcoma | 2016 | 21-Sep | Bmj Case Rep | 2016 |  |  | 10.1136/bcr-2016-216585 |  |
| 300 | Stewart, C. L.; Warner, S.; Ito, K.; Raoof, M.; Wu, G. X.; Kessler, J.; Kim, J. Y.; Fong, Y. | Cytoreduction for colorectal metastases: liver, lung, peritoneum, lymph nodes, bone, brain. When does it palliate, prolong survival, and potentially cure? | 2018 | Sep | Curr Probl Surg | 55 | 9 | 330-379 | 10.1067/j.cpsurg.2018.08.004 |  |
| 301 | Stillström, D.; Beermann, M.; Engstrand, J.; Freedman, J.; Nilsson, H. | Initial experience with irreversible electroporation of liver tumours | 2019 |  | Eur J Radiol Open | 6 |  | 62-67 | 10.1016/j.ejro.2019.01.004 | Wrong patient population |
| 302 | Sugimoto, K.; Abe, M.; Yoshimasu, Y.; Takeuchi, H.; Kasai, Y.; Itoi, T. | Irreversible electroporation of hepatocellular carcinoma: the role of ultrasonography | 2020 | Jul | Ultrasonography | 39 | 3 | 229-237 | 10.14366/usg.20023 | Wrong outcomes |
| 303 | Sugimoto, K.; Kakimi, K.; Takeuchi, H.; Fujieda, N.; Saito, K.; Sato, E.; Sakamaki, K.; Moriyasu, F.; Itoi, T. | Irreversible Electroporation versus Radiofrequency Ablation: Comparison of Systemic Immune Responses in Patients with Hepatocellular Carcinoma | 2019 | Jun | J Vasc Interv Radiol | 30 | 6 | 845-853.e6 | 10.1016/j.jvir.2019.03.002 |  |
| 304 | Sugimoto, K.; Moriyasu, F.; Saito, K.; Kobayashi, Y.; Itoi, T. | Multimodality imaging to assess immediate response following irreversible electroporation in patients with malignant hepatic tumors | 2017 | Jul | J Med Ultrason (2001) | 44 | 3 | 247-254 | 10.1007/s10396-016-0767-0 |  |
| 305 | Sugimoto, K.; Moriyasu, F.; Yoshiyuki, K.; Imai, Y. | Assessment of irreversible electroporation ablation zone using Kupffer-phase contrast-enhanced ultrasound images with Sonazoid | 2014 | Oct | J Med Ultrason (2001) | 41 | 4 | 531-2 | 10.1007/s10396-014-0547-7 |  |
| 306 | Sung, C. K.; Kim, H. B.; Jung, J. H.; Baik, K. Y.; Moon, K. W.; Kim, H. S.; Yi, J. H.; Chung, J. H. | Histological and Mathematical Analysis of the Irreversibly Electroporated Liver Tissue | 2017 | Aug | Technol Cancer Res Treat | 16 | 4 | 488-496 | 10.1177/1533034616640642 |  |
| 307 | Sutter, O.; Calvo, J.; N'Kontchou, G.; Nault, J. C.; Ourabia, R.; Nahon, P.; Ganne-Carrié, N.; Bourcier, V.; Zentar, N.; Bouhafs, F.; Sellier, N.; Diallo, A.; Seror, O. | Safety and Efficacy of Irreversible Electroporation for the Treatment of Hepatocellular Carcinoma Not Amenable to Thermal Ablation Techniques: A Retrospective Single-Center Case Series | 2017 | Sep | Radiology | 284 | 3 | 877-886 | 10.1148/radiol.2017161413 | Wrong patient population |
| 308 | Sutter, O.; Fihri, A.; Ourabia-Belkacem, R.; Sellier, N.; Diallo, A.; Seror, O. | Real-Time 3D Virtual Target Fluoroscopic Display for Challenging Hepatocellular Carcinoma Ablations Using Cone Beam CT | 2018 | 1-Jan | Technol Cancer Res Treat | 17 |  | 1.53E+15 | 10.1177/1533033818789634 | Wrong patient population |
| 309 | Suzuki, R.; Suzuki, T. | Reverse Genetics of Hepatitis C Virus Using an RNA Polymerase I-Mediated Transcription | 2024 |  | Methods Mol Biol | 2733 |  | 175-183 | 10.1007/978-1-0716-3533-9_11 |  |
| 310 | Sweeney, J.; Parikh, N.; El-Haddad, G.; Kis, B. | Ablation of Intrahepatic Cholangiocarcinoma | 2019 | Oct | Semin Intervent Radiol | 36 | 4 | 298-302 | 10.1055/s-0039-1696649 |  |
| 311 | Tam, A. L.; Melancon, M. P.; Abdelsalam, M.; Figueira, T. A.; Dixon, K.; Mcwatters, A.; Zhou, M.; Huang, Q.; Mawlawi, O.; Dunner, K., Jr.; Li, C.; Gupta, S. | Imaging Intratumoral Nanoparticle Uptake After Combining Nanoembolization with Various Ablative Therapies in Hepatic VX2 Rabbit Tumors | 2016 | Feb | J Biomed Nanotechnol | 12 | 2 | 296-307 | 10.1166/jbn.2016.2174 |  |
| 312 | Tameez Ud Din, A.; Tameez-Ud-Din, A.; Chaudhary, F. M. D.; Chaudhary, N. A.; Siddiqui, K. H. | Irreversible Electroporation For Liver Tumors: A Review Of Literature | 2019 | 25-Jun | Cureus | 11 | 6 | e4994 | 10.7759/cureus.4994 | Wrong patient population |
| 313 | Tamura, M.; Pedersoli, F.; Schulze-Hagen, M.; Zimmerman, M.; Isfort, P.; Kuhl, C. K.; Schmitz-Rode, T.; Bruners, P. | Predictors of Occlusion of Hepatic Blood Vessels after Irreversible Electroporation of Liver Tumors | 2020 | Dec | J Vasc Interv Radiol | 31 | 12 | 2033-2042.e1 | 10.1016/j.jvir.2020.08.005 |  |
| 314 | Tan, A. T.; Yang, N.; Lee Krishnamoorthy, T.; Oei, V.; Chua, A.; Zhao, X.; Tan, H. S.; Chia, A.; Le Bert, N.; Low, D.; Tan, H. K.; Kumar, R.; Irani, F. G.; Ho, Z. Z.; Zhang, Q.; Guccione, E.; Wai, L. E.; Koh, S.; Hwang, W.; Chow, W. C.; Bertoletti, A. | Use of Expression Profiles of HBV-DNA Integrated Into Genomes of Hepatocellular Carcinoma Cells to Select T Cells for Immunotherapy | 2019 | May | Gastroenterology | 156 | 6 | 1862-1876.e9 | 10.1053/j.gastro.2019.01.251 |  |
| 315 | Tarantino, L.; Busto, G.; Nasto, A.; Fristachi, R.; Cacace, L.; Talamo, M.; Accardo, C.; Bortone, S.; Gallo, P.; Tarantino, P.; Nasto, R. A.; Di Minno, M. N.; Ambrosino, P. | Percutaneous electrochemotherapy in the treatment of portal vein tumor thrombosis at hepatic hilum in patients with hepatocellular carcinoma in cirrhosis: A feasibility study | 2017 | 7-Feb | World J Gastroenterol | 23 | 5 | 906-918 | 10.3748/wjg.v23.i5.906 |  |
| 316 | Tasu, J. P.; Tougeron, D.; Rols, M. P. | Irreversible electroporation and electrochemotherapy in oncology: State of the art | 2022 | Nov | Diagn Interv Imaging | 103 | 11 | 499-509 | 10.1016/j.diii.2022.09.009 |  |
| 317 | Thuluvath, P. J.; To, C.; Amjad, W. | Role of Locoregional Therapies in Patients With Hepatocellular Cancer Awaiting Liver Transplantation | 2021 | 1-Jan | Am J Gastroenterol | 116 | 1 | 57-67 | 10.14309/ajg.0000000000000999 |  |
| 318 | Tian, G.; Zhao, Q.; Chen, F.; Jiang, T.; Wang, W. | Ablation of hepatic malignant tumors with irreversible electroporation: A systematic review and meta-analysis of outcomes | 2017 | 24-Jan | Oncotarget | 8 | 4 | 5853-5860 | 10.18632/oncotarget.14030 | Wrong patient population |
| 319 | Tian, L.; Qiao, Y.; Lee, P.; Wang, L.; Chang, A.; Ravi, S.; Rogers, T. A.; Lu, L.; Singhana, B.; Zhao, J.; Melancon, M. P. | Antitumor efficacy of liposome-encapsulated NVP-BEZ 235 in combination with irreversible electroporation | 2018 | Nov | Drug Deliv | 25 | 1 | 668-678 | 10.1080/10717544.2018.1444683 |  |
| 320 | Trabold, B.; Wiggermann, P.; Akyol, D.; Jung, E. M.; Nieaÿen, C.; Stroszczynski, C.; Graf, B. M.; Wiese, C. | Anesthesia for irreversible electroporation of hepatic malignant tumors | 2013 | Aug | J Clin Anesth | 25 | 5 | 430-431 | 10.1016/j.jclinane.2013.03.010 |  |
| 321 | Trotovšek, B.; Djokić, M.; Čemažar, M.; Serša, G. | New era of electrochemotherapy in treatment of liver tumors in conjunction with immunotherapies | 2021 | 28-Dec | World J Gastroenterol | 27 | 48 | 8216-8226 | 10.3748/wjg.v27.i48.8216 |  |
| 322 | Vailas, M.; Syllaios, A.; Hashemaki, N.; Sotiropoulou, M.; Schizas, D.; Papalampros, A.; Felekouras, E.; Pikoulis, E. | Irreversible electroporation and sarcomas: where do we stand? | 2019 | Jul-Aug | J Buon | 24 | 4 | 1354-1359 |  |  |
| 323 | Verloh, N.; Jensch, I.; Lürken, L.; Haimerl, M.; Dollinger, M.; Renner, P.; Wiggermann, P.; Werner, J. M.; Zeman, F.; Stroszczynski, C.; Beyer, L. P. | Similar complication rates for irreversible electroporation and thermal ablation in patients with hepatocellular tumors | 2019 | 3-Mar | Radiol Oncol | 53 | 1 | 116-122 | 10.2478/raon-2019-0011 | Wrong patient population |
| 324 | Vivas, I.; Iribarren, K.; Lozano, T.; Cano, D.; Lasarte-Cia, A.; Chocarro, S.; Gorraiz, M.; Sarobe, P.; Hervás-Stubbs, S.; Bilbao, J. I.; Casares, N.; Lasarte, J. J. | Therapeutic Effect of Irreversible Electroporation in Combination with Poly-ICLC Adjuvant in Preclinical Models of Hepatocellular Carcinoma | 2019 | Jul | J Vasc Interv Radiol | 30 | 7 | 1098-1105 | 10.1016/j.jvir.2019.02.023 |  |
| 325 | Vogel, J. A.; van Veldhuisen, E.; Agnass, P.; Crezee, J.; Dijk, F.; Verheij, J.; van Gulik, T. M.; Meijerink, M. R.; Vroomen, L. G.; van Lienden, K. P.; Besselink, M. G. | Time-Dependent Impact of Irreversible Electroporation on Pancreas, Liver, Blood Vessels and Nerves: A Systematic Review of Experimental Studies | 2016 |  | Plos One | 11 | 11 | e0166987 | 10.1371/journal.pone.0166987 |  |
| 326 | Vogel, J. A.; van Veldhuisen, E.; Alles, L. K.; Busch, O. R.; Dijk, F.; van Gulik, T. M.; Huijzer, G. M.; Besselink, M. G.; van Lienden, K. P.; Verheij, J. | Time-Dependent Impact of Irreversible Electroporation on Pathology and Ablation Size in the Porcine Liver: A 24-Hour Experimental Study | 2019 | 1-Jan | Technol Cancer Res Treat | 18 |  | 1.53E+15 | 10.1177/1533033819876899 |  |
| 327 | Vogl, T. J.; Emam, A.; Naguib, N. N.; Eichler, K.; Zangos, S. | How Effective Are Percutaneous Liver-Directed Therapies in Patients with Non-Colorectal Liver Metastases? | 2015 | Dec | Viszeralmedizin | 31 | 6 | 406-13 | 10.1159/000440677 |  |
| 328 | Vollherbst, D.; Bertheau, R. C.; Fritz, S.; Mogler, C.; Kauczor, H. U.; Ryschich, E.; Radeleff, B. A.; Pereira, P. L.; Sommer, C. M. | Electrochemical Effects after Transarterial Chemoembolization in Combination with Percutaneous Irreversible Electroporation: Observations in an Acute Porcine Liver Model | 2016 | Jun | J Vasc Interv Radiol | 27 | 6 | 913-921.e2 | 10.1016/j.jvir.2016.02.001 |  |
| 329 | Vollherbst, D.; Fritz, S.; Zelzer, S.; Wachter, M. F.; Wolf, M. B.; Stampfl, U.; Gnutzmann, D.; Bellemann, N.; Schmitz, A.; Knapp, J.; Pereira, P. L.; Kauczor, H. U.; Werner, J.; Radeleff, B. A.; Sommer, C. M. | Specific CT 3D rendering of the treatment zone after Irreversible Electroporation (IRE) in a pig liver model: the "Chebyshev Center Concept" to define the maximum treatable tumor size | 2014 | 10-Jan | Bmc Med Imaging | 14 |  | 2 | 10.1186/1471-2342-14-2 |  |
| 330 | Vroomen, L. G. P. H.; Petre, E. N.; Cornelis, F. H.; Solomon, S. B.; Srimathveeravalli, G. | Irreversible electroporation and thermal ablation of tumors in the liver, lung, kidney and bone: What are the differences? | 2017 | Sep | Diagn Interv Imaging | 98 | 9 | 609-617 | 10.1016/j.diii.2017.07.007 |  |
| 331 | Wade, R.; South, E.; Anwer, S.; Sharif-Hurst, S.; Harden, M.; Fulbright, H.; Hodgson, R.; Dias, S.; Simmonds, M.; Rowe, I.; Thornton, P.; Eastwood, A. | Ablative and non-surgical therapies for early and very early hepatocellular carcinoma: a systematic review and network meta-analysis | 2023 | Dec | Health Technol Assess | 27 | 29 | 1-172 | 10.3310/gk5221 | Wrong study design |
| 332 | Wagstaff, P. G.; Buijs, M.; van den Bos, W.; de Bruin, D. M.; Zondervan, P. J.; de la Rosette, J. J.; Laguna Pes, M. P. | Irreversible electroporation: state of the art | 2016 |  | Onco Targets Ther | 9 |  | 2437-46 | 10.2147/ott.S88086 |  |
| 333 | Wan, T.; Zhong, J.; Pan, Q.; Zhou, T.; Ping, Y.; Liu, X. | Exosome-mediated delivery of Cas9 ribonucleoprotein complexes for tissue-specific gene therapy of liver diseases | 2022 | 16-Sep | Sci Adv | 8 | 37 | eabp9435 | 10.1126/sciadv.abp9435 |  |
| 334 | Wang, H.; Wang, X.; Ye, X.; Ju, Y.; Cao, N.; Wang, S.; Cai, J. | Nonviral mcDNA-mediated bispecific CAR T cells kill tumor cells in an experimental mouse model of hepatocellular carcinoma | 2022 | 25-Jul | Bmc Cancer | 22 | 1 | 814 | 10.1186/s12885-022-09861-1 |  |
| 335 | Wang, K.; Wang, C.; Jiang, H.; Zhang, Y.; Lin, W.; Mo, J.; Jin, C. | Combination of Ablation and Immunotherapy for Hepatocellular Carcinoma: Where We Are and Where to Go | 2021 |  | Front Immunol | 12 |  | 792781 | 10.3389/fimmu.2021.792781 | Wrong outcomes |
| 336 | Wang, L.; Liu, B. X.; Long, H. Y. | Ablative strategies for recurrent hepatocellular carcinoma | 2023 | 27-Apr | World J Hepatol | 15 | 4 | 515-524 | 10.4254/wjh.v15.i4.515 |  |
| 337 | Wang, L.; Xu, J.; Yu, J.; Liang, P. | Review of clinical tumor ablation advance in Asia | 2021 |  | Int J Hyperthermia | 38 | 1 | 1639-1649 | 10.1080/02656736.2021.1983037 |  |
| 338 | Wang, W.; Hou, S.; Ni, J.; Sun, H.; Jiang, X.; Chen, Y.; Xu, L. | Effectiveness and safety of irreversible electroporation for recurrent hepatocellular carcinoma ineligible for thermal ablation after surgery | 2020 | Sep | J Interv Med | 3 | 3 | 151-155 | 10.1016/j.jimed.2020.07.009 | Wrong patient population |
| 339 | Wang, X.; Su, Z.; Lyu, T.; Figini, M.; Procissi, D.; Shangguan, J.; Sun, C.; Wang, B.; Shang, N.; Gu, S.; Ma, Q.; Gordon, A. C.; Lin, K.; Wang, J.; Lewandowski, R. J.; Salem, R.; Yaghmai, V.; Larson, A. C.; Zhang, Z. | (18)F-FDG PET Biomarkers Help Detect Early Metabolic Response to Irreversible Electroporation and Predict Therapeutic Outcomes in a Rat Liver Tumor Model | 2018 | Apr | Radiology | 287 | 1 | 137-145 | 10.1148/radiol.2017170920 |  |
| 340 | Wang, Z.; Lu, J.; Huang, W.; Wu, Z.; Gong, J.; Wang, Q.; Liu, Q.; Wang, C.; Zhu, Y.; Ding, X.; Wang, Z. | A retrospective study of CT-guided percutaneous irreversible electroporation (IRE) ablation: clinical efficacy and safety | 2021 | 5-Feb | Bmc Cancer | 21 | 1 | 124 | 10.1186/s12885-021-07820-w |  |
| 341 | Wardhana, G.; Almeida, J. P.; Abayazid, M.; Fütterer, J. J. | Development of a thermal model for irreversible electroporation: an approach to estimate and optimize the IRE protocols | 2021 | Aug | Int J Comput Assist Radiol Surg | 16 | 8 | 1325-1334 | 10.1007/s11548-021-02403-3 |  |
| 342 | Wei, Y.; Xiao, Y.; Wang, Z.; Hu, X.; Chen, G.; Ding, X.; Fan, Y.; Han, Y.; Huang, K.; Huang, X.; Kuang, M.; Lang, X.; Li, H.; Li, C.; Li, J.; Li, J.; Li, M.; Lu, Y.; Ni, C.; Niu, L.; Sun, J.; Tian, J.; Wang, H.; Wang, L.; Wu, P.; Xie, X.; Xing, W.; Xu, L.; Yang, P.; Yu, H.; Yuan, C.; Zhai, B.; Zhang, Y.; Zheng, J.; Zhou, Z.; Zhu, X.; Jiang, T.; Zhang, Y. | Chinese expert consensus of image-guided irreversible electroporation for pancreatic cancer | 2021 | Jul | J Cancer Res Ther | 17 | 3 | 613-618 | 10.4103/jcrt.JCRT_1663_20 |  |
| 343 | Weiss, J.; Garnon, J.; Dalili, D.; Cazzato, R. L.; Koch, G.; Auloge, P.; Gangi, A. | The Feasibility of Combined Microwave Ablation and Irreversible Electroporation for Central Liver Metastase | 2021 | Jun | Cardiovasc Intervent Radiol | 44 | 6 | 999-1001 | 10.1007/s00270-021-02790-9 |  |
| 344 | Wichtowski, M.; Nowaczyk, P.; Kocur, J.; Murawa, D. | Irreversible electroporation in the treatment of locally advanced pancreas and liver metastases of colorectal carcinoma | 2016 |  | Contemp Oncol (Pozn) | 20 | 1 | 39-44 | 10.5114/wo.2016.57815 |  |
| 345 | Wierzbicki, R.; Pawå‚Owicz, M.; Job, J.; Balawender, R.; Kostarczyk, W.; Stanuch, M.; Janc, K.; Skalski, A. | 3D mixed-reality visualization of medical imaging data as a supporting tool for innovative, minimally invasive surgery for gastrointestinal tumors and systemic treatment as a new path in personalized treatment of advanced cancer diseases | 2022 | Jan | J Cancer Res Clin Oncol | 148 | 1 | 237-243 | 10.1007/s00432-021-03680-w |  |
| 346 | Wierzbicki, R.; Pawå‚Owicz, M.; Job, J.; Balawender, R.; Kostarczyk, W.; Stanuch, M.; Janc, K.; Skalski, A. | 3D mixed-reality visualization of medical imaging data as a supporting tool for innovative, minimally invasive surgery for gastrointestinal tumors and systemic treatment as a new path in personalized treatment of advanced cancer diseases | 2022 | Jan | J Cancer Res Clin Oncol | 148 | 1 | 237-243 | 10.1007/s00432-021-03680-w |  |
| 347 | Wiggermann, P.; Zeman, F.; Niessen, C.; Agha, A.; Trabold, B.; Stroszczynski, C.; Jung, E. M. | Percutaneous irreversible electroporation (IRE) of hepatic malignant tumours: contrast-enhanced ultrasound (CEUS) findings | 2012 |  | Clin Hemorheol Microcirc | 52 | 4-Feb | 417-27 | 10.3233/ch-2012-1615 |  |
| 348 | Wu, L. M.; Zhang, L. L.; Chen, X. H.; Zheng, S. S. | Is irreversible electroporation safe and effective in the treatment of hepatobiliary and pancreatic cancers? | 2019 | Apr | Hepatobiliary Pancreat Dis Int | 18 | 2 | 117-124 | 10.1016/j.hbpd.2019.01.001 |  |
| 349 | Xiao, D.; Yao, C.; Liu, H.; Li, C.; Cheng, J.; Guo, F.; Tang, L. | Irreversible electroporation and apoptosis in human liver cancer cells induced by nanosecond electric pulses | 2013 | Oct | Bioelectromagnetics | 34 | 7 | 512-20 | 10.1002/bem.21796 |  |
| 350 | Xiu, H.; Nan, X.; Guo, D.; Wang, J.; Li, J.; Peng, Y.; Xiong, G.; Wang, S.; Wang, C.; Zhang, G.; Yang, Y.; Cai, Z. | Gp350-anchored extracellular vesicles: promising vehicles for delivering therapeutic drugs of B cell malignancies | 2022 | May | Asian J Pharm Sci | 17 | 3 | 462-474 | 10.1016/j.ajps.2022.03.004 |  |
| 351 | Xu, M.; Xie, L. T.; Xiao, Y. Y.; Liang, P.; Zhao, Q. Y.; Wang, Z. M.; Chai, W. L.; Wei, Y. T.; Xu, L. F.; Hu, X. K.; Kuang, M.; Niu, L. Z.; Yao, C. G.; Kong, H. Y.; Tian, G.; Xie, X. Y.; Cui, X. W.; Xu, D.; Zhao, J.; Jiang, T. A. | Chinese clinical practice guidelines for ultrasound-guided irreversible electroporation of liver cancer (version 2022) | 2022 | Oct | Hepatobiliary Pancreat Dis Int | 21 | 5 | 462-471 | 10.1016/j.hbpd.2022.08.006 |  |
| 352 | Xu, M.; Xu, D.; Dong, G.; Ren, Z.; Zhang, W.; Aji, T.; Zhao, Q.; Chen, X.; Jiang, T. | The Safety and Efficacy of Nanosecond Pulsed Electric Field in Patients With Hepatocellular Carcinoma: A Prospective Phase 1 Clinical Study Protocol | 2022 |  | Front Oncol | 12 |  | 869316 | 10.3389/fonc.2022.869316 |  |
| 353 | Xu, R. C.; Wang, F.; Sun, J. L.; Abuduwaili, W.; Zhang, G. C.; Liu, Z. Y.; Liu, T. T.; Dong, L.; Shen, X. Z.; Zhu, J. M. | A novel murine model of combined hepatocellular carcinoma and intrahepatic cholangiocarcinoma | 2022 | 9-Dec | J Transl Med | 20 | 1 | 579 | 10.1186/s12967-022-03791-z |  |
| 354 | Yang, F.; Zheng, X.; Koh, S.; Lu, J.; Cheng, J.; Li, P.; Du, C.; Chen, Y.; Chen, X.; Yang, L.; Chen, W.; Wong, R. W.; Wai, L. E.; Wang, T.; Zhang, Q.; Chen, W. | Messenger RNA electroporated hepatitis B virus (HBV) antigen-specific T cell receptor (TCR) redirected T cell therapy is well-tolerated in patients with recurrent HBV-related hepatocellular carcinoma post-liver transplantation: results from a phase I trial | 2023 | Aug | Hepatol Int | 17 | 4 | 850-859 | 10.1007/s12072-023-10524-x |  |
| 355 | Yang, Y.; Qin, Z.; Du, D.; Wu, Y.; Qiu, S.; Mu, F.; Xu, K.; Chen, J. | Safety and Short-Term Efficacy of Irreversible Electroporation and Allogenic Natural Killer Cell Immunotherapy Combination in the Treatment of Patients with Unresectable Primary Liver Cancer | 2019 | Jan | Cardiovasc Intervent Radiol | 42 | 1 | 48-59 | 10.1007/s00270-018-2069-y | Wrong patient population |
| 356 | Yeung, E. S.; Chung, M. W.; Wong, K.; Wong, C. Y.; So, E. C.; Chan, A. C. | An update on irreversible electroporation of liver tumours | 2014 | Aug | Hong Kong Med J | 20 | 4 | 313-6 | 10.12809/hkmj134190 | Wrong patient population |
| 357 | Yu, M.; Li, S. | Irreversible electroporation for liver cancer ablation: A meta analysis | 2021 | 15-Dec | Eur J Surg Oncol |  |  |  | 10.1016/j.ejso.2021.12.015 | Wrong study design |
| 358 | Yu, M.; Li, S. | Irreversible electroporation for liver cancer ablation: A meta analysis | 2022 | Jun | Eur J Surg Oncol | 48 | 6 | 1321-1330 | 10.1016/j.ejso.2021.12.015 | Wrong study design |
| 359 | Yun, J. H.; Fang, A.; Khorshidi, F.; Habibollahi, P.; Kutsenko, O.; Etezadi, V.; Hunt, S.; Nezami, N. | New Developments in Image-Guided Percutaneous Irreversible Electroporation of Solid Tumors | 2023 | Nov | Curr Oncol Rep | 25 | 11 | 1213-1226 | 10.1007/s11912-023-01452-y |  |
| 360 | Zeng, J.; Liu, G.; Li, Z. H.; Yang, Y.; Fang, G.; Li, R. R.; Xu, K. C.; Niu, L. | The Safety and Efficacy of Irreversible Electroporation for Large Hepatocellular Carcinoma | 2017 | Feb | Technol Cancer Res Treat | 16 | 1 | 120-124 | 10.1177/1533034616676445 | Wrong patient population |
| 361 | Zeng, J.; Qin, Z.; Zhou, L.; Fang, G.; Chen, J.; Li, J.; Niu, L.; Liang, B.; Xu, K. | Comparison between cryoablation and irreversible electroporation of rabbit livers at a location close to the gallbladder | 2017 | 1-Mar | Radiol Oncol | 51 | 1 | 40-46 | 10.1515/raon-2017-0003 |  |
| 362 | Zhang, S.; Chen, S.; Zhu, R. | Electroporation-Assisted Surface-Enhanced Raman Detection for Long-Term, Label-Free, and Noninvasive Molecular Profiling of Live Single Cells | 2023 | 24-Feb | Acs Sens | 8 | 2 | 555-564 | 10.1021/acssensors.2c01582 |  |
| 363 | Zhang, T.; Chen, J.; Niu, L.; Liu, Y.; Ye, G.; Jiang, M.; Qi, Z. | Clinical Safety and Efficacy of Locoregional Therapy Combined with Adoptive Transfer of Allogeneic γδ T Cells for Advanced Hepatocellular Carcinoma and Intrahepatic Cholangiocarcinoma | 2022 | Jan | J Vasc Interv Radiol | 33 | 1 | 19-27.e3 | 10.1016/j.jvir.2021.09.012 |  |
| 364 | Zhang, T.; Chen, J.; Niu, L.; Liu, Y.; Ye, G.; Jiang, M.; Qi, Z. | Clinical Safety and Efficacy of Locoregional Therapy Combined with Adoptive Transfer of Allogeneic γδ T Cells for Advanced Hepatocellular Carcinoma and Intrahepatic Cholangiocarcinoma | 2022 | Jan | J Vasc Interv Radiol | 33 | 1 | 19-27.e3 | 10.1016/j.jvir.2021.09.012 | Wrong patient population |
| 365 | Zhang, Xiaobo; Zhang, Xiao; Ding, Xiaoyi; Wang, Zhongmin; Fan, Yong; Chen, Guang; Hu, Xiaokun; Zheng, Jiasheng; Xue, Zhixiao; He, Xiaofeng | Novel irreversible electroporation ablation (Nano-knife) versus radiofrequency ablation for the treatment of solid liver tumors: a comparative, randomized, multicenter clinical study | 2022 |  | Frontiers In Oncology | 12 |  | 945123 |  | Wrong patient population |
| 366 | Zhang, Y.; Lv, Y.; Wang, Y.; Chang, T. T.; Rubinsky, B. | Pancreatic islets implanted in an irreversible electroporation generated extracellular matrix in the liver | 2023 | 1-Mar | Radiol Oncol | 57 | 1 | 51-58 | 10.2478/raon-2023-0006 |  |
| 367 | Zhang, Y.; White, S. B.; Nicolai, J. R.; Zhang, Z.; West, D. L.; Kim, D. H.; Goodwin, A. L.; Miller, F. H.; Omary, R. A.; Larson, A. C. | Multimodality imaging to assess immediate response to irreversible electroporation in a rat liver tumor model | 2014 | Jun | Radiology | 271 | 3 | 721-9 | 10.1148/radiol.14130989 |  |
| 368 | Zhao, J.; Qiao, J.; Zhou, M.; Gupta, S.; Li, C.; Melancon, M. P. | Anti-tumor Efficacy Study using Irreversible Electroporation and Doxorubicin-loaded Polymeric Micelles | 2015 | 20-Oct | Acs Macro Lett | 4 | 10 | 1081-1084 | 10.1021/acsmacrolett.5b00545 |  |
| 369 | Zhou, L.; Yin, S.; Chai, W.; Zhao, Q.; Tian, G.; Xu, D.; Jiang, T. | Irreversible electroporation in patients with liver tumours: treated-area patterns with contrast-enhanced ultrasound | 2020 | 23-Nov | World J Surg Oncol | 18 | 1 | 305 | 10.1186/s12957-020-02083-4 |  |
| 370 | Zhu, F.; Rhim, H. | Thermal ablation for hepatocellular carcinoma: what's new in 2019 | 2019 | Dec | Chin Clin Oncol | 8 | 6 | 58 | 10.21037/cco.2019.11.03 |  |
| 371 | Zimmerman, A.; Grand, D.; Charpentier, K. P. | Irreversible electroporation of hepatocellular carcinoma: patient selection and perspectives | 2017 |  | J Hepatocell Carcinoma | 4 |  | 49-58 | 10.2147/jhc.S129063 |  |
| 372 | Zou, Y. W.; Ren, Z. G.; Sun, Y.; Liu, Z. G.; Hu, X. B.; Wang, H. Y.; Yu, Z. J. | The latest research progress on minimally invasive treatments for hepatocellular carcinoma | 2023 | Feb | Hepatobiliary Pancreat Dis Int | 22 | 1 | 54-63 | 10.1016/j.hbpd.2022.08.004 |  |
