# Supplementary material for: Clinical and safety outcomes in unresectable, very early and early-stage hepatocellular carcinoma following Irreversible Electroporation (IRE) and Transarterial Chemoembolization (TACE): A systematic literature review and meta-analysis
Source: PLoS One. 2025 Apr 29;20(4):e0322113. doi: 10.1371/journal.pone.0322113 (PMC12083900; doi:10.1371/journal.pone.0322113)
Supplement: S19 Table — (DOCX) [file pone.0322113.s019.docx]

# S19 Table. All Unique Studies Identified, TACE SLR

| **Number** | **Authors** | **Title** | **Year** | **Month** | **Journal** | **Volume** | **Issue** | **Pages** | **DOI** | **Exclusion Reason (Full Text)** |
| --- | --- | --- | --- | --- | --- | --- | --- | --- | --- | --- |
| 1 | Alan, A. M.; Alan, O.; Asadov, R.; Demirtas, C. O.; Kani, H. T.; Yumuk, P. F.; Ozdogan, O. C.; Baltacioglu, F.; Gunduz, F. | Evaluation of the effectiveness of drug-eluting transarterial chemoebolization in hepatocellular carcinoma | 2023 | Mar | Hepatol Forum | 4 | 2 | 53-60 | 10.14744/hf.2022.2022.0048 |  |
| 2 | Bai, M.; Pan, T.; Zhou, C.; Li, M. A.; Chen, J.; Zeng, Z.; Zhu, D.; Wu, C.; Jiang, Z.; Li, Z.; Huang, M. | Transarterial chemoembolization with pirarubicin-eluting microspheres in patients with unresectable hepatocellular carcinoma: Preliminary results | 2019 | May | J Interv Med | 2 | 2 | 69-77 | 10.1016/j.jimed.2019.09.005 |  |
| 3 | Bargellini, Irene; Sacco, Rodolfo; Bozzi, Elena; Bertini, Marco; Ginanni, Barbara; Romano, Antonio; Cicorelli, Antonio; Tumino, Emanuele; Federici, Graziana; Cioni, Roberto | Transarterial chemoembolization in very early and early-stage hepatocellular carcinoma patients excluded from curative treatment: a prospective cohort study | 2012 |  | European journal of radiology | 81 | 6 | 1173-1178 |  |  |
| 4 | Cathomas, Marionna; Mueller, Flavian; Mertineit, Nando; Baumgartner, Iris; Candinas, Daniel; Berzigotti, Annalisa; Maurer, Martin H; Lachenmayer, Anja | Comparison of transarterial bland embolization and drug-eluting beads transarterial chemoembolization for very early and early hepatocellular carcinoma not amenable for surgery or ablation: a single center retrospective data analysis | 2023 |  | Journal of gastrointestinal oncology | 14 | 5 | 2167 |  |  |
| 5 | Chen, R. X.; Gan, Y. H.; Ge, N. L.; Chen, Y.; Ma, H.; Wang, Y.; Zhang, B. H.; Wang, Y. H.; Ye, S. L.; Luo, J. F.; Ren, Z. G. | Comparison of transarterial chemoembolization with radiofrequency ablation for unresectable Barcelona Clinic Liver Cancer stage 0/A hepatocellular carcinoma: a propensity score matching | 2016 | Feb | J Gastroenterol Hepatol | 31 | 2 | 442-9 | 10.1111/jgh.13077 |  |
| 6 | Chen, S.; Yu, W.; Zhang, K.; Liu, W.; Chen, Q. | Transarterial chemoembolization for unresectable hepatocellular carcinoma: A comparison of the efficacy and safety of 2 embolic agents | 2018 | May | Medicine (Baltimore) | 97 | 21 | e10832 | 10.1097/md.0000000000010832 |  |
| 7 | Chu, H. H.; Gwon, D. I.; Kim, J. H.; Ko, G. Y.; Shin, J. H.; Yoon, H. K. | Drug-Eluting Microsphere Versus Cisplatin-Based Transarterial Chemoembolization for the Treatment of Hepatocellular Carcinoma: Propensity Score-Matched Analysis | 2020 | Sep | AJR Am J Roentgenol | 215 | 3 | 745-752 | 10.2214/ajr.19.21669 |  |
| 8 | Golfieri, R.; Giampalma, E.; Renzulli, M.; Cioni, R.; Bargellini, I.; Bartolozzi, C.; Breatta, A D; Gandini, G.; Nani, R.; Gasparini, D.; Cucchetti, A.; Bolondi, L.; Trevisani, F.; on behalf of the PRECISION ITALIA STUDY GROUP | Randomised controlled trial of doxorubicin-eluting beads vs conventional chemoembolisation for hepatocellular carcinoma | 2014 |  | British Journal of Cancer | 111 | 2 | 255-264 | 10.1038/bjc.2014.199 |  |
| 9 | Hashem, E; Sait, S; Thomas, D Nicholson; Watson, C; Moeen, S; Peddu, P | Transarterial chemoembolisation for very early and early stage hepatocellular carcinoma: single-centre experience | 2023 |  | Clinical Radiology | 78 | 2 | e113-e122 |  |  |
| 10 | Haubold, J.; Reinboldt, M. P.; Wetter, A.; Li, Y.; Ludwig, J. M.; Lange, C.; Wedemeyer, H.; Schotten, C.; Umutlu, L.; Theysohn, J. | DSM-TACE of HCC: Evaluation of Tumor Response in Patients Ineligible for Other Systemic or Loco-Regional Therapies | 2020 | Sep | Rofo | 192 | 9 | 862-869 | 10.1055/a-1111-9955 |  |
| 11 | Huo, Y. R.; Xiang, H.; Chan, M. V.; Chan, C. | Survival, tumour response and safety of 70-150 µm versus 100-300 µm doxorubicin drug-eluting beads in transarterial chemoembolisation for hepatocellular carcinoma | 2019 | Dec | J Med Imaging Radiat Oncol | 63 | 6 | 802-811 | 10.1111/1754-9485.12971 |  |
| 12 | Hyun, Dongho; Cho, Sung Ki; Shin, Sung Wook; Park, Kwang Bo; Park, Hong Suk; Choo, Sung Wook; Do, Young Soo; Choo, In-wook; Lee, Min Woo; Rhim, Hyunchul | Early stage hepatocellular carcinomas not feasible for ultrasound-guided radiofrequency ablation: comparison of transarterial chemoembolization alone and combined therapy with transarterial chemoembolization and radiofrequency ablation | 2016 |  | CardioVascular and Interventional Radiology | 39 | 3 | 417-425 |  |  |
| 13 | Iezzi, R.; Pompili, M.; Posa, A.; Carchesio, F.; Siciliano, M.; Annicchiarico, B. E.; Agnes, S.; Giuliante, F.; Garcovich, M.; Cerrito, L.; Ponziani, F. R.; Basso, M.; Cassano, A.; Rapaccini, G. L.; De Gaetano, A. M.; Gasbarrini, A.; Manfredi, R. | Interventional oncology treatments for unresectable early stage HCC in patients with a high risk for intraprocedural bleeding: Is a single-step combined therapy safe and feasible? | 2019 | May | Eur J Radiol | 114 |  | 32-37 | 10.1016/j.ejrad.2019.02.030 |  |
| 14 | Ikeda, M.; Arai, Y.; Inaba, Y.; Tanaka, T.; Sugawara, S.; Kodama, Y.; Aramaki, T.; Anai, H.; Morita, S.; Tsukahara, Y.; Seki, H.; Sato, M.; Kamimura, K.; Azama, K.; Tsurusaki, M.; Sugihara, E.; Miyazaki, M.; Kobayashi, T.; Sone, M. | Conventional or Drug-Eluting Beads? Randomized Controlled Study of Chemoembolization for Hepatocellular Carcinoma: JIVROSG-1302 | 2022 | Sep | Liver Cancer | 11 | 5 | 440-450 | 10.1159/000525500 |  |
| 15 | Imai, Y.; Chikayama, T.; Nakazawa, M.; Watanabe, K.; Ando, S.; Mizuno, Y.; Yoshino, K.; Sugawara, K.; Hamaoka, K.; Fujimori, K.; Inao, M.; Nakayama, N.; Oka, M.; Nagoshi, S.; Mochida, S. | Usefulness of miriplatin as an anticancer agent for transcatheter arterial chemoembolization in patients with unresectable hepatocellular carcinoma | 2012 | Feb | J Gastroenterol | 47 | 2 | 179-86 | 10.1007/s00535-011-0475-x |  |
| 16 | Jiang, J. Q.; Huang, J. T.; Zhong, B. Y.; Wang, W. D.; Sun, J. H.; Wang, Q.; Ding, W. B.; Ni, C. F.; Zhu, X. L. | Transarterial Chemoembolization for Patients with Unresectable Hepatocellular Carcinoma with Child-Pugh B7 | 2023 |  | J Hepatocell Carcinoma | 10 |  | 1629-1638 | 10.2147/jhc.S422300 |  |
| 17 | Kim, Jong Woo; Kim, Jin Hyoung; Sung, Kyu-bo; Ko, Heung-kyu; Shin, Ji Hoon; Kim, Pyo Nyun; Choi, Hyun-kyung; Ko, Gi-young; Yoon, Hyun-ki; Chun, Seng-yong; Gwon, Dong Il | Transarterial Chemoembolization vs. Radiofrequency Ablation for the Treatment of Single Hepatocellular Carcinoma 2 cm or Smaller | 2014 |  | The American Journal of Gastroenterology | 109 | 8 | 1234-1240 | <http://dx.doi.org/10.1038/ajg.2014.152> |  |
| 18 | Kudo, Masatoshi; Ueshima, Kazuomi; Ikeda, Masafumi; Torimura, Takuji; Tanabe, Nobukazu; Aikata, Hiroshi; Izumi, Namiki; Yamasaki, Takahiro; Nojiri, Shunsuke; Hino, Keisuke | Randomised, multicentre prospective trial of transarterial chemoembolisation (TACE) plus sorafenib as compared with TACE alone in patients with hepatocellular carcinoma: TACTICS trial | 2020 |  | Gut | 69 | 8 | 1492-1501 |  |  |
| 19 | Lee, Moonhyung; Shin, Hyun Phil | Efficacy of Transarterial Chemoembolization (TACE) for Early-Stage Hepatocellular Carcinoma | 2023 |  | Medicina | 59 | 12 | 2174 |  |  |
| 20 | Lee, Myungsu; Chung, Jin Wook; Lee, Kwang-Hun; Won, Jong Yun; Chun, Ho Jong; Lee, Han Chu; Kim, Jin Hyoung; Lee, In Joon; Hur, Saebeom; Kim, Hyo-Cheol; Kim, Yoon Jun; Kim, Gyoung Min; Joo, Seung-Moon; Oh, Jung Suk | Korean Multicenter Registry of Transcatheter Arterial Chemoembolization with Drug-Eluting Embolic Agents for Nodular Hepatocellular Carcinomas: Six-Month Outcome Analysis | 2017 |  | Journal of Vascular and Interventional Radiology | 28 | 4 | 502-512 | <https://doi.org/10.1016/j.jvir.2016.08.017> |  |
| 21 | Lee, Yong Kang; Jung, Kyu Sik; Kim, Do Young; Choi, Jin Young; Kim, Beom Kyung; Kim, Seung Up; Park, Jun Yong; Ahn, Sang Hoon; Han, Kwang-Hyub; Kim, Gyoung Min; Kim, Man Deuk; Park, Sung Il; Won, Jong Yun; Lee, Do Yun | Conventional versus drug-eluting beads chemoembolization for hepatocellular carcinoma: Emphasis on the impact of tumor size | 2017 |  | Journal of Gastroenterology and Hepatology | 32 | 2 | 487-496 | <https://doi.org/10.1111/jgh.13501> |  |
| 22 | Manini, Matteo Angelo; Sangiovanni, Angelo; Martinetti, Laura; Viganò, Davide; La Mura, Vincenzo; Aghemo, Alessio; Iavarone, Massimo; Crespi, Silvia; Nicolini, Antonio; Colombo, Massimo | Transarterial chemoembolization with drug-eluting beads is effective for the maintenance of the Milan-in status in patients with a small hepatocellular carcinoma | 2015 |  | Liver Transplantation | 21 | 10 | 1259-1269 | <https://doi.org/10.1002/lt.24196> |  |
| 23 | Ou, H. Y.; Wu, Y. N.; Yu, C. Y.; Chen, C. L.; Hsu, H. W.; Weng, C. C.; Leung-Chit Tsang, L.; Huang, T. L.; Tong, Y. S.; Lim, W. X.; Cheng, Y. F. | Transarterial Chemoembolization Using 100-µm Drug-Eluting Microspheres in Patients with Hepatocellular Carcinoma: A Prospective Study and Midterm Follow-up | 2020 | Nov | J Vasc Interv Radiol | 31 | 11 | 1784-1791 | 10.1016/j.jvir.2020.06.009 |  |
| 24 | Rahman, Abdul; Naidu, J; Ngiu, CS; Yaakob, Y; Mohamed, Z; Othman, H; Jarmin, R; Elias, MH; RA, Raja Ali | Conventional versus doxorubicin-eluting beads transarterial chemoembolization for unresectable hepatocellular carcinoma: a tertiary medical centre experience in Malaysia | 2016 |  | Asian Pacific Journal of Cancer Prevention | | |  |  |  |
| 25 | Razi, Murtuza; Safiullah, Syed; Gu, Jianping; He, Xu; Razi, Mustafa; Kong, Jie | Comparison of tumor response following conventional versus drug-eluting bead transarterial chemoembolization in early-and very early-stage hepatocellular carcinoma | 2022 |  | Journal of Interventional Medicine | 5 | 1 | 14-Oct |  |  |
| 26 | Romero, A. M.; van der Holt, B.; Willemssen, F. E. J. A.; de Man, R. A.; Heijmen, B. J. M.; Habraken, S.; Westerveld, H.; van Delden, O. M.; Klümpen, H.; Tjwa, E. T. T. L. | Transarterial Chemoembolization With Drug-Eluting Beads Versus Stereotactic Body Radiation Therapy for Hepatocellular Carcinoma: Outcomes From a Multicenter, Randomized, Phase 2 Trial (the TRENDY Trial) | 2023 |  | International Journal of Radiation Oncology* Biology* Physics | | | | |  |
| 27 | Sheta, E.; El-Kalla, F.; El-Gharib, M.; Kobtan, A.; Elhendawy, M.; Abd-Elsalam, S.; Mansour, L.; Amer, I. | Comparison of single-session transarterial chemoembolization combined with microwave ablation or radiofrequency ablation in the treatment of hepatocellular carcinoma: a randomized-controlled study | 2016 | Oct | Eur J Gastroenterol Hepatol | 28 | 10 | 1198-203 | 10.1097/meg.0000000000000688 |  |
| 28 | Song, Myeong Jun; Chun, Ho Jong; Kim, Hee Yeon; Yoo, Sun Hong; Park, Chung-Hwa; Bae, Si Hyun; Choi, Jong Young; Im Chang, U; Yang, Jin Mo; Lee, Hae Giu | Comparative study between doxorubicin-eluting beads and conventional transarterial chemoembolization for treatment of hepatocellular carcinoma | 2012 |  | Journal of hepatology | 57 | 6 | 1244-1250 |  |  |
| 29 | Tay, Benjamin Wei Rong; Huang, Daniel Q; Mark, Muthiah; Thong, Neo Wee; Guan Huei, Lee; Gee, Lim Seng; Cheng, Low How; Mei, Lee Yin; Thurairajah, Prem; Chen, Lim Jia | Comparable Outcomes in Early Hepatocellular Carcinomas Treated with Trans-Arterial Chemoembolization and Radiofrequency Ablation | 2022 |  | Biomedicines | 10 | 10 | 2361 |  |  |
| 30 | Tovar-Felice, G.; García-Gámez, A.; Benito-Santamaría, V.; Balaguer-Paniagua, D.; Villalba-Auñón, J.; Sampere-Moragues, J. | Unresectable hepatocellular carcinoma treatment with doxorubicin-eluting polyethylene glycol microspheres: a single-center experience | 2021 | Sep | Hepat Oncol | 8 | 3 | Hep38 | 10.2217/hep-2020-0035 |  |
| 31 | Yun, Byung-Yoon; Lee, Hye Won; Min, In Kyung; Kim, Seung Up; Park, Jun Yong; Kim, Do Young; Ahn, Sang Hoon; Kim, Beom Kyung | Prognosis of early-stage hepatocellular carcinoma: comparison between trans-arterial chemoembolization and radiofrequency ablation | 2020 |  | Cancers | 12 | 9 | 2527 |  |  |
| 32 | Zhang, L.; Hong, W.; Wang, Z.; Zheng, C.; Liang, B.; Shi, H. | Safety and Effectiveness of Transarterial Chemoembolization in Hepatocellular Carcinoma Patients Aged Greater versus Less Than 80 Years | 2023 |  | Clin Interv Aging | 18 |  | 1883-1892 | 10.2147/cia.S429259 |  |
| 33 | Zhang, L.; Sun, J. H.; Ji, J. S.; Zhong, B. Y.; Zhou, G. H.; Song, J. J.; Hou, Z. H.; Huang, P.; Zhang, S.; Li, Z.; Zhu, X. L.; Ni, C. F. | Imaging Changes and Clinical Complications After Drug-Eluting Bead Versus Conventional Transarterial Chemoembolization for Unresectable Hepatocellular Carcinoma: Multicenter Study | 2021 | Oct | AJR Am J Roentgenol | 217 | 4 | 933-943 | 10.2214/ajr.20.24708 |  |
| 34 | Aal, A. K. A.; Moawad, S.; Lune, P. V.; El Khudari, H.; Hanaoka, M. M.; Abouldahab, N.; Gunn, A. J.; White, J.; Shoreibah, M.; Li, Y.; Saddekni, S.; Mahmoud, K. | Survival Outcomes of Very Small Drug-Eluting Beads Used in Chemoembolization of Unresectable Hepatocellular Carcinoma | 2019 | Sep | J Vasc Interv Radiol | 30 | 9 | 1325-1334.e2 | 10.1016/j.jvir.2019.05.006 | Wrong outcomes |
| 35 | Abdelmaksoud, A. H.; Mandooh, S.; Nabeel, M. M.; Elbaz, T. M.; Shousha, H. I.; Monier, A.; Elattar, I. A.; Abdelaziz, A. O. | Portal Vein Thrombosis in Unresectable Hcc Cases: a Single Center Study of Prognostic Factors and Management in 140 Patients | 2017 | 1-Jan | Asian Pac J Cancer Prev | 18 | 1 | 183-188 | 10.22034/apjcp.2017.18.1.183 |  |
| 36 | Abdelrahim, M.; Victor, D.; Esmail, A.; Kodali, S.; Graviss, E. A.; Nguyen, D. T.; Moore, L. W.; Saharia, A.; McMillan, R.; Fong, J. N.; Uosef, A.; Elshawwaf, M.; Heyne, K.; Ghobrial, R. M. | Transarterial Chemoembolization (TACE) Plus Sorafenib Compared to TACE Alone in Transplant Recipients with Hepatocellular Carcinoma: An Institution Experience | 2022 | 27-Jan | Cancers (Basel) | 14 | 3 |  | 10.3390/cancers14030650 |  |
| 37 | Abdelrahim, M.; Victor, D.; Esmail, A.; Kodali, S.; Graviss, E. A.; Nguyen, D. T.; Moore, L. W.; Saharia, A.; McMillan, R.; Fong, J. N. | Transarterial Chemoembolization (TACE) plus sorafenib compared to TACE alone in transplant recipients with hepatocellular carcinoma: An institution experience | 2022 |  | Cancers | 14 | 3 | 650 |  | Wrong patient population |
| 38 | Abdel-Rahman, O. M.; Elsayed, Z. | Yttrium-90 microsphere radioembolisation for unresectable hepatocellular carcinoma | 2016 | 16-Feb | Cochrane Database Syst Rev | 2 |  | Cd011313 | 10.1002/14651858.CD011313.pub2 |  |
| 39 | Abdel-Rahman, O.; Elsayed, Z. | External beam radiotherapy for unresectable hepatocellular carcinoma | 2017 | 7-Mar | Cochrane Database Syst Rev | 3 | 3 | Cd011314 | 10.1002/14651858.CD011314.pub2 |  |
| 40 | Abdel-Rahman, O.; Elsayed, Z. | Yttrium-90 microsphere radioembolisation for unresectable hepatocellular carcinoma | 2020 | 24-Jan | Cochrane Database Syst Rev | 1 | 1 | Cd011313 | 10.1002/14651858.CD011313.pub3 |  |
| 41 | Abdel-Rahman, O.; Elsayed, Z. A. | Combination trans arterial chemoembolization (TACE) plus sorafenib for the management of unresectable hepatocellular carcinoma: a systematic review of the literature | 2013 | Dec | Dig Dis Sci | 58 | 12 | 3389-96 | 10.1007/s10620-013-2872-x |  |
| 42 | Abi-Jaoudeh, N.; Dayyani, F.; Chen, P. J.; Fernando, D.; Fidelman, N.; Javan, H.; Liang, P. C.; Hwang, J. I.; Imagawa, D. K. | Phase I Trial on Arterial Embolization with Hypoxia Activated Tirapazamine for Unresectable Hepatocellular Carcinoma | 2021 |  | J Hepatocell Carcinoma | 8 | 421-434 |  | 10.2147/jhc.S304275 |  |
| 43 | Adachi, T.; Hiraoka, A.; Okazaki, H.; Nagamatsu, K.; Izumoto, H.; Yoshino, T.; Tsuruta, M.; Aibiki, T.; Okudaira, T.; Yamago, H.; Iwasaki, R.; Suga, Y.; Mori, K.; Miyata, H.; Tsubouchi, E.; Ninomiya, T.; Michitaka, K. | Exacerbation of psoriasis vulgaris by sorafenib treatment for hepatocellular carcinoma | 2020 | Oct | Clin J Gastroenterol | 13 | 5 | 891-895 | 10.1007/s12328-020-01134-3 |  |
| 44 | Agarwal, A.; Yadav, A. K.; Kumar, A.; Gupta, S.; Panwala, H. K.; Redhu, N.; Hariprasad, S.; Ranjan, P.; Arora, A.; Gupta, A. | Transarterial chemoembolization in unresectable hepatocellular carcinoma--assessing the factors affecting the survival: An audit from a tertiary care center in northern India | 2015 | Mar | Indian J Gastroenterol | 34 | 2 | 117-26 | 10.1007/s12664-015-0544-9 |  |
| 45 | Ahmed, F.; Onwumeh-Okwundu, J.; Yukselen, Z.; Endaya Coronel, M. K.; Zaidi, M.; Guntipalli, P.; Garimella, V.; Gudapati, S.; Mezidor, M. D.; Andrews, K.; Mouchli, M.; Shahini, E. | Atezolizumab plus bevacizumab versus sorafenib or atezolizumab alone for unresectable hepatocellular carcinoma: A systematic review | 2021 | 15-Nov | World J Gastrointest Oncol | 13 | 11 | 1813-1832 | 10.4251/wjgo.v13.i11.1813 |  |
| 46 | Ahmed, P.; Naz, N.; Sattar, A.; al Qamari, N.; Ali, I.; Farooq, F.; Asadullah, M.; Kumar, R. | Trans-Arterial Chemoembolization for the Treatment of Hepatocellular Carcinoma: A Single Tertiary Care Institute Experience | 2023 |  | Proceedings | 37 | 3 |  |  | Wrong patient population |
| 47 | Ahmed, S.; de Souza, N. N.; Qiao, W.; Kasai, M.; Keem, L. J.; Shelat, V. G. | Quality of Life in Hepatocellular Carcinoma Patients Treated with Transarterial Chemoembolization | 2016 |  | HPB Surg | 2016 |  | 6120143 | 10.1155/2016/6120143 |  |
| 48 | Ajit, Y.; Sudarsan, H.; Saumya, G.; Abhishek, A.; Navneet, R.; Piyush, R.; Anil, A.; Arun, G. | Transarterial chemoembolization in unresectable hepatocellular carcinoma with portal vein thrombosis: a perspective on survival | 2014 | Nov | Oman Med J | 29 | 6 | 430-6 | 10.5001/omj.2014.114 | Wrong patient population |
| 49 | Akinwande, O.; Philips, P.; Scoggins, C.; Martin, R. C. | Radioembolization Versus Chemoembolization (DEBDOX) for the Treatment of Unresectable Hepatocellular Carcinoma: A Propensity Matched Study | 2016 | Jan | Anticancer Res | 36 | 1 | 239-46 |  |  |
| 50 | Aktas, G.; Kus, T.; Emin Kalender, M.; Kervancioglu, S.; Sevinc, A.; Kul, S.; Camci, C. | Sorafenib with TACE improves the survival of hepatocellular carcinoma patients with more than 10 cm tumor: a single-center retrospective study | 2017 | Jan-Feb | J buon | 22 | 1 | 150-156 |  | Wrong patient population |
| 51 | Albarrak, J.; Al-Shamsi, H. | Current Status of Management of Hepatocellular Carcinoma in The Gulf Region: Challenges and Recommendations | 2023 | 28-Mar | Cancers (Basel) | 15 | 7 |  | 10.3390/cancers15072001 |  |
| 52 | Aliberti, C.; Carandina, R.; Sarti, D.; Mulazzani, L.; Pizzirani, E.; Guadagni, S.; Fiorentini, G. | Chemoembolization Adopting Polyethylene Glycol Drug-Eluting Embolics Loaded With Doxorubicin for the Treatment of Hepatocellular Carcinoma | 2017 | Aug | AJR Am J Roentgenol | 209 | 2 | 430-434 | 10.2214/ajr.16.17477 | Wrong patient population |
| 53 | Alkhatib, A.; Gomaa, A.; Allam, N.; Rewisha, E.; Waked, I. | Real Life Treatment of Hepatocellular Carcinoma: Impact of Deviation from Guidelines for Recommended Therapy | 2015 |  | Asian Pac J Cancer Prev | 16 | 16 | 6929-34 | 10.7314/apjcp.2015.16.16.6929 |  |
| 54 | Alregib, A. H.; Tan, H. Y.; Wong, Y. H.; Kasbollah, A.; Wong, E. H.; Abdullah, B. J. J.; Perkins, A. C.; Yeong, C. H. | Development and physicochemical characterization of a biodegradable microspheres formulation loaded with samarium-153 and doxorubicin for chemo-radioembolization of liver tumours | 2023 | Aug | J Labelled Comp Radiopharm | 66 | 10 | 308-320 | 10.1002/jlcr.4046 |  |
| 55 | Alsina, A. E.; Nakshabandi, A.; Makris, A. M.; Torres, E. A. | Liver transplantation for hepatocellular carcinoma in Puerto Ricans: underutilization of a curative therapy | 2014 | Dec | P R Health Sci J | 33 | 4 | 170-6 |  |  |
| 56 | Amini, A.; Gamblin, T. C. | Palliation: treating patients with inoperable biliary tract and primary liver tumors | 2014 | Apr | Surg Oncol Clin N Am | 23 | 2 | 383-97 | 10.1016/j.soc.2013.10.008 |  |
| 57 | Amioka, K.; Kawaoka, T.; Kinami, T.; Yamasaki, S.; Kosaka, M.; Johira, Y.; Yano, S.; Naruto, K.; Ando, Y.; Fujii, Y.; Uchikawa, S.; Ono, A.; Yamauchi, M.; Imamura, M.; Kosaka, Y.; Ohya, K.; Mori, N.; Takaki, S.; Tsuji, K.; Masaki, K.; Honda, Y.; Kouno, H.; Kohno, H.; Morio, K.; Moriya, T.; Naeshiro, N.; Nonaka, M.; Aisaka, Y.; Azakami, T.; Hiramatsu, A.; Aikata, H.; Oka, S. | Analysis of Lenvatinib's Efficacy against Intermediate-Stage Unresectable Hepatocellular Carcinoma | 2022 | 16-Oct | Cancers (Basel) | 14 | 20 |  | 10.3390/cancers14205066 |  |
| 58 | Amoyav, B.; Bloom, A. I.; Goldstein, Y.; Miller, R.; Sharam, M.; Fluksman, A.; Benny, O. | Drug-Eluting Porous Embolic Microspheres for Trans-Arterial Delivery of Dual Synergistic Anticancer Therapy for the Treatment of Liver Cancer | 2023 | Dec | Adv Healthc Mater | 12 | 30 | e2301548 | 10.1002/adhm.202301548 |  |
| 59 | Ananchuensook, P.; Sriphoosanaphan, S.; Suksawatamnauy, S.; Siripon, N.; Pinjaroen, N.; Geratikornsupuk, N.; Kerr, S. J.; Thanapirom, K.; Komolmit, P. | Validation and prognostic value of EZ-ALBI score in patients with intermediate-stage hepatocellular carcinoma treated with trans-arterial chemoembolization | 2022 | 14-Jun | BMC Gastroenterol | 22 | 1 | 295 | 10.1186/s12876-022-02366-y |  |
| 60 | Anand, S.; Pottakkat, B.; Raja, K.; Chandrasekar, S.; Satheesh, S. | Transarterial chemoembolization in patients with hepatocellular carcinoma beyond Barcelona-Clinic Liver Cancer- B and portal vein tumor thrombosis: Experience from a tertiary care center | 2021 | 27-Jan | Indian J Cancer | |  |  | 10.4103/ijc.IJC_769_19 |  |
| 61 | Anand, S.; Pottakkat, B.; Raja, K.; Chandrasekar, S.; Satheesh, S. | Transarterial chemoembolization in patients with hepatocellular carcinoma beyond Barcelona-Clinic Liver Cancer- B and portal vein tumor thrombosis: Experience from a tertiary care center | 2022 | Jul-Sep | Indian J Cancer | 59 | 3 | 325-329 | 10.4103/ijc.IJC_769_19 |  |
| 62 | And Biomechanics, A. B. | Retracted: Prognostic Comparison between cTACE and H101-TACE in Unresectable Hepatocellular Carcinoma (HCC): A Propensity-Score Matching Analysis | 2023 |  | Appl Bionics Biomech | 2023 |  | 9813798 | 10.1155/2023/9813798 |  |
| 63 | Andreozzi, G.; Lorenzoni, V.; Bargellini, I.; Cioni, R.; Turchetti, G. | Drug-eluting Microspheres Compared to Conventional Transarterial Chemoembolization as First Line Treatment for Unresectable Hepatocellular Carcinoma: A Single-center Retrospective Cost-utility Analysis | 2023 | Mar | Cardiovasc Intervent Radiol | 46 | 3 | 319-326 | 10.1007/s00270-022-03335-4 |  |
| 64 | Aoe, M.; Kanemitsu, T.; Ohki, T.; Kishi, S.; Ogura, Y.; Takenaka, Y.; Hashiba, T.; Ambe, H.; Furukawa, E.; Kurata, Y.; Ichikawa, M.; Ohara, K.; Honda, T.; Furuse, S.; Saito, K.; Toda, N.; Mise, N. | Incidence and risk factors of contrast-induced nephropathy after transcatheter arterial chemoembolization in hepatocellular carcinoma | 2019 | Sep | Clin Exp Nephrol | 23 | 9 | 1141-1146 | 10.1007/s10157-019-01751-4 |  |
| 65 | Aramaki, O.; Takayama, T.; Moriguchi, M.; Sakamoto, H.; Yodono, H.; Kokudo, N.; Yamanaka, N.; Kawasaki, S.; Sasaki, Y.; Kubota, K.; Otsuji, E.; Tanaka, S.; Matsuyama, Y.; Fujii, M. | Arterial chemoembolisation with cisplatin versus epirubicin for hepatocellular carcinoma (ACE 500 study): A multicentre, randomised controlled phase 2/3 trial | 2021 | Nov | Eur J Cancer | 157 |  | 373-382 | 10.1016/j.ejca.2021.08.027 | Wrong patient population |
| 66 | Arita, J.; Ichida, A.; Nagata, R.; Mihara, Y.; Kawaguchi, Y.; Ishizawa, T.; Akamatsu, N.; Kaneko, J.; Hasegawa, K. | Conversion surgery after preoperative therapy for advanced hepatocellular carcinoma in the era of molecular targeted therapy and immune checkpoint inhibitors | 2022 | Jul | J Hepatobiliary Pancreat Sci | 29 | 7 | 732-740 | 10.1002/jhbp.1135 |  |
| 67 | Arslan, M.; Degirmencioglu, S. | Risk Factors for Postembolization Syndrome After Transcatheter Arterial Chemoembolization | 2019 |  | Curr Med Imaging Rev | 15 | 4 | 380-385 | 10.2174/1573405615666181122145330 |  |
| 68 | Ashoori, N.; Paprottka, P.; Trumm, C.; Bamberg, F.; Kolligs, F. T.; Rentsch, M.; Reiser, M. F.; Jakobs, T. F. | Multimodality treatment with conventional transcatheter arterial chemoembolization and radiofrequency ablation for unresectable hepatocellular carcinoma | 2012 |  | Digestion | 85 | 1 | 18-26 | 10.1159/000334714 | Wrong patient population |
| 69 | Atay, M.; Ozdemir, H. | An Unusual Complication of Transarterial Chemoembolization of Hepatocellular Carcinoma; Pseudoaneurysm: A Case Report | 2022 |  | Curr Med Imaging | 18 | 11 | 1244-1247 | 10.2174/1573405618666220325101911 |  |
| 70 | Attia, N. M.; Othman, M. H. M. | Transcatheter arterial chemoembolization of hepatocellular carcinoma in patients with celiac axis occlusion using pancreaticoduodenal arcade as a challenging alternative route | 2017 |  | Eur J Radiol Open | 4 |  | 53-57 | 10.1016/j.ejro.2017.04.002 |  |
| 71 | Auer, T. A.; Sofue, K.; Ueshima, E.; Rauer, N.; Yamaguchi, T.; Gebauer, B.; Hamm, B.; Murakami, T.; Althoff, C. E. | Transarterial Chemoebolization in Hepatocellular Carcinoma: A Binational Japanese-German Study | 2022 |  | Journal of hepatocellular carcinoma | | | 695-705 |  |  |
| 72 | Ayyub, J.; Dabhi, K. N.; Gohil, N. V.; Tanveer, N.; Hussein, S.; Pingili, S.; Makkena, V. K.; Jaramillo, A. P.; Awosusi, B. L.; Nath, T. S. | Evaluation of the Safety and Efficacy of Conventional Transarterial Chemoembolization (cTACE) and Drug-Eluting Bead (DEB)-TACE in the Management of Unresectable Hepatocellular Carcinoma: A Systematic Review | 2023 | Jul | Cureus | 15 | 7 | e41943 | 10.7759/cureus.41943 | Wrong study design |
| 73 | Bai, J.; Huang, M.; Song, B.; Luo, W.; Ding, R. | The Current Status and Future Prospects for Conversion Therapy in the Treatment of Hepatocellular Carcinoma | 2023 | Jan-Dec | Technol Cancer Res Treat | 22 |  | 1.53E+16 | 10.1177/15330338231159718 |  |
| 74 | Bai, W.; Wang, Y. J.; Zhao, Y.; Qi, X. S.; Yin, Z. X.; He, C. Y.; Li, R. J.; Wu, K. C.; Xia, J. L.; Fan, D. M.; Han, G. H. | Sorafenib in combination with transarterial chemoembolization improves the survival of patients with unresectable hepatocellular carcinoma: a propensity score matching study | 2013 | Apr | J Dig Dis | 14 | 4 | 181-90 | 10.1111/1751-2980.12038 |  |
| 75 | Bannangkoon, K.; Hongsakul, K.; Tubtawee, T.; Janjindamai, P.; Akkakrisee, S.; Piratvisuth, T.; Geater, A. | Decision-Making Scoring System for the Repetition of Conventional Transarterial Chemoembolization in Patients With Inoperable Hepatocellular Carcinoma | 2022 | 1-Jul | Clin Transl Gastroenterol | 13 | 7 | e00506 | 10.14309/ctg.0000000000000506 |  |
| 76 | Bannerman, D.; Wan, W. | Multifunctional microbeads for drug delivery in TACE | 2016 | Sep | Expert Opin Drug Deliv | 13 | 9 | 1289-300 | 10.1080/17425247.2016.1192122 |  |
| 77 | Bargellini, I.; Lorenzoni, V.; Lorenzoni, G.; Scalise, P.; Andreozzi, G.; Bozzi, E.; Giorgi, L.; Cervelli, R.; Scandiffio, R.; Perrone, O.; Meccia, D. V.; Boccuzzi, A.; Daviddi, F.; Cicorelli, A.; Lunardi, A.; Crocetti, L.; Turchetti, G.; Cioni, R. | Duration of response after DEB-TACE compared to lipiodol-TACE in HCC-naïve patients: a propensity score matching analysis | 2021 | Oct | Eur Radiol | 31 | 10 | 7512-7522 | 10.1007/s00330-021-07905-x | Wrong patient population |
| 78 | Baterdene, O.; Miura, K.; Ueno, W.; Watanabe, S.; Tsukui, M.; Nomoto, H.; Goka, R.; Maeda, H.; Yamamoto, H.; Morimoto, N. | A successful case of transarterial chemoembolization for hyperprogressive disease induced by immunotherapy in a patient with unresectable hepatocellular carcinoma | 2022 | Dec | Clin J Gastroenterol | 15 | 6 | 1101-1107 | 10.1007/s12328-022-01697-3 |  |
| 79 | Bazine, A.; Fetohi, M.; Berri, M. A.; Essaadi, I.; Elbakraoui, K.; Ichou, M.; Errihani, H. | Spinal cord ischemia secondary to transcatheter arterial chemoembolization for hepatocellular carcinoma | 2014 | Sep | Case Rep Gastroenterol | 8 | 3 | 264-9 | 10.1159/000368075 |  |
| 80 | Becker, L. S.; Maschke, S. K.; Dewald, C. L. A.; Meine, T. C.; Winther, H. B. M.; Kirstein, M. M.; Kloeckner, R.; Meyer, B. C.; Wacker, F.; Hinrichs, J. B. | Two-dimensional parametric parenchymal blood flow in transarterial chemoembolisation for hepatocellular carcinoma: perfusion change quantification and tumour response prediction at 3 months post-intervention | 2021 | Feb | Clin Radiol | 76 | 2 | 160.e27-160.e33 | 10.1016/j.crad.2020.09.012 |  |
| 81 | Beheshti, M. V.; Meek, J. | Calculation of operating expenses for conventional transarterial chemoembolization in an academic medical center: a step toward defining the value of transarterial chemoembolization | 2014 | Apr | J Vasc Interv Radiol | 25 | 4 | 567-74 | 10.1016/j.jvir.2013.10.023 |  |
| 82 | Ben Khaled, N.; Seidensticker, M.; Ricke, J.; Mayerle, J.; Oehrle, B.; Rössler, D.; Teupser, D.; Ehmer, U.; Bitzer, M.; Waldschmidt, D.; Fuchs, M.; Reuken, P. A.; Lange, C. M.; Wege, H.; Kandulski, A.; Dechêne, A.; Venerito, M.; Berres, M. L.; Luedde, T.; Kubisch, I.; Reiter, F. P.; De Toni, E. N. | Atezolizumab and bevacizumab with transarterial chemoembolization in hepatocellular carcinoma: the DEMAND trial protocol | 2022 | Apr | Future Oncol | 18 | 12 | 1423-1435 | 10.2217/fon-2021-1261 |  |
| 83 | Bharadwaz, A.; Bak-Fredslund, K. P.; Villadsen, G. E.; Nielsen, J. E.; Simonsen, K.; Sandahl, T. D.; Grønbæk, H.; Nielsen, D. T. | Combination of radiofrequency ablation with transarterial chemoembolization for treatment of hepatocellular carcinoma: experience from a Danish tertiary liver center | 2016 | Jul | Acta Radiol | 57 | 7 | 844-51 | 10.1177/0284185115603246 | Wrong patient population |
| 84 | Bi, Y.; Jiao, D.; Ren, J.; Han, X. | Clinical Outcomes of Drug-Eluting Bead Transarterial Chemoembolization Loaded with Raltitrexed for the Treatment of Unresectable or Recurrent Hepatocellular Carcinoma | 2022 |  | Can J Gastroenterol Hepatol | 2022 |  | 2602121 | 10.1155/2022/2602121 | Wrong outcomes |
| 85 | Bi, Y.; Jiao, D.; Wang, Y.; Han, X.; Ren, J. | Preliminary outcomes of raltitrexed eluting bead-transarterial chemoembolization using Callispheres® beads for gastrointestinal adenocarcinoma liver metastasis | 2022 | 12-Jul | World J Surg Oncol | 20 | 1 | 229 | 10.1186/s12957-022-02696-x |  |
| 86 | Bi, Y.; Li, F.; Ren, J.; Han, X. | The safety and efficacy of oxaliplatin-loaded drug-eluting beads transarterial chemoembolization for the treatment of unresectable or advanced lung cancer | 2022 |  | Front Pharmacol | 13 |  | 1079707 | 10.3389/fphar.2022.1079707 |  |
| 87 | Bi, Y.; Ren, J.; Han, X. | Clinical Outcomes of Drug-eluting Beads Transarterial Chemoembolization for Unresectable Gastric Carcinoma | 2023 | Nov | J Gastrointest Surg | 27 | 11 | 2577-2579 | 10.1007/s11605-023-05738-1 |  |
| 88 | Bi, Y.; Ren, K.; Ren, J.; Ma, J.; Han, X. | Oxaliplatin Eluting CalliSpheres Microspheres for the Treatment of Unresectable or Recurrent Hepatocellular Carcinoma | 2022 |  | Front Pharmacol | 13 |  | 923585 | 10.3389/fphar.2022.923585 | Wrong patient population |
| 89 | Bi, Y.; Shi, X.; Ren, J.; Yi, M.; Han, X. | Transarterial chemoembolization of unresectable renal cell carcinoma with doxorubicin-loaded CalliSpheres drug-eluting beads | 2022 | 17-May | Sci Rep | 12 | 1 | 8136 | 10.1038/s41598-022-12334-x |  |
| 90 | Bi, Y.; Shi, X.; Ren, J.; Yi, M.; Han, X.; Song, M. | Clinical outcomes of doxorubicin-eluting CalliSpheres® beads-transarterial chemoembolization for unresectable or recurrent esophageal carcinoma | 2021 | 21-May | BMC Gastroenterol | 21 | 1 | 231 | 10.1186/s12876-021-01816-3 |  |
| 91 | Bi, Y.; Wang, Y.; Zhang, W.; Lu, H.; Ren, J.; Han, X. | Preliminary outcomes of DEB-TACE loaded with raltitrexed in the treatment of unresectable or recurrent hepatocellular carcinoma | 2023 | 22-Feb | Cancer Imaging | 23 | 1 | 19 | 10.1186/s40644-023-00534-1 | Wrong patient population |
| 92 | Bian, L. F.; Zhao, X. H.; Gao, B. L.; Zhang, S.; Ge, G. M.; Zhan, D. D.; Ye, T. T.; Zheng, Y. | Predictive model for acute abdominal pain after transarterial chemoembolization for liver cancer | 2020 | 14-Aug | World J Gastroenterol | 26 | 30 | 4442-4452 | 10.3748/wjg.v26.i30.4442 |  |
| 93 | Biederman, D. M.; Titano, J. J.; Bishay, V. L.; Durrani, R. J.; Dayan, E.; Tabori, N.; Patel, R. S.; Nowakowski, F. S.; Fischman, A. M.; Kim, E. | Radiation Segmentectomy versus TACE Combined with Microwave Ablation for Unresectable Solitary Hepatocellular Carcinoma Up to 3 cm: A Propensity Score Matching Study | 2017 | Jun | Radiology | 283 | 3 | 895-905 | 10.1148/radiol.2016160718 |  |
| 94 | Biederman, D. M.; Titano, J. J.; Korff, R. A.; Fischman, A. M.; Patel, R. S.; Nowakowski, F. S.; Lookstein, R. A.; Kim, E. | Radiation Segmentectomy versus Selective Chemoembolization in the Treatment of Early-Stage Hepatocellular Carcinoma | 2018 | Jan | J Vasc Interv Radiol | 29 | 1 | 30-37.e2 | 10.1016/j.jvir.2017.08.026 |  |
| 95 | Binzaqr, S.; Debordeaux, F.; Blanc, J. F.; Papadopoulos, P.; Hindie, E.; Lapouyade, B.; Pinaquy, J. B. | Efficacy of Selective Internal Radiation Therapy for Hepatocellular Carcinoma Post-Incomplete Response to Chemoembolization | 2023 | 1-Dec | Pharmaceuticals (Basel) | 16 | 12 |  | 10.3390/ph16121676 |  |
| 96 | Boldanova, T.; Fucile, G.; Vosshenrich, J.; Suslov, A.; Ercan, C.; Coto-Llerena, M.; Terracciano, L. M.; Zech, C. J.; Boll, D. T.; Wieland, S.; Heim, M. H. | Supervised learning based on tumor imaging and biopsy transcriptomics predicts response of hepatocellular carcinoma to transarterial chemoembolization | 2021 | 16-Nov | Cell Rep Med | 2 | 11 | 100444 | 10.1016/j.xcrm.2021.100444 |  |
| 97 | Bonekamp, S.; Halappa, V. G.; Geschwind, J. F.; Li, Z.; Corona-Villalobos, C. P.; Reyes, D.; Bhagat, N.; Cosgrove, D. P.; Pawlik, T. M.; Mezey, E.; Eng, J.; Kamel, I. R. | Unresectable hepatocellular carcinoma: MR imaging after intraarterial therapy. Part II. Response stratification using volumetric functional criteria after intraarterial therapy | 2013 | Aug | Radiology | 268 | 2 | 431-9 | 10.1148/radiol.13121637 |  |
| 98 | Bonekamp, S.; Li, Z.; Geschwind, J. F.; Halappa, V. G.; Corona-Villalobos, C. P.; Reyes, D.; Pawlik, T. M.; Bonekamp, D.; Eng, J.; Kamel, I. R. | Unresectable hepatocellular carcinoma: MR imaging after intraarterial therapy. Part I. Identification and validation of volumetric functional response criteria | 2013 | Aug | Radiology | 268 | 2 | 420-30 | 10.1148/radiol.13122307 |  |
| 99 | Borrego Rivas, S.; Martín Izquierdo, A.; Quiñones Castro, R.; Onecha Vallejo, V.; Alonso Fernández, N.; Alcoba Vega, L.; González Puente, I.; Jorquera Plaza, F. | Retirform purpura, a rare chemoembolization adverse outcome | 2023 | 30-Nov | Rev Esp Enferm Dig | |  |  | 10.17235/reed.2023.10063/2023 |  |
| 100 | Bossé, D.; Ng, T.; Ahmad, C.; Alfakeeh, A.; Alruzug, I.; Biagi, J.; Brierley, J.; Chaudhury, P.; Cleary, S.; Colwell, B.; Cripps, C.; Dawson, L. A.; Dorreen, M.; Ferland, E.; Galiatsatos, P.; Girard, S.; Gray, S.; Halwani, F.; Kopek, N.; Mahmud, A.; Martel, G.; Robillard, L.; Samson, B.; Seal, M.; Siddiqui, J.; Sideris, L.; Snow, S.; Thirwell, M.; Vickers, M.; Goodwin, R.; Goel, R.; Hsu, T.; Tsvetkova, E.; Ward, B.; Asmis, T. | Eastern Canadian Gastrointestinal Cancer Consensus Conference 2016 | 2016 | Dec | Curr Oncol | 23 | 6 | e605-e614 | 10.3747/co.23.3394 |  |
| 101 | Boulin, M.; Adam, H.; Guiu, B.; Aho, L. S.; Cercueil, J. P.; Di Martino, C.; Fagnoni, P.; Minello, A.; Jouve, J. L.; Hillon, P.; Bedenne, L.; Lepage, C. | Predictive factors of transarterial chemoembolisation toxicity in unresectable hepatocellular carcinoma | 2014 | Apr | Dig Liver Dis | 46 | 4 | 358-62 | 10.1016/j.dld.2013.12.012 | Wrong patient population |
| 102 | Boulin, M.; Schmitt, A.; Delhom, E.; Cercueil, J. P.; Wendremaire, M.; Imbs, D. C.; Fohlen, A.; Panaro, F.; Herrero, A.; Denys, A.; Guiu, B. | Improved stability of lipiodol-drug emulsion for transarterial chemoembolisation of hepatocellular carcinoma results in improved pharmacokinetic profile: Proof of concept using idarubicin | 2016 | Feb | Eur Radiol | 26 | 2 | 601-9 | 10.1007/s00330-015-3855-4 |  |
| 103 | Breitbach, C. J.; Moon, A.; Burke, J.; Hwang, T. H.; Kirn, D. H. | A Phase 2, Open-Label, Randomized Study of Pexa-Vec (JX-594) Administered by Intratumoral Injection in Patients with Unresectable Primary Hepatocellular Carcinoma | 2015 |  | Methods Mol Biol | 1317 |  | 343-57 | 10.1007/978-1-4939-2727-2_19 |  |
| 104 | Brennan, I. M.; Ahmed, M. | Imaging features following transarterial chemoembolization and radiofrequency ablation of hepatocellular carcinoma | 2013 | Aug | Semin Ultrasound CT MR | 34 | 4 | 336-51 | 10.1053/j.sult.2013.04.004 |  |
| 105 | Bressler, L.; Bath, N.; Manne, A.; Miller, E.; Cloyd, J. M. | Management of locally advanced intrahepatic cholangiocarcinoma: a narrative review | 2023 | Apr | Chin Clin Oncol | 12 | 2 | 15 | 10.21037/cco-22-115 |  |
| 106 | Buckstein, M.; Kim, E.; Özbek, U.; Tabrizian, P.; Gunasekaran, G.; Facciuto, M.; Rosenzweig, K.; Llovet, J. M.; Schwartz, M. | Combination Transarterial Chemoembolization and Stereotactic Body Radiation Therapy for Unresectable Single Large Hepatocellular Carcinoma: Results From a Prospective Phase 2 Trial | 2022 | 1-Oct | Int J Radiat Oncol Biol Phys | 114 | 2 | 221-230 | 10.1016/j.ijrobp.2022.05.021 |  |
| 107 | Buckstein, M.; Kim, E.; Fischman, A.; Blacksburg, S.; Facciuto, M.; Schwartz, M.; Rosenzweig, K. | Stereotactic body radiation therapy following transarterial chemoembolization for unresectable hepatocellular carcinoma | 2018 | Aug | J Gastrointest Oncol | 9 | 4 | 734-740 | 10.21037/jgo.2018.05.01 |  |
| 108 | Buijs, M.; Reyes, D. K.; Pawlik, T. M.; Blackford, A. L.; Salem, R.; Messersmith, W. A.; Weekes, C. D.; Mulcahy, M.; Kamel, I. R.; Geschwind, J. F. | Phase 2 trial of concurrent bevacizumab and transhepatic arterial chemoembolization in patients with unresectable hepatocellular carcinoma | 2013 | 1-Mar | Cancer | 119 | 5 | 1042-9 | 10.1002/cncr.27859 |  |
| 109 | Burrel, Marta; Reig, Mara; Forner, Alejandro; Barrufet, Marta; de Lope, Carlos Rodrguez; Tremosini, Silvia; Ayuso, Carmen; Llovet, Josep M; Real, Mara Isabel; Bruix, Jordi | Survival of patients with hepatocellular carcinoma treated by transarterial chemoembolisation (TACE) using Drug Eluting Beads. Implications for clinical practice and trial design | 2012 |  | Journal of hepatology | 56 | 6 | 1330-1335 |  |  |
| 110 | Butcher, Daniel A; Brandis, Kelli J; Wang, Haolu; Spannenburg, Liam; Bridle, Kim R; Crawford, Darrell HG; Liang, Xiaowen | Long-term survival and postoperative complications of pre-liver transplantation transarterial chemoembolisation in hepatocellular carcinoma: a systematic review and meta-analysis | 2022 |  | European Journal of Surgical Oncology | 48 | 3 | 621-631 |  | Wrong publication type |
| 111 | Bzeizi, K. I.; Arabi, M.; Jamshidi, N.; Albenmousa, A.; Sanai, F. M.; Al-Hamoudi, W.; Alghamdi, S.; Broering, D.; Alqahtani, S. A. | Conventional Transarterial Chemoembolization Versus Drug-Eluting Beads in Patients with Hepatocellular Carcinoma: A Systematic Review and Meta-Analysis | 2021 | 7-Dec | Cancers (Basel) | 13 | 24 |  | 10.3390/cancers13246172 | Wrong patient population |
| 112 | Cai, L.; Li, H.; Guo, J.; Zhao, W.; Duan, Y.; Hou, X.; Cheng, L.; Du, H.; Shao, X.; Diao, Z.; Li, C. | Drug-eluting bead transarterial chemoembolization is an effective downstaging option for subsequent radical treatments in patients with hepatocellular carcinoma: A cohort study | 2021 | Jul | Clin Res Hepatol Gastroenterol | 45 | 4 | 101535 | 10.1016/j.clinre.2020.09.002 | Wrong patient population |
| 113 | Cao, F.; Yang, Y.; Si, T.; Luo, J.; Zeng, H.; Zhang, Z.; Feng, D.; Chen, Y.; Zheng, J. | The Efficacy of TACE Combined With Lenvatinib Plus Sintilimab in Unresectable Hepatocellular Carcinoma: A Multicenter Retrospective Study | 2021 |  | Front Oncol | 11 |  | 783480 | 10.3389/fonc.2021.783480 | Wrong patient population |
| 114 | Cao, G.; Zhu, X.; Li, J.; Shen, L.; Yang, R.; Chen, H.; Wang, X.; Gao, S.; Xu, H.; Zhu, L.; Liu, P.; Guo, J. | A comparative study between Embosphere(®) and conventional transcatheter arterial chemoembolization for treatment of unresectable liver metastasis from GIST | 2014 | Feb | Chin J Cancer Res | 26 | 1 | 124-31 | 10.3978/j.issn.1000-9604.2014.02.11 |  |
| 115 | Cao, Y.; Ouyang, T.; Xiong, F.; Kan, X.; Chen, L.; Liang, B.; Zheng, C. | Efficacy of apatinib in patients with sorafenib-transarterial chemoembolization refractory hepatocellular carcinoma: a retrospective study | 2021 | Oct | Hepatol Int | 15 | 5 | 1268-1277 | 10.1007/s12072-021-10198-3 | Wrong patient population |
| 116 | Cappelli, A.; Cucchetti, A.; Cabibbo, G.; Mosconi, C.; Maida, M.; Attardo, S.; Pettinari, I.; Pinna, A. D.; Golfieri, R. | Refining prognosis after trans-arterial chemo-embolization for hepatocellular carcinoma | 2016 | May | Liver Int | 36 | 5 | 729-36 | 10.1111/liv.13029 |  |
| 117 | Carvalho, V. O.; Galastri, F. L.; Affonso, B. B.; Falsarella, P. M.; Valle, L. G. M.; Ferraz-Neto, B. H.; Rezende, M. B.; Motta-Leal-Filho, J. M. D.; Garcia, R. G.; Nasser, F. | Transarterial radioembolization for liver tumors as neoadjuvant therapy: three case reports | 2020 |  | Einstein (Sao Paulo) | 18 |  | eRC4990 | 10.31744/einstein_journal/2020RC4990 |  |
| 118 | Casadei Gardini, A.; Santini, D.; Aprile, G.; Silvestris, N.; Felli, E.; Foschi, F. G.; Ercolani, G.; Marisi, G.; Valgiusti, M.; Passardi, A.; Puzzoni, M.; Silletta, M.; Brunetti, O.; Cardellino, G. G.; Frassineti, G. L.; Scartozzi, M. | Antiangiogenic agents after first line and sorafenib plus chemoembolization: a systematic review | 2017 | 12-Sep | Oncotarget | 8 | 39 | 66699-66708 | 10.18632/oncotarget.19449 |  |
| 119 | Casadei Gardini, A.; Tamburini, E.; Iñarrairaegui, M.; Frassineti, G. L.; Sangro, B. | Radioembolization versus chemoembolization for unresectable hepatocellular carcinoma: a meta-analysis of randomized trials | 2018 |  | Onco Targets Ther | 11 |  | 7315-7321 | 10.2147/ott.S175715 | Wrong patient population |
| 120 | Casadei-Gardini, A.; Scartozzi, M.; Tada, T.; Yoo, C.; Shimose, S.; Masi, G.; Lonardi, S.; Frassineti, L. G.; Nicola, S.; Piscaglia, F.; Kumada, T.; Kim, H. D.; Koga, H.; Vivaldi, C.; Soldà, C.; Hiraoka, A.; Bang, Y.; Atsukawa, M.; Torimura, T.; Tsuj, K.; Itobayashi, E.; Toyoda, H.; Fukunishi, S.; Rimassa, L.; Rimini, M.; Cascinu, S.; Cucchetti, A. | Lenvatinib versus sorafenib in first-line treatment of unresectable hepatocellular carcinoma: An inverse probability of treatment weighting analysis | 2021 | Jun | Liver Int | 41 | 6 | 1389-1397 | 10.1111/liv.14817 |  |
| 121 | Cascella, T.; Garanzini, E. M.; Lanocita, R.; Morosi, C.; Riva, F.; Greco, G.; Sposito, C.; Mazzaferro, V.; Marchian, A.; Spreafico, C. | Long Term Survival Analysis in a Cohort of 125 Patients with Hepatocellular Carcinoma Treated with Transarterial Chemoembolization Using Small Drug Eluting Beads | 2022 | Jan | Cardiovasc Intervent Radiol | 45 | 1 | 54-61 | 10.1007/s00270-021-02991-2 | Wrong outcomes |
| 122 | Cascella, T.; Garanzini, E. M.; Lanocita, R.; Morosi, C.; Riva, F.; Greco, G.; Sposito, C.; Mazzaferro, V.; Marchianò, A.; Spreafico, C. | Long Term Survival Analysis in a Cohort of 125 Patients with Hepatocellular Carcinoma Treated with Transarterial Chemoembolization Using Small Drug Eluting Beads | 2022 | Jan | Cardiovasc Intervent Radiol | 45 | 1 | 54-61 | 10.1007/s00270-021-02991-2 | Wrong outcomes |
| 123 | Celsa, C.; Cabibbo, G.; Enea, M.; Battaglia, S.; Rizzo, G. E. M.; Busacca, A.; Giuffrida, P.; Stornello, C.; Brancatelli, G.; Cannella, R.; Gruttadauria, S.; Cammà, C. | Are radiological endpoints surrogate outcomes of overall survival in hepatocellular carcinoma treated with transarterial chemoembolization? | 2021 | May | Liver Int | 41 | 5 | 1105-1116 | 10.1111/liv.14822 | Wrong patient population |
| 124 | Cerreto, M.; Cardone, F.; Cerrito, L.; Stella, L.; Santopaolo, F.; Pallozzi, M.; Gasbarrini, A.; Ponziani, F. R. | The New Era of Systemic Treatment for Hepatocellular Carcinoma: From the First Line to the Optimal Sequence | 2023 | 26-Sep | Curr Oncol | 30 | 10 | 8774-8792 | 10.3390/curroncol30100633 |  |
| 125 | Chan, K. M.; Lai, Y.; Hung, H. C.; Lee, J. C.; Cheng, C. H.; Wang, Y. C.; Wu, T. H.; Lee, C. F.; Wu, T. J.; Chou, H. S.; Wang, C. T.; Chai, P. M.; Lien, H. Y.; Lee, W. C. | Significance of Physical Status and Liver Function Reserve for Outcome of Patients with Advanced Hepatocellular Carcinoma Receiving Lenvatinib Treatment | 2023 |  | J Hepatocell Carcinoma | 10 |  | 281-290 | 10.2147/jhc.S393964 |  |
| 126 | Chan, L. S.; Sze, D. Y.; Poultsides, G. A.; Louie, J. D.; Abdelrazek Mohammed, M. A.; Wang, D. S. | Yttrium-90 Radioembolization for Unresectable Combined Hepatocellular-Cholangiocarcinoma | 2017 | Sep | Cardiovasc Intervent Radiol | 40 | 9 | 1383-1391 | 10.1007/s00270-017-1648-7 |  |
| 127 | Chan, S. C.; Fan, S. T. | Selection of patients of hepatocellular carcinoma beyond the Milan criteria for liver transplantation | 2013 | Apr | Hepatobiliary Surg Nutr | 2 | 2 | 84-8 | 10.3978/j.issn.2304-3881.2012.12.04 |  |
| 128 | Chang, P. Y.; Huang, C. C.; Hung, C. H.; Yu, C. Y.; Wu, D. K.; Hwang, J. I.; Liang, P. C.; Wu, R. H.; Tsai, W. L.; Lin, Y. J.; Liu, Y. S.; Liang, H. L.; Lee, R. C.; Chen, C. H. | Multidisciplinary Taiwan Consensus Recommendations for the Use of DEBDOX-TACE in Hepatocellular Carcinoma Treatment | 2018 | Oct | Liver Cancer | 7 | 4 | 312-322 | 10.1159/000487608 |  |
| 129 | Chang, P. Y.; Lee, R. C.; Liang, P. C.; Liu, Y. S.; Chuang, V. P.; Wu, D. K.; Cheng, Y. F.; Huang, J. I.; Tseng, H. S.; Hung, C. F.; Wu, R. H.; Chern, M. C.; Cheng, H. M.; Wu, C. H.; Cheng, S. M.; Chiang, C. L.; Liang, H. L. | Multidisciplinary Taiwan consensus for the use of conventional TACE in hepatocellular carcinoma treatment | 2023 |  | Front Oncol | 13 |  | 1186674 | 10.3389/fonc.2023.1186674 |  |
| 130 | Chao, J.; Zhu, Q.; Chen, D.; An, X.; Liu, A.; Zhou, F.; Yuan, L.; Wang, Z.; Sun, H. | Case Report: Transarterial Chemoembolization in Combination With Tislelizumab Downstages Unresectable Hepatocellular Carcinoma Followed by Radical Salvage Resection | 2021 |  | Front Oncol | 11 |  | 667555 | 10.3389/fonc.2021.667555 |  |
| 131 | Chao, Y.; Chung, Y. H.; Han, G.; Yoon, J. H.; Yang, J.; Wang, J.; Shao, G. L.; Kim, B. I.; Lee, T. Y. | The combination of transcatheter arterial chemoembolization and sorafenib is well tolerated and effective in Asian patients with hepatocellular carcinoma: final results of the START trial | 2015 | 15-Mar | Int J Cancer | 136 | 6 | 1458-67 | 10.1002/ijc.29126 |  |
| 132 | Chapiro, J.; Duran, R.; Geschwind, J. F. | Combination of intra-arterial therapies and sorafenib: is there a clinical benefit? | 2014 | Jul | Radiol Med | 119 | 7 | 476-82 | 10.1007/s11547-014-0413-0 |  |
| 133 | Chapiro, J.; Duran, R.; Lin, M.; Schernthaner, R. E.; Wang, Z.; Gorodetski, B.; Geschwind, J. F. | Identifying Staging Markers for Hepatocellular Carcinoma before Transarterial Chemoembolization: Comparison of Three-dimensional Quantitative versus Non-three-dimensional Imaging Markers | 2015 | May | Radiology | 275 | 2 | 438-47 | 10.1148/radiol.14141180 |  |
| 134 | Chegai, F.; Merolla, S.; Greco, L.; Nezzo, M.; Mannelli, L.; Orlacchio, A. | Re: Baseline and Early MR Apparent Diffusion Coefficient Quantification as a Predictor of Response of Unresectable Hepatocellular Carcinoma to Doxorubicin Drug-Eluting Bead Chemoembolization | 2016 | Sep | J Vasc Interv Radiol | 27 | 9 | 1456-1458 | 10.1016/j.jvir.2016.05.007 |  |
| 135 | Chen, A.; Li, S.; Yao, Z.; Hu, J.; Cao, J.; Topatana, W.; Juengpanich, S.; Yu, H.; Shen, J.; Chen, M. | Adjuvant transarterial chemoembolization to sorafenib in unresectable hepatocellular carcinoma: A meta-analysis | 2021 | Feb | J Gastroenterol Hepatol | 36 | 2 | 302-310 | 10.1111/jgh.15180 | Wrong patient population |
| 136 | Chen, B. B.; Shih, I. L.; Wu, C. H.; Hsu, C.; Chen, C. H.; Shih, T. T.; Liu, K. L.; Liang, P. C. | Comparison of characteristics and transarterial chemoembolization outcomes in patients with unresectable hepatocellular carcinoma and different viral etiologies | 2014 | Mar | J Vasc Interv Radiol | 25 | 3 | 371-8 | 10.1016/j.jvir.2013.10.027 |  |
| 137 | Chen, B.; Dai, H.; Yang, J.; Zhang, G.; Wen, C.; Xiang, X.; Lin, R.; Huang, Y. | Transarterial Chemoembolization Followed by Hepatic Arterial Infusion Chemotherapy Combined a Tyrosine Kinase Inhibitor for Treatment of Large Hepatocellular Carcinoma | 2023 |  | Curr Cancer Drug Targets | 23 | 7 | 564-571 | 10.2174/1568009623666230215142941 |  |
| 138 | Chen, C. S.; Li, F. K.; Guo, C. Y.; Xiao, J. C.; Hu, H. T.; Cheng, H. T.; Zheng, L.; Zong, D. W.; Ma, J. L.; Jiang, L.; Li, H. L. | Tumor vascularity and lipiodol deposition as early radiological markers for predicting risk of disease progression in patients with unresectable hepatocellular carcinoma after transarterial chemoembolization | 2016 | 9-Feb | Oncotarget | 7 | 6 | 7241-52 | 10.18632/oncotarget.6892 |  |
| 139 | Chen, D.; Wang, R.; Meng, X.; Yan, H.; Jiang, S.; Feng, R.; Zhu, K.; Xu, X.; Dou, X.; Jin, L. | Prognostic value of serum γ-glutamyl transferase in unresectable hepatocellular carcinoma patients treated with transcatheter arterial chemoembolization combined with conformal radiotherapy | 2014 | Nov | Oncol Lett | 8 | 5 | 2298-2304 | 10.3892/ol.2014.2456 |  |
| 140 | Chen, H.; Nan, G.; Wei, D.; Zhai, R. Y.; Huang, M.; Yang, W. W.; Xing, B. C.; Zhu, X.; Xu, H. F.; Wang, X. D.; Zhang, X. Y.; Zhu, B. R.; Liu, P.; Cao, G.; Gao, S.; Hao, C. Y.; Yang, R. J.; Guo, J. H.; Zhang, X.; Gao, K.; Wang, K.; Wang, J. F.; Li, Z. Y.; Zhu, L. Z.; Ding, R.; Li, J.; Zhao, L.; Shao, Y. J.; Liu, H. C.; Xia, J. L.; Wang, L.; Kong, L. M.; Chen, Z. N.; Bian, H. | Hepatic Artery Injection of (131)I-Metuximab Combined with Transcatheter Arterial Chemoembolization for Unresectable Hepatocellular Carcinoma: A Prospective Nonrandomized, Multicenter Clinical Trial | 2022 | Apr | J Nucl Med | 63 | 4 | 556-559 | 10.2967/jnumed.121.262136 | Wrong patient population |
| 141 | Chen, H.; Nan, G.; Wei, D.; Zhai, R. Y.; Huang, M.; Yang, W. W.; Xing, B. C.; Zhu, X.; Xu, H. F.; Wang, X. D.; Zhang, X. Y.; Zhu, B. R.; Liu, P.; Cao, G.; Gao, S.; Hao, C. Y.; Yang, R. J.; Guo, J. H.; Zhang, X.; Gao, K.; Wang, K.; Wang, J. F.; Li, Z. Y.; Zhu, L. Z.; Ding, R.; Li, J.; Zhao, L.; Shao, Y. J.; Liu, H. C.; Xia, J. L.; Wang, L.; Kong, L. M.; Chen, Z. N.; Bian, H. | Hepatic artery injection of (131)I-metuximab combined with transcatheter arterial chemoembolization for unresectable hepatocellular carcinoma: a prospective non-randomized, multicenter clinical trial | 2021 | 2-Sep | J Nucl Med |  |  |  | 10.2967/jnumed.121.262136 |  |
| 142 | Chen, H.; Wang, X.; Zhu, W.; Li, Y.; Yu, Z.; Li, H.; Yang, Y.; Zhu, S.; Chen, X.; Wang, G. | Application of associating liver partition and portal vein ligation for staged hepatectomy for initially unresectable hepatocellular carcinoma | 2022 | 24-Nov | BMC Surg | 22 | 1 | 407 | 10.1186/s12893-022-01848-w | Wrong outcomes |
| 143 | Chen, J.; Zhang, D.; Yuan, Y. | Anti-PD-1/PD-L1 immunotherapy in conversion treatment of locally advanced hepatocellular carcinoma | 2023 | Jul | Clin Exp Med | 23 | 3 | 579-590 | 10.1007/s10238-022-00873-6 |  |
| 144 | Chen, K.; Luo, C. P.; Ge, D. X.; Wang, K. L.; Luo, Q.; Li, Y. Z.; You, X. M.; Xiang, B. D.; Li, L. Q.; Ma, L.; Zhong, J. H. | Case report: Conversion therapy to permit resection of initially unresectable hepatocellular carcinoma | 2022 |  | Front Oncol | 12 |  | 946693 | 10.3389/fonc.2022.946693 |  |
| 145 | Chen, L. C.; Chiou, W. Y.; Lin, H. Y.; Lee, M. S.; Lo, Y. C.; Huang, L. W.; Chang, C. M.; Hung, T. H.; Lin, C. W.; Tseng, K. C.; Liu, D. W.; Hsu, F. C.; Hung, S. K. | Comparing stereotactic ablative radiotherapy (SABR) versus re-trans-catheter arterial chemoembolization (re-TACE) for hepatocellular carcinoma patients who had incomplete response after initial TACE (TASABR): a randomized controlled trial | 2019 | 28-Mar | BMC Cancer | 19 | 1 | 275 | 10.1186/s12885-019-5461-3 |  |
| 146 | Chen, M.; Guo, X.; Shen, L.; Ding, J.; Yu, J.; Chen, X.; Wu, F.; Tu, J.; Zhao, Z.; Nakajima, M.; Song, J.; Shu, G.; Ji, J. | Monodisperse CaCO(3)-loaded gelatin microspheres for reversing lactic acid-induced chemotherapy resistance during TACE treatment | 2023 | 15-Mar | Int J Biol Macromol | 231 |  | 123160 | 10.1016/j.ijbiomac.2023.123160 |  |
| 147 | Chen, M.; Xu, R.; Chen, X.; Mai, Q. | Hepatic fibrosis and short-term clinical efficacy after hepatic artery embolization for unresectable hepatocellular carcinoma using doxorubicin-eluting HepaSphere | 2020 | Mar | Transl Cancer Res | 9 | 3 | 1361-1370 | 10.21037/tcr.2020.01.15 |  |
| 148 | Chen, P.; Yuan, P.; Chen, B.; Sun, J.; Shen, H.; Qian, Y. | Evaluation of drug-eluting beads versus conventional transcatheter arterial chemoembolization in patients with unresectable hepatocellular carcinoma: A systematic review and meta-analysis | 2017 | Feb | Clin Res Hepatol Gastroenterol | 41 | 1 | 75-85 | 10.1016/j.clinre.2016.05.013 | Wrong outcomes |
| 149 | Chen, R.; Kong, W.; Gan, Y.; Ge, N.; Chen, Y.; Ding, H.; Luo, J.; Wang, W.; Ren, Z. | Tumour stiffness associated with tumour response to conventional transarterial chemoembolisation for hepatocellular carcinoma: preliminary findings | 2019 | Oct | Clin Radiol | 74 | 10 | 814.e1-814.e7 | 10.1016/j.crad.2019.07.008 |  |
| 150 | Chen, R.; Li, L.; Li, Y.; Song, K.; Shen, C.; Ma, P.; Wang, Z. | Efficacy and safety of transcatheter arterial chemoembolization-lenvatinib sequential therapy for patients with unresectable hepatocellular carcinoma: a single-arm clinical study | 2022 | Jun | J Gastrointest Oncol | 13 | 3 | 1367-1375 | 10.21037/jgo-22-525 |  |
| 151 | Chen, S. W.; Lin, L. C.; Kuo, Y. C.; Liang, J. A.; Kuo, C. C.; Chiou, J. F. | Phase 2 study of combined sorafenib and radiation therapy in patients with advanced hepatocellular carcinoma | 2014 | 1-Apr | Int J Radiat Oncol Biol Phys | 88 | 5 | 1041-7 | 10.1016/j.ijrobp.2014.01.017 |  |
| 152 | Chen, S.; Cai, H.; Wu, Z.; Tang, S.; Chen, L.; Wang, F.; Zhuang, W.; Guo, W. | Anlotinib combined with transarterial chemoembolization for unresectable hepatocellular carcinoma associated with hepatitis B virus: a retrospective controlled study | 2023 |  | Front Oncol | 13 |  | 1235786 | 10.3389/fonc.2023.1235786 | Wrong patient population |
| 153 | Chen, S.; Wu, Z.; Shi, F.; Mai, Q.; Wang, L.; Wang, F.; Zhuang, W.; Chen, X.; Chen, H.; Xu, B.; Lai, J.; Guo, W. | Lenvatinib plus TACE with or without pembrolizumab for the treatment of initially unresectable hepatocellular carcinoma harbouring PD-L1 expression: a retrospective study | 2021 | 28-Aug | J Cancer Res Clin Oncol | |  |  | 10.1007/s00432-021-03767-4 |  |
| 154 | Chen, S.; Wu, Z.; Shi, F.; Mai, Q.; Wang, L.; Wang, F.; Zhuang, W.; Chen, X.; Chen, H.; Xu, B.; Lai, J.; Guo, W. | Lenvatinib plus TACE with or without pembrolizumab for the treatment of initially unresectable hepatocellular carcinoma harbouring PD-L1 expression: a retrospective study | 2022 | Aug | J Cancer Res Clin Oncol | 148 | 8 | 2115-2125 | 10.1007/s00432-021-03767-4 |  |
| 155 | Chen, S.; Yu, W.; Zhang, K.; Liu, W.; Chen, C. | Comparison of the efficacy and safety of conventional transarterial chemoembolization with and without drug-eluting beads embolization for the treatment of unresectable large hepatocellular carcinoma | 2021 | Apr | Hepatol Res | 51 | 4 | 482-489 | 10.1111/hepr.13620 | Wrong patient population |
| 156 | Chen, S.; Yuan, B.; Yu, W.; Wang, X.; He, C.; Chen, C. | Comparison of Arterial Infusion Chemotherapy and Chemoembolization for Locally Advanced Hepatocellular Carcinoma: a Multicenter Retrospective Study | 2022 | Nov | J Gastrointest Surg | 26 | 11 | 2292-2300 | 10.1007/s11605-022-05421-x |  |
| 157 | Chen, W. J.; Yuan, S. F.; Zhu, L. J.; Sun, X. N.; Zheng, W. | Three-dimensional conformal radiotherapy in combination with transcatheter arterial chemoembolization in the treatment of hepatocellular carcinoma | 2014 | Jul-Sep | J buon | 19 | 3 | 692-7 |  |  |
| 158 | Chen, X. L.; Yu, H. C.; Fan, Q. G.; Yuan, Q.; Jiang, W. K.; Rui, S. Z.; Zhou, W. C. | Comparative effectiveness of interventional therapeutic modalities for unresectable hepatocellular carcinoma: A systematic review and network meta-analysis | 2022 | Oct | Oncol Lett | 24 | 4 | 366 | 10.3892/ol.2022.13486 | Wrong publication type |
| 159 | Chen, X.; Lai, L.; Ye, J.; Li, L. | Downstaging Therapies for Unresectable Hepatocellular Carcinoma Prior to Hepatic Resection: A Systematic Review and Meta-Analysis | 2021 |  | Front Oncol | 11 |  | 740762 | 10.3389/fonc.2021.740762 |  |
| 160 | Chen, Y. C.; Huang, C. W.; Li, C. C.; Chang, T. K.; Su, W. C.; Chen, P. J.; Yeh, Y. S.; Chang, Y. T.; Tsai, H. L.; Shih, M. P.; Wang, J. Y. | Efficacy of transarterial chemoembolization with drug-eluting beads combined with systemic chemotherapy and targeted therapy in colorectal cancer liver metastasis | 2023 | 1-Dec | World J Surg Oncol | 21 | 1 | 378 | 10.1186/s12957-023-03253-w |  |
| 161 | Chen, Yu-Xing; Zhang, Jin-Xing; Zhou, Chun-Gao; Liu, Jin; Liu, Sheng; Shi, Hai-Bin; Zu, Qing-Quan | Comparison of the efficacy and safety of transarterial chemoembolization with or without lenvatinib for unresectable hepatocellular carcinoma: a retrospective propensity score"“matched analysis | 2022 |  | Journal of hepatocellular carcinoma | | | 685-694 |  |  |
| 162 | Chen, Z. H.; Hong, Y. F.; Chen, X.; Chen, J.; Lin, Q.; Lin, J.; Li, X.; Wen, J. Y.; Ruan, D. Y.; Dong, M.; Wei, L.; Wang, T. T.; Lin, Z. X.; Ma, X. K.; Wu, D. H.; Wu, X. Y.; Xu, R. | Comparison of five staging systems in predicting the survival rate of patients with hepatocellular carcinoma undergoing trans-arterial chemoembolization therapy | 2018 | Jan | Oncol Lett | 15 | 1 | 855-862 | 10.3892/ol.2017.7419 |  |
| 163 | Chen, Z.; Fu, X.; Qiu, Z.; Mu, M.; Jiang, W.; Wang, G.; Zhong, Z.; Qi, H.; Gao, F. | CT-guided (125)I brachytherapy for hepatocellular carcinoma in high-risk locations after transarterial chemoembolization combined with microwave ablation: a propensity score-matched study | 2023 | 1-Mar | Radiol Oncol | 57 | 1 | 127-139 | 10.2478/raon-2023-0012 |  |
| 164 | Chen, Z.; Xie, H.; Hu, M.; Huang, T.; Hu, Y.; Sang, N.; Zhao, Y. | Recent progress in treatment of hepatocellular carcinoma | 2020 |  | Am J Cancer Res | 10 | 9 | 2993-3036 |  |  |
| 165 | Cheng, A. L.; Amarapurkar, D.; Chao, Y.; Chen, P. J.; Geschwind, J. F.; Goh, K. L.; Han, K. H.; Kudo, M.; Lee, H. C.; Lee, R. C.; Lesmana, L. A.; Lim, H. Y.; Paik, S. W.; Poon, R. T.; Tan, C. K.; Tanwandee, T.; Teng, G.; Park, J. W. | Re-evaluating transarterial chemoembolization for the treatment of hepatocellular carcinoma: Consensus recommendations and review by an International Expert Panel | 2014 | Feb | Liver Int | 34 | 2 | 174-83 | 10.1111/liv.12314 |  |
| 166 | Cheung, F.; Wang, X.; Wang, N.; Yuen, M. F.; Ziea, T. C.; Tong, Y.; Wong, V. T.; Feng, Y. | Chinese Medicines as an Adjuvant Therapy for Unresectable Hepatocellular Carcinoma during Transarterial Chemoembolization: A Meta-Analysis of Randomized Controlled Trials | 2013 |  | Evid Based Complement Alternat Med | 2013 |  | 487919 | 10.1155/2013/487919 | Wrong patient population |
| 167 | Cheung, T. T.; Poon, R. T.; Jenkins, C. R.; Chu, F. S.; Chok, K. S.; Chan, A. C.; Tsang, S. H.; Dai, W. C.; Yau, T. C.; Chan, S. C.; Fan, S. T.; Lo, C. M. | Survival analysis of high-intensity focused ultrasound therapy vs. transarterial chemoembolization for unresectable hepatocellular carcinomas | 2014 | Jul | Liver Int | 34 | 6 | e136-43 | 10.1111/liv.12474 | Wrong outcomes |
| 168 | Chiang, C. L.; Chan, A. C. Y.; Chiu, K. W. H.; Kong, F. S. | Combined Stereotactic Body Radiotherapy and Checkpoint Inhibition in Unresectable Hepatocellular Carcinoma: A Potential Synergistic Treatment Strategy | 2019 |  | Front Oncol | 9 |  | 1157 | 10.3389/fonc.2019.01157 |  |
| 169 | Chiang, C. L.; Chiu, K. W. H.; Chan, K. S. K.; Lee, F. A. S.; Li, J. C. B.; Wan, C. W. S.; Dai, W. C.; Lam, T. C.; Chen, W.; Wong, N. S. M.; Cheung, A. L. Y.; Lee, V. W. Y.; Lau, V. W. H.; El Helali, A.; Man, K.; Kong, F. M. S.; Lo, C. M.; Chan, A. C. | Sequential transarterial chemoembolisation and stereotactic body radiotherapy followed by immunotherapy as conversion therapy for patients with locally advanced, unresectable hepatocellular carcinoma (START-FIT): a single-arm, phase 2 trial | 2023 | Feb | Lancet Gastroenterol Hepatol | 8 | 2 | 169-178 | 10.1016/s2468-1253(22)00339-9 |  |
| 170 | Chidambaranathan-Reghupaty, S.; Fisher, P. B.; Sarkar, D. | Hepatocellular carcinoma (HCC): Epidemiology, etiology and molecular classification | 2021 |  | Adv Cancer Res | 149 |  | Jan-61 | 10.1016/bs.acr.2020.10.001 |  |
| 171 | Chintalapati, S. P.; Patel, A.; Conjeevaram, H. | Gastric and duodenal ischaemia after transarterial chemoembolisation for hepatocellular carcinoma: an unexpected but significant complication | 2018 | 21-Feb | BMJ Case Rep | 2018 |  |  | 10.1136/bcr-2017-223339 |  |
| 172 | Chiu, S. H.; Chang, P. Y.; Shih, Y. L.; Huang, W. Y.; Ko, K. H.; Chang, W. C.; Huang, G. S. | Efficacy and Safety of Supplemental Transarterial Chemoembolization Through Extrahepatic Collateral Arteries with Drug-eluting Beads: Treatment for Unresectable Hepatocellular Carcinoma | 2020 |  | Drug Des Devel Ther | 14 |  | 5029-5041 | 10.2147/dddt.S266470 | Wrong patient population |
| 173 | Cho, Y. Y.; Yu, S. J.; Yoo, J. J.; Lee, M.; Lee, D. H.; Cho, Y.; Yoon, K. W.; Cho, E. J.; Lee, J. H.; Kim, Y. J.; Yoon, J. H. | The Model to Estimate Survival in Ambulatory Hepatocellular Carcinoma Patients Aids in the Decision for TACE Retreatment | 2020 | Apr | J Clin Gastroenterol | 54 | 4 | 370-377 | 10.1097/mcg.0000000000001148 |  |
| 174 | Cho, Y.; Lee, S.; Park, S. J. | Effectiveness of intraprocedural dual-phase cone-beam computed tomography in detecting hepatocellular carcinoma and improving treatment outcomes following conventional transarterial chemoembolization | 2021 |  | PLoS One | 16 | 1 | e0245911 | 10.1371/journal.pone.0245911 |  |
| 175 | Choi, C.; Koom, W. S.; Kim, T. H.; Yoon, S. M.; Kim, J. H.; Lee, H. S.; Nam, T. K.; Seong, J. | A prospective phase 2 multicenter study for the efficacy of radiation therapy following incomplete transarterial chemoembolization in unresectable hepatocellular carcinoma | 2014 | 1-Dec | Int J Radiat Oncol Biol Phys | 90 | 5 | 1051-60 | 10.1016/j.ijrobp.2014.08.011 |  |
| 176 | Choi, J. W.; Kim, H. C. | Radioembolization for hepatocellular carcinoma: what clinicians need to know | 2022 | Mar | J Liver Cancer | 22 | 1 | 13-Apr | 10.17998/jlc.2022.01.16 |  |
| 177 | Choi, J.; Ryu, J. K.; Lee, S. H.; Hwang, J. H.; Ahn, D. W.; Kim, Y. T.; Yoon, Y. B.; Yoon, C. J.; Kang, S. G.; Chung, J. W. | Palliative treatment of unresectable hepatocellular carcinoma with obstructive jaundice using biliary drainage with subsequent transarterial chemoembolization | 2013 | Sep | J Palliat Med | 16 | 9 | 1026-33 | 10.1089/jpm.2013.0067 |  |
| 178 | Chok, K. S.; Cheung, T. T.; Lo, R. C.; Chu, F. S.; Tsang, S. H.; Chan, A. C.; Sharr, W. W.; Fung, J. Y.; Dai, W. C.; Chan, S. C.; Fan, S. T.; Lo, C. M. | Pilot study of high-intensity focused ultrasound ablation as a bridging therapy for hepatocellular carcinoma patients wait-listed for liver transplantation | 2014 | Aug | Liver Transpl | 20 | 8 | 912-21 | 10.1002/lt.23892 |  |
| 179 | Chu, H. H.; Kim, J. H.; Yoon, H. K.; Ko, H. K.; Gwon, D. I.; Kim, P. N.; Sung, K. B.; Ko, G. Y.; Kim, S. Y.; Park, S. H. | Chemoembolization Combined with Radiofrequency Ablation for Medium-Sized Hepatocellular Carcinoma: A Propensity-Score Analysis | 2019 | Oct | J Vasc Interv Radiol | 30 | 10 | 1533-1543 | 10.1016/j.jvir.2019.06.006 | Wrong patient population |
| 180 | Chu, Hee Ho; Gwon, Dong IL; Kim, Gun Ha; Kim, Jin Hyoung; Ko, Gi-Young; Shin, Ji Hoon; Ko, Heung-Kyu; Yoon, Hyun-Ki | Balloon-occluded transarterial chemoembolization versus conventional transarterial chemoembolization for the treatment of single hepatocellular carcinoma: a propensity score matching analysis | 2023 |  | European radiology | 33 | 4 | 2655-2664 |  | Wrong outcomes |
| 181 | Chuang, Y. H.; Cheng, Y. F.; Tsang, L. L.; Ou, H. Y.; Hsu, H. W.; Lim, W. X.; Huang, P. H.; Weng, C. C.; Yu, C. Y. | Efficacy and Safety of Combined Ethanol-Lipiodol Mixture and Drug-Eluting Bead TACE for Large HCC | 2023 |  | J Hepatocell Carcinoma | 10 |  | 81-90 | 10.2147/jhc.S398434 | Wrong patient population |
| 182 | Cohen, G. S.; Black, M. | Multidisciplinary management of hepatocellular carcinoma: a model for therapy | 2013 |  | J Multidiscip Healthc | 6 |  | 189-95 | 10.2147/jmdh.S41206 |  |
| 183 | Collettini, F.; Schnapauff, D.; Poellinger, A.; Denecke, T.; Schott, E.; Berg, T.; Wust, P.; Hamm, B.; Gebauer, B. | Hepatocellular carcinoma: computed-tomography-guided high-dose-rate brachytherapy (CT-HDRBT) ablation of large (5-7 cm) and very large (>7 cm) tumours | 2012 | May | Eur Radiol | 22 | 5 | 1101-9 | 10.1007/s00330-011-2352-7 |  |
| 184 | Collettini, F.; Schreiber, N.; Schnapauff, D.; Denecke, T.; Wust, P.; Schott, E.; Hamm, B.; Gebauer, B. | CT-guided high-dose-rate brachytherapy of unresectable hepatocellular carcinoma | 2015 | May | Strahlenther Onkol | 191 | 5 | 405-12 | 10.1007/s00066-014-0781-3 |  |
| 185 | Comito, T.; Loi, M.; Franzese, C.; Clerici, E.; Franceschini, D.; Badalamenti, M.; Teriaca, M. A.; Rimassa, L.; Pedicini, V.; Poretti, D.; Solbiati, L. A.; Torzilli, G.; Ceriani, R.; Lleo, A.; Aghemo, A.; Santoro, A.; Scorsetti, M. | Stereotactic Radiotherapy after Incomplete Transarterial (Chemo-) Embolization (TAE\TACE) versus Exclusive TAE or TACE for Treatment of Inoperable HCC: A Phase III Trial (NCT02323360) | 2022 | 16-Nov | Curr Oncol | 29 | 11 | 8802-8813 | 10.3390/curroncol29110692 | Wrong outcomes |
| 186 | Cosgrove, D. P.; Reyes, D. K.; Pawlik, T. M.; Feng, A. L.; Kamel, I. R.; Geschwind, J. F. | Open-Label Single-Arm Phase II Trial of Sorafenib Therapy with Drug-eluting Bead Transarterial Chemoembolization in Patients with Unresectable Hepatocellular Carcinoma: Clinical Results | 2015 | Nov | Radiology | 277 | 2 | 594-603 | 10.1148/radiol.2015142481 |  |
| 187 | Couri, T.; Pillai, A. | Goals and targets for personalized therapy for HCC | 2019 | Mar | Hepatol Int | 13 | 2 | 125-137 | 10.1007/s12072-018-9919-1 |  |
| 188 | Craig, P.; Young, S.; Golzarian, J. | Current Trends in the Treatment of Hepatocellular Carcinoma with Transarterial Embolization: Variability in Technical Aspects | 2019 | Sep | Cardiovasc Intervent Radiol | 42 | 9 | 1322-1328 | 10.1007/s00270-019-02232-7 |  |
| 189 | Criss, C. R.; Makary, M. S. | Recent Advances in Image-Guided Locoregional Therapies for Primary Liver Tumors | 2023 | 13-Jul | Biology (Basel) | 12 | 7 |  | 10.3390/biology12070999 |  |
| 190 | Cui, W.; Fan, W.; Zhang, Q.; Wen, J.; Huang, Y.; Yang, J.; Li, J.; Wang, Y. | Comparison of two transarterial chemoembolization regimens in patients with unresectable hepatocellular carcinoma: raltitrexed plus oxaliplatin versus 5-fluorouracil plus oxaliplatin | 2017 | 3-Oct | Oncotarget | 8 | 45 | 79165-79174 | 10.18632/oncotarget.16298 | Wrong patient population |
| 191 | Cura, M.; Heithaus, E. | Efficacy and safety of transarterial chemoembolization with 70- to 150-µm drug-eluting beads alone or in combination with 100- to 300-µm drug-eluting beads | 2018 | Oct | Proc (Bayl Univ Med Cent) | 31 | 4 | 428-431 | 10.1080/08998280.2018.1499363 | Wrong patient population |
| 192 | da Fonseca, L.; Carrilho, F. J. | Expanding TACTICS trial into a different setting in hepatocellular carcinoma | 2020 | May-Jun | Ann Hepatol | 19 | 3 | 230-231 | 10.1016/j.aohep.2020.02.001 |  |
| 193 | Dadrass, F.; Sher, A.; Kim, E. | Update on Locoregional Therapies for Liver Cancer: Radiation Segmentectomy | 2023 | 23-Nov | Curr Oncol | 30 | 12 | 10075-10084 | 10.3390/curroncol30120732 |  |
| 194 | Dai, Q. S.; Gu, H. L.; Ye, S.; Zhang, Y. J.; Lin, X. J.; Lau, W. Y.; Peng, Z. W.; Chen, M. S. | Transarterial chemoembolization vs. conservative treatment for unresectable infiltrating hepatocellular carcinoma: A retrospective comparative study | 2014 | Nov | Mol Clin Oncol | 2 | 6 | 1047-1054 | 10.3892/mco.2014.391 | Wrong patient population |
| 195 | Dai, W. C.; Cheung, T. T.; Chok, K. S.; Chan, A. C.; Sharr, W. W.; Tsang, S. H.; Yuen, W. K.; Chan, S. C.; Fan, S. T.; Lo, C. M.; Poon, R. T. | Radiofrequency ablation versus transarterial chemoembolization for unresectable solitary hepatocellular carcinomas sized 5-8 cm | 2015 | Mar | HPB (Oxford) | 17 | 3 | 226-31 | 10.1111/hpb.12324 | Wrong patient population |
| 196 | Dai, Y.; Jiang, H.; Jiang, H.; Zhao, S.; Zeng, X.; Sun, R.; Zheng, R. | Optimal timing of combining sorafenib with trans-arterial chemoembolization in patients with hepatocellular carcinoma: A meta-analysis | 2021 | Dec | Transl Oncol | 14 | 12 | 101238 | 10.1016/j.tranon.2021.101238 |  |
| 197 | Daniele, A.; Divella, R.; Quaranta, M.; Mattioli, V.; Casamassima, P.; Paradiso, A.; Garrisi, V. M.; Gadaleta, C. D.; Gadaleta-Caldarola, G.; Savino, E.; Maci, R.; Bellizzi, A.; Fazio, V. | Clinical and prognostic role of circulating MMP-2 and its inhibitor TIMP-2 in HCC patients prior to and after trans-hepatic arterial chemo-embolization | 2014 | Feb | Clin Biochem | 47 | 3 | 184-90 | 10.1016/j.clinbiochem.2013.11.022 |  |
| 198 | de Korompay, N.; Alshammari, M.; Klass, D.; Chou, F. Y.; Chung, J.; Ho, S.; Liu, D. M. | Intraprocedural Parenchymal Blood Volume Is a Predictor of Treatment Response for Chemoembolization in Hepatocellular Carcinoma: Results of a Prospective Study | 2018 | Jul | J Vasc Interv Radiol | 29 | 7 | 928-935 | 10.1016/j.jvir.2018.01.783 |  |
| 199 | Dejima, I.; Hori, A.; Nakamura, T.; Hori, S.; Sonomura, T.; Tedokon, Y. | [Transarterial Chemoembolization for Unresectable Gastric Cancer in the Esophago-Gastric Junction-A Case Report] | 2022 | Mar | Gan To Kagaku Ryoho | 49 | 3 | 321-323 |  |  |
| 200 | Delicque, J.; Hermida, M.; Piron, L.; Allimant, C.; Belgour, A.; Pageaux, G. P.; Ben Bouallegue, F.; Assenat, E.; Mariano-Goulart, D.; Guiu, B.; Cassinotto, C. | Intra arterial treatment of hepatocellular carcinoma: Comparison of MELD score variations between radio-embolization and chemo-embolization | 2019 | Nov | Diagn Interv Imaging | 100 | 11 | 689-697 | 10.1016/j.diii.2019.05.006 |  |
| 201 | Deng, G. L.; Zeng, S.; Shen, H. | Chemotherapy and target therapy for hepatocellular carcinoma: New advances and challenges | 2015 | 18-Apr | World J Hepatol | 7 | 5 | 787-98 | 10.4254/wjh.v7.i5.787 |  |
| 202 | Deng, Z.; Jin, Z.; Qin, Y.; Wei, M.; Wang, J.; Lu, T.; Zhang, L.; Zeng, J.; Bao, L.; Guo, Y.; Peng, M.; Xu, B.; Wen, Z. | Efficacy of the association liver partition and portal vein ligation for staged hepatectomy for the treatment of solitary huge hepatocellular carcinoma: a retrospective single-center study | 2021 | 30-Mar | World J Surg Oncol | 19 | 1 | 95 | 10.1186/s12957-021-02199-1 |  |
| 203 | Dev, A.; Sood, A.; Choudhury, S. R.; Karmakar, S. | Paclitaxel nanocrystalline assemblies as a potential transcatheter arterial chemoembolization (TACE) candidate for unresectable hepatocellular carcinoma | 2020 | Feb | Mater Sci Eng C Mater Biol Appl | 107 |  | 110315 | 10.1016/j.msec.2019.110315 |  |
| 204 | Dhamija, E.; Paul, S. B.; Gamanagatti, S. R.; Acharya, S. K. | Biliary complications of arterial chemoembolization of hepatocellular carcinoma | 2015 | Nov | Diagn Interv Imaging | 96 | 11 | 1169-75 | 10.1016/j.diii.2015.06.017 |  |
| 205 | Dhondt, Elisabeth; Lambert, Bieke; Hermie, Laurens; Huyck, Lynn; Vanlangenhove, Peter; Geerts, Anja; Verhelst, Xavier; Aerts, Maridi; Vanlander, Aude; Berrevoet, Frederik | 90Y radioembolization versus drug-eluting bead chemoembolization for unresectable hepatocellular carcinoma: results from the TRACE phase II randomized controlled trial | 2022 |  | Radiology | 303 | 3 | 699-710 |  | Wrong outcomes |
| 206 | Diaz-Rodriguez, P. E.; Muns-Aponte, C. M.; Velazquez-Acevedo, S. I.; Ortiz-Malave, C. M.; Acevedo, J.; Merced-Ortiz, F. G. | An Uncommon Case of Myocarditis Secondary to Durvalumab Plus Tremelimumab | 2023 | Aug | Cureus | 15 | 8 | e43628 | 10.7759/cureus.43628 |  |
| 207 | Dinh, V. Y.; Bhatia, S.; Narayanan, G.; Yrizarry, J.; Savaraj, N.; O'Brien, C.; Martin, P.; Feun, L. | Pilot Study of Intrahepatic Artery Chemotherapy in Combination with Sorafenib in Hepatocellular Carcinoma | 2016 | Jul | Anticancer Res | 36 | 7 | 3555-63 |  |  |
| 208 | Dong, G.; Zheng, Q. D.; Ma, M.; Wu, S. F.; Zhang, R.; Yao, R. R.; Dong, Y. Y.; Ma, H.; Gao, D. M.; Ye, S. L.; Cui, J. F.; Ren, Z. G.; Chen, R. X. | Angiogenesis enhanced by treatment damage to hepatocellular carcinoma through the release of GDF15 | 2018 | Mar | Cancer Med | 7 | 3 | 820-830 | 10.1002/cam4.1330 |  |
| 209 | Dong, H.; Ge, D.; Qu, B.; Zhu, P.; Wu, Q.; Wang, T.; Wang, J.; Li, Z. | Transarterial chemoembolization with or without multikinase inhibitors for patients with unresectable hepatocellular carcinoma: a systematic review and meta-analysis of randomized controlled trials | 2023 |  | Front Oncol | 13 |  | 1139025 | 10.3389/fonc.2023.1139025 | Wrong study design |
| 210 | Dong, J.; Li, W.; Dong, A.; Mao, S.; Shen, L.; Li, S.; Gong, X.; Wu, P. | Gene therapy for unresectable hepatocellular carcinoma using recombinant human adenovirus type 5 | 2014 | Aug | Med Oncol | 31 | 8 | 95 | 10.1007/s12032-014-0095-4 |  |
| 211 | Dong, X.; Wang, Y.; Hao, J.; Chen, L.; Sun, T.; Zhang, W.; Sun, B.; Zhu, L.; Guo, Y.; Zheng, C. | Evaluation of Transarterial Chemoembolization Protocol with Drug-Eluting Beads in Combination with Lipiodol for Hepatocellular Carcinoma: A Single-Center Controlled Study | 2022 |  | J Oncol | 2022 |  | 1090313 | 10.1155/2022/1090313 | Wrong outcomes |
| 212 | Dong, Z.; Lin, Y.; Lin, F.; Luo, X.; Lin, Z.; Zhang, Y.; Li, L.; Li, Z. P.; Feng, S. T.; Cai, H.; Peng, Z. | Prediction of Early Treatment Response to Initial Conventional Transarterial Chemoembolization Therapy for Hepatocellular Carcinoma by Machine-Learning Model Based on Computed Tomography | 2021 |  | J Hepatocell Carcinoma | 8 |  | 1473-1484 | 10.2147/jhc.S334674 |  |
| 213 | Dorn, D. P.; Bryant, M. K.; Zarzour, J.; Smith, J. K.; Redden, D. T.; Saddekni, S.; Abdel Aal, A. K.; Gray, S.; White, J.; Eckhoff, D. E.; DuBay, D. A. | Chemoembolization outcomes for hepatocellular carcinoma in cirrhotic patients with compromised liver function | 2014 | Jul | HPB (Oxford) | 16 | 7 | 648-55 | 10.1111/hpb.12194 | Wrong patient population |
| 214 | Duan, Ruihua; Gong, Fen; Wang, Yan; Huang, Caixia; Wu, Jiaming; Hu, Leihao; Liu, Min; Qiu, Shijun; Lu, Liming; Lin, Yisheng | Transarterial chemoembolization (TACE) plus tyrosine kinase inhibitors versus TACE in patients with hepatocellular carcinoma: a systematic review and meta-analysis | 2023 |  | World Journal of Surgical Oncology | 21 | 1 | 120 |  | Wrong patient population |
| 215 | Duan, X. H.; Ju, S. G.; Han, X. W.; Ren, J. Z.; Li, F. Y.; Chen, P. F.; Wu, Y. Y.; Li, H. | Arsenic trioxide-eluting Callispheres beads is more effective and equally tolerant compared with arsenic trioxide/lipiodol emulsion in the transcatheter arterial chemoembolization treatment for unresectable hepatocellular carcinoma patients | 2020 | Feb | Eur Rev Med Pharmacol Sci | 24 | 3 | 1468-1480 | 10.26355/eurrev_202002_20206 | Wrong patient population |
| 216 | Duan, X.; Li, H.; Chen, P.; Sun, T.; Kuang, D.; Lu, H.; Qiao, B.; Fan, Z.; Ren, Z.; Han, X. | Transcatheter arterial chemoembolization using CalliSpheres beads loaded with arsenic trioxide for unresectable large or huge hepatocellular carcinoma: a prospective study | 2023 | 15-Aug | Eur Radiol |  |  |  | 10.1007/s00330-023-10097-1 | Wrong patient population |
| 217 | Duan, X.; Li, H.; Kuang, D.; Chen, P.; Zhang, K.; Li, Y.; He, X.; Xing, C.; Wang, H.; Liu, Y.; Xie, L.; Zhang, S.; Zhang, Q.; Zhu, P.; Dong, H.; Xie, J.; Li, H.; Wang, Y.; Shi, M.; Jiang, G.; Xu, Y.; Zhou, S.; Shang, C.; Ren, J.; Han, X. | Transcatheter arterial chemoembolization plus apatinib with or without camrelizumab for unresectable hepatocellular carcinoma: a multicenter retrospective cohort study | 2023 | Aug | Hepatol Int | 17 | 4 | 915-926 | 10.1007/s12072-023-10519-8 |  |
| 218 | Duan, Xuhua; Liu, Juanfang; Han, Xinwei; Ren, Jianzhuang; Li, Hao; Li, Fengyao; Ju, Shuguang | Comparison of treatment response, survival profiles, as well as safety profiles between Callispheres® microsphere transarterial chemoembolization and conventional transarterial chemoembolization in huge hepatocellular carcinoma | 2022 |  | Frontiers in Oncology | 11 |  | 793581 |  | Wrong patient population |
| 219 | Dumago, Mark P; Agas, Ryan Anthony F; Jainar, Carl Jay E; Yap, Eugene T; Co, Lester Bryan A; Ortin, Teresa T Sy | Stereotactic Body Radiation Therapy With or Without Transarterial Chemoembolization Versus Transarterial Chemoembolization Alone in Early-Stage Hepatocellular Carcinoma: A Systematic Review and Meta-Analysis | 2023 |  | Journal of Gastrointestinal Cancer | | | 13-Jan |  |  |
| 220 | Duran, R.; Namur, J.; Pascale, F.; Czuczman, P.; Bascal, Z.; Kilpatrick, H.; Whomsley, R.; Ryan, S.; Lewis, A. L.; Denys, A. | Vandetanib-eluting Radiopaque Beads: Pharmacokinetics, Safety, and Efficacy in a Rabbit Model of Liver Cancer | 2019 | Dec | Radiology | 293 | 3 | 695-703 | 10.1148/radiol.2019190305 |  |
| 221 | Eggert, T.; Greten, T. F. | Current Standard and Future Perspectives in Non-Surgical Therapy for Hepatocellular Carcinoma | 2017 |  | Digestion | 96 | 1 | 4-Jan | 10.1159/000464282 |  |
| 222 | El-Serag, H. B.; Zhu, A. X.; Johnson, M. S. | The treatment path in hepatocellular carcinoma | 2017 | Aug | Clin Adv Hematol Oncol | 15 Suppl 9 | 8 | 20-Jan |  |  |
| 223 | Eltawil, K. M.; Berry, R.; Abdolell, M.; Molinari, M. | Quality of life and survival analysis of patients undergoing transarterial chemoembolization for primary hepatic malignancies: a prospective cohort study | 2012 | May | HPB (Oxford) | 14 | 5 | 341-50 | 10.1111/j.1477-2574.2012.00455.x |  |
| 224 | Endo, K.; Kuroda, H.; Abe, T.; Sato, H.; Kooka, Y.; Oikawa, T.; Sato, A.; Nishiya, M.; Sugai, T.; Takikawa, Y. | Two hepatectomy cases for initially unresectable hepatocellular carcinoma after achieving a radiological complete response to sequential therapy with lenvatinib and transcatheter arterial chemoembolization | 2021 | Oct | Hepatol Res | 51 | 10 | 1082-1086 | 10.1111/hepr.13665 |  |
| 225 | Eng, J. Y.; Soon, S. Y.; Winnie Ling, H. Y. | Curative lung metastectomy and complete pathological response after neo-adjuvant GEMOX chemotherapy for relapse fibrolamellar hepatocellular carcinoma | 2018 | Feb | Med J Malaysia | 73 | 1 | 46-48 |  |  |
| 226 | English, K.; Brodin, N. P.; Shankar, V.; Zhu, S.; Ohri, N.; Golowa, Y. S.; Cynamon, J.; Bellemare, S.; Kaubisch, A.; Kinkhabwala, M.; Kalnicki, S.; Garg, M. K.; Guha, C.; Kabarriti, R. | Association of Addition of Ablative Therapy Following Transarterial Chemoembolization With Survival Rates in Patients With Hepatocellular Carcinoma | 2020 | 2-Nov | JAMA Netw Open | 3 | 11 | e2023942 | 10.1001/jamanetworkopen.2020.23942 | Wrong patient population |
| 227 | Erhardt, A.; Kolligs, F.; Dollinger, M.; Schott, E.; Wege, H.; Bitzer, M.; Gog, C.; Lammert, F.; Schuchmann, M.; Walter, C.; Blondin, D.; Ohmann, C.; Häussinger, D. | TACE plus sorafenib for the treatment of hepatocellular carcinoma: results of the multicenter, phase II SOCRATES trial | 2014 | Nov | Cancer Chemother Pharmacol | 74 | 5 | 947-54 | 10.1007/s00280-014-2568-8 |  |
| 228 | Facciorusso, A.; Di Maso, M.; Muscatiello, N. | Drug-eluting beads versus conventional chemoembolization for the treatment of unresectable hepatocellular carcinoma: A meta-analysis | 2016 | Jun | Dig Liver Dis | 48 | 6 | 571-7 | 10.1016/j.dld.2016.02.005 | Wrong patient population |
| 229 | Facciorusso, A.; Mariani, L.; Sposito, C.; Spreafico, C.; Bongini, M.; Morosi, C.; Cascella, T.; Marchianò, A.; Camerini, T.; Bhoori, S.; Brunero, F.; Barone, M.; Mazzaferro, V. | Drug-eluting beads versus conventional chemoembolization for the treatment of unresectable hepatocellular carcinoma | 2016 | Mar | J Gastroenterol Hepatol | 31 | 3 | 645-53 | 10.1111/jgh.13147 | Wrong patient population |
| 230 | Facciorusso, A.; Serviddio, G.; Muscatiello, N. | Local ablative treatments for hepatocellular carcinoma: An updated review | 2016 | 6-Nov | World J Gastrointest Pharmacol Ther | 7 | 4 | 477-489 | 10.4292/wjgpt.v7.i4.477 |  |
| 231 | Fako, V.; Wang, X. W. | The status of transarterial chemoembolization treatment in the era of precision oncology | 2017 | Apr | Hepat Oncol | 4 | 2 | 55-63 | 10.2217/hep-2017-0009 |  |
| 232 | Falette Puisieux, M.; Pellat, A.; Assaf, A.; Ginestet, C.; Brezault, C.; Dhooge, M.; Soyer, P.; Coriat, R. | Therapeutic Management of Advanced Hepatocellular Carcinoma: An Updated Review | 2022 | 10-May | Cancers (Basel) | 14 | 10 |  | 10.3390/cancers14102357 |  |
| 233 | Fan, W.; Wu, Y.; Lu, M.; Yao, W.; Cui, W.; Zhao, Y.; Wang, Y.; Li, J. | A meta-analysis of the efficacy and safety of iodine [(131)I] metuximab infusion combined with TACE for treatment of hepatocellular carcinoma | 2019 | Aug | Clin Res Hepatol Gastroenterol | 43 | 4 | 451-459 | 10.1016/j.clinre.2018.09.006 | Wrong patient population |
| 234 | Fang, Z.; Yang, H.; Mao, Y. | Letter to the editor: Liver transarterial chemoembolization plus sunitinib for unresectable hepatocellular carcinoma | 2022 | Nov | Clin Res Hepatol Gastroenterol | 46 | 9 | 101984 | 10.1016/j.clinre.2022.101984 |  |
| 235 | Farid, K.; Elalfy, H.; Abo El-Khair, S. M.; Elgamal, H.; Besheer, T.; Elmokadem, A.; Shabana, W.; Abed, S.; Elegezy, M.; El-Khalek, A. A.; El-Morsy, A.; Negm, A.; Elsamanoudy, A. Z.; El Deek, B.; Amer, T.; El-Bendary, M. | Prognostic value of vascular endothelial growth factor in both conventional and drug eluting beads transarterial chemoembolization for treatment of unresectable hepatocellular carcinoma in HCV patients | 2020 | Dec | Expert Rev Gastroenterol Hepatol | 14 | 12 | 1203-1214 | 10.1080/17474124.2020.1823215 |  |
| 236 | Felga, G.; Evangelista, A. S.; Salvalaggio, P. R.; Curvelo, L. A.; Della Guardia, B.; Almeida, M. D.; Afonso, R. C.; Ferraz-Neto, B. H. | Clinical profile and liver explant findings in patients with and without pretransplant downstaging for hepatocellular carcinoma | 2012 | Oct | Transplant Proc | 44 | 8 | 2399-402 | 10.1016/j.transproceed.2012.07.135 |  |
| 237 | Feng, J. K.; Liu, Z. H.; Fu, Z. G.; Chai, Z. T.; Sun, J. X.; Wang, K.; Cheng, Y. Q.; Zhu, H. F.; Xiang, Y. J.; Zhou, L. P.; Shi, J.; Guo, W. X.; Zhai, J.; Cheng, S. Q. | Efficacy and safety of transarterial chemoembolization plus antiangiogenic- targeted therapy and immune checkpoint inhibitors for unresectable hepatocellular carcinoma with portal vein tumor thrombus in the real world | 2022 |  | Front Oncol | 12 |  | 954203 | 10.3389/fonc.2022.954203 |  |
| 238 | Feng, J. K.; Sun, J. X.; Liu, Z. H.; Gu, J. W.; Chen, Z. H.; Liu, C.; Guo, W. X.; Shi, J.; Cheng, S. Q. | Efficacy and Safety of Transarterial Chemoembolization for the Treatment of Unresectable Hepatocellular Carcinoma Associated with Bile Duct Tumor Thrombus: A Real-World Retrospective Cohort Study | 2021 |  | Cancer Manag Res | 13 |  | 3551-3560 | 10.2147/cmar.S307065 |  |
| 239 | Finn, R. S. | Advanced HCC: emerging molecular therapies | 2012 | Mar | Minerva Gastroenterol Dietol | 58 | 1 | 25-34 |  |  |
| 240 | Fiorentini, G.; Sarti, D.; Carandina, R.; Mulazzani, L.; Mincarelli, C.; Candelari, R.; Argirò, R.; Fiorentini, C.; Aliberti, C. | A review discussing the use of polyethylene glycol microspheres in the treatment of hepatocellular carcinoma | 2019 | Mar | Future Oncol | 15 | 7 | 695-703 | 10.2217/fon-2018-0425 | Wrong patient population |
| 241 | Fiorica, F.; Greco, C.; Boccia, S.; Sartori, S.; Stefanelli, A.; Cartei, F.; Ursino, S. | Hypofractionated stereotactic radiotherapy after transarterial chemoembolisation failure in an unresectable hepatocellular carcinoma: a case presentation | 2013 |  | Case Reports Hepatol | 2013 |  | 146215 | 10.1155/2013/146215 |  |
| 242 | Firouznia, K.; Ghanaati, H.; Alavian, S. M.; Azadeh, P.; Nasiri Toosi, M.; Haj Mirzaian, A.; Najafi, S.; Shakiba, M.; Jalali, A. H. | Transcatheter arterial chemoembolization therapy for patients with unresectable hepatocellular carcinoma | 2014 | Dec | Hepat Mon | 14 | 12 | e25792 | 10.5812/hepatmon.25792 | Wrong patient population |
| 243 | Forner, A.; Llovet, J. M.; Bruix, J. | Chemoembolization for intermediate HCC: is there proof of survival benefit? | 2012 | Apr | J Hepatol | 56 | 4 | 984-6 | 10.1016/j.jhep.2011.08.017 |  |
| 244 | Freeman, E.; Cheung, W.; Ferdousi, S.; Kavnoudias, H.; Majeed, A.; Kemp, W.; Roberts, S. K. | Irreversible electroporation versus radiofrequency ablation for hepatocellular carcinoma: a single centre propensity-matched comparison | 2021 | Aug | Scand J Gastroenterol | 56 | 8 | 942-947 | 10.1080/00365521.2021.1930145 |  |
| 245 | Frenette, C. T.; Osorio, R. C.; Stark, J.; Fok, B.; Boktour, M. R.; Guy, J.; Rhee, J.; Osorio, R. W. | Conventional TACE and drug-eluting bead TACE as locoregional therapy before orthotopic liver transplantation: comparison of explant pathologic response | 2014 | 15-Oct | Transplantation | 98 | 7 | 781-7 | 10.1097/tp.0000000000000121 |  |
| 246 | Fu, F.; Wan, Y.; Wu, T. | Kanglaite injection combined with hepatic arterial intervention for unresectable hepatocellular carcinoma: a meta-analysis | 2014 | Aug | J Cancer Res Ther | 10 Suppl 1 |  | 38-41 | 10.4103/0973-1482.139753 |  |
| 247 | Fu, Y.; Zhao, X.; Yun, Q.; Zhu, X.; Zhu, Y.; Li, Q.; Hu, K.; Wang, J.; Qiao, Z. | Transarterial chemoembolization (TACE) plus percutaneous ethanol injection (PEI) for the treatment of unresectable hepatocellular carcinoma: a meta-analysis of randomized controlled trials | 2015 |  | Int J Clin Exp Med | 8 | 7 | 10388-400 |  | Wrong patient population |
| 248 | Fu, Z.; Li, X.; Zhong, J.; Chen, X.; Cao, K.; Ding, N.; Liu, L.; Zhang, X.; Zhai, J.; Qu, Z. | Lenvatinib in combination with transarterial chemoembolization for treatment of unresectable hepatocellular carcinoma (uHCC): a retrospective controlled study | 2021 | Jun | Hepatol Int | 15 | 3 | 663-675 | 10.1007/s12072-021-10184-9 | Wrong patient population |
| 249 | Fujita, M.; Okai, K.; Hayashi, M.; Abe, K.; Takahashi, A.; Kimura, T.; Kenjo, A.; Marubashi, S.; Hashimoto, Y.; Ohira, H. | Huge Hepatocellular Carcinoma Treated with Radical Hepatectomy after Drug-eluting Bead Transarterial Chemoembolization | 2019 | 15-Apr | Intern Med | 58 | 8 | 1103-1110 | 10.2169/internalmedicine.1214-18 |  |
| 250 | Fukushima, T.; Morimoto, M.; Kobayashi, S.; Ueno, M.; Sano, Y.; Kawano, K.; Asama, H.; Nagashima, S.; Maeda, S. | Repeated transarterial chemoembolization with epirubicin-loaded superabsorbent polymer microspheres vs. conventional transarterial chemoembolization for hepatocellular carcinoma | 2021 | Jun | Mol Clin Oncol | 14 | 6 | 119 | 10.3892/mco.2021.2281 |  |
| 251 | Gan, L.; Lang, M.; Tian, X.; Ren, S.; Li, G.; Liu, Y.; Han, R.; Zhu, K.; Li, H.; Wu, Q.; Cui, Y.; Zhang, W.; Fang, F.; Li, Q.; Song, T. | A Retrospective Analysis of Conversion Therapy with Lenvatinib, Sintilimab, and Arterially-Directed Therapy in Patients with Initially Unresectable Hepatocellular Carcinoma | 2023 |  | J Hepatocell Carcinoma | 10 |  | 673-686 | 10.2147/jhc.S404675 |  |
| 252 | Gao, Huifeng; He, Jian; Cheng, Chien-shan; Zhuang, Liping; Chen, Hao; Meng, Zhiqiang | Unresectable hepatocellular carcinoma: transarterial chemoembolisation plus Huachansu"“a single-center randomised controlled trial | 2023 |  | BMJ Supportive & Palliative Care | | |  |  |  |
| 253 | Gao, S.; Yang, Z.; Zheng, Z.; Yao, J.; Deng, M.; Xie, H.; Zheng, S.; Zhou, L. | Doxorubicin-eluting bead versus conventional TACE for unresectable hepatocellular carcinoma: a meta-analysis | 2013 | Jun | Hepatogastroenterology | 60 | 124 | 813-20 | 10.5754/hge121025 | Article inaccessible |
| 254 | Gao, X.; Chen, Z.; Chen, Z.; Liu, X.; Luo, Y.; Xiao, J.; Gao, Y.; Ma, Y.; Liu, C.; Leo, H. L.; Yu, H.; Guo, Q. | Visualization and Evaluation of Chemoembolization on a 3D Decellularized Organ Scaffold | 2021 | 13-Dec | ACS Biomater Sci Eng | 7 | 12 | 5642-5653 | 10.1021/acsbiomaterials.1c01005 |  |
| 255 | Gao, Y.; Li, Z.; Hong, Y.; Li, T.; Hu, X.; Sun, L.; Chen, Z.; Chen, Z.; Luo, Z.; Wang, X.; Kong, J.; Li, G.; Wang, H. L.; Leo, H. L.; Yu, H.; Xi, L.; Guo, Q. | Decellularized liver as a translucent ex vivo model for vascular embolization evaluation | 2020 | May | Biomaterials | 240 |  | 119855 | 10.1016/j.biomaterials.2020.119855 |  |
| 256 | Gasparrini, F.; Lenci, I.; Gagliardi, M. G.; Spada, M.; Salimei, F.; Orlacchio, A. | Repeated TACE in HCC after Fontan surgery and situs viscerum inversus: A case report | 2021 | Sep | Radiol Case Rep | 16 | 9 | 2564-2569 | 10.1016/j.radcr.2021.06.031 |  |
| 257 | Ge, N. J.; Shi, Z. Y.; Yu, X. H.; Huang, X. J.; Wu, Y. S.; Chen, Y. Y.; Zhang, J.; Yang, Y. F. | Genetic Variants in ASCT2 Gene are Associated with the Prognosis of Transarterial Chemoembolisation-Treated Early- Stage Hepatocelluar Carcinoma | 2015 |  | Asian Pac J Cancer Prev | 16 | 9 | 4103-7 | 10.7314/apjcp.2015.16.9.4103 |  |
| 258 | Georgiades, C.; Geschwind, J. F.; Harrison, N.; Hines-Peralta, A.; Liapi, E.; Hong, K.; Wu, Z.; Kamel, I.; Frangakis, C. | Lack of response after initial chemoembolization for hepatocellular carcinoma: does it predict failure of subsequent treatment? | 2012 | Oct | Radiology | 265 | 1 | 115-23 | 10.1148/radiol.12112264 |  |
| 259 | Geschwind, J. F.; Gholam, P. M.; Goldenberg, A.; Mantry, P.; Martin, R. C.; Piperdi, B.; Zigmont, E.; Imperial, J.; Babajanyan, S.; Foreman, P. K.; Cohn, A. | Use of Transarterial Chemoembolization (TACE) and Sorafenib in Patients with Unresectable Hepatocellular Carcinoma: US Regional Analysis of the GIDEON Registry | 2016 | Feb | Liver Cancer | 5 | 1 | 37-46 | 10.1159/000367757 | Wrong patient population |
| 260 | Geschwind, Jean-François; Kudo, Masatoshi; Marrero, Jorge A.; Venook, Alan P.; Chen, Xiao-Ping; Bronowicki, Jean-Pierre; Dagher, Lucy; Furuse, Junji; Ladrón de Guevara, Laura; Papandreou, Christos | TACE treatment in patients with sorafenib-treated unresectable hepatocellular carcinoma in clinical practice: final analysis of GIDEON | 2016 |  | Radiology | 279 | 2 | 630-640 |  | Wrong patient population |
| 261 | Gholam, P. M.; Iyer, R.; Johnson, M. S. | Multidisciplinary Management of Patients with Unresectable Hepatocellular Carcinoma: A Critical Appraisal of Current Evidence | 2019 | 22-Jun | Cancers (Basel) | 11 | 6 |  | 10.3390/cancers11060873 |  |
| 262 | Gish, R. C.; Scott, J. A.; Yu, H. | Transarterial chemoembolization of hepatocellular carcinoma via extrahepatic collateral artery from a supraduodenal and cystic artery trunk, originating from the gastroduodenal artery: A case report | 2021 | Oct | Radiol Case Rep | 16 | 10 | 3064-3067 | 10.1016/j.radcr.2021.07.041 |  |
| 263 | Gjoreski, A.; Jovanoska, I.; Risteski, F.; Prgova Veljanova, B.; Nedelkovski, D.; Dimov, V.; Popova Jovanovska, R.; Grozdanovska Angelovska, B.; Mitrevski, N.; Dimova, B. | Single-center randomized trial comparing conventional chemoembolization versus doxorubicin-loaded polyethylene glycol microspheres for early- and intermediate-stage hepatocellular carcinoma | 2021 | 1-May | Eur J Cancer Prev | 30 | 3 | 258-266 | 10.1097/cej.0000000000000623 | Wrong patient population |
| 264 | Gjoreski, A.; Popova-Jovanovska, R.; Eftimovska-Rogac, I.; Vejseli, J. | Safety Profile and Efficacy of Chemoembolization with Doxorubicin - Loaded Polyethylene Glycol Microspheres in Patients with Hepatocellular Carcinoma | 2019 | 15-Mar | Open Access Maced J Med Sci | 7 | 5 | 742-746 | 10.3889/oamjms.2019.179 | Wrong patient population |
| 265 | Goda, Y.; Morimoto, M.; Irie, K.; Kobayashi, S.; Ueno, M.; Moriya, S.; Tezuka, S.; Ohkawa, S.; Morinaga, S.; Numata, K.; Tanaka, K.; Maeda, S. | Switch to miriplatin for multinodular hepatocellular carcinoma unresponsive to transarterial chemoembolization with epirubicin: a prospective study | 2017 | 1-Dec | Jpn J Clin Oncol | 47 | 12 | 1151-1156 | 10.1093/jjco/hyx131 |  |
| 266 | Golfieri, R. | SIR-Spheres yttrium-90 radioembolization for the treatment of unresectable liver cancers | 2014 | Jun | Hepat Oncol | 1 | 3 | 265-283 | 10.2217/hep.14.6 |  |
| 267 | Golfieri, R.; Bilbao, J. I.; Carpanese, L.; Cianni, R.; Gasparini, D.; Ezziddin, S.; Paprottka, P. M.; Fiore, F.; Cappelli, A.; Rodriguez, M.; Ettorre, G. M.; Saltarelli, A.; Geatti, O.; Ahmadzadehfar, H.; Haug, A. R.; Izzo, F.; Giampalma, E.; Sangro, B.; Pizzi, G.; Notarianni, E.; Vit, A.; Wilhelm, K.; Jakobs, T. F.; Lastoria, S. | Comparison of the survival and tolerability of radioembolization in elderly vs. younger patients with unresectable hepatocellular carcinoma | 2013 | Oct | J Hepatol | 59 | 4 | 753-61 | 10.1016/j.jhep.2013.05.025 |  |
| 268 | Gomes, A. S.; Monteleone, P. A.; Sayre, J. W.; Finn, R. S.; Sadeghi, S.; Tong, M. J.; Britten, C. D.; Busuttil, R. W. | Comparison of Triple-Drug Transcatheter Arterial Chemoembolization (TACE) With Single-Drug TACE Using Doxorubicin-Eluting Beads: Long-Term Survival in 313 Patients | 2017 | Oct | AJR Am J Roentgenol | 209 | 4 | 722-732 | 10.2214/ajr.17.18219 |  |
| 269 | Goto, Y.; Niizeki, T.; Fukutomi, S.; Shirono, T.; Shimose, S.; Iwamoto, H.; Kojima, S.; Kanno, H.; Uchino, Y.; Sasaki, S.; Shirahama, N.; Muroya, D.; Nomura, Y.; Akashi, M.; Nakayama, G.; Hirakawa, Y.; Sato, T.; Yoshitomi, M.; Sakai, H.; Hisaka, T.; Kakuma, T.; Koga, H.; Torimura, T.; Akagi, Y.; Okuda, K. | Preoperative Chemotherapy Followed by Hepatectomy for Potentially Resectable UICC7 Stage IIIA, IIIB Hepatocellular Carcinoma; A Phase II Clinical Trial | 2023 | 25-Sep | Kurume Med J | 68 | 3.4 | 239-245 | 10.2739/kurumemedj.MS6834010 |  |
| 270 | Gross, A.; Albrecht, T. | Transarterial Chemoembolisation (TACE) with Degradable Starch Microspheres (DSM) and Anthracycline in Patients with Locally Extensive Hepatocellular Carcinoma (HCC): Safety and Efficacy | 2020 | Mar | Cardiovasc Intervent Radiol | 43 | 3 | 402-410 | 10.1007/s00270-019-02364-w |  |
| 271 | Grumme, J.; Werncke, T.; Meine, T. C.; Becker, L. S.; Kloeckner, R.; Maschke, S. K.; Kirstein, M. M.; Vogel, A.; Wacker, F. K.; Meyer, B. C.; Hinrichs, J. B.; Rodt, T. | Transarterial chemoembolization for hepatocellular carcinoma: quality of life, tumour response, safety and survival comparing two types of drug-eluting beads | 2020 | Oct | Abdom Radiol (NY) | 45 | 10 | 3326-3336 | 10.1007/s00261-019-02349-w | Wrong patient population |
| 272 | Guiu, B.; Chevallier, P.; Assenat, E.; Barbier, E.; Merle, P.; Bouvier, A.; Dumortier, J.; Nguyen-Khac, E.; Gugenheim, J.; Rode, A.; Oberti, F.; Valette, P. J.; Yzet, T.; Chevallier, O.; Barbare, J. C.; Latournerie, M.; Boulin, M. | Idarubicin-loaded Beads for Chemoembolization of Hepatocellular Carcinoma: The IDASPHERE II Single-Arm Phase II Trial | 2019 | Jun | Radiology | 291 | 3 | 801-808 | 10.1148/radiol.2019182399 | Wrong patient population |
| 273 | Guiu, B.; Schmitt, A.; Reinhardt, S.; Fohlen, A.; Pohl, T.; Wendremaire, M.; Denys, A.; Blümmel, J.; Boulin, M. | Idarubicin-loaded ONCOZENE drug-eluting embolic agents for chemoembolization of hepatocellular carcinoma: in vitro loading and release and in vivo pharmacokinetics | 2015 | Feb | J Vasc Interv Radiol | 26 | 2 | 262-70 | 10.1016/j.jvir.2014.08.021 |  |
| 274 | Gummadi, S.; Stanczak, M.; Lyshchik, A.; Forsberg, F.; Shaw, C. M.; Eisenbrey, J. R. | Contrast-enhanced ultrasound identifies early extrahepatic collateral contributing to residual hepatocellular tumor viability after transarterial chemoembolization | 2018 | Jun | Radiol Case Rep | 13 | 3 | 713-718 | 10.1016/j.radcr.2018.04.001 |  |
| 275 | Guo, B.; Zhou, Y.; Liu, Z.; Chen, Q.; Chen, X.; Xiao, Z. | TACE plus PD-1 successfully achieves conversion therapy for unresectable HCC with multiple macrovascular invasion: Case report | 2023 | Aug | Clin Res Hepatol Gastroenterol | 47 | 7 | 102169 | 10.1016/j.clinre.2023.102169 |  |
| 276 | Guo, J. G.; Zhao, L. P.; Rao, Y. F.; Gao, Y. P.; Guo, X. J.; Zhou, T. Y.; Feng, Z. Y.; Sun, J. H.; Lu, X. Y. | Novel multimodal analgesia regimen improves post-TACE pain in patients with hepatocellular carcinoma | 2018 | Dec | Hepatobiliary Pancreat Dis Int | 17 | 6 | 510-516 | 10.1016/j.hbpd.2018.08.001 |  |
| 277 | Guo, J. H.; Zhu, X.; Li, X. T.; Yang, R. J. | Impact of serum vascular endothelial growth factor on prognosis in patients with unresectable hepatocellular carcinoma after transarterial chemoembolization | 2012 | Mar | Chin J Cancer Res | 24 | 1 | 36-43 | 10.1007/s11670-012-0036-8 |  |
| 278 | Guo, J.; Bao, X.; Liu, F.; Guo, J.; Wu, Y.; Xiong, F.; Lu, J. | Efficacy of Invariant Natural Killer T Cell Infusion Plus Transarterial Embolization vs Transarterial Embolization Alone for Hepatocellular Carcinoma Patients: A Phase 2 Randomized Clinical Trial | 2023 |  | J Hepatocell Carcinoma | 10 |  | 1379-1388 | 10.2147/jhc.S416933 |  |
| 279 | Guo, L.; Wei, X.; Feng, S.; Zhai, J.; Guo, W.; Shi, J.; Lau, W. Y.; Meng, Y.; Cheng, S. | Radiotherapy prior to or after transcatheter arterial chemoembolization for the treatment of hepatocellular carcinoma with portal vein tumor thrombus: a randomized controlled trial | 2022 | Dec | Hepatol Int | 16 | 6 | 1368-1378 | 10.1007/s12072-022-10423-7 |  |
| 280 | Guo, P.; Pi, X.; Gao, F.; Li, Q.; Li, D.; Feng, W.; Cao, W. | Transarterial chemoembolization plus lenvatinib with or without programmed death-1 inhibitors for patients with unresectable hepatocellular carcinoma: A propensity score matching study | 2022 |  | Front Oncol | 12 |  | 945915 | 10.3389/fonc.2022.945915 |  |
| 281 | Guo, W.; Chen, S.; Wu, Z.; Zhuang, W.; Yang, J. | Efficacy and Safety of Transarterial Chemoembolization Combined With Anlotinib for Unresectable Hepatocellular Carcinoma: A Retrospective Study | 2020 | Jan-Dec | Technol Cancer Res Treat | 19 |  | 1.53E+15 | 10.1177/1533033820965587 | Wrong patient population |
| 282 | Guo, W.; Gao, J.; Zhuang, W.; Wu, Z.; Li, B.; Chen, S. | Efficacy and safety of hepatic arterial infusion chemotherapy combined with transarterial embolization for unresectable hepatocellular carcinoma: A propensity score-matching cohort study | 2020 | Jun | JGH Open | 4 | 3 | 477-483 | 10.1002/jgh3.12285 | Wrong outcomes |
| 283 | Ha, F.; Wang, X.; Han, T.; Jia, K.; Wang, S.; Song, D. | Value of Preoperative Systemic Immune-Inflammation Index and Albumin-Bilirubin Grade in Patients with Hepatocellular Carcinoma Undergoing Transarterial Embolization | 2023 | Apr | Turk J Gastroenterol | 34 | 4 | 413-420 | 10.5152/tjg.2023.22296 |  |
| 284 | Ha, Y.; Lee, J. B.; Shim, J. H.; Kim, K. M.; Lim, Y. S.; Yoon, H. K.; Shin, Y. M.; Lee, H. C. | Validation and reappraisal of the assessment for retreatment with transarterial chemoembolization score for unresectable non-metastatic hepatocellular carcinoma in a hepatitis b virus-endemic region | 2016 | Oct | Eur Radiol | 26 | 10 | 3510-8 | 10.1007/s00330-015-4185-2 |  |
| 285 | Habbel, V. S. A.; Zeile, M.; Stavrou, G. A.; Wacker, F.; Brüning, R.; Oldhafer, K. J.; Rodt, T. | Correlation between SACE (Subjective Angiographic Chemoembolization Endpoint) score and tumor response and its impact on survival after DEB-TACE in patients with hepatocellular carcinoma | 2019 | Oct | Abdom Radiol (NY) | 44 | 10 | 3463-3479 | 10.1007/s00261-019-02128-7 | Wrong patient population |
| 286 | Hamaya, S.; Oura, K.; Morishita, A.; Masaki, T. | Cisplatin in Liver Cancer Therapy | 2023 | 29-Jun | Int J Mol Sci | 24 | 13 |  | 10.3390/ijms241310858 |  |
| 287 | Hammond, J. S.; Franko, J.; Holloway, S. E.; Heckman, J. T.; Orons, P. D.; Gamblin, T. C. | Gemcitabine transcatheter arterial chemoembolization for unresectable hepatocellular carcinoma | 2014 | Jul-Aug | Hepatogastroenterology | 61 | 133 | 1339-43 |  | Wrong patient population |
| 288 | Han, G.; Yang, J.; Shao, G.; Teng, G.; Wang, M.; Yang, J.; Liu, Z.; Feng, G.; Yang, R.; Lu, L.; Chao, Y.; Wang, J. | Sorafenib in combination with transarterial chemoembolization in Chinese patients with hepatocellular carcinoma: a subgroup interim analysis of the START trial | 2013 | Mar | Future Oncol | 9 | 3 | 403-10 | 10.2217/fon.13.11 | Wrong patient population |
| 289 | Han, Kichang; Kim, Jin Hyoung | Transarterial chemoembolization in hepatocellular carcinoma treatment: Barcelona clinic liver cancer staging system | 2015 |  | World journal of gastroenterology: WJG | 21 | 36 | 10327 |  |  |
| 290 | Han, T.; Yang, X.; Zhang, Y.; Li, G.; Liu, L.; Chen, T.; Zheng, Z. | The clinical safety and efficacy of conventional transcatheter arterial chemoembolization and drug-eluting beads-transcatheter arterial chemoembolization for unresectable hepatocellular carcinoma: A meta-analysis | 2019 | 13-Nov | Biosci Trends | 13 | 5 | 374-381 | 10.5582/bst.2019.01153 | Wrong patient population |
| 291 | Han, Y.; Cao, G.; Sun, B.; Wang, J.; Yan, D.; Xu, H.; Shi, Q.; Liu, Z.; Zhi, W.; Xu, L.; Liu, B.; Zou, Y. | Regorafenib combined with transarterial chemoembolization for unresectable hepatocellular carcinoma: a real-world study | 2021 | 20-Oct | BMC Gastroenterol | 21 | 1 | 393 | 10.1186/s12876-021-01967-3 |  |
| 292 | Han, Z.; Yang, F.; Zhang, Y.; Wang, J.; Ni, Q.; Zhu, H.; Zhou, X.; Gao, H.; Lu, J. | Prognostic efficacy and prognostic factors of TACE plus TKI with ICIs for the treatment of unresectable hepatocellular carcinoma: A retrospective study | 2022 |  | Front Oncol | 12 |  | 1029951 | 10.3389/fonc.2022.1029951 |  |
| 293 | Hao, M. Z.; Lin, H. L.; Chen, Q. Z.; Hu, Y. B.; Chen, J. B.; Zheng, J. X.; Zhou, D.; Zhang, H. | Safety and efficacy of transcatheter arterial chemoembolization with embospheres in treatment of hepatocellular carcinoma | 2017 | Jan | J Dig Dis | 18 | 1 | 31-39 | 10.1111/1751-2980.12435 | Wrong patient population |
| 294 | Hasan, I.; Gani, R. A.; Lesmana, L. A.; Kresno, S. B.; Pandelaki, J.; Suwarto, S. | The Association between Peripheral Th17, Th1, IL-17, and IFN-γ Levels and TACE Response in Patients with Unresectable Hepatocellular Carcinoma with or without Cirrhosis | 2020 | Oct | Acta Med Indones | 52 | 4 | 326-333 |  |  |
| 295 | Hassanin, T. M.; Fouad, Y.; Hassnine, A.; Eisawy, M.; Farag, N.; Abdel Ghany, W. | Quality of Life after Transcatheter Arterial Chemoembolization Combined with Radiofrequency Ablation in Patients with Unresectable Hepatocellular Carcinoma Compared with Transcatheter Arterial Chemoembolization alone | 2021 | 1-Apr | Asian Pac J Cancer Prev | 22 | 4 | 1255-1261 | 10.31557/apjcp.2021.22.4.1255 |  |
| 296 | He, C.; Ge, N.; Wang, X.; Li, H.; Chen, S.; Yang, Y. | Conversion Therapy of Large Unresectable Hepatocellular Carcinoma With Ipsilateral Portal Vein Tumor Thrombus Using Portal Vein Embolization Plus Transcatheter Arterial Chemoembolization | 2022 |  | Front Oncol | 12 |  | 923566 | 10.3389/fonc.2022.923566 |  |
| 297 | He, C.; Zhang, Y.; Lin, X. | Increased Overall Survival and Decreased Cancer-Specific Mortality in Patients with Hepatocellular Carcinoma Treated by Transarterial Chemoembolization and Human Adenovirus Type-5 Combination Therapy: a Competing Risk Analysis | 2018 | Jun | J Gastrointest Surg | 22 | 6 | 989-997 | 10.1007/s11605-018-3703-3 |  |
| 298 | He, K.; Yang, Z.; Liu, X.; Yang, Y.; Song, W.; Wang, S.; Chen, Y. | Identification of Potential Predictors of Prognosis and Sorafenib-Associated Survival Benefits in Patients with Hepatocellular Carcinoma after Transcatheter Arterial Chemoembolization | 2022 | 29-Dec | Curr Oncol | 30 | 1 | 476-491 | 10.3390/curroncol30010038 |  |
| 299 | He, M. K.; Le, Y.; Li, Q. J.; Yu, Z. S.; Li, S. H.; Wei, W.; Guo, R. P.; Shi, M. | Hepatic artery infusion chemotherapy using mFOLFOX versus transarterial chemoembolization for massive unresectable hepatocellular carcinoma: a prospective non-randomized study | 2017 | 23-Oct | Chin J Cancer | 36 | 1 | 83 | 10.1186/s40880-017-0251-2 | Wrong patient population |
| 300 | He, M.; Jiang, N.; Yin, X.; Xu, A.; Mu, K. | Conventional and drug-eluting beads transarterial chemoembolization in patients with unresectable intrahepatic cholangiocarcinoma: a systematic review and pooled analysis | 2023 | Jan | J Cancer Res Clin Oncol | 149 | 1 | 531-540 | 10.1007/s00432-022-04485-1 |  |
| 301 | He, M.; Li, Q.; Shen, J.; Tan, G.; Li, Q.; Lai, J.; Wei, W.; Zhang, Y.; Zou, R.; Chen, M.; Guo, R.; Shi, M. | Predictive factors for the benefit of triple-drug transarterial chemoembolization for patients with unresectable hepatocellular carcinoma | 2019 | Aug | Cancer Med | 8 | 9 | 4200-4213 | 10.1002/cam4.2355 |  |
| 302 | He, P.; Ren, E.; Chen, B.; Chen, H.; Cheng, H.; Gao, X.; Liang, X.; Liu, H.; Li, J.; Li, B.; Chen, A.; Chu, C.; Chen, X.; Mao, J.; Zhang, Y.; Liu, G. | A super-stable homogeneous Lipiodol-hydrophilic chemodrug formulation for treatment of hepatocellular carcinoma | 2022 |  | Theranostics | 12 | 4 | 1769-1782 | 10.7150/thno.68456 |  |
| 303 | He, P.; Ren, E.; Chen, B.; Chen, H.; Cheng, H.; Gao, X.; Liang, X.; Liu, H.; Li, J.; Li, B.; Chen, A.; Chu, C.; Chen, X.; Mao, J.; Zhang, Y.; Liu, G. | A super-stable homogeneous Lipiodol-hydrophilic chemodrug formulation for treatment of hepatocellular carcinoma | 2022 |  | Theranostics | 12 | 4 | 1769-1782 | 10.7150/thno.68456 |  |
| 304 | He, Q.; Lu, W. S.; Liu, Y.; Guan, Y. S.; Kuang, A. R. | 131I-labeled metuximab combined with chemoembolization for unresectable hepatocellular carcinoma | 2013 | 21-Dec | World J Gastroenterol | 19 | 47 | 9104-10 | 10.3748/wjg.v19.i47.9104 |  |
| 305 | Hendi, M.; Mou, Y.; Lv, J.; Zhang, B.; Cai, X. | Hepatic Arterial Infusion Chemotherapy Is a Feasible Treatment Option for Hepatocellular Carcinoma: A New Update | 2021 | Oct | Gastrointest Tumors | 8 | 4 | 145-152 | 10.1159/000516405 |  |
| 306 | Henry, J. C.; Malhotra, L.; Khabiri, H.; Guy, G.; Michaels, A.; Hanje, J.; Azevedo, M.; Bloomston, M.; Schmidt, C. R. | Best radiological response to trans-arterial chemoembolization for hepatocellular carcinoma does not imply better outcomes | 2013 | Mar | HPB (Oxford) | 15 | 3 | 196-202 | 10.1111/j.1477-2574.2012.00529.x | Wrong patient population |
| 307 | Herman, T.; Kaempf, A.; Schlansky, B.; Nabavizadeh, N. | Low Utilization of External Beam Radiation Therapy for Patients With Unresectable Hepatocellular Carcinoma: An Analysis of the United Network for Organ Sharing Database | 2022 | 1-Oct | Int J Radiat Oncol Biol Phys | 114 | 2 | 231-237 | 10.1016/j.ijrobp.2022.05.028 |  |
| 308 | Hidaka, H.; Izumi, N.; Aramaki, T.; Ikeda, M.; Inaba, Y.; Imanaka, K.; Okusaka, T.; Kanazawa, S.; Kaneko, S.; Kora, S.; Saito, H.; Furuse, J.; Matsui, O.; Yamashita, T.; Yokosuka, O.; Morita, S.; Arioka, H.; Kudo, M.; Arai, Y. | Subgroup analysis of efficacy and safety of orantinib in combination with TACE in Japanese HCC patients in a randomized phase III trial (ORIENTAL) | 2019 | 3-May | Med Oncol | 36 | 6 | 52 | 10.1007/s12032-019-1272-2 | Wrong outcomes |
| 309 | Hieu, L. T.; Van Thanh, L.; Van Quang, V.; Khue, D. K.; Anh, N. H. N.; Tien, D. D. | Spinal cord ischemia after transcatheter artery chemoembolization for hepatocellular carcinoma: A case-report | 2023 | May | Int J Surg Case Rep | 106 |  | 108258 | 10.1016/j.ijscr.2023.108258 |  |
| 310 | Higashihara, H.; Osuga, K.; Onishi, H.; Nakamoto, A.; Tsuboyama, T.; Maeda, N.; Hori, M.; Kim, T.; Tomiyama, N. | Diagnostic accuracy of C-arm CT during selective transcatheter angiography for hepatocellular carcinoma: comparison with intravenous contrast-enhanced, biphasic, dynamic MDCT | 2012 | Apr | Eur Radiol | 22 | 4 | 872-9 | 10.1007/s00330-011-2324-y |  |
| 311 | Higashihara, H.; Osuga, K.; Onishi, H.; Nakamoto, A.; Tsuboyama, T.; Tomiyama, N. | The diagnostic value of dual-phase cone-beam CT during hepatic arteriography in transarterial chemoembolization for hepatocellular carcinoma | 2021 | 26-Mar | Medicine (Baltimore) | 100 | 12 | e24902 | 10.1097/md.0000000000024902 |  |
| 312 | Hiraoka, A.; Kumada, T.; Atsukawa, M.; Hirooka, M.; Tsuji, K.; Ishikawa, T.; Takaguchi, K.; Kariyama, K.; Itobayashi, E.; Tajiri, K.; Shimada, N.; Shibata, H.; Ochi, H.; Tada, T.; Toyoda, H.; Nouso, K.; Tsutsui, A.; Nagano, T.; Itokawa, N.; Hayama, K.; Imai, M.; Joko, K.; Koizumi, Y.; Hiasa, Y.; Michitaka, K. | Early Relative Change in Hepatic Function with Lenvatinib for Unresectable Hepatocellular Carcinoma | 2019 |  | Oncology | 97 | 6 | 334-340 | 10.1159/000502095 |  |
| 313 | Hiraoka, A.; Kumada, T.; Atsukawa, M.; Hirooka, M.; Tsuji, K.; Ishikawa, T.; Takaguchi, K.; Kariyama, K.; Itobayashi, E.; Tajiri, K.; Shimada, N.; Shibata, H.; Ochi, H.; Tada, T.; Toyoda, H.; Nouso, K.; Tsutsui, A.; Nagano, T.; Itokawa, N.; Hayama, K.; Imai, M.; Joko, K.; Tanaka, H.; Tamai, T.; Koizumi, Y.; Hiasa, Y.; Michitaka, K.; Kudo, M. | Important Clinical Factors in Sequential Therapy Including Lenvatinib against Unresectable Hepatocellular Carcinoma | 2019 |  | Oncology | 97 | 5 | 277-285 | 10.1159/000501281 |  |
| 314 | Hiraoka, A.; Kumada, T.; Kariyama, K.; Toyoda, H.; Yasuda, S.; Tsuji, K.; Hatanaka, T.; Kakizaki, S.; Naganuma, A.; Ishikawa, T.; Tada, T.; Takaguchi, K.; Itobayashi, E.; Shimada, N.; Shibata, H.; Tanaka, T.; Tsutsui, A.; Nagano, T.; Imai, M.; Nakamura, S.; Nouso, K. | Simple Scoring System for Predicting TACE Unsuitable among Intermediate-Stage Hepatocellular Carcinoma Patients in the Multiple Systemic Treatment Era | 2022 |  | Oncology | 100 | 2 | 65-73 | 10.1159/000520292 |  |
| 315 | Hiraoka, A.; Kumada, T.; Kariyama, K.; Toyoda, H.; Yasuda, S.; Tsuji, K.; Hatanaka, T.; Kakizaki, S.; Naganuma, A.; Ishikawa, T.; Tada, T.; Takaguchi, K.; Itobayashi, E.; Shimada, N.; Shibata, H.; Tanaka, T.; Tsutsui, A.; Nagano, T.; Imai, M.; Nakamura, S.; Nouso, K. | Simple Scoring System for Predicting TACE Unsuitable among Intermediate-Stage Hepatocellular Carcinoma Patients in the Multiple Systemic Treatment Era | 2022 |  | Oncology | 100 | 2 | 65-73 | 10.1159/000520292 |  |
| 316 | Hiraoka, A.; Kumada, T.; Kudo, M.; Hirooka, M.; Koizumi, Y.; Hiasa, Y.; Tajiri, K.; Toyoda, H.; Tada, T.; Ochi, H.; Joko, K.; Shimada, N.; Deguchi, A.; Ishikawa, T.; Imai, M.; Tsuji, K.; Michitaka, K. | Hepatic Function during Repeated TACE Procedures and Prognosis after Introducing Sorafenib in Patients with Unresectable Hepatocellular Carcinoma: Multicenter Analysis | 2017 |  | Dig Dis | 35 | 6 | 602-610 | 10.1159/000480256 |  |
| 317 | Hiraoka, A.; Kumada, T.; Michitaka, K.; Kudo, M. | Newly Proposed ALBI Grade and ALBI-T Score as Tools for Assessment of Hepatic Function and Prognosis in Hepatocellular Carcinoma Patients | 2019 | Oct | Liver Cancer | 8 | 5 | 312-325 | 10.1159/000494844 |  |
| 318 | Hiraoka, A.; Kumada, T.; Tada, T.; Hirooka, M.; Kariyama, K.; Tani, J.; Atsukawa, M.; Takaguchi, K.; Itobayashi, E.; Fukunishi, S.; Tsuji, K.; Ishikawa, T.; Tajiri, K.; Ochi, H.; Yasuda, S.; Toyoda, H.; Ogawa, C.; Nishimura, T.; Hatanaka, T.; Kakizaki, S.; Shimada, N.; Kawata, K.; Naganuma, A.; Tanaka, T.; Ohama, H.; Nouso, K.; Morishita, A.; Tsutsui, A.; Nagano, T.; Itokawa, N.; Okubo, T.; Arai, T.; Imai, M.; Koizumi, Y.; Nakamura, S.; Joko, K.; Iijima, H.; Hiasa, Y.; Kudo, M. | Early experience of atezolizumab plus bevacizumab treatment for unresectable hepatocellular carcinoma BCLC-B stage patients classified as beyond up to seven criteria - Multicenter analysis | 2022 | Mar | Hepatol Res | 52 | 3 | 308-316 | 10.1111/hepr.13734 |  |
| 319 | Hiraoka, A.; Kumada, T.; Tada, T.; Hirooka, M.; Kariyama, K.; Tani, J.; Atsukawa, M.; Takaguchi, K.; Itobayashi, E.; Fukunishi, S.; Tsuji, K.; Ishikawa, T.; Tajiri, K.; Ochi, H.; Yasuda, S.; Toyoda, H.; Ogawa, C.; Nishimura, T.; Hatanaka, T.; Kakizaki, S.; Shimada, N.; Kawata, K.; Naganuma, A.; Tanaka, T.; Ohama, H.; Nouso, K.; Morishita, A.; Tsutsui, A.; Nagano, T.; Itokawa, N.; Okubo, T.; Arai, T.; Imai, M.; Koizumi, Y.; Nakamura, S.; Joko, K.; Iijima, H.; Hiasa, Y.; Kudo, M. | Early experience of atezolizumab plus bevacizumab treatment for unresectable hepatocellular carcinoma BCLC-B stage patients classified as beyond up to seven criteria - Multicenter analysis | 2022 | Mar | Hepatol Res | 52 | 3 | 308-316 | 10.1111/hepr.13734 |  |
| 320 | Hiraoka, A.; Kumada, T.; Tada, T.; Hirooka, M.; Kariyama, K.; Tani, J.; Atsukawa, M.; Takaguchi, K.; Itobayashi, E.; Fukunishi, S.; Tsuji, K.; Ishikawa, T.; Tajiri, K.; Ochi, H.; Yasuda, S.; Toyoda, H.; Ogawa, C.; Nishimura, T.; Hatanaka, T.; Kakizaki, S.; Shimada, N.; Kawata, K.; Naganuma, A.; Kosaka, H.; Shibata, H.; Aoki, T.; Tanaka, T.; Ohama, H.; Nouso, K.; Morishita, A.; Tsutsui, A.; Nagano, T.; Itokawa, N.; Okubo, T.; Arai, T.; Imai, M.; Koizumi, Y.; Nakamura, S.; Joko, K.; Iijima, H.; Kaibori, M.; Hiasa, Y.; Kudo, M. | Does first-line treatment have prognostic impact for unresectable HCC?-Atezolizumab plus bevacizumab versus lenvatinib | 2023 | Jan | Cancer Med | 12 | 1 | 325-334 | 10.1002/cam4.4854 |  |
| 321 | Ho, L. M.; Lam, S. K.; Zhang, J.; Chiang, C. L.; Chan, A. C.; Cai, J. | Association of Multi-Phasic MR-Based Radiomic and Dosimetric Features with Treatment Response in Unresectable Hepatocellular Carcinoma Patients following Novel Sequential TACE-SBRT-Immunotherapy | 2023 | 9-Feb | Cancers (Basel) | 15 | 4 |  | 10.3390/cancers15041105 |  |
| 322 | Ho, Shu-Yein; Liu, Po-Hong; Hsu, Chia-Yang; Huang, Yi-Hsiang; Lei, Hao-Jan; Liao, Jia-I; Su, Chien-Wei; Hou, Ming-Chih; Huo, Teh-Ia | Surgical resection versus transarterial chemoembolization for patients with hepatocellular carcinoma beyond Milan criteria: prognostic role of tumor burden score | 2023 |  | Scientific reports | 13 | 1 | 13871 |  | Wrong patient population |
| 323 | Hong, S.; Choi, W. S.; Purushothaman, B.; Koh, J.; Kim, H. C.; Chung, J. W.; Song, J. M.; Choi, J. W. | Drug delivery in transarterial chemoembolization of hepatocellular carcinoma: Ex vivo evaluation using transparent tissue imaging | 2022 | Dec | Acta Biomater | 154 |  | 523-535 | 10.1016/j.actbio.2022.10.044 |  |
| 324 | Hou, Y. F.; Wei, Y. G.; Yang, J. Y.; Wen, T. F.; Xu, M. Q.; Yan, L. N.; Li, B. | Combined hepatectomy and radiofrequency ablation versus TACE in improving survival of patients with unresectable BCLC stage B HCC | 2016 | Aug | Hepatobiliary Pancreat Dis Int | 15 | 4 | 378-85 | 10.1016/s1499-3872(16)60089-9 |  |
| 325 | Hu, H.; Duan, Z.; Long, X.; Hertzanu, Y.; Shi, H.; Liu, S.; Yang, Z. | Sorafenib combined with transarterial chemoembolization versus transarterial chemoembolization alone for advanced-stage hepatocellular carcinoma: a propensity score matching study | 2014 |  | PLoS One | 9 | 5 | e96620 |  |  |
| 326 | Hu, K.; Lu, S.; Li, M.; Zhang, F.; Tang, B.; Yuan, J.; Shan, Y.; Xu, P.; Chen, R.; Ren, Z.; Yin, X. | A Novel Pre-treatment Model Predicting Risk of Developing Refractoriness to Transarterial Chemoembolization in Unresectable Hepatocellular Carcinoma | 2020 |  | J Cancer | 11 | 15 | 4589-4596 | 10.7150/jca.44847 |  |
| 327 | Hu, L.; Lin, J.; Shi, X.; Wang, A. | Efficacy of transarterial therapy combined with first-line tyrosine kinase inhibitors for unresectable hepatocellular carcinoma: a network meta-analysis | 2023 | 21-Jul | World J Surg Oncol | 21 | 1 | 208 | 10.1186/s12957-023-03098-3 |  |
| 328 | Hu, Y.; Pan, T.; Cai, X.; He, Q. S.; Zheng, Y. B.; Huang, M. S.; Jiang, Z. B.; Chen, J. W.; Wu, C. | Addition of transarterial chemoembolization improves outcome of tyrosine kinase and immune checkpoint inhibitors regime in patients with unresectable hepatocellular carcinoma | 2023 | 31-Aug | J Gastrointest Oncol | 14 | 4 | 1837-1848 | 10.21037/jgo-23-486 |  |
| 329 | Huang, D. J.; Li, Y. H.; Luo, Y. C.; Huang, J. Z.; He, H. Y. | Effective hepatic artery chemoembolization for advanced hepatocellular carcinoma with multiple tumor thrombi and pulmonary metastases: A case report | 2016 | Sep | Oncol Lett | 12 | 3 | 2103-2106 | 10.3892/ol.2016.4846 |  |
| 330 | Huang, D.; Chen, Y.; Chen, S.; Zeng, Q.; Zhao, J.; Wu, R.; Li, Y. | TACE plus percutaneous chemotherapy-lipiodol treatment of unresectable pedunculated hepatocellular carcinoma | 2017 | Jul | Medicine (Baltimore) | 96 | 30 | e7650 | 10.1097/md.0000000000007650 | Wrong patient population |
| 331 | Huang, J.; Huang, W.; Zhan, M.; Guo, Y.; Liang, L.; Cai, M.; Lin, L.; He, M.; Lian, H.; Lu, L.; Zhu, K. | Drug-Eluting Bead Transarterial Chemoembolization Combined with FOLFOX-Based Hepatic Arterial Infusion Chemotherapy for Large or Huge Hepatocellular Carcinoma | 2021 |  | J Hepatocell Carcinoma | 8 |  | 1445-1458 | 10.2147/jhc.S339379 | Wrong patient population |
| 332 | Huang, J.; Wang, Z. G.; Tao, Q. F.; Yang, Y.; Yuan, S. X.; Gu, F. M.; Liu, H.; Pan, Z. Y.; Jiang, B. G.; Lau, W. Y.; Zhou, W. P. | Efficacy and safety of Lenvatinib-based combination therapies for patients with unresectable hepatocellular carcinoma: a single center retrospective study | 2023 |  | Front Immunol | 14 |  | 1198562 | 10.3389/fimmu.2023.1198562 |  |
| 333 | Huang, K.; Zhou, Q.; Wang, R.; Cheng, D.; Ma, Y. | Doxorubicin-eluting beads versus conventional transarterial chemoembolization for the treatment of hepatocellular carcinoma | 2014 | May | J Gastroenterol Hepatol | 29 | 5 | 920-5 | 10.1111/jgh.12439 | Wrong patient population |
| 334 | Huang, M.; Lin, Q.; Wang, H.; Chen, J.; Bai, M.; Wang, L.; Zhu, K.; Jiang, Z.; Guan, S.; Li, Z.; Qian, J.; Li, M.; Pang, P.; Shan, H. | Survival benefit of chemoembolization plus Iodine125 seed implantation in unresectable hepatitis B-related hepatocellular carcinoma with PVTT: a retrospective matched cohort study | 2016 | Oct | Eur Radiol | 26 | 10 | 3428-36 | 10.1007/s00330-015-4198-x | Wrong patient population |
| 335 | Huang, T.; Qi, H.; Shen, L.; Wu, Y.; Song, Z.; Cao, F.; Liu, Y.; Xie, L.; Chen, S.; Tang, T.; Li, H.; Zhang, Y.; Feng, L.; Zhang, H.; Chen, J.; Fan, W. | Benefits of step-by-step debulking microwave ablation for huge unresectable hepatocellular carcinoma patients after transcatheter arterial chemoembolization refractoriness | 2022 |  | Int J Hyperthermia | 39 | 1 | 935-945 | 10.1080/02656736.2022.2093413 |  |
| 336 | Huang, Y. H.; Chen, W.; Li, J. P.; Chen, B.; Yang, J. Y. | Clinical value of continuous administration of sorafenib in combination with modified transarterial chemoembolization in patients with unresectable hepatocellular carcinoma | 2013 | Jan | Chin Med J (Engl) | 126 | 2 | 385-6 |  |  |
| 337 | Huang, Y. H.; Park, B. V.; Chen, Y. F.; Gaba, R. C.; Guzman, G.; Lokken, R. P. | Locoregional Therapy of Hepatocellular-Cholangiocarcinoma versus Hepatocellular Carcinoma: A Propensity Score-Matched Study | 2019 | Sep | J Vasc Interv Radiol | 30 | 9 | 1317-1324 | 10.1016/j.jvir.2019.03.024 |  |
| 338 | Huang, Y.; Chen, B.; Liu, N.; Li, N.; Dao, H.; Chen, W.; Yang, J. | Overall survival in response to sorafenib with transarterial chemoembolization for BCLC stage B hepatocellular carcinoma: propensity score analysis | 2017 | Jun | Int J Clin Pharmacol Ther | 55 | 6 | 498-508 | 10.5414/cp202787 | Wrong patient population |
| 339 | Huang, Y.; Cheng, X.; Sun, P.; Li, T.; Song, Z.; Zheng, Q. | Supplementary Sorafenib Therapies for Hepatocellular Carcinoma-A Systematic Review and Meta-Analysis: Supplementary Sorafenib for Liver Cancer | 2019 | Aug | J Clin Gastroenterol | 53 | 7 | 486-494 | 10.1097/mcg.0000000000001175 |  |
| 340 | Huang, Y.; Jia, Z.; Tu, J.; Shen, T.; Tian, F.; Jiang, G. | Supplemental conventional transarterial embolization/chemoembolization therapy via extrahepatic arteries for hepatocellular carcinoma | 2017 |  | J Cancer Res Ther | 13 | 4 | 720-724 | 10.4103/jcrt.JCRT_993_16 | Wrong patient population |
| 341 | Huang, Z. M.; Lai, C. X.; Zuo, M. X.; An, C.; Wang, X. C.; Huang, J. H.; Ning, E. | Adjuvant cytokine-induced killer cells with minimally invasive therapies augmented therapeutic efficacy of unresectable hepatocellular carcinoma | 2020 |  | J Cancer Res Ther | 16 | 7 | 1603-1610 | 10.4103/jcrt.JCRT_962_19 | Wrong patient population |
| 342 | Hui, Y.; Ruihua, T.; Jing, L.; Yaxiong, L.; Ji, L.; Linjie, Y.; Dongyan, S.; Mingliang, J.; Qingsheng, H.; Junling, S. | Meta-Analysis of Doxorubicin-Eluting Beads via Transcatheter Arterial Chemoembolization in the Treatment of Unresectable Hepatocellular Carcinoma | 2015 | Jun | Hepatogastroenterology | 62 | 140 | 1002-6 |  | Article inaccessible |
| 343 | Huo, Y. R.; Eslick, G. D. | Transcatheter Arterial Chemoembolization Plus Radiotherapy Compared With Chemoembolization Alone for Hepatocellular Carcinoma: A Systematic Review and Meta-analysis | 2015 | Sep | JAMA Oncol | 1 | 6 | 756-65 | 10.1001/jamaoncol.2015.2189 | Wrong patient population |
| 344 | Ichida, A.; Akamatsu, N.; Nagata, R.; Mihara, Y.; Kawaguchi, Y.; Bae, S. K.; Ishizawa, T.; Kaneko, J.; Arita, J.; Hasegawa, K. | Efficacy and Safety of Lenvatinib for the Treatment of Recurrent Hepatocellular Carcinoma After Living Donor Liver Transplantation: A Report of Two Cases | 2022 | Feb | Anticancer Res | 42 | 2 | 1161-1167 | 10.21873/anticanres.15581 |  |
| 345 | Ichida, A.; Akamatsu, N.; Nagata, R.; Mihara, Y.; Kawaguchi, Y.; Bae, S. K.; Ishizawa, T.; Kaneko, J.; Arita, J.; Hasegawa, K. | Efficacy and Safety of Lenvatinib for the Treatment of Recurrent Hepatocellular Carcinoma After Living Donor Liver Transplantation: A Report of Two Cases | 2022 | Feb | Anticancer Res | 42 | 2 | 1161-1167 | 10.21873/anticanres.15581 |  |
| 346 | Ichikawa, T.; Machida, N.; Sasaki, H.; Tenmoku, A.; Kaneko, H.; Negishi, R.; Oi, I.; Fujino, M. A. | Early Prediction of the Outcome Using Tumor Markers and mRECIST in Unresectable Hepatocellular Carcinoma Patients Who Underwent Transarterial Chemoembolization | 2016 |  | Oncology | 91 | 6 | 317-330 | 10.1159/000448999 |  |
| 347 | Idée, J. M.; Guiu, B. | Use of Lipiodol as a drug-delivery system for transcatheter arterial chemoembolization of hepatocellular carcinoma: a review | 2013 | Dec | Crit Rev Oncol Hematol | 88 | 3 | 530-49 | 10.1016/j.critrevonc.2013.07.003 |  |
| 348 | Iezzi, R.; Cesario, V.; Siciliani, L.; Campanale, M.; De Gaetano, A. M.; Siciliano, M.; Agnes, S.; Giuliante, F.; Grieco, A.; Pompili, M.; Rapaccini, G. L.; Gasbarrini, A.; Bonomo, L. | Single-step multimodal locoregional treatment for unresectable hepatocellular carcinoma: balloon-occluded percutaneous radiofrequency thermal ablation (BO-RFA) plus transcatheter arterial chemoembolization (TACE) | 2013 | Jun | Radiol Med | 118 | 4 | 555-69 | 10.1007/s11547-012-0914-7 |  |
| 349 | Iezzi, R.; Pompili, M.; Annicchiarico, E. B.; Garcovich, M.; Siciliano, M.; Gasbarrini, A.; Manfredi, R. | Hug sign': a new radiological sign of intraprocedural success after combined treatment for hepatocellular carcinoma | 2017 | Jul | Hepat Oncol | 4 | 3 | 69-73 | 10.2217/hep-2017-0017 |  |
| 350 | Iezzi, R.; Posa, A.; Santoro, M.; Tanzilli, A.; Cerrito, L.; Ponziani, F. R.; Pompili, M.; Grieco, A.; Rapaccini, G. L.; Gasbarrini, A.; Manfredi, R. | Balloon-Occluded Radiofrequency Ablation as Bridge to TACE in the Treatment of Advanced HCC with Arterioportal Shunt | 2022 | 23-Feb | Curr Radiopharm | |  |  | 10.2174/1874471015666220223102426 |  |
| 351 | Iezzi, R.; Posa, A.; Santoro, M.; Tanzilli, A.; Cerrito, L.; Ponziani, F. R.; Pompili, M.; Grieco, A.; Rapaccini, G. L.; Gasbarrini, A.; Manfredi, R. | Balloon-Occluded Radiofrequency Ablation as Bridge to TACE in the Treatment of Advanced HCC with Arterioportal Shunt | 2022 |  | Curr Radiopharm | 15 | 3 | 194-198 | 10.2174/1874471015666220223102426 |  |
| 352 | Iezzi, R.; Posa, A.; Tanzilli, A.; Carchesio, F.; Pompili, M.; Manfredi, R. | Balloon-Occluded MWA (b-MWA) Followed by Balloon-Occluded TACE (b-TACE): Technical Note on a New Combined Single-Step Therapy for Single Large HCC | 2020 | Nov | Cardiovasc Intervent Radiol | 43 | 11 | 1702-1707 | 10.1007/s00270-020-02583-6 | Wrong patient population |
| 353 | Ikeda, M.; Arai, Y.; Park, S. J.; Takeuchi, Y.; Anai, H.; Kim, J. K.; Inaba, Y.; Aramaki, T.; Kwon, S. H.; Yamamoto, S.; Okusaka, T. | Prospective study of transcatheter arterial chemoembolization for unresectable hepatocellular carcinoma: an Asian cooperative study between Japan and Korea | 2013 | Apr | J Vasc Interv Radiol | 24 | 4 | 490-500 | 10.1016/j.jvir.2013.01.003 | Wrong patient population |
| 354 | Ikeda, M.; Kudo, M.; Aikata, H.; Nagamatsu, H.; Ishii, H.; Yokosuka, O.; Torimura, T.; Morimoto, M.; Ikeda, K; Kumada, H. | Transarterial chemoembolization with miriplatin vs. epirubicin for unresectable hepatocellular carcinoma: a phase III randomized trial | 2018 |  | Journal of gastroenterology | 53 | 2 | 281-290 |  | Wrong patient population |
| 355 | Ilagan, C. H.; Goldman, D. A.; Gönen, M.; Aveson, V. G.; Babicky, M.; Balachandran, V. P.; Drebin, J. A.; Jarnagin, W. R.; Wei, A. C.; Kingham, T. P. | Recurrence of hepatocellular carcinoma after complete radiologic response to trans-arterial embolization: A retrospective study on patterns, treatments, and prognoses | 2022 |  | Annals of Surgical Oncology | 29 | 11 | 6815-6826 |  |  |
| 356 | Imai, N.; Ikeda, K.; Kawamura, Y.; Sezaki, H.; Hosaka, T.; Akuta, N.; Kobayashi, M.; Saitoh, S.; Suzuki, F.; Suzuki, Y.; Arase, Y.; Kumada, H. | Transcatheter arterial chemotherapy using miriplatin-lipiodol suspension with or without embolization for unresectable hepatocellular carcinoma | 2012 | Mar | Jpn J Clin Oncol | 42 | 3 | 175-82 | 10.1093/jjco/hyr189 |  |
| 357 | Imai, N.; Yokoyama, S.; Yamamoto, K.; Ito, T.; Ishizu, Y.; Honda, T.; Ishigami, M. | Safety and Efficacy of Glass Membrane Pumping Emulsification Device in Transarterial Chemoembolization for Hepatocellular Carcinoma: First Clinical Outcomes | 2021 | Nov | Anticancer Res | 41 | 11 | 5817-5820 | 10.21873/anticanres.15399 |  |
| 358 | Imai, Y.; Hirooka, M.; Koizumi, Y.; Nakamura, Y.; Watanabe, T.; Yoshida, O.; Tokumoto, Y.; Takeshita, E.; Abe, M.; Tanaka, H.; Kurata, M.; Kitazawa, S.; Hiasa, Y. | Histological assessment of the efficacy of drug-eluting beads in portal tumor thrombosis of hepatocellular carcinoma | 2017 | Mar | Radiol Case Rep | 12 | 1 | 179-184 | 10.1016/j.radcr.2016.11.006 |  |
| 359 | Imamura, A.; Taguchi, H.; Takano, H.; Funatsu, H.; Nakamura, K.; Arimitsu, H.; Chiba, S. | Whole-liver transcatheter arterial chemoinfusion and bland embolization with fine-powder cisplatin and trisacryl gelatin microspheres for treating unresectable multiple hepatocellular carcinoma | 2021 | May | Jpn J Radiol | 39 | 5 | 494-502 | 10.1007/s11604-020-01078-1 | Wrong patient population |
| 360 | Ishikawa, T.; Imai, M.; Sato, R.; Jimbo, R.; Kobayashi, Y.; Sato, T.; Iwanaga, A.; Sano, T.; Yokoyama, J.; Honma, T. | Prognostic Value of TACE With Irinotecan-loaded Drug-eluting Beads (DEBIRI) in Patients With Liver Metastases from Unresectable Colorectal Cancer | 2023 | Aug | Anticancer Res | 43 | 8 | 3647-3651 | 10.21873/anticanres.16545 |  |
| 361 | Ishizaki, M.; Kaibori, M.; Matsushima, H.; Kosaka, H.; Matsui, K.; Sekimoto, M. | Long-term complete response to lenvatinib in a patient with unresectable hepatocellular carcinoma | 2021 | Dec | Clin J Gastroenterol | 14 | 6 | 1700-1705 | 10.1007/s12328-021-01506-3 |  |
| 362 | Iwazawa, J.; Hashimoto, N.; Ohue, S.; Mitani, T. | Initial safety and outcomes of miriplatin plus low-dose epirubicin for transarterial chemoembolisation of hepatocellular carcinoma | 2012 | Nov | Anticancer Res | 32 | 11 | 5039-44 |  | Wrong patient population |
| 363 | Iwazawa, J.; Ohue, S.; Hashimoto, N.; Muramoto, O.; Mitani, T. | Survival after C-arm CT-assisted chemoembolization of unresectable hepatocellular carcinoma | 2012 | Dec | Eur J Radiol | 81 | 12 | 3985-92 | 10.1016/j.ejrad.2012.08.012 | Wrong patient population |
| 364 | Jang, E. S.; Yoon, J. H.; Chung, J. W.; Cho, E. J.; Yu, S. J.; Lee, J. H.; Kim, Y. J.; Lee, H. S.; Kim, C. Y. | Survival of infiltrative hepatocellular carcinoma patients with preserved hepatic function after treatment with transarterial chemoembolization | 2013 | Apr | J Cancer Res Clin Oncol | 139 | 4 | 635-43 | 10.1007/s00432-012-1364-2 | Wrong patient population |
| 365 | Jazieh, K. A.; Arabi, M.; Khankan, A. A. | Transarterial therapy: an evolving treatment modality of hepatocellular carcinoma | 2014 | Nov-Dec | Saudi J Gastroenterol | 20 | 6 | 333-41 | 10.4103/1319-3767.145315 |  |
| 366 | Ji, J.; Gu, J.; Wu, J. Z.; Yang, W.; Shi, H. B.; Liu, S.; Zhou, W. Z. | The "Six-and-Twelve" Score for Recurrent HCC Patients Receiving TACE: Does it Still Work? | 2021 | May | Cardiovasc Intervent Radiol | 44 | 5 | 720-727 | 10.1007/s00270-021-02791-8 |  |
| 367 | Jia, K. F.; Wang, H.; Yu, C. L.; Yin, W. L.; Zhang, X. D.; Wang, F.; Sun, C.; Shen, W. | ASARA, a prediction model based on Child-Pugh class in hepatocellular carcinoma patients undergoing transarterial chemoembolization | 2023 | Oct | Hepatobiliary Pancreat Dis Int | 22 | 5 | 490-497 | 10.1016/j.hbpd.2022.02.007 | Wrong outcomes |
| 368 | Jia, K. F.; Wang, H.; Yu, C. L.; Yin, W. L.; Zhang, X. D.; Wang, F.; Sun, C.; Shen, W. | ASARA, a prediction model based on Child-Pugh class in hepatocellular carcinoma patients undergoing transarterial chemoembolization | 2022 | 25-Feb | Hepatobiliary Pancreat Dis Int | | |  | 10.1016/j.hbpd.2022.02.007 |  |
| 369 | Jiang, G.; Ling, S.; Zhan, Q.; Zhuang, L.; Xu, X. | Downstaging treatment for patients with hepatocelluar carcinoma before transplantation | 2021 | Apr | Transplant Rev (Orlando) | 35 | 2 | 100606 | 10.1016/j.trre.2021.100606 |  |
| 370 | Jiang, J.; Diaz, D. A.; Nuguru, S. P.; Mittra, A.; Manne, A. | Stereotactic Body Radiation Therapy (SBRT) Plus Immune Checkpoint Inhibitors (ICI) in Hepatocellular Carcinoma and Cholangiocarcinoma | 2022 | 22-Dec | Cancers (Basel) | 15 | 1 |  | 10.3390/cancers15010050 |  |
| 371 | Jiang, N.; Zhong, B.; Huang, J.; Li, W.; Zhang, S.; Zhu, X.; Ni, C.; Shen, J. | Transarterial chemoembolization combined with molecularly targeted agents plus immune checkpoint inhibitors for unresectable hepatocellular carcinoma: a retrospective cohort study | 2023 |  | Front Immunol | 14 |  | 1205636 | 10.3389/fimmu.2023.1205636 |  |
| 372 | Jiang, T.; Zeng, Z. C.; Yang, P.; Hu, Y. | Exploration of Superior Modality: Safety and Efficacy of Hypofractioned Image-Guided Intensity Modulated Radiation Therapy in Patients with Unresectable but Confined Intrahepatic Hepatocellular Carcinoma | 2017 |  | Can J Gastroenterol Hepatol | 2017 |  | 6267981 | 10.1155/2017/6267981 |  |
| 373 | Jiang, W.; Zeng, Z. C. | Is it time to adopt external beam radiotherapy in the NCCN guidelines as a therapeutic strategy for intermediate/advanced hepatocellular carcinoma? | 2013 |  | Oncology | 84 Suppl 1 |  | 69-74 | 10.1159/000345893 |  |
| 374 | Jin, P. P.; Shao, S. Y.; Wu, W. T.; Zhao, X. Y.; Huang, B. F.; Fu, Q. H.; Que, R. S.; Hu, Q. D. | Combination of transarterial chemoembolization and sorafenib improves outcomes of unresectable hepatocellular carcinoma: an updated systematic review and meta-analysis | 2018 | 1-Dec | Jpn J Clin Oncol | 48 | 12 | 1058-1069 | 10.1093/jjco/hyy138 | Wrong patient population |
| 375 | Jindal, A.; Thadi, A.; Shailubhai, K. | Hepatocellular Carcinoma: Etiology and Current and Future Drugs | 2019 | Mar-Apr | J Clin Exp Hepatol | 9 | 2 | 221-232 | 10.1016/j.jceh.2019.01.004 |  |
| 376 | Ju, S.; Zhou, C.; Hu, J.; Wang, Y.; Wang, C.; Liu, J.; Yang, C.; Huang, S.; Li, T.; Chen, Y.; Bai, Y.; Yao, W.; Xiong, B. | Late combination of transarterial chemoembolization with apatinib and camrelizumab for unresectable hepatocellular carcinoma is superior to early combination | 2022 | 27-Mar | BMC Cancer | 22 | 1 | 335 | 10.1186/s12885-022-09451-1 |  |
| 377 | Ju, S.; Zhou, C.; Yang, C.; Wang, C.; Liu, J.; Wang, Y.; Huang, S.; Li, T.; Chen, Y.; Bai, Y.; Yao, W.; Xiong, B. | Apatinib Plus Camrelizumab With/Without Chemoembolization for Hepatocellular Carcinoma: A Real-World Experience of a Single Center | 2021 |  | Front Oncol | 11 |  | 835889 | 10.3389/fonc.2021.835889 | Wrong patient population |
| 378 | Ju, S.; Zhou, C.; Yang, C.; Wang, C.; Liu, J.; Wang, Y.; Huang, S.; Li, T.; Chen, Y.; Bai, Y.; Yao, W.; Xiong, B. | Apatinib Plus Camrelizumab With/Without Chemoembolization for Hepatocellular Carcinoma: A Real-World Experience of a Single Center | 2021 |  | Front Oncol | 11 |  | 835889 | 10.3389/fonc.2021.835889 |  |
| 379 | Jung, E.; Shin, J. H.; Kim, J. H.; Yoon, H. K.; Ko, G. Y.; Sung, K. B. | Arterial dissections during transcatheter arterial chemoembolization for hepatocellular carcinoma: a 19-year clinical experience at a single medical institution | 2017 | Jul | Acta Radiol | 58 | 7 | 842-848 | 10.1177/0284185116676654 |  |
| 380 | Kaewdech, A.; Sripongpun, P.; Assawasuwannakit, S.; Wetwittayakhlang, P.; Jandee, S.; Chamroonkul, N.; Piratvisuth, T. | FAIL-T (AFP, AST, tumor sIze, ALT, and Tumor number): a model to predict intermediate-stage HCC patients who are not good candidates for TACE | 2023 |  | Front Med (Lausanne) | 10 |  | 1077842 | 10.3389/fmed.2023.1077842 |  |
| 381 | Kaewdech, A.; Sripongpun, P.; Cheewasereechon, N.; Jandee, S.; Chamroonkul, N.; Piratvisuth, T. | Validation of the "Six-and-Twelve" Prognostic Score in Transarterial Chemoembolization-Treated Hepatocellular Carcinoma Patients | 2021 | 18-Feb | Clin Transl Gastroenterol | 12 | 2 | e00310 | 10.14309/ctg.0000000000000310 |  |
| 382 | Kallini, J. R.; Gabr, A.; Salem, R.; Lewandowski, R. J. | Transarterial Radioembolization with Yttrium-90 for the Treatment of Hepatocellular Carcinoma | 2016 | May | Adv Ther | 33 | 5 | 699-714 | 10.1007/s12325-016-0324-7 |  |
| 383 | Kalra, N.; Mahajan, D.; Chawla, Y.; Khandelwal, N. | Selective doxorubicin drug eluting beads chemoembolization of hypovascular hepatocellular carcinoma using cone beam computed tomography | 2012 | Oct | Indian J Radiol Imaging | 22 | 4 | 254-6 | 10.4103/0971-3026.111472 |  |
| 384 | Kamimura, K.; Suda, T.; Tamura, Y.; Takamura, M.; Yokoo, T.; Igarashi, M.; Kawai, H.; Yamagiwa, S.; Nomoto, M.; Aoyagi, Y. | Phase I study of miriplatin combined with transarterial chemotherapy using CDDP powder in patients with hepatocellular carcinoma | 2012 | 20-Sep | BMC Gastroenterol | 12 |  | 127 | 10.1186/1471-230x-12-127 |  |
| 385 | Kamran, A. U.; Liu, Y.; Li, F. E.; Liu, S.; Wu, J. L.; Zhang, Y. W. | Transcatheter Arterial Chemoembolization With Gelatin Sponge Microparticles Treated for BCLC Stage B Hepatocellular Carcinoma: A Single Center Retrospective Study | 2015 | Dec | Medicine (Baltimore) | 94 | 52 | e2154 | 10.1097/md.0000000000002154 |  |
| 386 | Kao, S.; Sung, K. | Editorial for "Predicting the Outcome of Transcatheter Arterial Embolization Therapy for Unresectable Hepatocellular Carcinoma Based on Radiomics of Preoperative Multiparameter MRI" | 2020 | Oct | J Magn Reson Imaging | 52 | 4 | 1091-1092 | 10.1002/jmri.27166 |  |
| 387 | Kaplan, D. E.; Mehta, R.; D'Addeo, K.; Gade, T. P.; Taddei, T. H. | Transarterial Chemoembolization within First 3 Months of Sorafenib Initiation Improves Overall Survival in Hepatocellular Carcinoma: A Retrospective, Multi-Institutional Study with Propensity Matching | 2018 | Apr | J Vasc Interv Radiol | 29 | 4 | 540-549.e4 | 10.1016/j.jvir.2017.11.033 | Wrong patient population |
| 388 | Karanicolas, P.; Beecroft, J. R.; Cosby, R.; David, E.; Kalyvas, M.; Kennedy, E.; Sapisochin, G.; Wong, R.; Zbuk, K. | Regional Therapies for Colorectal Liver Metastases: Systematic Review and Clinical Practice Guideline | 2021 | Mar | Clin Colorectal Cancer | 20 | 1 | 20-28 | 10.1016/j.clcc.2020.09.008 |  |
| 389 | Kashfi, S.; Murdakhayev, E.; Rehmani, R.; Sharma, S. | Post-Embolization Syndrome Complicated by Hypertensive Emergency and Severely Elevated Transaminases | 2021 | Jun | Cureus | 13 | 6 | e15446 | 10.7759/cureus.15446 |  |
| 390 | Katayama, K.; Ohkawa, K.; Imanaka, K.; Sakakibara, M.; Miyazaki, M.; Kimura, H.; Ishihara, A.; Matsunaga, T.; Murata, M.; Nakazawa, T.; Nakanishi, K. | Computed tomography during hepatic arteriography pattern may predict hepatocellular carcinoma recurrence following transarterial chemoembolization | 2014 | Dec | Hepatol Res | 44 | 14 | E455-63 | 10.1111/hepr.12337 |  |
| 391 | Katsanos, K.; Kitrou, P.; Spiliopoulos, S.; Maroulis, I.; Petsas, T.; Karnabatidis, D. | Comparative effectiveness of different transarterial embolization therapies alone or in combination with local ablative or adjuvant systemic treatments for unresectable hepatocellular carcinoma: A network meta-analysis of randomized controlled trials | 2017 |  | PLoS One | 12 | 9 | e0184597 | 10.1371/journal.pone.0184597 | Wrong patient population |
| 392 | Kawabe, N.; Hashimoto, S.; Nakano, T.; Nakaoka, K.; Fukui, A.; Yoshioka, K. | Transcatheter arterial infusion chemotherapy with cisplatin in combination with transcatheter arterial chemoembolization decreases intrahepatic distant recurrence of unresectable hepatocellular carcinoma | 2021 | Jun | JGH Open | 5 | 6 | 705-711 | 10.1002/jgh3.12573 |  |
| 393 | Kawamura, Y.; Akuta, N.; Shindoh, J.; Matsumura, M.; Okubo, S.; Tominaga, L.; Fujiyama, S.; Hosaka, T.; Saitoh, S.; Sezaki, H.; Suzuki, F.; Suzuki, Y.; Ikeda, K.; Arase, Y.; Hashimoto, M.; Kozuka, T.; Kumada, H. | Well-preserved liver function enhances the clinical impact of curative-intent subsequent treatment during lenvatinib treatment for unresectable hepatocellular carcinoma | 2023 | Feb | Clin J Gastroenterol | 16 | 1 | 12-Jan | 10.1007/s12328-022-01723-4 |  |
| 394 | Kawamura, Y.; Kobayashi, M.; Shindoh, J.; Kobayashi, Y.; Okubo, S.; Tominaga, L.; Kajiwara, A.; Kasuya, K.; Iritani, S.; Fujiyama, S.; Hosaka, T.; Saitoh, S.; Sezaki, H.; Akuta, N.; Suzuki, F.; Suzuki, Y.; Ikeda, K.; Arase, Y.; Hashimoto, M.; Kozuka, T.; Kumada, H. | Lenvatinib-Transarterial Chemoembolization Sequential Therapy as an Effective Treatment at Progression during Lenvatinib Therapy for Advanced Hepatocellular Carcinoma | 2020 | Dec | Liver Cancer | 9 | 6 | 756-770 | 10.1159/000510299 |  |
| 395 | Kawaoka, T.; Aikata, H.; Hyogo, H.; Morio, R.; Morio, K.; Hatooka, M.; Fukuhara, T.; Kobayashi, T.; Naeshiro, N.; Miyaki, D.; Hiramatsu, A.; Imamura, M.; Kawakami, Y.; Takahashi, S.; Waki, K.; Tsuji, K.; Kohno, H.; Kohno, H.; Moriya, T.; Chayama, K. | Comparison of hepatic arterial infusion chemotherapy versus sorafenib monotherapy in patients with advanced hepatocellular carcinoma | 2015 | Sep | J Dig Dis | 16 | 9 | 505-12 | 10.1111/1751-2980.12267 |  |
| 396 | Ke, L.; Shen, R.; Fan, W.; Hu, W.; Shen, S.; Li, S.; Kuang, M.; Liang, L.; Li, J.; Peng, B.; Hua, Y. | The role of associating liver partition and portal vein ligation for staged hepatectomy in unresectable hepatitis B virus-related hepatocellular carcinoma | 2020 | Nov | Ann Transl Med | 8 | 21 | 1402 | 10.21037/atm-20-2420 |  |
| 397 | Ke, Q.; Xin, F.; Fang, H.; Zeng, Y.; Wang, L.; Liu, J. | Corrigendum: The Significance of Transarterial Chemo(embolization) Combined With Tyrosine Kinase Inhibitors and Immune Check Point Inhibitors for Unresectable Hepatocellular Carcinoma in the Era of Systemic Therapy: A Systematic Review | 2022 |  | Front Immunol | 13 |  | 952446 | 10.3389/fimmu.2022.952446 |  |
| 398 | Ke, Q.; Xin, F.; Fang, H.; Zeng, Y.; Wang, L.; Liu, J. | The Significance of Transarterial Chemo(Embolization) Combined With Tyrosine Kinase Inhibitors and Immune Checkpoint Inhibitors for Unresectable Hepatocellular Carcinoma in the Era of Systemic Therapy: A Systematic Review | 2022 |  | Front Immunol | 13 |  | 913464 | 10.3389/fimmu.2022.913464 |  |
| 399 | Keane, F. K.; Tanguturi, S. K.; Zhu, A. X.; Dawson, L. A.; Hong, T. S. | Radiotherapy for liver tumors | 2015 | Apr | Hepat Oncol | 2 | 2 | 133-146 | 10.2217/hep.15.7 |  |
| 400 | Kennedy, A. S.; Sangro, B. | Nonsurgical treatment for localized hepatocellular carcinoma | 2014 | Mar | Curr Oncol Rep | 16 | 3 | 373 | 10.1007/s11912-013-0373-x | Wrong patient population |
| 401 | Khalid, M. A.; Achakzai, I. K.; Hanif, F. M.; Ahmed, S.; Majid, Z.; Luck, N. H. | To determine the prognostic value of the albumin-bilirubin grade (ALBI) in patients underwent transarterial chemoembolization for unresectable hepatocellular carcinoma | 2019 | Spring | Gastroenterol Hepatol Bed Bench | 12 | 2 | 110-115 |  |  |
| 402 | Khan, A. R.; Wei, X.; Xu, X. | Portal Vein Tumor Thrombosis and Hepatocellular Carcinoma - The Changing Tides | 2021 |  | J Hepatocell Carcinoma | 8 |  | 1089-1115 | 10.2147/jhc.S318070 |  |
| 403 | Kim, D. Y.; Han, K. H. | Transarterial chemoembolization versus transarterial radioembolization in hepatocellular carcinoma: optimization of selecting treatment modality | 2016 | Nov | Hepatol Int | 10 | 6 | 883-892 | 10.1007/s12072-016-9722-9 |  |
| 404 | Kim, D. Y.; Ryu, H. J.; Choi, J. Y.; Park, J. Y.; Lee, D. Y.; Kim, B. K.; Kim, S. U.; Ahn, S. H.; Chon, C. Y.; Han, K. H. | Radiological response predicts survival following transarterial chemoembolisation in patients with unresectable hepatocellular carcinoma | 2012 | Jun | Aliment Pharmacol Ther | 35 | 11 | 1343-50 | 10.1111/j.1365-2036.2012.05089.x |  |
| 405 | Kim, H. C. | Radioembolization for the treatment of hepatocellular carcinoma | 2017 | Jun | Clin Mol Hepatol | 23 | 2 | 109-114 | 10.3350/cmh.2017.0004 |  |
| 406 | Kim, H. C.; Miyayama, S.; Chung, J. W. | Selective Chemoembolization of Caudate Lobe Hepatocellular Carcinoma: Anatomy and Procedural Techniques | 2019 | Jan-Feb | Radiographics | 39 | 1 | 289-302 | 10.1148/rg.2019180110 |  |
| 407 | Kim, H. D.; An, J.; Kim, J. H.; Gwon, D. I.; Shin, J. H.; Ko, G. Y.; Yoon, H. K.; Sung, K. B.; Kim, K. M.; Lee, H. C. | Impact of the Interval between Transarterial Chemoembolization Sessions on Survival in Patients with Unresectable Hepatocellular Carcinoma | 2016 | Apr | J Vasc Interv Radiol | 27 | 4 | 504-13 | 10.1016/j.jvir.2015.12.005 |  |
| 408 | Kim, H.; Yu, S. J.; Yeo, I.; Cho, Y. Y.; Lee, D. H.; Cho, Y.; Cho, E. J.; Lee, J. H.; Kim, Y. J.; Lee, S.; Jun, J.; Park, T.; Yoon, J. H.; Kim, Y. | Prediction of Response to Sorafenib in Hepatocellular Carcinoma: A Putative Marker Panel by Multiple Reaction Monitoring-Mass Spectrometry (MRM-MS) | 2017 | Jul | Mol Cell Proteomics | 16 | 7 | 1312-1323 | 10.1074/mcp.M116.066704 |  |
| 409 | Kim, J. W.; Seong, J.; Yun, M.; Lee, I. J.; Yoon, H. I.; Cho, H. J.; Han, K. H. | Usefulness of positron emission tomography with fluorine-18-fluorodeoxyglucose in predicting treatment response in unresectable hepatocellular carcinoma patients treated with external beam radiotherapy | 2012 | 1-Mar | Int J Radiat Oncol Biol Phys | 82 | 3 | 1172-8 | 10.1016/j.ijrobp.2010.11.076 |  |
| 410 | Kim, Jong Woo; Kim, Jin Hyoung; Won, Hyung Jin; Shin, Yong Moon; Yoon, Hyun-Ki; Sung, Kyu-Bo; Kim, Pyo Nyun | Hepatocellular carcinomas 2"“3cm in diameter: Transarterial chemoembolization plus radiofrequency ablation vs. radiofrequency ablation alone | 2012 |  | European Journal of Radiology | 81 | 3 | e189-e193 | <https://doi.org/10.1016/j.ejrad.2011.01.122> | Wrong intervention |
| 411 | Kim, K. H.; Kim, M. S.; Chang, J. S.; Han, K. H.; Kim, D. Y.; Seong, J. | Therapeutic benefit of radiotherapy in huge (≥ 10 cm) unresectable hepatocellular carcinoma | 2014 | May | Liver Int | 34 | 5 | 784-94 | 10.1111/liv.12436 | Wrong patient population |
| 412 | Kim, N.; Cheng, J.; Jung, I.; Liang, J.; Shih, Y. L.; Huang, W. Y.; Kimura, T.; Lee, V. H. F.; Zeng, Z. C.; Zhenggan, R.; Kay, C. S.; Heo, S. J.; Won, J. Y.; Seong, J. | Stereotactic body radiation therapy vs. radiofrequency ablation in Asian patients with hepatocellular carcinoma | 2020 | Jul | J Hepatol | 73 | 1 | 121-129 | 10.1016/j.jhep.2020.03.005 |  |
| 413 | Kim, S. I.; Jin, Y. J.; Cho, S. G.; Shin, W. Y.; Kim, J. M.; Lee, J. W. | Duodenal perforation and esophageal ischemia following transarterial chemoembolization for hepatocellular carcinoma: A case report | 2016 | Jul | Medicine (Baltimore) | 95 | 27 | e3987 | 10.1097/md.0000000000003987 |  |
| 414 | Kimura, Y.; Kaneko, R.; Yano, Y.; Kamada, K.; Ikehara, T.; Nagai, H.; Sato, Y.; Igarashi, Y. | The Prognosis of Hepatocellular Carcinoma Treated with Sorafenib in Combination with TACE | 2020 | 1-Jun | Asian Pac J Cancer Prev | 21 | 6 | 1797-1805 | 10.31557/apjcp.2020.21.6.1797 |  |
| 415 | Kirchner, T.; Marquardt, S.; Werncke, T.; Kirstein, M. M.; Brunkhorst, T.; Wacker, F.; Vogel, A.; Rodt, T. | Comparison of health-related quality of life after transarterial chemoembolization and transarterial radioembolization in patients with unresectable hepatocellular carcinoma | 2019 | Apr | Abdom Radiol (NY) | 44 | 4 | 1554-1561 | 10.1007/s00261-018-1802-y |  |
| 416 | Kirstein, M. M.; Scheiner, B.; Pinter, M.; Vogel, A. | Letter: sequential or combined systemic treatment for unresectable hepatocellular carcinoma-authors' reply | 2020 | Sep | Aliment Pharmacol Ther | 52 | 5 | 917-918 | 10.1111/apt.15980 |  |
| 417 | Kirstein, M. M.; Schweitzer, N.; Schmidt, S.; Klöpper, A.; Ringe, K. I.; Lehmann, U.; Manns, M. P.; Wedemeyer, H.; Vogel, A. | Long-lasting tumour response to sorafenib therapy in advanced hepatocellular carcinoma | 2014 | Dec | Acta Gastroenterol Belg | 77 | 4 | 386-8 |  |  |
| 418 | Kishi, Y.; Shimada, K.; Nara, S.; Esaki, M.; Kosuge, T. | Role of hepatectomy for recurrent or initially unresectable hepatocellular carcinoma | 2014 | 27-Dec | World J Hepatol | 6 | 12 | 836-43 | 10.4254/wjh.v6.i12.836 |  |
| 419 | Kishore, S. A.; Bajwa, R.; Madoff, D. C. | Embolotherapeutic Strategies for Hepatocellular Carcinoma: 2020 Update | 2020 | 26-Mar | Cancers (Basel) | 12 | 4 |  | 10.3390/cancers12040791 | Wrong patient population |
| 420 | Kishore, S.; Friedman, T.; Madoff, D. C. | Update on Embolization Therapies for Hepatocellular Carcinoma | 2017 | Jun | Curr Oncol Rep | 19 | 6 | 40 | 10.1007/s11912-017-0597-2 |  |
| 421 | Kloeckner, R.; Weinmann, A.; Prinz, F.; Pinto dos Santos, D.; Ruckes, C.; Dueber, C.; Pitton, M. B. | Conventional transarterial chemoembolization versus drug-eluting bead transarterial chemoembolization for the treatment of hepatocellular carcinoma | 2015 | 10-Jun | BMC Cancer | 15 |  | 465 | 10.1186/s12885-015-1480-x | Wrong patient population |
| 422 | Klompenhouwer, E. G.; Dresen, R. C.; Verslype, C.; Laenen, A.; Bonne, L.; Vandecaveye, V.; Maleux, G. | Transarterial Radioembolization Following Chemoembolization for Unresectable Hepatocellular Carcinoma: Response Based on Apparent Diffusion Coefficient Change is an Independent Predictor for Survival | 2018 | Nov | Cardiovasc Intervent Radiol | 41 | 11 | 1716-1726 | 10.1007/s00270-018-1991-3 |  |
| 423 | Klompenhouwer, E. G.; Dresen, R. C.; Verslype, C.; Laenen, A.; De Hertogh, G.; Deroose, C. M.; Bonne, L.; Vandevaveye, V.; Maleux, G. | Safety and Efficacy of Transarterial Radioembolisation in Patients with Intermediate or Advanced Stage Hepatocellular Carcinoma Refractory to Chemoembolisation | 2017 | Dec | Cardiovasc Intervent Radiol | 40 | 12 | 1882-1890 | 10.1007/s00270-017-1739-5 |  |
| 424 | Ko, K. L.; Mak, L. Y.; Cheung, K. S.; Yuen, M. F. | Hepatocellular carcinoma: recent advances and emerging medical therapies | 2020 |  | F1000Res | 9 |  |  | 10.12688/f1000research.24543.1 |  |
| 425 | Kodama, K.; Kawaoka, T.; Aikata, H.; Uchikawa, S.; Inagaki, Y.; Hatooka, M.; Morio, K.; Nakahara, T.; Murakami, E.; Tsuge, M.; Hiramatsu, A.; Imamura, M.; Kawakami, Y.; Masaki, K.; Honda, Y.; Mori, N.; Takaki, S.; Tsuji, K.; Kohno, H.; Kohno, H.; Moriya, T.; Nonaka, M.; Hyogo, H.; Aisaka, Y.; Chayama, K. | Comparison of clinical outcome of hepatic arterial infusion chemotherapy and sorafenib for advanced hepatocellular carcinoma according to macrovascular invasion and transcatheter arterial chemoembolization refractory status | 2018 | Oct | J Gastroenterol Hepatol | 33 | 10 | 1780-1786 | 10.1111/jgh.14152 |  |
| 426 | Koga, H.; Iwamoto, H.; Suzuki, H.; Shimose, S.; Nakano, M.; Kawaguchi, T. | Clinical practice guidelines and real-life practice in hepatocellular carcinoma: A Japanese perspective | 2023 | Apr | Clin Mol Hepatol | 29 | 2 | 242-251 | 10.3350/cmh.2023.0102 |  |
| 427 | Kohorst, M. A.; Warad, D. M.; Matsumoto, J. M.; Heimbach, J. K.; El-Youssef, M.; Arndt, C. A. S.; Rodriguez, V.; Nageswara Rao, A. A. | Management of pediatric hepatocellular carcinoma: A multimodal approach | 2017 | Sep | Pediatr Transplant | 21 | 6 |  | 10.1111/petr.13007 |  |
| 428 | Kokabi, N.; Camacho, J. C.; Xing, M.; Edalat, F.; Mittal, P. K.; Kim, H. S. | Immediate post-doxorubicin drug-eluting beads chemoembolization Mr Apparent diffusion coefficient quantification predicts response in unresectable hepatocellular carcinoma: A pilot study | 2015 | Oct | J Magn Reson Imaging | 42 | 4 | 981-9 | 10.1002/jmri.24845 |  |
| 429 | Kokabi, N.; Ludwig, J. M.; Camacho, J. C.; Xing, M.; Mittal, P. K.; Kim, H. S. | Baseline and Early MR Apparent Diffusion Coefficient Quantification as a Predictor of Response of Unresectable Hepatocellular Carcinoma to Doxorubicin Drug-Eluting Bead Chemoembolization | 2015 | Dec | J Vasc Interv Radiol | 26 | 12 | 1777-86 | 10.1016/j.jvir.2015.08.023 | Wrong patient population |
| 430 | Kolligs, F. T.; Bilbao, J. I.; Jakobs, T.; Iñarrairaegui, M.; Nagel, J. M.; Rodriguez, M.; Haug, A.; D'Avola, D.; Op den Winkel, M.; Martinez-Cuesta, A.; Trumm, C.; Benito, A.; Tatsch, K.; Zech, C. J.; Hoffmann, R. T.; Sangro, B. | Pilot randomized trial of selective internal radiation therapy vs. chemoembolization in unresectable hepatocellular carcinoma | 2015 | Jun | Liver Int | 35 | 6 | 1715-21 | 10.1111/liv.12750 |  |
| 431 | Kollmann, D.; Selzner, N.; Selzner, M. | Bridging to liver transplantation in HCC patients | 2017 | Sep | Langenbecks Arch Surg | 402 | 6 | 863-871 | 10.1007/s00423-017-1609-2 |  |
| 432 | Kondo, Y.; Kimura, O.; Shimosegawa, T. | Radiation therapy has been shown to be adaptable for various stages of hepatocellular carcinoma | 2015 | 7-Jan | World J Gastroenterol | 21 | 1 | 94-101 | 10.3748/wjg.v21.i1.94 |  |
| 433 | Kong, Jie-Yu; Li, Shu-Mei; Fan, Hai-Yan; Zhang, Lan; Zhao, Hui-Jin; Li, Sheng-Mian | Transarterial chemoembolization extends long-term survival in patients with unresectable hepatocellular carcinoma | 2018 |  | Medicine | 97 | 33 |  |  | Wrong patient population |
| 434 | Kong, M.; Hong, S. E.; Choi, W. S.; Choi, J.; Kim, Y. | Treatment outcomes of helical intensity-modulated radiotherapy for unresectable hepatocellular carcinoma | 2013 | May | Gut Liver | 7 | 3 | 343-51 | 10.5009/gnl.2013.7.3.343 |  |
| 435 | Kong, Shun-Yu; Song, Jiao-Jiao; Jin, Yao-Qi; Deng, Man-Jun; Yan, Jing-Xin | Hepatic arterial infusion chemotherapy versus transarterial chemoembolization for patients with unresectable hepatocellular carcinoma: a systematic review and meta-analysis | 2023 |  | Acta Clinica Belgica | 78 | 2 | 171-179 |  | Wrong study design |
| 436 | Kong, Y. L.; Sun, J. J.; Zhang, H. Y.; Xing, Y.; Wang, C.; Liu, Y.; He, X. J.; Kong, L. H.; Liu, C. L. | Clinical evaluation of percutaneous endovascular radiofrequency ablation for portal vein tumor thrombus: experience in 120 patients | 2023 | Feb | Surg Endosc | 37 | 2 | 1173-1180 | 10.1007/s00464-022-09639-3 |  |
| 437 | Kosaka, Y.; Kawaoka, T.; Kosaka, M.; Shirane, Y.; Miura, R.; Murakami, S.; Johira, Y.; Yano, S.; Amioka, K.; Naruto, K.; Ando, Y.; Kodama, K.; Uchikawa, S.; Fujino, H.; Ohno, A.; Nakahara, T.; Murakami, E.; Okamoto, W.; Yamauchi, M.; Imamura, M.; Aikata, H. | Successful Lenvatinib Re-challenge following Atezolizumab Plus Bevacizumab Combination Therapy Failure for Unresectable Hepatocellular Carcinoma | 2023 | 15-Jun | Intern Med | 62 | 12 | 1771-1774 | 10.2169/internalmedicine.9581-22 |  |
| 438 | Kreidieh, M.; Zeidan, Y. H.; Shamseddine, A. | The Combination of Stereotactic Body Radiation Therapy and Immunotherapy in Primary Liver Tumors | 2019 |  | J Oncol | 2019 |  | 4304817 | 10.1155/2019/4304817 |  |
| 439 | Krzyston, H.; Morse, B.; Deperalta, D.; Rishi, A.; Kayaleh, R.; El-Haddad, G.; Smith, J.; Druta, M.; Kis, B. | Liver-directed treatments of liver-dominant metastatic leiomyosarcoma | 2020 | Sep | Diagnostic and Interventional Radiology | 26 | 5 | 449-455 | 10.5152/dir.2020.19405 |  |
| 440 | Kubota, K.; Hidaka, H.; Nakazawa, T.; Okuwaki, Y.; Yamane, K.; Inoue, T.; Uojima, H.; Takada, J.; Tanaka, Y.; Shibuya, A.; Fujii, K.; Woodhams, R.; Matsunaga, K.; Kokubu, S.; Koizumi, W. | Prospective, randomized, controlled study of the efficacy of transcatheter arterial chemoembolization with miriplatin for hepatocellular carcinoma | 2018 | Feb | Hepatol Res | 48 | 3 | E98-e106 | 10.1111/hepr.12933 |  |
| 441 | Kucukay, F.; Badem, S.; Karan, A.; Ozdemir, M.; Okten, R. S.; Ozbulbul, N. I.; Kucukay, M. B.; Unlu, I.; Bostanci, E. B.; Akdogan, M. | A Single-Center Retrospective Comparison of Doxorubicin-Loaded HepaSphere Transarterial Chemoembolization with Conventional Transarterial Chemoembolization for Patients with Unresectable Hepatocellular Carcinoma | 2015 | Nov | J Vasc Interv Radiol | 26 | 11 | 1622-9 | 10.1016/j.jvir.2015.07.017 | Wrong patient population |
| 442 | Kudo, M. | Atezolizumab plus Bevacizumab Followed by Curative Conversion (ABC Conversion) in Patients with Unresectable, TACE-Unsuitable Intermediate-Stage Hepatocellular Carcinoma | 2022 | Sep | Liver Cancer | 11 | 5 | 399-406 | 10.1159/000526163 |  |
| 443 | Kudo, M.; Aoki, T.; Ueshima, K.; Tsuchiya, K.; Morita, M.; Chishina, H.; Takita, M.; Hagiwara, S.; Minami, Y.; Ida, H.; Nishida, N.; Ogawa, C.; Tomonari, T.; Nakamura, N.; Kuroda, H.; Takebe, A.; Takeyama, Y.; Hidaka, M.; Eguchi, S.; Chan, S. L.; Kurosaki, M.; Izumi, N. | Achievement of Complete Response and Drug-Free Status by Atezolizumab plus Bevacizumab Combined with or without Curative Conversion in Patients with Transarterial Chemoembolization-Unsuitable, Intermediate-Stage Hepatocellular Carcinoma: A Multicenter Proof-Of-Concept Study | 2023 | Sep | Liver Cancer | 12 | 4 | 321-338 | 10.1159/000529574 |  |
| 444 | Kudo, M.; Arizumi, T. | Transarterial Chemoembolization in Combination with a Molecular Targeted Agent: Lessons Learned from Negative Trials (Post-TACE, BRISK-TA, SPACE, ORIENTAL, and TACE-2) | 2017 |  | Oncology | 93 Suppl 1 |  | 127-134 | 10.1159/000481243 |  |
| 445 | Kudo, M.; Cheng, A. L.; Park, J. W.; Park, J. H.; Liang, P. C.; Hidaka, H.; Izumi, N.; Heo, J.; Lee, Y. J.; Sheen, I. S.; Chiu, C. F.; Arioka, H.; Morita, S.; Arai, Y. | Orantinib versus placebo combined with transcatheter arterial chemoembolisation in patients with unresectable hepatocellular carcinoma (ORIENTAL): a randomised, double-blind, placebo-controlled, multicentre, phase 3 study | 2018 | Jan | Lancet Gastroenterol Hepatol | 3 | 1 | 37-46 | 10.1016/s2468-1253(17)30290-x | Wrong patient population |
| 446 | Kudo, M.; Han, G.; Finn, R. S.; Poon, R. T.; Blanc, J. F.; Yan, L.; Yang, J.; Lu, L.; Tak, W. Y.; Yu, X.; Lee, J. H.; Lin, S. M.; Wu, C.; Tanwandee, T.; Shao, G.; Walters, I. B.; Dela Cruz, C.; Poulart, V.; Wang, J. H. | Brivanib as adjuvant therapy to transarterial chemoembolization in patients with hepatocellular carcinoma: A randomized phase III trial | 2014 | Nov | Hepatology | 60 | 5 | 1697-707 | 10.1002/hep.27290 |  |
| 447 | Kudo, M.; Ikeda, M.; Takayama, T.; Numata, K.; Izumi, N.; Furuse, J.; Okusaka, T.; Kadoya, M.; Yamashita, S.; Ito, Y.; Kokudo, N. | Safety and efficacy of sorafenib in Japanese patients with hepatocellular carcinoma in clinical practice: a subgroup analysis of GIDEON | 2016 | Dec | J Gastroenterol | 51 | 12 | 1150-1160 | 10.1007/s00535-016-1204-2 | Wrong patient population |
| 448 | Kudo, M.; Ueshima, K.; Chan, S.; Minami, T.; Chishina, H.; Aoki, T.; Takita, M.; Hagiwara, S.; Minami, Y.; Ida, H.; Takenaka, M.; Sakurai, T.; Watanabe, T.; Morita, M.; Ogawa, C.; Wada, Y.; Ikeda, M.; Ishii, H.; Izumi, N.; Nishida, N. | Lenvatinib as an Initial Treatment in Patients with Intermediate-Stage Hepatocellular Carcinoma Beyond Up-To-Seven Criteria and Child-Pugh A Liver Function: A Proof-Of-Concept Study | 2019 | 31-Jul | Cancers (Basel) | 11 | 8 |  | 10.3390/cancers11081084 |  |
| 449 | Kudo, M.; Ueshima, K.; Yokosuka, O.; Ogasawara, S.; Obi, S.; Izumi, N.; Aikata, H.; Nagano, H.; Hatano, E.; Sasaki, Y.; Hino, K.; Kumada, T.; Yamamoto, K.; Imai, Y.; Iwadou, S.; Ogawa, C.; Okusaka, T.; Kanai, F.; Akazawa, K.; Yoshimura, K. I.; Johnson, P.; Arai, Y. | Sorafenib plus low-dose cisplatin and fluorouracil hepatic arterial infusion chemotherapy versus sorafenib alone in patients with advanced hepatocellular carcinoma (SILIUS): a randomised, open label, phase 3 trial | 2018 | Jun | Lancet Gastroenterol Hepatol | 3 | 6 | 424-432 | 10.1016/s2468-1253(18)30078-5 |  |
| 450 | Kudo, Masatoshi; Ueshima, Kazuomi; Ikeda, Masafumi; Torimura, Takuji; Tanabe, Nobukazu; Aikata, Hiroshi; Izumi, Namiki; Yamasaki, Takahiro; Nojiri, Shunsuke; Hino, Keisuke | Final results of TACTICS: a randomized, prospective trial comparing transarterial chemoembolization plus sorafenib to transarterial chemoembolization alone in patients with unresectable hepatocellular carcinoma | 2022 |  | Liver Cancer | 11 | 4 | 354-367 |  | Wrong patient population |
| 451 | Kuroda, H.; Oikawa, T.; Ninomiya, M.; Fujita, M.; Abe, K.; Okumoto, K.; Katsumi, T.; Sato, W.; Igarashi, G.; Iino, C.; Endo, T.; Tanabe, N.; Numao, H.; Fukuda, S.; Iijima, K.; Masamune, A.; Ohira, H.; Ueno, Y.; Takikawa, Y. | Objective Response by mRECIST to Initial Lenvatinib Therapy Is an Independent Factor Contributing to Deep Response in Hepatocellular Carcinoma Treated with Lenvatinib-Transcatheter Arterial Chemoembolization Sequential Therapy | 2022 | Jul | Liver Cancer | 11 | 4 | 383-396 | 10.1159/000522424 |  |
| 452 | Kwok, T. W.; Wong, S. M.; Yu, C. H.; Young, W. M. | Hypersensitivity Reactions to Transarterial Chemoembolization with Cisplatin and Ethiodized Oil: A Retrospective Cohort Study | 2023 | May | J Vasc Interv Radiol | 34 | 5 | 799-806.e2 | 10.1016/j.jvir.2022.12.470 |  |
| 453 | Labadie, K. P.; Schaub, S. K.; Khorsand, D.; Johnson, G.; Apisarnthanarax, S.; Park, J. O. | Multidisciplinary approach for multifocal, bilobar hepatocellular carcinoma: A case report and literature review | 2019 | 27-Jan | World J Hepatol | 11 | 1 | 119-126 | 10.4254/wjh.v11.i1.119 |  |
| 454 | Lakhoo, J.; Adams, R.; Dave, A.; Luo, L.; Vargo, C. J.; Isaacson, A. J.; Sher, A.; Fischman, A.; Yee, D. C.; Ryan, S.; Patel, S.; Duchac, D.; Brown, D. B. | Radiopaque beads loaded with doxorubicin in the treatment of patients with hepatocellular carcinoma: A retrospective, multi-center study | 2020 |  | Cancer Treat Res Commun | 25 |  | 100208 | 10.1016/j.ctarc.2020.100208 | Wrong patient population |
| 455 | Lan, Tian; Chang, Lei; Rahmathullah, MN; Wu, Long; Yuan, Yu-Feng | Comparative efficacy of interventional therapies for early-stage hepatocellular carcinoma: a PRISMA-compliant systematic review and network meta-analysis | 2016 |  | Medicine | 95 | 15 |  |  | Wrong outcomes |
| 456 | Lanza, E.; Donadon, M.; Poretti, D.; Pedicini, V.; Tramarin, M.; Roncalli, M.; Rhee, H.; Park, Y. N.; Torzilli, G. | Transarterial Therapies for Hepatocellular Carcinoma | 2016 | Nov | Liver Cancer | 6 | 1 | 27-33 | 10.1159/000449347 |  |
| 457 | Lanza, E.; Masetti, C.; Messana, G.; Muglia, R.; Pugliese, N.; Ceriani, R.; Lleo de Nalda, A.; Rimassa, L.; Torzilli, G.; Poretti, D.; D'Antuono, F.; Politi, L. S.; Pedicini, V.; Aghemo, A. | Sarcopenia as a predictor of survival in patients undergoing bland transarterial embolization for unresectable hepatocellular carcinoma | 2020 |  | PLoS One | 15 | 6 | e0232371 | 10.1371/journal.pone.0232371 |  |
| 458 | Lanza, E.; Muglia, R.; Bolengo, I.; Poretti, D.; D'Antuono, F.; Ceriani, R.; Torzilli, G.; Pedicini, V. | Survival analysis of 230 patients with unresectable hepatocellular carcinoma treated with bland transarterial embolization | 2020 |  | PLoS One | 15 | 1 | e0227711 | 10.1371/journal.pone.0227711 |  |
| 459 | Lawson, Alexander; Kamarajah, Sivesh K; Parente, Alessandro; Pufal, Kamil; Sundareyan, Ramanivas; Pawlik, Timothy M; Ma, Yuk Ting; Shah, Tahir; Kharkhanis, Salil; Dasari, Bobby VM | Outcomes of Transarterial Embolisation (TAE) vs. Transarterial Chemoembolisation (TACE) for Hepatocellular Carcinoma: A Systematic Review and Meta-Analysis | 2023 |  | Cancers | 15 | 12 | 3166 |  | Wrong study design |
| 460 | Lee, A.; Lee, J.; Yang, H.; Sung, S. Y.; Jeon, C. H.; Kim, S. H.; Choi, M. H.; Lee, Y. J.; Chun, H. J.; Bae, S. H. | Multidisciplinary treatment with immune checkpoint inhibitors for advanced stage hepatocellular carcinoma | 2022 | Mar | J Liver Cancer | 22 | 1 | 75-83 | 10.17998/jlc.2022.03.04 |  |
| 461 | Lee, E. W.; Khan, S. | Recent advances in transarterial embolotherapies in the treatment of hepatocellular carcinoma | 2017 | Dec | Clin Mol Hepatol | 23 | 4 | 265-272 | 10.3350/cmh.2017.0111 |  |
| 462 | Lee, J. M.; Lee, K. W.; Kim, H. C.; Yi, N. J.; Suh, K. S. | No touch isolation technique for the prevention of postoperative recurrence of hepatocellular carcinoma after liver transplantation-combined with trans-arterial radioembolization | 2020 | Dec | Surg Oncol | 35 |  | 189-190 | 10.1016/j.suronc.2020.08.024 |  |
| 463 | Lee, J. S.; Choi, H. J.; Kim, B. K.; Park, J. Y.; Kim, D. Y.; Ahn, S. H.; Han, K. H.; Baek, S. E.; Chung, Y. E.; Park, M. S.; Kim, M. J.; Rhee, H.; Kim, S. U. | The Modified Response Evaluation Criteria in Solid Tumors (RECIST) Yield a More Accurate Prognoses Than the RECIST 1.1 in Hepatocellular Carcinoma Treated with Transarterial Radioembolization | 2020 | 15-Nov | Gut Liver | 14 | 6 | 765-774 | 10.5009/gnl19197 |  |
| 464 | Lee, M. Y.; Chuang, V. P.; Wei, C. J.; Cheng, T. Y.; Cherng, M. T. | Histopathologic correlation of hepatocellular carcinoma after transcatheter arterial chemoembolization with polyvinyl alcohol particle of various sizes | 2012 | Sep | Eur J Radiol | 81 | 9 | 1976-9 | 10.1016/j.ejrad.2011.05.004 |  |
| 465 | Lee, S. G.; Cho, S. M.; Whang, K.; Jang, Y. G.; Kim, J.; Choi, J. | Spinal Cord Infarction After Transarterial Chemoembolization for Hepatocellular Carcinoma | 2022 | Oct | Korean J Neurotrauma | 18 | 2 | 404-409 | 10.13004/kjnt.2022.18.e65 |  |
| 466 | Lee, S. W.; Lee, H. L.; Han, N. I.; Kwon, J. H.; Nam, S. W.; Jang, J. W.; Bae, S. H.; Choi, J. Y.; Yoon, S. K. | Transarterial infusion of epirubicin and cisplatin combined with systemic infusion of 5-fluorouracil versus transarterial chemoembolization using doxorubicin for unresectable hepatocellular carcinoma with portal vein tumor thrombosis: a retrospective analysis | 2017 | Oct | Ther Adv Med Oncol | 9 | 10 | 615-626 | 10.1177/1758834017728018 |  |
| 467 | Lee, Shou-Wu; Lee, Teng-Yu; Cheng, Yu-Chi; Yen, Chieh-Ling; Yang, Sheng Shun | The correlation with tumor radiological characteristics and prognosis of patients with early-stage hepatocellular carcinoma receiving transcatheter arterial chemoembolization | 2021 |  | Medicine | 100 | 26 |  |  |  |
| 468 | Lee, T. Y.; Lin, C. C.; Chen, C. Y.; Wang, T. E.; Lo, G. H.; Chang, C. S.; Chao, Y. | Combination of transcatheter arterial chemoembolization and interrupted dosing sorafenib improves patient survival in early-intermediate stage hepatocellular carcinoma: A post hoc analysis of the START trial | 2017 | Sep | Medicine (Baltimore) | 96 | 37 | e7655 | 10.1097/md.0000000000007655 | Wrong patient population |
| 469 | Lee, V. H. F.; Seong, J.; Yoon, S. M.; Wong, T. C. L.; Wang, B.; Zhang, J. L.; Chiang, C. L.; Ho, P. P. Y.; Dawson, L. A. | Contrasting Some Differences in Managing Advanced Unresectable Hepatocellular Carcinoma Between the East and the West | 2019 | Aug | Clin Oncol (R Coll Radiol) | 31 | 8 | 560-569 | 10.1016/j.clon.2019.06.002 |  |
| 470 | Lei, K.; Deng, Z. F.; Wang, J. G.; You, K.; Xu, J.; Liu, Z. J. | PNI-Based Nomograms to Predict Tumor Progression and Survival for Patients with Unresectable Hepatocellular Carcinoma Undergoing Transcatheter Arterial Chemoembolization | 2023 | 6-Jan | J Clin Med | 12 | 2 |  | 10.3390/jcm12020486 |  |
| 471 | Lei, K.; Deng, Z.; Wang, J.; Wang, H.; Hu, R.; Li, Y.; Wang, X.; Xu, J.; You, K.; Liu, Z. | A novel nomogram based on the hematological prognosis risk scoring system can predict the overall survival of patients with hepatocellular carcinoma | 2023 | Nov | J Cancer Res Clin Oncol | 149 | 16 | 14631-14640 | 10.1007/s00432-023-05255-3 |  |
| 472 | Lei, K.; Wang, J. G.; Li, Y.; Wang, H. X.; Xu, J.; You, K.; Liu, Z. J. | Prognostic value of preoperative prealbumin levels in patients with unresectable hepatocellular carcinoma undergoing transcatheter arterial chemoembolisation | 2023 | Aug | Heliyon | 9 | 8 | e18494 | 10.1016/j.heliyon.2023.e18494 |  |
| 473 | Lencioni, Riccardo; Petruzzi, Pasquale; Crocetti, Laura | Chemoembolization of hepatocellular carcinoma | 2013 |  | Seminars in interventional radiology | 30 | 1 | 003-011 |  | Wrong patient population |
| 474 | Leowattana, W.; Leowattana, T.; Leowattana, P. | Systemic treatment for unresectable hepatocellular carcinoma | 2023 | 14-Mar | World J Gastroenterol | 29 | 10 | 1551-1568 | 10.3748/wjg.v29.i10.1551 |  |
| 475 | Lewis, S.; Dawson, L.; Barry, A.; Stanescu, T.; Mohamad, I.; Hosni, A. | Stereotactic body radiation therapy for hepatocellular carcinoma: From infancy to ongoing maturity | 2022 | Aug | JHEP Rep | 4 | 8 | 100498 | 10.1016/j.jhepr.2022.100498 |  |
| 476 | Li, H. Z.; Tan, J.; Tang, T.; An, T. Z.; Li, J. X.; Xiao, Y. D. | Chemoembolization Plus Microwave Ablation vs Chemoembolization Alone in Unresectable Hepatocellular Carcinoma Beyond the Milan Criteria: A Propensity Scoring Matching Study | 2021 |  | J Hepatocell Carcinoma | 8 |  | 1311-1322 | 10.2147/jhc.S338456 | Wrong patient population |
| 477 | Li, H.; Li, S.; Geng, J.; Zhao, S.; Tan, K.; Yang, Z.; Feng, D.; Liu, L. | Efficacy evaluation of the combination therapy of sorafenib and transarterial chemoembolization for unresectable HCC: a systematic review and meta-analysis of comparative studies | 2020 | Apr | Ann Transl Med | 8 | 8 | 540 | 10.21037/atm.2020.02.115 | Wrong patient population |
| 478 | Li, H.; Wang, J.; Zhang, G.; Kuang, D.; Li, Y.; He, X.; Xing, C.; Wang, Y.; Shi, M.; Han, X.; Ren, J.; Duan, X. | Transarterial chemoembolization combined donafenib with/without PD-1 for unresectable HCC in a multicenter retrospective study | 2023 |  | Front Immunol | 14 |  | 1277329 | 10.3389/fimmu.2023.1277329 |  |
| 479 | Li, H.; Wu, Z.; Chen, J.; Su, K.; Guo, L.; Xu, K.; Gu, T.; Jiang, Y.; Wang, P.; Zeng, H.; Chi, H.; He, K.; Han, Y. | External radiotherapy combined with sorafenib has better efficacy in unresectable hepatocellular carcinoma: a systematic review and meta-analysis | 2023 | Sep | Clin Exp Med | 23 | 5 | 1537-1549 | 10.1007/s10238-022-00972-4 | Wrong study design |
| 480 | Li, J. H.; Wang, Y.; Xie, X. Y.; Yin, X.; Zhang, L.; Chen, R. X.; Ren, Z. G. | Aspirin in combination with TACE in treatment of unresectable HCC: a matched-pairs analysis | 2016 |  | Am J Cancer Res | 6 | 9 | 2109-2116 |  |  |
| 481 | Li, J. H.; Xie, X. Y.; Zhang, L.; Le, F.; Ge, N. L.; Li, L. X.; Gan, Y. H.; Chen, Y.; Zhang, J. B.; Xue, T. C.; Chen, R. X.; Xia, J. L.; Zhang, B. H.; Ye, S. L.; Wang, Y. H.; Ren, Z. G. | Oxaliplatin and 5-fluorouracil hepatic infusion with lipiodolized chemoembolization in large hepatocellular carcinoma | 2015 | 7-Apr | World J Gastroenterol | 21 | 13 | 3970-7 | 10.3748/wjg.v21.i13.3970 | Wrong patient population |
| 482 | Li, J. R.; Yang, D. L.; Wang, J. M.; Tian, W.; Wei, W.; Luo, C. P.; Qi, L. N.; Ma, L.; Zhong, J. H. | Importance of optimizing duration of adjuvant immune checkpoint inhibitor therapy to treat postoperative hepatocellular carcinoma after conversion therapy: a case report | 2023 | Nov | J Surg Case Rep | 2023 | 11 | rjad610 | 10.1093/jscr/rjad610 |  |
| 483 | Li, J. X.; Wu, H.; Huang, J. W.; Zeng, Y. | The influence on liver function after transcatheter arterial chemoembolization combined with percutaneous radiofrequency ablation in patients with hepatocellular carcinoma | 2012 | Sep | J Formos Med Assoc | 111 | 9 | 510-5 | 10.1016/j.jfma.2011.05.016 |  |
| 484 | Li, J.; Kong, M.; Yu, G.; Wang, S.; Shi, Z.; Han, H.; Lin, Y.; Shi, J.; Song, J. | Safety and efficacy of transarterial chemoembolization combined with tyrosine kinase inhibitors and camrelizumab in the treatment of patients with advanced unresectable hepatocellular carcinoma | 2023 |  | Front Immunol | 14 |  | 1188308 | 10.3389/fimmu.2023.1188308 |  |
| 485 | Li, J.; Lei, J.; Wang, W.; Lin, L.; Wang, J.; Ma, L.; Yan, L. | Reasons for Dropout from Transcatheter Arterial Chemoembolization (TACE) when Served as a Down-Staging Therapy for Advanced Hepatocellular Carcinoma | 2014 | May | Hepatogastroenterology | 61 | 131 | 717-21 |  |  |
| 486 | Li, J.; Wang, N.; Shi, C.; Liu, Q.; Song, J.; Ye, X. | Short-term efficacy and safety of callispheres drug-loaded microsphere embolization in primary hepatocellular carcinoma | 2021 | Jul | J Cancer Res Ther | 17 | 3 | 733-739 | 10.4103/jcrt.JCRT_1848_20 | Wrong patient population |
| 487 | Li, J.; Xu, W.; Li, D.; Liu, T.; Zhang, Y. S.; Ding, J.; Chen, X. | Locally Deployable Nanofiber Patch for Sequential Drug Delivery in Treatment of Primary and Advanced Orthotopic Hepatomas | 2018 | 24-Jul | ACS Nano | 12 | 7 | 6685-6699 | 10.1021/acsnano.8b01729 |  |
| 488 | Li, J.; Zhang, F.; Yang, J.; Zhang, Y.; Wang, Y.; Fan, W.; Huang, Y.; Wang, W.; Ran, H.; Ke, S. | Combination of individualized local control and target-specific agent to improve unresectable liver cancer managements: a matched case-control study | 2015 | Jun | Target Oncol | 10 | 2 | 287-95 | 10.1007/s11523-014-0338-5 | Wrong patient population |
| 489 | Li, L.; Tian, J.; Liu, P.; Wang, X.; Zhu, Z. | Transarterial chemoembolization combination therapy vs monotherapy in unresectable hepatocellular carcinoma: a meta-analysis | 2016 | 2-Jun | Tumori | 2016 | 3 | 301-10 | 10.5301/tj.5000491 | Wrong patient population |
| 490 | Li, L.; Zhao, W.; Wang, M.; Hu, J.; Wang, E.; Zhao, Y.; Liu, L. | Transarterial chemoembolization plus sorafenib for the management of unresectable hepatocellular carcinoma: a systematic review and meta-analysis | 2018 | 4-Sep | BMC Gastroenterol | 18 | 1 | 138 | 10.1186/s12876-018-0849-0 | Wrong patient population |
| 491 | Li, Ningjie; Yang, Ping; Fang, Jun | Transarterial chemoembolization (TACE) plus apatinib vs. TACE alone for hepatocellular carcinoma | 2022 |  | Clinics and Research in Hepatology and Gastroenterology | 46 | 9 | 102022 |  | Wrong patient population |
| 492 | Li, P. P.; Huang, G.; Jia, N. Y.; Pan, Z. Y.; Liu, H.; Yang, Y.; He, C. J.; Lau, W. Y.; Yang, Y. F.; Zhou, W. P. | Associating liver partition and portal vein ligation for staged hepatectomy versus sequential transarterial chemoembolization and portal vein embolization in staged hepatectomy for HBV-related hepatocellular carcinoma: a randomized comparative study | 2022 | Feb | Hepatobiliary Surg Nutr | 11 | 1 | 38-51 | 10.21037/hbsn-20-264 |  |
| 493 | Li, P. P.; Huang, G.; Jia, N. Y.; Pan, Z. Y.; Liu, H.; Yang, Y.; He, C. J.; Lau, W. Y.; Yang, Y. F.; Zhou, W. P. | Associating liver partition and portal vein ligation for staged hepatectomy versus sequential transarterial chemoembolization and portal vein embolization in staged hepatectomy for HBV-related hepatocellular carcinoma: a randomized comparative study | 2022 | Feb | Hepatobiliary Surg Nutr | 11 | 1 | 38-51 | 10.21037/hbsn-20-264 |  |
| 494 | Li, Q. J.; He, M. K.; Chen, H. W.; Fang, W. Q.; Zhou, Y. M.; Xu, L.; Wei, W.; Zhang, Y. J.; Guo, Y.; Guo, R. P.; Chen, M. S.; Shi, M. | Hepatic Arterial Infusion of Oxaliplatin, Fluorouracil, and Leucovorin Versus Transarterial Chemoembolization for Large Hepatocellular Carcinoma: A Randomized Phase III Trial | 2022 | 10-Jan | J Clin Oncol | 40 | 2 | 150-160 | 10.1200/jco.21.00608 | Wrong patient population |
| 495 | Li, Q. J.; He, M. K.; Chen, H. W.; Fang, W. Q.; Zhou, Y. M.; Xu, L.; Wei, W.; Zhang, Y. J.; Guo, Y.; Guo, R. P.; Chen, M. S.; Shi, M. | Hepatic Arterial Infusion of Oxaliplatin, Fluorouracil, and Leucovorin Versus Transarterial Chemoembolization for Large Hepatocellular Carcinoma: A Randomized Phase III Trial | 2022 | 10-Jan | J Clin Oncol | 40 | 2 | 150-160 | 10.1200/jco.21.00608 | Wrong patient population |
| 496 | Li, Q.; Wu, T.; Ma, X. A.; Jing, L.; Han, L. L.; Guo, H. | Prognostic role of ABO blood group in patients with unresectable hepatocellular carcinoma after transarterial chemoembolization | 2018 |  | Ther Clin Risk Manag | 14 |  | 991-998 | 10.2147/tcrm.S160089 |  |
| 497 | Li, S. Q.; Wu, J. Y.; Wu, J. Y.; Xie, H.; Li, J. H.; Zeng, Z. X.; Fu, Y. K.; Liu, D. Y.; Li, H.; Chen, W. Z.; Huang, J. Y.; Yan, M. L. | Transarterial Chemoembolization Plus Lenvatinib and PD-1 Inhibitors for Hepatocellular Carcinoma with Main Trunk Portal Vein Tumor Thrombus: A Multicenter Retrospective Study | 2023 |  | J Hepatocell Carcinoma | 10 |  | 1799-1811 | 10.2147/jhc.S428980 |  |
| 498 | Li, S.; Guo, J. H.; Lu, J.; Wang, C.; Wang, H. | Prognostic Value of Preoperative Prognostic Nutritional Index and Body Mass Index Combination in Patients with Unresectable Hepatocellular Carcinoma After Transarterial Chemoembolization | 2021 |  | Cancer Manag Res | 13 |  | 1637-1650 | 10.2147/cmar.S290983 |  |
| 499 | Li, S.; He, X.; Dang, L.; Xu, F.; Fang, J.; Li, F.; Wang, W. | Efficacy of (125)I Versus Non-(125)I Combined with Transcatheter Arterial Chemoembolization for the Treatment of Unresectable Hepatocellular Carcinoma with Obstructive Jaundice | 2018 | Feb | Dig Dis Sci | 63 | 2 | 321-328 | 10.1007/s10620-017-4899-x |  |
| 500 | Li, S.; Li, Y. | Is lenvatinib in combination with transarterial chemoembolization benefit for unresectable hepatocellular carcinoma (uHCC)? The age interference needs to be eliminated | 2023 | 9-Nov | Hepatol Int |  |  |  | 10.1007/s12072-023-10611-z |  |
| 501 | Li, S.; Wu, J.; Wu, J.; Fu, Y.; Zeng, Z.; Li, Y.; Li, H.; Liao, W.; Yan, M. | Prediction of early treatment response to the combination therapy of TACE plus lenvatinib and anti-PD-1 antibody immunotherapy for unresectable hepatocellular carcinoma: Multicenter retrospective study | 2023 |  | Front Immunol | 14 |  | 1109771 | 10.3389/fimmu.2023.1109771 |  |
| 502 | Li, W.; Pei, Y.; Wang, Z.; Liu, J. | Efficacy of transarterial chemoembolization monotherapy or combination conversion therapy in unresectable hepatocellular carcinoma: A systematic review and meta-analysis | 2022 |  | Front Oncol | 12 |  | 930868 | 10.3389/fonc.2022.930868 | Wrong study design |
| 503 | Li, X. L.; Guo, W. X.; Hong, X. D.; Yang, L.; Wang, K.; Shi, J.; Li, N.; Wu, M. C.; Cheng, S. Q. | Efficacy of the treatment of transarterial chemoembolization combined with radiotherapy for hepatocellular carcinoma with portal vein tumor thrombus: A propensity score analysis | 2016 | Oct | Hepatol Res | 46 | 11 | 1088-1098 | 10.1111/hepr.12657 | Wrong patient population |
| 504 | Li, X.; Chen, J.; Wang, X.; Bai, T.; Lu, S.; Wei, T.; Tang, Z.; Huang, C.; Zhang, B.; Liu, B.; Li, L.; Wu, F. | Outcomes and prognostic factors in initially unresectable hepatocellular carcinoma treated using conversion therapy with lenvatinib and TACE plus PD-1 inhibitors | 2023 |  | Front Oncol | 13 |  | 1110689 | 10.3389/fonc.2023.1110689 |  |
| 505 | Li, X.; Fu, Z.; Chen, X.; Cao, K.; Zhong, J.; Liu, L.; Ding, N.; Zhang, X.; Zhai, J.; Qu, Z. | Efficacy and Safety of Lenvatinib Combined With PD-1 Inhibitors Plus TACE for Unresectable Hepatocellular Carcinoma Patients in China Real-World | 2022 |  | Front Oncol | 12 |  | 950266 | 10.3389/fonc.2022.950266 |  |
| 506 | Li, X.; He, G.; Su, F.; Chu, Z.; Xu, L.; Zhang, Y.; Zhou, J.; Ding, Y. | Regorafenib-loaded poly (lactide-co-glycolide) microspheres designed to improve transarterial chemoembolization therapy for hepatocellular carcinoma | 2020 | Nov | Asian J Pharm Sci | 15 | 6 | 739-751 | 10.1016/j.ajps.2020.01.001 |  |
| 507 | Li, X.; Sun, W.; Ding, X.; Li, W.; Chen, J. | Prognostic model of immune checkpoint inhibitors combined with anti-angiogenic agents in unresectable hepatocellular carcinoma | 2022 |  | Front Immunol | 13 |  | 1060051 | 10.3389/fimmu.2022.1060051 |  |
| 508 | Li, X.; Wang, X.; Bai, T.; Chen, J.; Lu, S.; Wei, T.; Tang, Z.; Zhao, G.; Lu, H.; Li, L.; Wu, F. | Conversion surgery for initially unresectable hepatocellular carcinoma using lenvatinib combined with TACE plus PD-1 inhibitor: A real-world observational study | 2023 | 18-Dec | Dig Liver Dis |  |  |  | 10.1016/j.dld.2023.11.027 |  |
| 509 | Li, Y.; Zhao, R.; Li, X. | Clinical outcomes of hepatocellular carcinoma patients after hepatectomy treated with TACE in combination with sorafenib: a propensity score matched analysis | 2023 | 31-May | Transl Cancer Res | 12 | 5 | 1088-1099 | 10.21037/tcr-22-2784 | Wrong patient population |
| 510 | Li, Y.; Zheng, Y. B.; Zhao, W.; Liu, B.; Hu, B. S.; He, X.; Huang, J. W.; Lu, L. G. | Sorafenib in combination with transarterial chemoembolization and radiofrequency ablation in the treatment for unresectable hepatocellular carcinoma | 2013 | Dec | Med Oncol | 30 | 4 | 730 | 10.1007/s12032-013-0730-5 | Wrong patient population |
| 511 | Li, Z.; Han, X. | Re: Survival and Tolerability of Transarterial Chemoembolization in Greater Versus less than 70 Years of Age Patients with Unresectable Hepatocellular Carcinoma-A Propensity Analysis | 2021 | Apr | Cardiovasc Intervent Radiol | 44 | 4 | 662 | 10.1007/s00270-020-02701-4 |  |
| 512 | Li, Z.; Li, Q.; Wang, X.; Li, S.; Chen, W.; Jin, X.; Liu, X.; Dai, Z.; Liu, X.; Zheng, X.; Li, P.; Zhang, H.; Zhang, Q.; Luo, H.; Liu, R. | Carbon Ion Radiotherapy Acts as the Optimal Treatment Strategy for Unresectable Liver Cancer During the Coronavirus Disease 2019 Crisis | 2021 |  | Front Public Health | 9 |  | 767617 | 10.3389/fpubh.2021.767617 |  |
| 513 | Liang, H. Y.; Lu, L. G.; Hu, B. S.; Li, Y.; Shao, P. J. | Effects of sorafenib combined with chemoembolization and radiofrequency ablation for large, unresectable hepatocellular carcinomas | 2013 | Nov | Chin Med J (Engl) | 126 | 22 | 4270-6 |  | Wrong patient population |
| 514 | Liang, K. H.; Lin, C. L.; Chen, S. F.; Chiu, C. W.; Yang, P. C.; Chang, M. L.; Lin, C. C.; Sung, K. F.; Yeh, C.; Hung, C. F.; Chien, R. N.; Yeh, C. T. | GALNT14 genotype effectively predicts the therapeutic response in unresectable hepatocellular carcinoma treated with transcatheter arterial chemoembolization | 2016 | Mar | Pharmacogenomics | 17 | 4 | 353-66 | 10.2217/pgs.15.179 |  |
| 515 | Liao, R.; Zhang, X. D.; Li, G. Z.; Qin, K. L.; Yan, X. | Comparison of transcatheter arterial chemoembolization with raltitrexed plus liposomal doxorubicin vs. tegafur plus pirarubicin for unresectable hepatocellular carcinoma | 2020 | Aug | J Gastrointest Oncol | 11 | 4 | 747-759 | 10.21037/jgo-20-59 | Wrong patient population |
| 516 | Liao, Y.; Wang, B.; Huang, Z. L.; Shi, M.; Yu, X. J.; Zheng, L.; Li, S.; Li, L. | Increased circulating Th17 cells after transarterial chemoembolization correlate with improved survival in stage III hepatocellular carcinoma: a prospective study | 2013 |  | PLoS One | 8 | 4 | e60444 | 10.1371/journal.pone.0060444 |  |
| 517 | Lin, C. Y.; Liu, Y. S.; Pan, K. T.; Chen, C. B.; Hung, C. F.; Chou, C. T. | The short-term safety and efficacy of TANDEM microspheres of various sizes and doxorubicin loading concentrations for hepatocellular carcinoma treatment | 2021 | 10-Jun | Sci Rep | 11 | 1 | 12277 | 10.1038/s41598-021-91021-9 | Wrong patient population |
| 518 | Lin, C.; He, Y.; Liu, M.; Wu, A.; Zhang, J.; Li, S.; Li, S.; Cao, Q.; Liu, F. | Vessels That Encapsulate Tumor Clusters (VETC) Predict cTACE Response in Hepatocellular Carcinoma | 2023 |  | J Hepatocell Carcinoma | 10 |  | 383-397 | 10.2147/jhc.S395903 | Wrong patient population |
| 519 | Lin, H.; Luo, B.; Peng, F.; Fang, C.; Gan, Y.; Yang, X.; Li, B.; Li, Y.; Su, S. | The efficacy of transarterial chemoembolization in downstaging unresectable hepatocellular carcinoma to curative therapy: a predicted regression model | 2022 | Oct | Invest New Drugs | 40 | 5 | 1146-1152 | 10.1007/s10637-022-01261-3 |  |
| 520 | Lin, H.; Wu, H.; Cong, N.; Liu, B.; Liu, C.; Han, D. | Transarterial Chemoembolization Followed by Radiotherapy Versus Sandwich Treatment for Unresectable or Ablative Hepatocellular Carcinoma | 2020 | Jan-Dec | Technol Cancer Res Treat | 19 |  | 1.53E+15 | 10.1177/1533033820983799 | Wrong patient population |
| 521 | Lin, K. Y.; Lin, Z. W.; Chen, Q. J.; Luo, L. P.; Zhang, J. X.; Chen, J. H.; Wang, K.; Tai, S.; Zhang, Z. B.; Wang, S. F.; Zhang, J. Y.; You, W. Y.; Wang, B.; You, P. H.; Lin, K. C.; Yang, T.; Zeng, Y. Y. | Perioperative safety, oncologic outcome, and risk factors of salvage liver resection for initially unresectable hepatocellular carcinoma converted by transarterial chemoembolization plus tyrosine kinase inhibitor and anti-PD-1 antibody: a retrospective multicenter study of 83 patients | 2023 | Dec | Hepatol Int | 17 | 6 | 1477-1489 | 10.1007/s12072-023-10561-6 |  |
| 522 | Lin, W. Y.; Luo, T. Y.; Tsai, S. C.; Kao, C. H.; Tang, I. C.; Huang, P. W. | A comparison of Re-188-MN-16ET-lipiodol and transcatheter arterial chemoembolization in the treatment of hepatoma: an animal study | 2013 | Apr | Nucl Med Biol | 40 | 3 | 437-41 | 10.1016/j.nucmedbio.2012.11.007 |  |
| 523 | Lin, X. J.; Li, Q. J.; Lao, X. M.; Yang, H.; Li, S. P. | Transarterial injection of recombinant human type-5 adenovirus H101 in combination with transarterial chemoembolization (TACE) improves overall and progressive-free survival in unresectable hepatocellular carcinoma (HCC) | 2015 | 15-Oct | BMC Cancer | 15 |  | 707 | 10.1186/s12885-015-1715-x | Wrong patient population |
| 524 | Lin, Y. H.; Hung, S. K.; Chiou, W. Y.; Lee, M. S.; Shen, B. J.; Chen, L. C.; Liu, D. W.; Tsai, W. T.; Lin, P. H.; Shih, Y. T.; Hsu, F. C.; Tsai, S. J.; Chan, M. W. Y.; Lin, H. Y. | Significant symptoms alleviation and tumor volume reduction after combined simultaneously integrated inner-escalated boost and volumetric-modulated arc radiotherapy in a patient with unresectable bulky hepatocellular carcinoma: A care-compliant case report | 2016 | Aug | Medicine (Baltimore) | 95 | 34 | e4717 | 10.1097/md.0000000000004717 |  |
| 525 | Lin, Z. Z.; Shau, W. Y.; Hsu, C.; Shao, Y. Y.; Yeh, Y. C.; Kuo, R. N.; Hsu, C. H.; Yang, J. C.; Cheng, A. L.; Lai, M. S. | Radiofrequency ablation is superior to ethanol injection in early-stage hepatocellular carcinoma irrespective of tumor size | 2013 |  | PLoS One | 8 | 11 | e80276 | 10.1371/journal.pone.0080276 |  |
| 526 | Lin, Z.; Chen, D.; Hu, X.; Huang, D.; Chen, Y.; Zhang, J.; Li, X.; Zou, X. | Clinical efficacy of HAIC (FOLFOX) combined with lenvatinib plus PD-1 inhibitors vs. TACE combined with lenvatinib plus PD-1 inhibitors in the treatment of advanced hepatocellular carcinoma with portal vein tumor thrombus and arterioportal fistulas | 2023 |  | Am J Cancer Res | 13 | 11 | 5455-5465 |  |  |
| 527 | Liu, B.; Gao, S.; Guo, J.; Kou, F.; Liu, S.; Zhang, X.; Wang, X.; Cao, G.; Chen, H.; Liu, P.; Xu, H.; Gao, Q.; Yang, R.; Zhu, X. | A Novel Nomogram for Predicting the Overall Survival in Patients with Unresectable HCC after TACE plus Hepatic Arterial Infusion Chemotherapy | 2023 | Aug | Transl Oncol | 34 |  | 101705 | 10.1016/j.tranon.2023.101705 |  |
| 528 | Liu, B.; Huang, J. W.; Li, Y.; Hu, B. S.; He, X.; Zhao, W.; Zheng, Y. B.; Lu, L. G. | Arsenic trioxide transarterial chemoembolization with and without additional intravenous administration of arsenic trioxide in unresectable hepatocellular carcinoma with lung metastasis: a single-blind, randomized trial | 2015 | Jun | J Cancer Res Clin Oncol | 141 | 6 | 1103-8 | 10.1007/s00432-014-1866-1 |  |
| 529 | Liu, B.; Zhu, X.; Gao, S.; Guo, J.; Wang, X.; Cao, G.; Zhu, L.; Liu, P.; Xu, H.; Chen, H.; Zhang, X.; Liu, S.; Kou, F. | Safety and efficacy of hepatic arterial infusion chemotherapy with raltitrexed and oxaliplatin post-transarterial chemoembolization for unresectable hepatocellular carcinoma | 2019 | May | J Interv Med | 2 | 2 | 91-96 | 10.1016/j.jimed.2019.07.006 |  |
| 530 | Liu, C. H.; Peng, C. M.; Hwang, J. I.; Liang, P. C.; Chen, P. J.; Abi-Jaoudeh, N.; Giiang, L. H.; Tyan, Y. S. | Phase I Dose-Escalation Study of Tirapazamine Chemoembolization for Unresectable Early- and Intermediate-Stage Hepatocellular Carcinoma | 2022 | Aug | J Vasc Interv Radiol | 33 | 8 | 926-933.e1 | 10.1016/j.jvir.2022.04.031 | Wrong study design |
| 531 | Liu, C.; Jia, B. S.; Zou, B. W.; Du, H.; Yan, L. N.; Yang, J. Y.; Jiang, L.; Wen, T. F.; Lu, W. S. | Neutrophil-to-lymphocyte and aspartate-to-alanine aminotransferase ratios predict hepatocellular carcinoma prognosis after transarterial embolization | 2017 | Nov | Medicine (Baltimore) | 96 | 45 | e8512 | 10.1097/md.0000000000008512 |  |
| 532 | Liu, C.; Li, L.; Lu, W. S.; Du, H.; Yan, L. N.; Yang, J. Y.; Wen, T. F.; Zeng, G. J.; Jiang, L.; Yang, J. | Neutrophil-lymphocyte Ratio Plus Prognostic Nutritional Index Predicts the Outcomes of Patients with Unresectable Hepatocellular Carcinoma After Transarterial Chemoembolization | 2017 | 24-Oct | Sci Rep | 7 | 1 | 13873 | 10.1038/s41598-017-13239-w |  |
| 533 | Liu, Chong; Li, Tian; He, Jin-tong; Shao, Haibo | TACE combined with microwave ablation therapy vs. TACE alone for treatment of early-and intermediate-stage hepatocellular carcinomas larger than 5 cm: a meta-analysis | 2020 |  | Diagn Interv Radiol | 26 | 6 | 575-583 | doi: 10.5152/dir.2020.19615 | Wrong study design |
| 534 | Liu, D.; Wang, J.; Ma, Z.; Zhang, N.; Zhao, Y.; Yang, X.; Wen, Z.; Xie, H. | Treatment of unresectable intrahepatic cholangiocarcinoma using transarterial chemoembolisation with irinotecan-eluting beads: analysis of efficacy and safety | 2022 | Aug | Cardiovasc Intervent Radiol | 45 | 8 | 1092-1101 | 10.1007/s00270-022-03108-z |  |
| 535 | Liu, F.; Meng, Z.; Shao, G.; Wang, J.; Wang, Z.; Yang, J.; Yip, C. S.; He, D. | Patterns of sorafenib and TACE treatment of unresectable hepatocellular carcinoma in a Chinese population: subgroup analysis of the GIDEON study | 2017 | Feb | Mol Biol Rep | 44 | 1 | 149-158 | 10.1007/s11033-016-4092-x | Wrong patient population |
| 536 | Liu, G. H.; Qiu, M. K.; Wang, Y.; Zhang, T. T.; Wang, L. J.; Guan, W. B.; Ou, J. M.; Chen, L. T. | Liver transplantation for advanced-stage primary hepatic yolk sac tumor: A case report and literature review | 2023 | 15-Dec | Medicine (Baltimore) | 102 | 50 | e35821 | 10.1097/md.0000000000035821 |  |
| 537 | Liu, J. N.; Li, J. J.; Yan, S.; Zhang, G. N.; Yi, P. S. | Corrigendum: Transarterial chemoembolization combined with lenvatinib versus transarterial chemoembolization combined with sorafenib for unresectable hepatocellular carcinoma: a systematic review and meta-analysis | 2023 |  | Front Oncol | 13 |  | 1233247 | 10.3389/fonc.2023.1233247 |  |
| 538 | Liu, J. N.; Li, J. J.; Yan, S.; Zhang, G. N.; Yi, P. S. | Transarterial chemoembolization combined with lenvatinib versus transarterial chemoembolization combined with sorafenib for unresectable hepatocellular carcinoma: A systematic review and meta-analysis | 2023 |  | Front Oncol | 13 |  | 1074793 | 10.3389/fonc.2023.1074793 |  |
| 539 | Liu, J.; Wang, D.; Zhou, J.; Wang, L.; Zhang, N.; Zhou, L.; Zeng, J.; Liu, J.; Yang, M. | N6-methyladenosine reader YTHDC2 and eraser FTO may determine hepatocellular carcinoma prognoses after transarterial chemoembolization | 2021 | May | Arch Toxicol | 95 | 5 | 1621-1629 | 10.1007/s00204-021-03021-3 |  |
| 540 | Liu, J.; Wei, S.; Yang, L.; Yu, J.; Yan, D.; Yi, P. | Efficacy and safety of transarterial chemoembolization plus lenvatinib with or without programmed death-1 inhibitors in the treatment of unresectable hepatocellular carcinoma: a systematic review and meta-analysis | 2023 | Nov | J Cancer Res Clin Oncol | 149 | 15 | 14451-14461 | 10.1007/s00432-023-05231-x |  |
| 541 | Liu, J.; Yan, J.; Zhou, C.; Ma, Q.; Jin, Q.; Yang, Z. | miR-1285-3p acts as a potential tumor suppressor miRNA via downregulating JUN expression in hepatocellular carcinoma | 2015 | Jan | Tumour Biol | 36 | 1 | 219-25 | 10.1007/s13277-014-2622-5 |  |
| 542 | Liu, J.; Zhang, J.; Wang, Y.; Shu, G.; Lou, C.; Du, Z. | HAIC versus TACE for patients with unresectable hepatocellular carcinoma: A systematic review and meta-analysis | 2022 | 23-Dec | Medicine (Baltimore) | 101 | 51 | e32390 | 10.1097/md.0000000000032390 | Wrong study design |
| 543 | Liu, Juanfang; Zhang, Wenguang; Lu, Huibin; Li, Hongbin; Zhou, Xueliang; Li, Jing; Han, Xinwei | Drug-eluting bead trans-arterial chemoembolization combined with microwave ablation therapy vs. microwave ablation alone for early stage hepatocellular carcinoma: a preliminary investigation of clinical value | 2022 |  | Journal of cancer research and clinical oncology | 148 | 7 | 1781-1788 |  |  |
| 544 | Liu, K.; Zheng, X.; Lu, D.; Tan, Y.; Hou, C.; Dai, J.; Shi, W.; Jiang, B.; Yao, Y.; Lu, Y.; Cao, Q.; Chen, R.; Zhang, W.; Xie, J.; Chen, L.; Jiang, M.; Zhang, Z.; Liu, L.; Liu, J.; Li, J.; Lv, W.; Wu, X. | A multi-institutional study to predict the benefits of DEB-TACE and molecular targeted agent sequential therapy in unresectable hepatocellular carcinoma using a radiological-clinical nomogram | 2023 | 20-Oct | Radiol Med |  |  |  | 10.1007/s11547-023-01736-0 | Wrong patient population |
| 545 | Liu, L.; Chen, H.; Wang, M.; Zhao, Y.; Cai, G.; Qi, X.; Han, G. | Combination therapy of sorafenib and TACE for unresectable HCC: a systematic review and meta-analysis | 2014 |  | PLoS One | 9 | 3 | e91124 | 10.1371/journal.pone.0091124 | Wrong patient population |
| 546 | Liu, L.; Xu, X.; Liang, X.; Zhang, X.; Wen, J.; Chen, K.; Su, X.; Ma, Y.; Teng, Z.; Lu, G.; Xu, J. | Periodic mesoporous organosilica-coated magnetite nanoparticles combined with lipiodol for transcatheter arterial chemoembolization to inhibit the progression of liver cancer | 2021 | Jun | J Colloid Interface Sci | 591 |  | 211-220 | 10.1016/j.jcis.2021.02.022 |  |
| 547 | Liu, L.; Zhang, Q.; Geng, J.; Li, S.; Zhao, S.; Zhang, X.; Hu, J.; Feng, D. | Comparison of radiofrequency ablation combined with sorafenib or sorafenib alone in patients with ECOG performance score 1: identifying optimal candidates | 2020 | May | Ann Transl Med | 8 | 9 | 583 | 10.21037/atm.2020.03.71 |  |
| 548 | Liu, M.; Liu, J.; Wang, L.; Wu, H.; Zhou, C.; Zhu, H.; Xu, N.; Xie, Y. | Association of serum microRNA expression in hepatocellular carcinomas treated with transarterial chemoembolization and patient survival | 2014 |  | PLoS One | 9 | 10 | e109347 | 10.1371/journal.pone.0109347 |  |
| 549 | Liu, Q.; Fan, D.; Adah, D.; Wu, Z.; Liu, R.; Yan, Q. T.; Zhang, Y.; Du, Z. Y.; Wang, D.; Li, Y.; Bao, S. Y.; Liu, L. P. | CRISPR/Cas9–mediated hypoxia-inducible factor–1α knockout enhances the antitumor effect of transarterial embolization in hepatocellular carcinoma | 2018 | Nov | Oncol Rep | 40 | 5 | 2547-2557 | 10.3892/or.2018.6667 |  |
| 550 | Liu, S.; Xiong, R.; Duan, C.; Tang, J.; Yin, T.; Dai, S. | PD-1 combined with lenvatinib and TACE for the transformational treatment of hepatocellular carcinoma combined with portal vein tumor thrombus: a case report and literature review | 2023 |  | Front Oncol | 13 |  | 1199143 | 10.3389/fonc.2023.1199143 |  |
| 551 | Liu, S.; Zhao, G.; Yu, G.; Guo, N.; Zhang, Y.; Li, Q.; Wang, Z. | Transcatheter arterial chemoembolization combined with low-dose apatinib in the treatment of unresectable hepatocellular carcinoma in elderly patients: Efficacy and safety | 2020 | Sep | J Cancer Res Ther | 16 | 5 | 1165-1170 | 10.4103/jcrt.JCRT_1030_19 | Wrong patient population |
| 552 | Liu, W.; Xie, Z.; Shen, K.; Jiang, L.; Liu, C.; Ge, Y.; Yu, J.; Jia, W.; Ma, J.; Chen, H. | Analysis of the safety and effectiveness of TACE combined with targeted immunotherapy in the treatment of intermediate and advanced hepatocellular carcinoma | 2023 | 27-Jul | Med Oncol | 40 | 9 | 251 | 10.1007/s12032-023-02082-x |  |
| 553 | Liu, X.; Luo, J.; Zhang, L.; Yang, F.; Peng, D. | SIB-IMRT combined with apatinib for unresectable hepatocellular carcinoma in patients with poor response to transarterial chemoembolization | 2022 | 28-Feb | Clin Res Hepatol Gastroenterol | | | 101897 | 10.1016/j.clinre.2022.101897 |  |
| 554 | Liu, X.; Luo, J.; Zhang, L.; Yang, F.; Peng, D. | SIB-IMRT combined with apatinib for unresectable hepatocellular carcinoma in patients with poor response to transarterial chemoembolization | 2022 | Jun-Jul | Clin Res Hepatol Gastroenterol | 46 | 6 | 101897 | 10.1016/j.clinre.2022.101897 |  |
| 555 | Liu, X.; Wang, X.; Luo, Y.; Wang, M.; Chen, Z.; Han, X.; Zhou, S.; Wang, J.; Kong, J.; Yu, H.; Wang, X.; Tang, X.; Guo, Q. | A 3D Tumor-Mimicking In Vitro Drug Release Model of Locoregional Chemoembolization Using Deep Learning-Based Quantitative Analyses | 2023 | Apr | Adv Sci (Weinh) | 10 | 11 | e2206195 | 10.1002/advs.202206195 |  |
| 556 | Liu, X.; Xie, Y.; Qi, X.; Xu, K. | Transcatheter arterial chemoembolization (TACE) with iRGD peptide in rabbit VX2 liver tumor | 2020 |  | J Cancer Res Ther | 16 | 7 | 1703-1709 | 10.4103/jcrt.JCRT_1411_20 |  |
| 557 | Liu, Y.; Feng, Y.; Wang, X.; Yang, X.; Hu, Y.; Li, Y.; Zhang, Q.; Huang, Y.; Shi, K.; Ran, C.; Hou, J.; Jiang, L.; Li, J.; Wang, X. | SPARC Negatively Correlates With Prognosis After Transarterial Chemoembolization and Facilitates Proliferation and Metastasis of Hepatocellular Carcinoma via ERK/MMP Signaling Pathways | 2020 |  | Front Oncol | 10 |  | 813 | 10.3389/fonc.2020.00813 |  |
| 558 | Liu, Y.; Yan, J.; Wang, F. | Effects of TACE combined with precise RT on p53 gene expression and prognosis of HCC patients | 2018 | Nov | Oncol Lett | 16 | 5 | 5733-5738 | 10.3892/ol.2018.9374 | Wrong patient population |
| 559 | Lo, C. H.; Huang, W. Y.; Lee, M. S.; Lin, K. T.; Lin, T. P.; Chang, P. Y.; Fan, C. Y.; Jen, Y. M. | Stereotactic ablative radiotherapy for unresectable hepatocellular carcinoma patients who failed or were unsuitable for transarterial chemoembolization | 2014 | Mar | Eur J Gastroenterol Hepatol | 26 | 3 | 345-52 | 10.1097/meg.0000000000000032 |  |
| 560 | Lobo, L.; Yakoub, D.; Picado, O.; Ripat, C.; Pendola, F.; Sharma, R.; ElTawil, R.; Kwon, D.; Venkat, S.; Portelance, L.; Yechieli, R. | Unresectable Hepatocellular Carcinoma: Radioembolization Versus Chemoembolization: A Systematic Review and Meta-analysis | 2016 | Nov | Cardiovasc Intervent Radiol | 39 | 11 | 1580-1588 | 10.1007/s00270-016-1426-y | Wrong patient population |
| 561 | Loffroy, R.; Favelier, S.; Cherblanc, V.; Estivalet, L. | C-arm dual-phase cone-beam CT: a revolutionary real-time imaging modality to assess drug-eluting beads TACE success in liver cancer patients | 2013 | Aug | Quant Imaging Med Surg | 3 | 4 | 196-9 | 10.3978/j.issn.2223-4292.2013.08.05 |  |
| 562 | Lokesh, K. N.; Chaudhuri, T.; Lakshmaiah, K. C.; Babu, K. G.; Dasappa, L.; Jacob, L. A.; Suresh Babu, M. C.; Rudresha, A. H.; Rajeev, L. K. | Advanced hepatocellular carcinoma: A regional cancer center experience of 48 cases | 2017 | Jul-Sep | Indian J Cancer | 54 | 3 | 526-529 | 10.4103/ijc.IJC_373_17 |  |
| 563 | Long, J.; Chen, B.; Liu, Z. | Comparative efficacy and safety of molecular targeted agents combined with transarterial chemoembolization in the treatment of unresectable hepatocellular carcinoma: a network meta-analysis | 2023 |  | Front Oncol | 13 |  | 1179431 | 10.3389/fonc.2023.1179431 |  |
| 564 | Long, J.; Liu, L.; Yang, X.; Lu, X.; Qin, L. | Impact of combining Lenvatinib with Transarterial chemoembolization for unresectable hepatocellular carcinoma | 2023 | Nov-Dec | Pak J Med Sci | 39 | 6 | 1847-1852 | 10.12669/pjms.39.6.7944 | Wrong patient population |
| 565 | Long, T.; Yang, Z.; Zeng, H.; Wu, W.; Hu, Z.; Yang, Z.; Hu, D.; Zhou, Z.; Chen, M.; Zhang, Y. | Comparable Clinical Outcomes Between Transarterial Chemoembolization or Hepatic Arterial Infusion Chemotherapy Combined with Tyrosine Kinase Inhibitors and PD-1 Inhibitors in Unresectable Hepatocellular Carcinoma | 2023 |  | J Hepatocell Carcinoma | 10 |  | 1849-1859 | 10.2147/jhc.S436211 |  |
| 566 | Long, Y.; Huang, J.; Liao, J.; Zhang, D.; Huang, Z.; He, X.; Zhang, L. | Safety and Survival Outcomes of Liver Resection following Triple Combination Conversion Therapy for Initially Unresectable Hepatocellular Carcinoma | 2023 | 17-Dec | Cancers (Basel) | 15 | 24 |  | 10.3390/cancers15245878 |  |
| 567 | Lopera Valle, J. S.; Puello Correa, D. F.; Sanín, E. | "Six-and-Twelve" Prognostic Score in Transarterial Chemoembolization-Treated Hepatocellular Carcinoma Patients | 2023 | Nov | Cureus | 15 | 11 | e49575 | 10.7759/cureus.49575 |  |
| 568 | Lu, G.; Ou, L.; Cao, M.; Hu, M. | Case Report and Literature Review of Multi-drugs Synergy and Targeted Comprehensive Treatment in Advanced Hepatocellular Carcinoma | 2023 | 1-Aug | Curr Cancer Drug Targets | |  |  | 10.2174/1568009623666230801093031 |  |
| 569 | Lu, H.; Liang, B.; Xia, X.; Zheng, C. | Efficacy and safety analysis of TACE + Donafenib + Toripalimab versus TACE + Sorafenib in the treatment of unresectable hepatocellular carcinoma: a retrospective study | 2023 | 25-Oct | BMC Cancer | 23 | 1 | 1033 | 10.1186/s12885-023-11535-5 |  |
| 570 | Lu, H.; Ye, Q.; Zheng, C.; Fan, L.; Xia, X. | Efficacy and safety analysis of TACE + sunitinib vs. sunitinib in the treatment of unresectable advanced renal cell carcinoma: a retrospective study | 2023 | 24-Mar | BMC Cancer | 23 | 1 | 270 | 10.1186/s12885-023-10754-0 |  |
| 571 | Lu, J.; Zhong, B. Y.; Zhu, H. D.; Guo, J. H.; Teng, G. J. | Embolotherapy of unresectable hepatocellular carcinoma: Eastern perspective | 2019 | Dec | Chin Clin Oncol | 8 | 6 | 60 | 10.21037/cco.2019.11.01 |  |
| 572 | Lu, L.; Zeng, J.; Wen, Z.; Tang, C.; Xu, N. | Transcatheter arterial chemoembolisation followed by three-dimensional conformal radiotherapy versus transcatheter arterial chemoembolisation alone for primary hepatocellular carcinoma in adults | 2019 | 16-Feb | Cochrane Database Syst Rev | 2 | 2 | Cd012244 | 10.1002/14651858.CD012244.pub2 |  |
| 573 | Luerken, L.; Haimerl, M.; Doppler, M.; Uller, W.; Beyer, L. P.; Stroszczynski, C.; Einspieler, I. | Update on Percutaneous Local Ablative Procedures for the Treatment of Hepatocellular Carcinoma | 2022 | Oct | Rofo | 194 | 10 | 1075-1086 | 10.1055/a-1768-0954 |  |
| 574 | Luo, J.; Huang, Z.; Wang, M.; Li, T.; Huang, J. | Prognostic role of multiparameter MRI and radiomics in progression of advanced unresectable hepatocellular carcinoma following combined transcatheter arterial chemoembolization and lenvatinib therapy | 2022 | 8-Mar | BMC Gastroenterol | 22 | 1 | 108 | 10.1186/s12876-022-02129-9 |  |
| 575 | Luo, M. C.; Wu, J. Y.; Wu, J. Y.; Lin, Z. T.; Li, Y. N.; Zeng, Z. X.; Wei, S. M.; Yan, M. L. | Early Tumor Marker Response Predicts Treatment Outcomes in Patients with Unresectable Hepatocellular Carcinoma Receiving Combined Lenvatinib, Immune Checkpoint Inhibitors, and Transcatheter Arterial Chemoembolization Therapy | 2023 |  | J Hepatocell Carcinoma | 10 |  | 1827-1837 | 10.2147/jhc.S425674 |  |
| 576 | Lv, T. R.; Hu, H. J.; Liu, F.; Regmi, P.; Jin, Y. W.; Li, F. Y. | The effect of trans arterial chemoembolization in the management of intrahepatic cholangiocarcinoma. A systematic review and meta-analysis | 2022 | May | Eur J Surg Oncol | 48 | 5 | 956-966 | 10.1016/j.ejso.2022.01.009 |  |
| 577 | Lym, J. S.; Nguyen, Q. V.; Ahn da, W.; Huynh, C. T.; Jae, H. J.; Kim, Y. I.; Lee, D. S. | Sulfamethazine-based pH-sensitive hydrogels with potential application for transcatheter arterial chemoembolization therapy | 2016 | 1-Sep | Acta Biomater | 41 |  | 253-63 | 10.1016/j.actbio.2016.05.018 |  |
| 578 | Lyu, N.; Kong, Y.; Li, X.; Guo, N.; Lai, J.; Li, J.; Zhao, M. | Effect and Safety of Prophylactic Parecoxib for Pain Control of Transarterial Chemoembolization in Liver Cancer: A Single-Center, Parallel-Group, Randomized Trial | 2022 | Jan | J Am Coll Radiol | 19 | 1 Pt A | 61-70 | 10.1016/j.jacr.2021.09.029 |  |
| 579 | Lyu, N.; Kong, Y.; Li, X.; Guo, N.; Lai, J.; Li, J.; Zhao, M. | Effect and Safety of Prophylactic Parecoxib for Pain Control of Transarterial Chemoembolization in Liver Cancer: A Single-Center, Parallel-Group, Randomized Trial | 2022 | Jan | J Am Coll Radiol | 19 | 1 Pt A | 61-70 | 10.1016/j.jacr.2021.09.029 |  |
| 580 | Ma, J.; Bo, Z.; Zhao, Z.; Yang, J.; Yang, Y.; Li, H.; Yang, Y.; Wang, J.; Su, Q.; Wang, J.; Chen, K.; Yu, Z.; Wang, Y.; Chen, G. | Machine Learning to Predict the Response to Lenvatinib Combined with Transarterial Chemoembolization for Unresectable Hepatocellular Carcinoma | 2023 | 19-Jan | Cancers (Basel) | 15 | 3 |  | 10.3390/cancers15030625 |  |
| 581 | Ma, J.; Wang, J. H. | 131I-Labeled-Metuximab Plus Transarterial Chemoembolization in Combination Therapy for Unresectable Hepatocellular Carcinoma: Results from a Multicenter Phase IV Clinical Study | 2015 |  | Asian Pac J Cancer Prev | 16 | 17 | 7441-7 | 10.7314/apjcp.2015.16.17.7441 | Wrong patient population |
| 582 | Ma, X. L.; Jiang, M.; Zhao, Y.; Wang, B. L.; Shen, M. N.; Zhou, Y.; Zhang, C. Y.; Sun, Y. F.; Chen, J. W.; Hu, B.; Gong, Z. J.; Zhang, X.; Cao, Y.; Pan, B. S.; Zhou, J.; Fan, J.; Yang, X. R.; Guo, W. | Application of Serum Annexin A3 in Diagnosis, Outcome Prediction and Therapeutic Response Evaluation for Patients with Hepatocellular Carcinoma | 2018 | Jun | Ann Surg Oncol | 25 | 6 | 1686-1694 | 10.1245/s10434-018-6402-0 |  |
| 583 | Ma, X.; Li, R. S.; Wang, J.; Huang, Y. Q.; Li, P. Y.; Wang, J.; Su, H. B.; Wang, R. L.; Zhang, Y. M.; Liu, H. H.; Zhang, C. E.; Ma, Z. J.; Wang, J. B.; Zhao, Y. L.; Xiao, X. H. | The Therapeutic Efficacy and Safety of Compound Kushen Injection Combined with Transarterial Chemoembolization in Unresectable Hepatocellular Carcinoma: An Update Systematic Review and Meta-Analysis | 2016 |  | Front Pharmacol | 7 |  | 70 | 10.3389/fphar.2016.00070 |  |
| 584 | Ma, Y. N.; Jiang, X.; Liu, H.; Song, P.; Tang, W. | Conversion therapy for initially unresectable hepatocellular carcinoma: Current status and prospects | 2023 | 23-Dec | Biosci Trends |  |  |  | 10.5582/bst.2023.01322 |  |
| 585 | Ma, Y.; Duan, L.; Li, L.; Lu, W.; Li, B.; Chen, X. | (131)Iodine-DEM TACE vs. conventional TACE in cirrhotic patients with hepatocellular carcinoma: a single center experiment | 2021 | Apr | J Gastrointest Oncol | 12 | 2 | 762-769 | 10.21037/jgo-21-105 | Wrong patient population |
| 586 | Maeda, N.; Osuga, K.; Higashihara, H.; Tomoda, K.; Mikami, K.; Nakazawa, T.; Nakamura, H.; Tomiyama, N. | Transarterial chemoembolization with cisplatin as second-line treatment for hepatocellular carcinoma unresponsive to chemoembolization with epirubicin-Lipiodol emulsion | 2012 | Feb | Cardiovasc Intervent Radiol | 35 | 1 | 82-9 | 10.1007/s00270-010-0086-6 |  |
| 587 | Mahnken, A. H. | (90)Y-glass microspheres for hepatic neoplasia | 2015 |  | Future Oncol | 11 | 9 | 1343-54 | 10.2217/fon.15.12 |  |
| 588 | Mahnken, A. H. | Current status of transarterial radioembolization | 2016 | 28-May | World J Radiol | 8 | 5 | 449-59 | 10.4329/wjr.v8.i5.449 |  |
| 589 | Makary, M. S.; Khandpur, U.; Cloyd, J. M.; Mumtaz, K.; Dowell, J. D. | Locoregional Therapy Approaches for Hepatocellular Carcinoma: Recent Advances and Management Strategies | 2020 | 15-Jul | Cancers (Basel) | 12 | 7 |  | 10.3390/cancers12071914 |  |
| 590 | Makary, M. S.; Ramsell, S.; Miller, E.; Beal, E. W.; Dowell, J. D. | Hepatocellular carcinoma locoregional therapies: Outcomes and future horizons | 2021 | 21-Nov | World J Gastroenterol | 27 | 43 | 7462-7479 | 10.3748/wjg.v27.i43.7462 |  |
| 591 | Malagari, K.; Pomoni, M.; Moschouris, H.; Bouma, E.; Koskinas, J.; Stefaniotou, A.; Marinis, A.; Kelekis, A.; Alexopoulou, E.; Chatziioannou, A.; Chatzimichael, K.; Dourakis, S.; Kelekis, N.; Rizos, S.; Kelekis, D. | Chemoembolization with doxorubicin-eluting beads for unresectable hepatocellular carcinoma: five-year survival analysis | 2012 | Oct | Cardiovasc Intervent Radiol | 35 | 5 | 1119-28 | 10.1007/s00270-012-0394-0 |  |
| 592 | Malfertheiner, P.; Verslype, C.; Kolligs, F. T.; Schütte, K.; Vandecaveye, V.; Paprottka, P. M.; Ricke, J. | The effectiveness of selective internal radiation therapy in challenging cases of liver-predominant unresectable hepatocellular carcinoma | 2014 | Nov | Future Oncol | 10 | 15 Suppl | 17-27 | 10.2217/fon.14.218 |  |
| 593 | Manas, D.; Bell, J. K.; Mealing, S.; Davies, H.; Baker, H.; Holmes, H.; Hubner, R. A. | The cost-effectiveness of TheraSphere in patients with hepatocellular carcinoma who are eligible for transarterial embolization | 2021 | Feb | Eur J Surg Oncol | 47 | 2 | 401-408 | 10.1016/j.ejso.2020.08.027 |  |
| 594 | Manjunatha, N.; Ganduri, V.; Rajasekaran, K.; Duraiyarasan, S.; Adefuye, M. | Transarterial Chemoembolization and Unresectable Hepatocellular Carcinoma: A Narrative Review | 2022 | Aug | Cureus | 14 | 8 | e28439 | 10.7759/cureus.28439 |  |
| 595 | Mansour, M. A.; Khalifa, M. O. | Antibiotic prophylaxis in transarterial chemoembolization of hepatocellular carcinoma | 2018 | Mar | Arab J Gastroenterol | 19 | 1 | 16-20 | 10.1016/j.ajg.2018.02.002 |  |
| 596 | Maraj, T.; Mirrahimi, A.; Dey, C. | Survival of Patients with Colorectal Liver Metastases after Transarterial Chemoembolization Using Irinotecan-Eluting Microspheres: A Single-Center Retrospective Analysis Comparing RECIST 1.1 and Choi Criteria | 2023 | Jun | J Vasc Interv Radiol | 34 | 6 | 983-990.e1 | 10.1016/j.jvir.2023.02.005 |  |
| 597 | Marti, J.; Giacca, M.; Alshebeeb, K.; Bahl, S.; Hua, C.; Horn, J. C.; BouAyache, J.; Patel, R.; Facciuto, M.; Schwartz, M.; Florman, S.; Kim, E.; Gunasekaran, G. | Analysis of Preoperative Portal Vein Embolization Outcomes in Patients with Hepatocellular Carcinoma: A Single-Center Experience | 2018 | Jul | J Vasc Interv Radiol | 29 | 7 | 920-926 | 10.1016/j.jvir.2018.01.780 |  |
| 598 | Mason, M. C.; Massarweh, N. N.; Salami, A.; Sultenfuss, M. A.; Anaya, D. A. | Post-embolization syndrome as an early predictor of overall survival after transarterial chemoembolization for hepatocellular carcinoma | 2015 | Dec | HPB (Oxford) | 17 | 12 | 1137-44 | 10.1111/hpb.12487 |  |
| 599 | Massani, M.; Stecca, T.; Ruffolo, C.; Bassi, N. | Should we routinely use DEBTACE for unresectable HCC? cTACE versus DEBTACE: a single-center survival analysis | 2017 | Mar | Updates Surg | 69 | 1 | 67-73 | 10.1007/s13304-017-0414-3 |  |
| 600 | Matsumoto, T.; Endo, J.; Hashida, K.; Ichikawa, H.; Kojima, S.; Takashimizu, S.; Watanabe, N.; Yamagami, T.; Hasebe, T. | Balloon-occluded transarterial chemoembolization using a 1.8-French tip coaxial microballoon catheter for hepatocellular carcinoma: technical and safety considerations | 2015 | Apr | Minim Invasive Ther Allied Technol | 24 | 2 | 94-100 | 10.3109/13645706.2014.951657 |  |
| 601 | Matsumoto, T.; Endo, J.; Hashida, K.; Mizukami, H.; Nagata, J.; Ichikawa, H.; Kojima, S.; Takashimizu, S.; Yamagami, T.; Watanabe, N.; Hasebe, T. | Balloon-occluded arterial stump pressure before balloon-occluded transarterial chemoembolization | 2016 |  | Minim Invasive Ther Allied Technol | 25 | 1 | 22-8 | 10.3109/13645706.2015.1086381 |  |
| 602 | Mazioti, A.; Gatselis, N. K.; Rountas, C.; Zachou, K.; Filippiadis, D. K.; Tepetes, K.; Koukoulis, G. K.; Fezoulidis, I.; Dalekos, G. N. | Safety and efficacy of transcatheter arterial chemoemboliazation in the real-life management of unresectable hepatocellular carcinoma | 2013 |  | Hepat Mon | 13 | 8 | e7070 | 10.5812/hepatmon.7070 | Wrong outcomes |
| 603 | Mazzaferro, V.; Gorgen, A.; Roayaie, S.; Droz Dit Busset, M.; Sapisochin, G. | Liver resection and transplantation for intrahepatic cholangiocarcinoma | 2020 | Feb | J Hepatol | 72 | 2 | 364-377 | 10.1016/j.jhep.2019.11.020 |  |
| 604 | McNally, M. E.; Martinez, A.; Khabiri, H.; Guy, G.; Michaels, A. J.; Hanje, J.; Kirkpatrick, R.; Bloomston, M.; Schmidt, C. R. | Inflammatory markers are associated with outcome in patients with unresectable hepatocellular carcinoma undergoing transarterial chemoembolization | 2013 | Mar | Ann Surg Oncol | 20 | 3 | 923-8 | 10.1245/s10434-012-2639-1 |  |
| 605 | Mearini, L. | High intensity focused ultrasound, liver disease and bridging therapy | 2013 | 21-Nov | World J Gastroenterol | 19 | 43 | 7494-9 | 10.3748/wjg.v19.i43.7494 |  |
| 606 | Megías Vericat, J. E.; García Marcos, R.; López Briz, E.; Gómez Muñoz, F.; Ramos Ruiz, J.; Martínez Rodrigo, J. J.; Poveda Andrés, J. L. | Trans-arterial chemoembolization with doxorubicin-eluting particles versus conventional trans-arterial chemoembolization in unresectable hepatocellular carcinoma: A study of effectiveness, safety and costs | 2015 | Nov-Dec | Radiologia | 57 | 6 | 496-504 | 10.1016/j.rx.2015.01.008 | Wrong patient population |
| 607 | Mei, J.; Yu, H.; Qin, L.; Jia, Z. | FOLFOX-HAIC for Unresectable Large Hepatocellular Carcinoma: The Effectiveness Has Yet to be Determined | 2022 | 1-Jun | J Clin Oncol | 40 | 16 | 1841 | 10.1200/jco.21.02533 |  |
| 608 | Melchiorre, F.; Patella, F.; Pescatori, L.; Pesapane, F.; Fumarola, E.; Biondetti, P.; Brambillasca, P.; Monaco, C.; Ierardi, A. M.; Franceschelli, G.; Carrafiello, G. | DEB-TACE: a standard review | 2018 | Dec | Future Oncol | 14 | 28 | 2969-2984 | 10.2217/fon-2018-0136 |  |
| 609 | Meng, X. P.; Wang, Y. C.; Ju, S.; Lu, C. Q.; Zhong, B. Y.; Ni, C. F.; Zhang, Q.; Yu, Q.; Xu, J.; Ji, J.; Zhang, X. M.; Tang, T. Y.; Yang, G.; Zhao, Z. | Radiomics Analysis on Multiphase Contrast-Enhanced CT: A Survival Prediction Tool in Patients With Hepatocellular Carcinoma Undergoing Transarterial Chemoembolization | 2020 |  | Front Oncol | 10 |  | 1196 | 10.3389/fonc.2020.01196 |  |
| 610 | Meyer, T.; Fox, R.; Ma, Y. T.; Ross, P. J.; James, M. W.; Sturgess, R.; Stubbs, C.; Stocken, D. D.; Wall, L.; Watkinson, A.; Hacking, N.; Evans, T. R. J.; Collins, P.; Hubner, R. A.; Cunningham, D.; Primrose, J. N.; Johnson, P. J.; Palmer, D. H. | Sorafenib in combination with transarterial chemoembolisation in patients with unresectable hepatocellular carcinoma (TACE 2): a randomised placebo-controlled, double-blind, phase 3 trial | 2017 | Aug | Lancet Gastroenterol Hepatol | 2 | 8 | 565-575 | 10.1016/s2468-1253(17)30156-5 | Wrong patient population |
| 611 | Minami, Y.; Kudo, M. | Imaging Modalities for Assessment of Treatment Response to Nonsurgical Hepatocellular Carcinoma Therapy: Contrast-Enhanced US, CT, and MRI | 2015 | Mar | Liver Cancer | 4 | 2 | 106-14 | 10.1159/000367733 |  |
| 612 | Mo, A.; Velten, C.; Jiang, J. M.; Tang, J.; Ohri, N.; Kalnicki, S.; Mirhaji, P.; Nemoto, K.; Aasman, B.; Garg, M.; Guha, C.; Brodin, N. P.; Kabarriti, R. | Improving Adjuvant Liver-Directed Treatment Recommendations for Unresectable Hepatocellular Carcinoma: An Artificial Intelligence-Based Decision-Making Tool | 2022 | Jun | JCO Clin Cancer Inform | 6 |  | e2200024 | 10.1200/cci.22.00024 |  |
| 613 | Mohnike, K.; Steffen, I. G.; Seidensticker, M.; Hass, P.; Damm, R.; Peters, N.; Seidensticker, R.; Schütte, K.; Arend, J.; Bornschein, J.; Streitparth, T.; Wybranski, C.; Wieners, G.; Stübs, P.; Malfertheiner, P.; Pech, M.; Ricke, J. | Radioablation by Image-Guided (HDR) Brachytherapy and Transarterial Chemoembolization in Hepatocellular Carcinoma: A Randomized Phase II Trial | 2019 | Feb | Cardiovasc Intervent Radiol | 42 | 2 | 239-249 | 10.1007/s00270-018-2127-5 |  |
| 614 | Molla, N.; AlMenieir, N.; Simoneau, E.; Aljiffry, M.; Valenti, D.; Metrakos, P.; Boucher, L. M.; Hassanain, M. | The role of interventional radiology in the management of hepatocellular carcinoma | 2014 | Jun | Curr Oncol | 21 | 3 | e480-92 | 10.3747/co.21.1829 |  |
| 615 | Moreno-Luna, L. E.; Yang, J. D.; Sanchez, W.; Paz-Fumagalli, R.; Harnois, D. M.; Mettler, T. A.; Gansen, D. N.; de Groen, P. C.; Lazaridis, K. N.; Narayanan Menon, K. V.; Larusso, N. F.; Alberts, S. R.; Gores, G. J.; Fleming, C. J.; Slettedahl, S. W.; Harmsen, W. S.; Therneau, T. M.; Wiseman, G. A.; Andrews, J. C.; Roberts, L. R. | Efficacy and safety of transarterial radioembolization versus chemoembolization in patients with hepatocellular carcinoma | 2013 | Jun | Cardiovasc Intervent Radiol | 36 | 3 | 714-23 | 10.1007/s00270-012-0481-2 |  |
| 616 | Morimoto, M.; Kobayashi, S.; Moriya, S.; Ueno, M.; Tezuka, S.; Irie, K.; Goda, Y.; Ohkawa, S. | Short-term efficacy of transarterial chemoembolization with epirubicin-loaded superabsorbent polymer microspheres for hepatocellular carcinoma: comparison with conventional transarterial chemoembolization | 2017 | Feb | Abdom Radiol (NY) | 42 | 2 | 612-619 | 10.1007/s00261-016-0900-y | Wrong patient population |
| 617 | Morimoto, M.; Numata, K.; Kondo, M.; Moriya, S.; Morita, S.; Maeda, S.; Tanaka, K. | Radiofrequency ablation combined with transarterial chemoembolization for subcapsular hepatocellular carcinoma: a prospective cohort study | 2013 | Mar | Eur J Radiol | 82 | 3 | 497-503 | 10.1016/j.ejrad.2012.09.014 |  |
| 618 | Morisco, F.; Camera, S.; Guarino, M.; Tortora, R.; Cossiga, V.; Vitiello, A.; Cordone, G.; Caporaso, N.; Di Costanzo, G. G. | Laser ablation is superior to TACE in large-sized hepatocellular carcinoma: a pilot case-control study | 2018 | 3-Apr | Oncotarget | 9 | 25 | 17483-17490 | 10.18632/oncotarget.24756 |  |
| 619 | Morishita, A.; Tani, J.; Nomura, T.; Takuma, K.; Nakahara, M.; Oura, K.; Tadokoro, T.; Fujita, K.; Shi, T.; Yamana, H.; Matsui, T.; Takata, T.; Sanomura, T.; Nishiyama, Y.; Himoto, T.; Tomonari, T.; Moriya, A.; Senoo, T.; Takaguchi, K.; Masaki, T. | Efficacy of Combined Therapy with Drug-Eluting Beads-Transcatheter Arterial Chemoembolization Followed by Conventional Transcatheter Arterial Chemoembolization for Unresectable Hepatocellular Carcinoma: A Multi-Center Study | 2021 | 14-Sep | Cancers (Basel) | 13 | 18 |  | 10.3390/cancers13184605 |  |
| 620 | Morse, M. A.; Hanks, B. A.; Suhocki, P.; Doan, P. L.; Liu, E. A.; Frost, P.; Bernard, S. A.; Tsai, A.; Moore, D. T.; O'Neil, B. H. | Improved time to progression for transarterial chemoembolization compared with transarterial embolization for patients with unresectable hepatocellular carcinoma | 2012 | Sep | Clin Colorectal Cancer | 11 | 3 | 185-90 | 10.1016/j.clcc.2011.11.003 |  |
| 621 | Mosconi, C.; Gramenzi, A.; Biselli, M.; Cappelli, A.; Bruno, A.; De Benedittis, C.; Cucchetti, A.; Modestino, F.; Peta, G.; Bianchi, G.; Trevisani, F.; Golfieri, R. | Survival and Tolerability of Transarterial Chemoembolization in Greater Versus less than 70 Years of Age Patients with Unresectable Hepatocellular Carcinoma: A Propensity Score Analysis | 2020 | Jul | Cardiovasc Intervent Radiol | 43 | 7 | 1015-1024 | 10.1007/s00270-020-02451-3 | Wrong patient population |
| 622 | Mosconi, C.; Gramenzi, A.; Cappelli, A.; Biselli, M.; Golfieri, R. | Reply to "Re: Survival and Tolerability of Transarterial Chemoembolization in Greater Versus less than 70 Years of Age Patients with Unresectable Hepatocellular Carcinoma-A Propensity Analysis" | 2021 | Jul | Cardiovasc Intervent Radiol | 44 | 7 | 1135-1136 | 10.1007/s00270-021-02799-0 |  |
| 623 | Mouli, S. K.; Goff, L. W. | Local Arterial Therapies in the Management of Unresectable Hepatocellular Carcinoma | 2017 | 27-Oct | Curr Treat Options Oncol | 18 | 11 | 67 | 10.1007/s11864-017-0509-6 |  |
| 624 | Mu, C.; Shen, J.; Zhu, X.; Peng, W.; Zhang, X.; Wen, T. | The efficacy and safety of lenvatinib plus transarterial chemoembolization in combination with PD-1 antibody in treatment of unresectable recurrent hepatocellular carcinoma: a case series report | 2023 |  | Front Oncol | 13 |  | 1096955 | 10.3389/fonc.2023.1096955 |  |
| 625 | Muhammad, A.; Dhamija, M.; Vidyarthi, G.; Amodeo, D.; Boyd, W.; Miladinovic, B.; Kumar, A. | Comparative effectiveness of traditional chemoembolization with or without sorafenib for hepatocellular carcinoma | 2013 | 27-Jul | World J Hepatol | 5 | 7 | 364-71 | 10.4254/wjh.v5.i7.364 | Wrong patient population |
| 626 | Mumdzhiev, N.; Borisov, B.; Tenev, R.; Radicheva, M. | Treating unresectable intrahepatic cholangiocarcinoma with transarterial chemoembolization and an unusual progression with cardiac involvement | 2023 | 30-Apr | Folia Med (Plovdiv) | 65 | 2 | 326-330 | 10.3897/folmed.65.e76329 |  |
| 627 | Murata, S.; Mine, T.; Sugihara, F.; Yasui, D.; Yamaguchi, H.; Ueda, T.; Onozawa, S.; Kumita, S. | Interventional treatment for unresectable hepatocellular carcinoma | 2014 | 7-Oct | World J Gastroenterol | 20 | 37 | 13453-65 | 10.3748/wjg.v20.i37.13453 |  |
| 628 | Muszbek, N.; Remak, E.; Evans, R.; Brennan, V. K.; Colaone, F.; Shergill, S.; Mullan, D.; Ross, P. J. | Cost-utility analysis of selective internal radiation therapy with Y-90 resin microspheres in hepatocellular carcinoma | 2021 | Mar | Future Oncol | 17 | 9 | 1055-1068 | 10.2217/fon-2020-1004 |  |
| 629 | Nagamatsu, H.; Sumie, S.; Niizeki, T.; Tajiri, N.; Iwamoto, H.; Aino, H.; Nakano, M.; Shimose, S.; Satani, M.; Okamura, S.; Kuromatsu, R.; Matsugaki, S.; Kurogi, J.; Kajiwara, M.; Koga, H.; Torimura, T. | Hepatic arterial infusion chemoembolization therapy for advanced hepatocellular carcinoma: multicenter phase II study | 2016 | Feb | Cancer Chemother Pharmacol | 77 | 2 | 243-50 | 10.1007/s00280-015-2892-7 |  |
| 630 | Nakasumi, K.; Yamamoto, N.; Takami, T.; Itoh, H.; Itamoto, K.; Horikirizono, H.; Iseri, T.; Nakaichi, M.; Nemoto, Y.; Sunahara, H.; Tani, K. | Effect of drug-eluting bead transarterial chemoembolization loaded with cisplatin on normal dogs | 2022 | 13-Jan | J Vet Med Sci | 84 | 1 | 114-120 | 10.1292/jvms.21-0396 |  |
| 631 | Nakasumi, K.; Yamamoto, N.; Takami, T.; Itoh, H.; Itamoto, K.; Horikirizono, H.; Iseri, T.; Nakaichi, M.; Nemoto, Y.; Sunahara, H.; Tani, K. | Effect of drug-eluting bead transarterial chemoembolization loaded with cisplatin on normal dogs | 2022 | 13-Jan | J Vet Med Sci | 84 | 1 | 114-120 | 10.1292/jvms.21-0396 |  |
| 632 | Nam, J. Y.; Choe, A. R.; Sinn, D. H.; Lee, J. H.; Kim, H. Y.; Yu, S. J.; Kim, Y. J.; Yoon, J. H.; Lee, J. M.; Chung, J. W.; Choi, S. Y.; Lee, J. K.; Baek, S. Y.; Lee, H. A.; Kim, T. H.; Yoo, K. | A differential risk assessment and decision model for Transarterial chemoembolization in hepatocellular carcinoma based on hepatic function | 2020 | 1-Jun | BMC Cancer | 20 | 1 | 504 | 10.1186/s12885-020-06975-2 |  |
| 633 | Naorungroj, T.; Naksanguan, T.; Chinthammitr, Y. | Pulmonary lipiodol embolism after transcatheter arterial chemoembolization for hepatocellular carcinoma: a case report and literature review | 2013 | Feb | J Med Assoc Thai | 96 Suppl 2 |  | S270-5 |  |  |
| 634 | Narang-Master, J.; Rizzolo, D. | Managing localized unresectable hepatocellular carcinoma | 2015 | Jan | Jaapa | 28 | 1 | 27-34 | 10.1097/01.JAA.0000458854.46279.d8 |  |
| 635 | Naugler, W. E. | Battle Royale: Systemic Versus Locoregional Therapy for Unresectable Hepatocellular Carcinoma | 2018 | Jun | AJR Am J Roentgenol | 210 | 6 | 1366-1367 | 10.2214/ajr.17.19392 |  |
| 636 | Negussie, A. H.; Dreher, M. R.; Johnson, C. G.; Tang, Y.; Lewis, A. L.; Storm, G.; Sharma, K. V.; Wood, B. J. | Synthesis and characterization of image-able polyvinyl alcohol microspheres for image-guided chemoembolization | 2015 | Jun | J Mater Sci Mater Med | 26 | 6 | 198 | 10.1007/s10856-015-5530-3 |  |
| 637 | Newell, P. H.; Wu, Y.; Hoen, H.; Uppal, R.; Thiesing, J. T.; Sasadeusz, K.; Cassera, M. A.; Wolf, R. F.; Hansen, P.; Hammill, C. W. | Multimodal treatment of unresectable hepatocellular carcinoma to achieve complete response results in improved survival | 2015 | May | HPB (Oxford) | 17 | 5 | 454-60 | 10.1111/hpb.12377 |  |
| 638 | Ni, S.; Liu, L.; Shu, Y. | Sequential transcatheter arterial chemoembolization, three-dimensional conformal radiotherapy, and high-intensity focused ultrasound treatment for unresectable hepatocellular carcinoma patients | 2012 | Jul | J Biomed Res | 26 | 4 | 260-7 | 10.7555/jbr.26.20120016 | Wrong patient population |
| 639 | Niessen, C.; Unterpaintner, E.; Goessmann, H.; Schlitt, H. J.; Mueller-Schilling, M.; Wohlgemuth, W. A.; Stroszczynski, C.; Wiggermann, P. | Degradable starch microspheres versus ethiodol and doxorubicin in transarterial chemoembolization of hepatocellular carcinoma | 2014 | Feb | J Vasc Interv Radiol | 25 | 2 | 240-7 | 10.1016/j.jvir.2013.10.007 |  |
| 640 | Nishi, M.; Saeki, I.; Yamasaki, T.; Maeda, M.; Hisanaga, T.; Iwamoto, T.; Matsumoto, T.; Hidaka, I.; Ishikawa, T.; Takami, T.; Sakaida, I. | Hemobilia immediately after transcatheter arterial chemoembolization using drug-eluting beads for hepatocellular carcinoma with intrahepatic bile duct invasion | 2018 | Mar | Hepatol Res | 48 | 4 | 329-332 | 10.1111/hepr.12995 |  |
| 641 | Nishida, K.; Lefor, A. K.; Funabiki, T. | Rupture of Hepatocellular Carcinoma after Transarterial Chemoembolization followed by Massive Gastric Bleeding | 2018 |  | Case Reports Hepatol | 2018 |  | 4576276 | 10.1155/2018/4576276 |  |
| 642 | Nojiri, S.; Kusakabe, A.; Fujiwara, K.; Shinkai, N.; Matsuura, K.; Iio, E.; Miyaki, T.; Nomura, T.; Sobue, S.; Sano, H.; Hasegawa, I.; Ohno, T.; Takahashi, Y.; Orito, E.; Joh, T. | Clinical factors related to long-term administration of sorafenib in patients with hepatocellular carcinoma | 2012 |  | Cancer Manag Res | 4 |  | 423-9 | 10.2147/cmar.S38684 |  |
| 643 | Nong, X.; Zhang, Y. M.; Liang, J. C.; Xie, J. L.; Zhang, Z. M. | Complete response by patients with advanced hepatocellular carcinoma after combination immune/targeted therapy and transarterial chemoembolization: two case reports and literature review | 2022 | Aug | Transl Cancer Res | 11 | 8 | 2973-2984 | 10.21037/tcr-21-2691 |  |
| 644 | Nosaka, T.; Matsuda, H.; Sugata, R.; Akazawa, Y.; Takahashi, K.; Naito, T.; Ohtani, M.; Kinoshita, K.; Tsujikawa, T.; Sato, Y.; Maeda, Y.; Tamamura, H.; Nakamoto, Y. | Longer Survival and Preserved Liver Function after Proton Beam Therapy for Patients with Unresectable Hepatocellular Carcinoma | 2023 | 30-Mar | Curr Oncol | 30 | 4 | 3915-3926 | 10.3390/curroncol30040296 |  |
| 645 | Nouri, Y. M.; Kim, J. H.; Yoon, H. K.; Ko, H. K.; Shin, J. H.; Gwon, D. I. | Update on Transarterial Chemoembolization with Drug-Eluting Microspheres for Hepatocellular Carcinoma | 2019 | Jan | Korean J Radiol | 20 | 1 | 34-49 | 10.3348/kjr.2018.0088 |  |
| 646 | Odagiri, N.; Hai, H.; Thuy, L. T. T.; Dong, M. P.; Suoh, M.; Kotani, K.; Hagihara, A.; Uchida-Kobayashi, S.; Tamori, A.; Enomoto, M.; Kawada, N. | Early Change in the Plasma Levels of Circulating Soluble Immune Checkpoint Proteins in Patients with Unresectable Hepatocellular Carcinoma Treated by Lenvatinib or Transcatheter Arterial Chemoembolization | 2020 | 24-Jul | Cancers (Basel) | 12 | 8 |  | 10.3390/cancers12082045 |  |
| 647 | Odagiri, N.; Tamori, A.; Kotani, K.; Motoyama, H.; Kawamura, E.; Hagihara, A.; Fujii, H.; Uchida-Kobayashi, S.; Enomoto, M.; Kawada, N. | A case of hepatocellular carcinoma with "pseudoprogression" followed by complete response to atezolizumab plus bevacizumab | 2023 | Jun | Clin J Gastroenterol | 16 | 3 | 392-396 | 10.1007/s12328-023-01761-6 |  |
| 648 | Ogasawara, S.; Ooka, Y.; Koroki, K.; Maruta, S.; Kanzaki, H.; Kanayama, K.; Kobayashi, K.; Kiyono, S.; Nakamura, M.; Kanogawa, N.; Saito, T.; Kondo, T.; Suzuki, E.; Nakamoto, S.; Tawada, A.; Chiba, T.; Arai, M.; Kato, J.; Kato, N. | Switching to systemic therapy after locoregional treatment failure: Definition and best timing | 2020 | Apr | Clin Mol Hepatol | 26 | 2 | 155-162 | 10.3350/cmh.2019.0021n |  |
| 649 | Ogawa, K.; Kamimura, K.; Watanabe, Y.; Motai, Y.; Kumaki, D.; Seki, R.; Sakamaki, A.; Abe, S.; Kawai, H.; Suda, T.; Yamagiwa, S.; Terai, S. | Effect of double platinum agents, combination of miriplatin-transarterial oily chemoembolization and cisplatin-hepatic arterial infusion chemotherapy, in patients with hepatocellular carcinoma: Report of two cases | 2017 | 16-Jun | World J Clin Cases | 5 | 6 | 238-246 | 10.12998/wjcc.v5.i6.238 |  |
| 650 | Okabe, K.; Beppu, T.; Masuda, T.; Hayashi, H.; Okabe, H.; Komori, H.; Horino, K.; Sugiyama, S.; Ishiko, T.; Takamori, H.; Yamanaka, T.; Baba, H. | Portal vein embolization can prevent intrahepatic metastases to non-embolized liver | 2012 | Mar-Apr | Hepatogastroenterology | 59 | 114 | 538-41 | 10.5754/hge09764 |  |
| 651 | Okimoto, K.; Ogasawara, S.; Chiba, T.; Ooka, Y.; Oobu, M.; Azemoto, R.; Kanogawa, N.; Motoyama, T.; Suzuki, E.; Tawada, A.; Yoshikawa, M.; Yokosuka, O. | Efficacy of transcatheter arterial chemoembolization with miriplatin-lipiodol water-soluble contrast agent emulsion in patients with hepatocellular carcinoma | 2013 | Dec | Anticancer Res | 33 | 12 | 5603-9 |  | Wrong patient population |
| 652 | Orlacchio, A.; Chegai, F.; Francioso, S.; Merolla, S.; Monti, S.; Angelico, M.; Tisone, G.; Mannelli, L. | Repeated Transarterial Chemoembolization with Degradable Starch "¨Microspheres (DSMs-TACE) of Unresectable Hepatocellular Carcinoma: A Prospective Pilot Study | 2018 | Aug | Curr Med Imaging Rev | 14 | 4 | 637-645 | 10.2174/1573405613666170616123657 | Wrong patient population |
| 653 | Orlacchio, A.; Chegai, F.; Roma, S.; Merolla, S.; Bosa, A.; Francioso, S. | Degradable starch microspheres transarterial chemoembolization (DSMs-TACE) in patients with unresectable hepatocellular carcinoma (HCC): long-term results from a single-center 137-patient cohort prospective study | 2020 | Jan | Radiol Med | 125 | 1 | 98-106 | 10.1007/s11547-019-01093-x | Wrong patient population |
| 654 | Orlacchio, A.; Gasparrini, F.; Lenci, I.; Gagliardi, M. G.; Spada, M.; Guazzaroni, M.; Ciccarese, G.; Angelico, M. | Transarterial chemoembolization for hepatocellular carcinoma in Fontan surgery patient | 2020 | Dec | Radiol Case Rep | 15 | 12 | 2602-2606 | 10.1016/j.radcr.2020.09.056 |  |
| 655 | Ortega López, N. | PET/Computed Tomography in Evaluation of Transarterial Chemoembolization | 2015 | Oct | PET Clin | 10 | 4 | 507-17 | 10.1016/j.cpet.2015.05.006 |  |
| 656 | Osuga, K.; Arai, Y.; Anai, H.; Takeuchi, Y.; Aramaki, T.; Sugihara, E.; Yamamoto, T.; Inaba, Y.; Ganaha, F.; Seki, H.; Sadaoka, S.; Sato, M.; Kobayashi, T.; Kodama, Y.; Inoh, S.; Yamakado, K. | Phase I/II multicenter study of transarterial chemoembolization with a cisplatin fine powder and porous gelatin particles for unresectable hepatocellular carcinoma: Japan Interventional Radiology in Oncology Study Group Study 0401 | 2012 | Oct | J Vasc Interv Radiol | 23 | 10 | 1278-85 | 10.1016/j.jvir.2012.06.028 | Wrong patient population |
| 657 | Oura, K.; Morishita, A.; Hamaya, S.; Fujita, K.; Masaki, T. | The Roles of Epigenetic Regulation and the Tumor Microenvironment in the Mechanism of Resistance to Systemic Therapy in Hepatocellular Carcinoma | 2023 | 1-Feb | Int J Mol Sci | 24 | 3 |  | 10.3390/ijms24032805 |  |
| 658 | Oura, K.; Takuma, K.; Nakahara, M.; Tadokoro, T.; Fujita, K.; Mimura, S.; Tani, J.; Morishita, A.; Kobara, H.; Masaki, T. | Multimodal treatment involving molecular targeted agents and on-demand transcatheter arterial chemoembolization for advanced hepatocellular carcinoma: A case report and review of the literature | 2021 | Aug | Mol Clin Oncol | 15 | 2 | 154 | 10.3892/mco.2021.2316 |  |
| 659 | Ouyang, T.; Liu, J.; Shi, C.; Zhu, L.; Guo, X. | Drug-Eluting Bead Transarterial Chemoembolization versus Conventional Transarterial Chemoembolization Both Combined Apatinib for Hepatocellular Carcinoma: A Retrospective, Propensity-Score Matched Study | 2021 |  | J Hepatocell Carcinoma | 8 |  | 1459-1471 | 10.2147/jhc.S338309 | Article inaccessible |
| 660 | Padia, S. A.; Johnson, G. E.; Horton, K. J.; Ingraham, C. R.; Kogut, M. J.; Kwan, S.; Vaidya, S.; Monsky, W. L.; Park, J. O.; Bhattacharya, R.; Hippe, D. S.; Harris, W. P. | Segmental Yttrium-90 Radioembolization versus Segmental Chemoembolization for Localized Hepatocellular Carcinoma: Results of a Single-Center, Retrospective, Propensity Score-Matched Study | 2017 | Jun | J Vasc Interv Radiol | 28 | 6 | 777-785.e1 | 10.1016/j.jvir.2017.02.018 | Wrong patient population |
| 661 | Pai, Y. W.; Hsieh, P. F.; Tung, H.; Wu, C. Y.; Ching, C. T.; Chang, M. H. | Prognosis of cerebral lipiodol embolism caused by transarterial chemoembolization | 2016 | Oct | Neurol Res | 38 | 10 | 857-63 | 10.1080/01616412.2016.1201928 |  |
| 662 | Palmer, D. H.; Malagari, K.; Kulik, L. M. | Role of locoregional therapies in the wake of systemic therapy | 2020 | Feb | J Hepatol | 72 | 2 | 277-287 | 10.1016/j.jhep.2019.09.023 |  |
| 663 | Pan, X.; Wu, S. J.; Tang, Y.; Zhou, Y. F.; Luo, J. W.; Fang, Z. T. | Safety and Efficacy of Transarterial Chemoembolization Combined with Tyrosine Kinase Inhibitor and Immune Checkpoint Inhibitors for Unresectable Hepatocellular Carcinoma: A Single Center Experience | 2023 |  | J Hepatocell Carcinoma | 10 |  | 883-892 | 10.2147/jhc.S404500 |  |
| 664 | Pang, Y.; Eresen, A.; Zhang, Z.; Hou, Q.; Wang, Y.; Yaghmai, V.; Zhang, Z. | Adverse events of sorafenib in hepatocellular carcinoma treatment | 2022 |  | Am J Cancer Res | 12 | 6 | 2770-2782 |  |  |
| 665 | Park, J. W.; Amarapurkar, D.; Chao, Y.; Chen, P. J.; Geschwind, J. F.; Goh, K. L.; Han, K. H.; Kudo, M.; Lee, H. C.; Lee, R. C.; Lesmana, L. A.; Lim, H. Y.; Paik, S. W.; Poon, R. T.; Tan, C. K.; Tanwandee, T.; Teng, G.; Cheng, A. L. | Consensus recommendations and review by an International Expert Panel on Interventions in Hepatocellular Carcinoma (EPOIHCC) | 2013 | Mar | Liver Int | 33 | 3 | 327-37 | 10.1111/liv.12083 |  |
| 666 | Park, J. W.; Koh, Y. H.; Kim, H. B.; Kim, H. Y.; An, S.; Choi, J. I.; Woo, S. M.; Nam, B. H. | Phase II study of concurrent transarterial chemoembolization and sorafenib in patients with unresectable hepatocellular carcinoma | 2012 | Jun | J Hepatol | 56 | 6 | 1336-42 | 10.1016/j.jhep.2012.01.006 | Wrong patient population |
| 667 | Park, J.; Kim, H. C.; Lee, J. H.; Cho, E.; Kim, M.; Hur, S.; Jae, H. J.; Lee, M.; Chung, J. W. | Chemoembolisation for hepatocellular carcinoma with bile duct invasion: is preprocedural biliary drainage mandatory? | 2018 | Apr | Eur Radiol | 28 | 4 | 1540-1550 | 10.1007/s00330-017-5110-7 |  |
| 668 | Park, W. D.; Li, B. T.; Maher, R.; Samra, J. S.; Clarke, S.; Bernard, E. J.; Bailey, D. L.; Pavlakis, N. | Dramatic response to selective internal radiation therapy for unresectable hepatocellular carcinoma | 2015 | Feb | Oxf Med Case Reports | 2015 | 2 | 194-5 | 10.1093/omcr/omv007 |  |
| 669 | Pascual, S.; Herrera, I.; Irurzun, J. | New advances in hepatocellular carcinoma | 2016 | 28-Mar | World J Hepatol | 8 | 9 | 421-38 | 10.4254/wjh.v8.i9.421 |  |
| 670 | Patel, A.; Subbanna, I.; Bhargavi, V.; Swamy, S.; Kallur, K. G.; Patil, S. | Transarterial Radioembolization (TARE) with (131) Iodine-Lipiodol for Unresectable Primary Hepatocellular Carcinoma: Experience from a Tertiary Care Center in India | 2021 | Apr | South Asian J Cancer | 10 | 2 | 81-86 | 10.1055/s-0041-1731600 |  |
| 671 | Patel, M. V.; Davies, H.; Williams, A. O.; Bromilow, T.; Baker, H.; Mealing, S.; Holmes, H.; Anderson, N.; Ahmed, O. | Transarterial therapies in patients with hepatocellular carcinoma eligible for transarterial embolization: a US cost-effectiveness analysis | 2023 | Jan-Dec | J Med Econ | 26 | 1 | 1061-1071 | 10.1080/13696998.2023.2248840 |  |
| 672 | Patidar, Y.; Mukund, A.; Sarin, S. K. | Transarterial Chemoembolization in Unresectable Hepatocellular Carcinoma with Portal Vein Tumor Thrombosis: A Tertiary Care Center Experience | 2021 | Apr | Indian J Radiol Imaging | 31 | 2 | 270-276 | 10.1055/s-0041-1734367 | Wrong patient population |
| 673 | Peck-Radosavljevic, Markus; Kudo, Masatoshi; Raoul, Jean-Luc; Lee, Han Chu; Decaens, Thomas; Heo, Jeong; Lin, Shi-Ming; Shan, Hong; Yang, Yefa; Bayh, Inga | Outcomes of patients (pts) with hepatocellular carcinoma (HCC) treated with transarterial chemoembolization (TACE): Global OPTIMIS final analysis | 2018 |  |  |  |  |  |  | Wrong patient population |
| 674 | Pei, Y.; Li, W.; Wang, Z.; Liu, J. | Successful conversion therapy for unresectable hepatocellular carcinoma is getting closer: A systematic review and meta-analysis | 2022 |  | Front Oncol | 12 |  | 978823 | 10.3389/fonc.2022.978823 |  |
| 675 | Pelizzaro, Filippo; Haxhi, Selion; Penzo, Barbara; Vitale, Alessandro; Giannini, Edoardo G; Sansone, Vito; Rapaccini, Gian Ludovico; Di Marco, Maria; Caturelli, Eugenio; Magalotti, Donatella | Transarterial chemoembolization for hepatocellular carcinoma in clinical practice: temporal trends and survival outcomes of an iterative treatment | 2022 |  | Frontiers in Oncology | 12 |  | 822507 |  |  |
| 676 | Peng, C. W.; Teng, W.; Lui, K. W.; Hung, C. F.; Jeng, W. J.; Huang, C. H.; Chen, W. T.; Lin, C. C.; Lin, C. Y.; Lin, S. M.; Sheen, I. S. | Complete response at first transarterial chemoembolization predicts favorable outcome in hepatocellular carcinoma | 2021 |  | Am J Cancer Res | 11 | 10 | 4956-4965 |  |  |
| 677 | Peng, N.; Mao, L.; Tao, Y.; Xiao, K.; Yuan, G.; He, S. | Callispheres® drug-eluting beads transarterial chemoembolization might be an efficient and safety down-staging therapy in unresectable liver cancer patients | 2022 | 9-Aug | World J Surg Oncol | 20 | 1 | 254 | 10.1186/s12957-022-02717-9 | Wrong outcomes |
| 678 | Peng, T. R.; Wu, T. W.; Wu, C. C.; Chang, S. Y.; Chan, C. Y.; Hsu, C. S. | Transarterial chemoembolization with or without sorafenib for hepatocellular carcinoma: A real-world propensity score-matched study | 2022 | Apr-Jun | Tzu Chi Med J | 34 | 2 | 219-225 | 10.4103/tcmj.tcmj_84_21 | Wrong outcomes |
| 679 | Pinato, D. J.; Karamanakos, G.; Arizumi, T.; Adjogatse, D.; Kim, Y. W.; Stebbing, J.; Kudo, M.; Jang, J. W.; Sharma, R. | Dynamic changes of the inflammation-based index predict mortality following chemoembolisation for hepatocellular carcinoma: a prospective study | 2014 | Dec | Aliment Pharmacol Ther | 40 | 12-Nov | 1270-81 | 10.1111/apt.12992 |  |
| 680 | Pinato, D. J.; Sharma, R. | An inflammation-based prognostic index predicts survival advantage after transarterial chemoembolization in hepatocellular carcinoma | 2012 | Aug | Transl Res | 160 | 2 | 146-52 | 10.1016/j.trsl.2012.01.011 |  |
| 681 | Pino, C.; Vecchio, G.; Fronda, M.; Calandri, M.; Aldinucci, M.; Spampinato, C. | TwinLiverNet: Predicting TACE Treatment Outcome from CT scans for Hepatocellular Carcinoma using Deep Capsule Networks | 2021 | Nov | Annu Int Conf IEEE Eng Med Biol Soc | 2021 |  | 3039-3043 | 10.1109/embc46164.2021.9630913 |  |
| 682 | Pistre, P.; Guiu, B.; Gehin, S.; Boulin, M. | Intra-arterial idarubicin_lipiodol without embolization can provide prolonged complete response in hepatocellular carcinoma: A case report | 2020 | Mar | J Oncol Pharm Pract | 26 | 2 | 507-510 | 10.1177/1078155219861422 |  |
| 683 | Pitton, M. B.; Kloeckner, R.; Ruckes, C.; Wirth, G. M.; Eichhorn, W.; Wörns, M. A.; Weinmann, A.; Schreckenberger, M.; Galle, P. R.; Otto, G.; Dueber, C. | Randomized comparison of selective internal radiotherapy (SIRT) versus drug-eluting bead transarterial chemoembolization (DEB-TACE) for the treatment of hepatocellular carcinoma | 2015 | Apr | Cardiovasc Intervent Radiol | 38 | 2 | 352-60 | 10.1007/s00270-014-1012-0 | Wrong patient population |
| 684 | Pollock, R. F.; Brennan, V. K.; Shergill, S.; Colaone, F. | A systematic literature review and network meta-analysis of first-line treatments for unresectable hepatocellular carcinoma based on data from randomized controlled trials | 2021 | Mar | Expert Rev Anticancer Ther | 21 | 3 | 341-349 | 10.1080/14737140.2021.1842204 | Wrong patient population |
| 685 | Pourbaghi, M.; Haghani, L.; Zhao, K.; Karimi, A.; Marinelli, B.; Erinjeri, J. P.; Geschwind, J. H.; Yarmohammadi, H. | Anti-Glycolytic Drugs in the Treatment of Hepatocellular Carcinoma: Systemic and Locoregional Options | 2023 | 10-Jul | Curr Oncol | 30 | 7 | 6609-6622 | 10.3390/curroncol30070485 |  |
| 686 | Prajapati, H. J.; Kim, H. S. | Treatment algorithm based on the multivariate survival analyses in patients with advanced hepatocellular carcinoma treated with trans-arterial chemoembolization | 2017 |  | PLoS One | 12 | 2 | e0170750 | 10.1371/journal.pone.0170750 |  |
| 687 | Prajapati, H. J.; Rafi, S.; El-Rayes, B. F.; Kauh, J. S.; Kooby, D. A.; Kim, H. S. | Safety and feasibility of same-day discharge of patients with unresectable hepatocellular carcinoma treated with doxorubicin drug-eluting bead transcatheter chemoembolization | 2012 | Oct | J Vasc Interv Radiol | 23 | 10 | 1286-93.e1 | 10.1016/j.jvir.2012.07.003 | Wrong comparator |
| 688 | Prajapati, H. J.; Spivey, J. R.; Hanish, S. I.; El-Rayes, B. F.; Kauh, J. S.; Chen, Z.; Kim, H. S. | mRECIST and EASL responses at early time point by contrast-enhanced dynamic MRI predict survival in patients with unresectable hepatocellular carcinoma (HCC) treated by doxorubicin drug-eluting beads transarterial chemoembolization (DEB TACE) | 2013 | Apr | Ann Oncol | 24 | 4 | 965-73 | 10.1093/annonc/mds605 | Wrong patient population |
| 689 | Prajapati, H. J.; Xing, M.; Spivey, J. R.; Hanish, S. I.; El-Rayes, B. F.; Kauh, J. S.; Chen, Z.; Kim, H. S. | Survival, efficacy, and safety of small versus large doxorubicin drug-eluting beads TACE chemoembolization in patients with unresectable HCC | 2014 | Dec | AJR Am J Roentgenol | 203 | 6 | W706-14 | 10.2214/ajr.13.12308 | Wrong patient population |
| 690 | Prasoppokakorn, T.; Thanapirom, K.; Treeprasertsuk, S. | Nephrotic Syndrome Induced by Lenvatinib Treatment for Hepatocellular Carcinoma | 2022 |  | Case Reports Hepatol | 2022 |  | 5101856 | 10.1155/2022/5101856 |  |
| 691 | Qian, K.; Zhang, F.; Allison, S. K.; Zheng, C.; Yang, X. | Image-guided locoregional non-intravascular interventional treatments for hepatocellular carcinoma: Current status | 2021 | Feb | J Interv Med | 4 | 1 | 7-Jan | 10.1016/j.jimed.2020.10.008 |  |
| 692 | Qian, X. H.; Yan, Y. C.; Gao, B. Q.; Wang, W. L. | Prevalence, diagnosis, and treatment of primary hepatic gastrointestinal stromal tumors | 2020 | 28-Oct | World J Gastroenterol | 26 | 40 | 6195-6206 | 10.3748/wjg.v26.i40.6195 |  |
| 693 | Qu, S.; Wu, D.; Hu, Z. | Neutrophil-to-Lymphocyte Ratio and Early Tumor Shrinkage as Predictive Biomarkers in Unresectable Hepatocellular Carcinoma Patients Treated With Lenvatinib, PD-1 Inhibitors, in Combination With TACE | 2023 | Jan-Dec | Technol Cancer Res Treat | 22 |  | 1.53E+16 | 10.1177/15330338231206704 |  |
| 694 | Qu, S.; Zhang, X.; Wu, Y.; Meng, Y.; Pan, H.; Fang, Q.; Hu, L.; Zhang, J.; Wang, R.; Wei, L.; Wu, D. | Efficacy and Safety of TACE Combined With Lenvatinib Plus PD-1 Inhibitors Compared With TACE Alone for Unresectable Hepatocellular Carcinoma Patients: A Prospective Cohort Study | 2022 |  | Front Oncol | 12 |  | 874473 | 10.3389/fonc.2022.874473 | Wrong patient population |
| 695 | Qu, W. F.; Ding, Z. B.; Qu, X. D.; Tang, Z.; Zhu, G. Q.; Fu, X. T.; Zhang, Z. H.; Zhang, X.; Huang, A.; Tang, M.; Tian, M. X.; Jiang, X. F.; Huang, R.; Tao, C. Y.; Fang, Y.; Gao, J.; Wu, X. L.; Zhou, J.; Fan, J.; Liu, W. R.; Shi, Y. H. | Conversion therapy for initially unresectable hepatocellular carcinoma using a combination of toripalimab, lenvatinib plus TACE: real-world study | 2022 | 2-Sep | BJS Open | 6 | 5 |  | 10.1093/bjsopen/zrac114 |  |
| 696 | Ranieri, G.; Ammendola, M.; Marech, I.; Laterza, A.; Abbate, I.; Oakley, C.; Vacca, A.; Sacco, R.; Gadaleta, C. D. | Vascular endothelial growth factor and tryptase changes after chemoembolization in hepatocarcinoma patients | 2015 | 21-May | World J Gastroenterol | 21 | 19 | 6018-25 | 10.3748/wjg.v21.i19.6018 |  |
| 697 | Raphael, M. J.; Karanicolas, P. J. | Regional Therapy for Colorectal Cancer Liver Metastases: Which Modality and When? | 2022 | 20-Aug | J Clin Oncol | 40 | 24 | 2806-2817 | 10.1200/jco.21.02505 |  |
| 698 | Ray, C. E., Jr.; Edwards, A.; Smith, M. T.; Leong, S.; Kondo, K.; Gipson, M.; Rochon, P. J.; Gupta, R.; Messersmith, W.; Purcell, T.; Durham, J. | Metaanalysis of survival, complications, and imaging response following chemotherapy-based transarterial therapy in patients with unresectable intrahepatic cholangiocarcinoma | 2013 | Aug | J Vasc Interv Radiol | 24 | 8 | 1218-26 | 10.1016/j.jvir.2013.03.019 |  |
| 699 | Razi, M.; Jianping, G.; Xu, H.; Ahmed, M. J. | Conventional versus drug-eluting bead transarterial chemoembolization: A better option for treatment of unresectable hepatocellular carcinoma | 2021 | Feb | J Interv Med | 4 | 1 | 14-Nov | 10.1016/j.jimed.2020.10.006 | Wrong patient population |
| 700 | Razi, Murtuza; Safiullah, Syed; Gu, Jianping; He, Xu; Razi, Mustafa; Kong, Jie | Comparison of tumor response following conventional versus drug-eluting bead transarterial chemoembolization in early-and very early-stage hepatocellular carcinoma | 2022 |  | Journal of Interventional Medicine | 5 | 1 | 14-Oct |  | Wrong publication type |
| 701 | Recchia, F.; Passalacqua, G.; Filauri, P.; Doddi, M.; Boscarato, P.; Candeloro, G.; Necozione, S.; Desideri, G.; Rea, S. | Chemoembolization of unresectable hepatocellular carcinoma: Decreased toxicity with slow-release doxorubicin"‘eluting beads compared with lipiodol | 2012 | May | Oncol Rep | 27 | 5 | 1377-83 | 10.3892/or.2012.1651 | Wrong patient population |
| 702 | Ren, B.; Wang, W.; Shen, J.; Li, W.; Ni, C.; Zhu, X. | Transarterial Chemoembolization (TACE) Combined with Sorafenib versus TACE Alone for Unresectable Hepatocellular Carcinoma: A Propensity Score Matching Study | 2019 |  | J Cancer | 10 | 5 | 1189-1196 | 10.7150/jca.28994 | Wrong patient population |
| 703 | Ren, Y.; Chen, L.; Huang, S.; Zhou, C.; Liu, J.; Shi, Q.; Yang, C.; Chen, R.; Zheng, C.; Han, P.; Xiong, B. | Transarterial chemoembolization of unresectable systemic chemotherapy refractory liver metastases: a retrospective single-center analysis | 2020 | Sep | Abdom Radiol (NY) | 45 | 9 | 2862-2870 | 10.1007/s00261-020-02584-6 |  |
| 704 | Ren, Y.; Guo, Y.; Chen, L.; Sun, T.; Zhang, W.; Sun, B.; Zhu, L.; Xiong, F.; Zheng, C. | Efficacy of Drug-Eluting Beads Transarterial Chemoembolization Plus Camrelizumab Compared With Conventional Transarterial Chemoembolization Plus Camrelizumab for Unresectable Hepatocellular Carcinoma | 2022 | Jan-Dec | Cancer Control | 29 |  | 1.07E+16 | 10.1177/10732748221076806 |  |
| 705 | Richter, G.; Radeleff, B.; Stroszczynski, C.; Pereira, P.; Helmberger, T.; Barakat, M.; Huppert, P. | Safety and Feasibility of Chemoembolization with Doxorubicin-Loaded Small Calibrated Microspheres in Patients with Hepatocellular Carcinoma: Results of the MIRACLE I Prospective Multicenter Study | 2018 | Apr | Cardiovasc Intervent Radiol | 41 | 4 | 587-593 | 10.1007/s00270-017-1839-2 | Wrong patient population |
| 706 | Rognoni, C.; Ciani, O.; Sommariva, S.; Facciorusso, A.; Tarricone, R.; Bhoori, S.; Mazzaferro, V. | Trans-arterial radioembolization in intermediate-advanced hepatocellular carcinoma: systematic review and meta-analyses | 2016 | 1-Nov | Oncotarget | 7 | 44 | 72343-72355 | 10.18632/oncotarget.11644 |  |
| 707 | Roth, Gael S; Hernandez, Olivier; Daabek, Najeh; Brusset, Bleuenn; Teyssier, Yann; Ghelfi, Julien; Hilleret, Marie Noelle; Sengel, Christian; Bricault, Ivan; Decaens, Thomas | Safety and Efficacy of Transarterial Chemoembolization in Elderly Patients with Intermediate Hepatocellular Carcinoma | 2022 |  | Cancers | 14 | 7 | 1634 |  | Wrong patient population |
| 708 | Rudnick, S. R.; Russo, M. W. | Liver transplantation beyond or downstaging within the Milan criteria for hepatocellular carcinoma | 2018 | Mar | Expert Rev Gastroenterol Hepatol | 12 | 3 | 265-275 | 10.1080/17474124.2018.1417035 |  |
| 709 | Sacco, Rodolfo; Tapete, Gherardo; Simonetti, Natalia; Sellitri, Rossella; Natali, Veronica; Melissari, Sara; Cabibbo, Giuseppe; Biscaglia, Lilia; Bresci, Giampaolo; Giacomelli, Luca | Transarterial chemoembolization for the treatment of hepatocellular carcinoma: a review | 2017 |  | Journal of hepatocellular carcinoma | 4 |  | 105 |  |  |
| 710 | Sato, N.; Beppu, T.; Kinoshita, K.; Yuki, H.; Suyama, K.; Chiyonaga, S.; Motohara, T.; Komohara, Y.; Hara, A.; Akahoshi, S. | Conversion Hepatectomy for Huge Hepatocellular Carcinoma With Arterioportal Shunt After Chemoembolization and Lenvatinib Therapy | 2019 | Oct | Anticancer Res | 39 | 10 | 5695-5701 | 10.21873/anticanres.13768 |  |
| 711 | Savic, Lynn Jeanette; Chen, Evan; Nezami, Nariman; Murali, Nikitha; Hamm, Charlie Alexander; Wang, Clinton; Lin, MingDe; Schlachter, Todd; Hong, Kelvin; Georgiades, Christos | Conventional vs. Drug-Eluting Beads Transarterial Chemoembolization for Unresectable Hepatocellular Carcinoma-A Propensity Score Weighted Comparison of Efficacy and Safety | 2022 |  | Cancers | 14 | 23 | 5847 |  | Wrong outcomes |
| 712 | Sayan, M.; Yegya-Raman, N.; Greco, S. H.; Gui, B.; Zhang, A.; Chundury, A.; Grandhi, M. S.; Hochster, H. S.; Kennedy, T. J.; Langan, R. C.; Malhotra, U.; Rustgi, V. K.; Shah, M. M.; Spencer, K. R.; Carpizo, D. R.; Nosher, J. L.; Jabbour, S. K. | Rethinking the Role of Radiation Therapy in the Treatment of Unresectable Hepatocellular Carcinoma: A Data Driven Treatment Algorithm for Optimizing Outcomes | 2019 |  | Front Oncol | 9 |  | 345 | 10.3389/fonc.2019.00345 |  |
| 713 | Sayana, H.; Yousef, O.; Clarkston, W. K. | Massive upper gastrointestinal hemorrhage due to invasive hepatocellular carcinoma and hepato-gastric fistula | 2013 | 14-Nov | World J Gastroenterol | 19 | 42 | 7472-5 | 10.3748/wjg.v19.i42.7472 |  |
| 714 | Scaffaro, L. A.; Kruel, C. D.; Stella, S. F.; Gravina, G. L.; Machado Filho, G.; Borges de Almeida, C. P.; Pinto, L. C.; Alvares-da-Silva, M. R.; Kruel, C. R. | Transarterial Embolization for Hepatocellular Carcinoma: A Comparison between Nonspherical PVA and Microspheres | 2015 |  | Biomed Res Int | 2015 |  | 435120 | 10.1155/2015/435120 | Wrong patient population |
| 715 | Schmid, I.; von Schweinitz, D. | Pediatric hepatocellular carcinoma: challenges and solutions | 2017 |  | J Hepatocell Carcinoma | 4 |  | 15-21 | 10.2147/jhc.S94008 |  |
| 716 | Schnapauff, D.; Tegel, B. R.; Powerski, M. J.; Colletini, F.; Hamm, B.; Gebauer, B. | Interstitial Brachytherapy in Combination With Previous Transarterial Embolization in Patients With Unresectable Hepatocellular Carcinoma | 2019 | Mar | Anticancer Res | 39 | 3 | 1329-1336 | 10.21873/anticanres.13245 |  |
| 717 | Scoggins, C. R. | TACE or TARE for Unresectable Neuroendocrine Liver Metastases: Can we Finally Start to Focus on Value? | 2021 | Apr | Ann Surg Oncol | 28 | 4 | 1876-1877 | 10.1245/s10434-021-09598-4 |  |
| 718 | Scott, A.; Wong, P.; Melstrom, L. G. | Surgery and hepatic artery infusion therapy for intrahepatic cholangiocarcinoma | 2023 | Jul | Surgery | 174 | 1 | 113-115 | 10.1016/j.surg.2023.01.019 |  |
| 719 | Sebastian, N. T.; Miller, E. D.; Yang, X.; Diaz, D. A.; Tan, Y.; Dowell, J.; Spain, J.; Rikabi, A.; Elliott, E.; Knopp, M.; Williams, T. M. | A Pilot Trial Evaluating Stereotactic Body Radiation Therapy to Induce Hyperemia in Combination With Transarterial Chemoembolization for Hepatocellular Carcinoma | 2020 | 1-Dec | Int J Radiat Oncol Biol Phys | 108 | 5 | 1276-1283 | 10.1016/j.ijrobp.2020.07.033 | Wrong intervention |
| 720 | Sefrioui, D.; Verdier, V.; Savoye-Collet, C.; Beaussire, L.; Ghomadi, S.; Gangloff, A.; Goria, O.; Riachi, G.; Montialoux, H.; Schwarz, L.; Tuech, J. J.; Frebourg, T.; Michel, P.; Sarafan-Vasseur, N.; Di Fiore, F. | Circulating DNA changes are predictive of disease progression after transarterial chemoembolization | 2022 | 1-Feb | Int J Cancer | 150 | 3 | 532-541 | 10.1002/ijc.33829 |  |
| 721 | Sefrioui, D.; Verdier, V.; Savoye-Collet, C.; Beaussire, L.; Ghomadi, S.; Gangloff, A.; Goria, O.; Riachi, G.; Montialoux, H.; Schwarz, L.; Tuech, J. J.; Frebourg, T.; Michel, P.; Sarafan-Vasseur, N.; Di Fiore, F. | Circulating DNA changes are predictive of disease progression after transarterial chemoembolization | 2022 | 1-Feb | Int J Cancer | 150 | 3 | 532-541 | 10.1002/ijc.33829 |  |
| 722 | Seinstra, B. A.; Defreyne, L.; Lambert, B.; Lam, M. G.; Verkooijen, H. M.; van Erpecum, K. J.; van Hoek, B.; van Erkel, A. R.; Coenraad, M. J.; Al Younis, I.; van Vlierberghe, H.; van den Bosch, M. A. | Transarterial radioembolization versus chemoembolization for the treatment of hepatocellular carcinoma (TRACE): study protocol for a randomized controlled trial | 2012 | 23-Aug | Trials | 13 |  | 144 | 10.1186/1745-6215-13-144 |  |
| 723 | Seki, A.; Hori, S. | Switching the loaded agent from epirubicin to cisplatin: salvage transcatheter arterial chemoembolization with drug-eluting microspheres for unresectable hepatocellular carcinoma | 2012 | Jun | Cardiovasc Intervent Radiol | 35 | 3 | 555-62 | 10.1007/s00270-011-0176-0 |  |
| 724 | Seko, Y.; Ikeda, K.; Kawamura, Y.; Fukushima, T.; Hara, T.; Sezaki, H.; Hosaka, T.; Akuta, N.; Suzuki, F.; Kobayashi, M.; Suzuki, Y.; Saitoh, S.; Arase, Y.; Kumada, H. | Antitumor efficacy of transcatheter arterial chemoembolization with warmed miriplatin in hepatocellular carcinoma | 2013 | Sep | Hepatol Res | 43 | 9 | 942-9 | 10.1111/hepr.12041 | Wrong patient population |
| 725 | Shaghaghi, M.; Aliyari, G. Hasabeh M.; Ameli, S.; Ghadimi, M.; Hazhirkarzar, B.; Rezvani Habibabadi, R.; Tang, H.; Khoshpouri, P.; Wu, Q.; Pandey, A.; Pandey, P.; Baghdadi, A.; Kamel, I. R. | Role of tumor margin and ADC change in defining the need for additional treatments after the first TACE in patients with unresectable HCC | 2020 | Dec | Eur J Radiol | 133 |  | 109389 | 10.1016/j.ejrad.2020.109389 |  |
| 726 | Shao, W.; Li, C.; Tang, J.; Song, J.; Li, Z.; Sun, J.; Xu, Y.; Zheng, Z.; Cao, J.; Zhang, L. | Efficacy And Safety Of Raltitrexed Plus Oxaliplatin-Based Transarterial Chemoembolization In Patients With Unresectable Hepatocellular Carcinoma | 2019 |  | Cancer Manag Res | 11 |  | 9863-9869 | 10.2147/cmar.S217524 | Wrong patient population |
| 727 | She, W. H.; Cheung, T. T.; Yau, T. C.; Chan, A. C.; Chok, K. S.; Chu, F. S.; Liu, R. K.; Poon, R. T.; Chan, S. C.; Fan, S. T.; Lo, C. M. | Survival analysis of transarterial radioembolization with yttrium-90 for hepatocellular carcinoma patients with HBV infection | 2014 | Aug | Hepatobiliary Surg Nutr | 3 | 4 | 185-93 | 10.3978/j.issn.2304-3881.2014.07.09 |  |
| 728 | Shen, A.; Liu, S.; Yu, W.; Deng, H.; Li, Q. | p53 gene therapy-based transarterial chemoembolization for unresectable hepatocellular carcinoma: A prospective cohort study | 2015 | Nov | J Gastroenterol Hepatol | 30 | 11 | 1651-6 | 10.1111/jgh.13009 | Wrong patient population |
| 729 | Shen, H.; Zheng, S.; Chen, R.; Jin, X.; Xu, X.; Jing, C.; Lin, J.; Zhang, J.; Zhang, M.; Zhang, L.; Xie, X.; Guo, K.; Ren, Z.; Lin, S.; Zhang, B. | Prognostic significance of serum procalcitonin in patients with unresectable hepatocellular carcinoma treated with transcatheter arterial chemoembolization: A retrospective analysis of 509 cases | 2017 | Jul | Medicine (Baltimore) | 96 | 28 | e7438 | 10.1097/md.0000000000007438 |  |
| 730 | Shen, J.; Wang, W. S.; Zhu, X. L.; Ni, C. F. | High Epithelial Cell Adhesion Molecule-Positive Circulating Tumor Cell Count Predicts Poor Survival of Patients with Unresectable Hepatocellular Carcinoma Treated with Transcatheter Arterial Chemoembolization | 2018 | Dec | J Vasc Interv Radiol | 29 | 12 | 1678-1684 | 10.1016/j.jvir.2018.07.030 |  |
| 731 | Shen, J.; Wang, X.; Wang, N.; Wen, S.; Yang, G.; Li, L.; Fu, J.; Pan, X. | HBV reactivation and its effect on survival in HBV-related hepatocarcinoma patients undergoing transarterial chemoembolization combined with tyrosine kinase inhibitors plus immune checkpoint inhibitors | 2023 |  | Front Cell Infect Microbiol | 13 |  | 1179689 | 10.3389/fcimb.2023.1179689 |  |
| 732 | Shen, P. C.; Chang, W. C.; Lo, C. H.; Yang, J. F.; Lee, M. S.; Dai, Y. H.; Lin, C. S.; Fan, C. Y.; Huang, W. Y. | Comparison of Stereotactic Body Radiation Therapy and Transarterial Chemoembolization for Unresectable Medium-Sized Hepatocellular Carcinoma | 2019 | 1-Oct | Int J Radiat Oncol Biol Phys | 105 | 2 | 307-318 | 10.1016/j.ijrobp.2019.05.066 |  |
| 733 | Shen, T.; Jia, Z.; Huang, Y.; Li, S.; Jiang, G.; Cheng, L. | Chemoembolization for hepatocellular carcinoma fed by right internal thoracic artery | 2017 | Nov | Medicine (Baltimore) | 96 | 45 | e8634 | 10.1097/md.0000000000008634 |  |
| 734 | Shi, M.; Lu, L. G.; Fang, W. Q.; Guo, R. P.; Chen, M. S.; Li, Y.; Luo, J.; Xu, L.; Zou, R. H.; Lin, X. J.; Zhang, Y. Q. | Roles played by chemolipiodolization and embolization in chemoembolization for hepatocellular carcinoma: single-blind, randomized trial | 2013 | 2-Jan | J Natl Cancer Inst | 105 | 1 | 59-68 | 10.1093/jnci/djs464 |  |
| 735 | Shi, Q.; Liu, J.; Li, T.; Zhou, C.; Wang, Y.; Huang, S.; Yang, C.; Chen, Y.; Xiong, B. | Comparison of DEB-TACE and cTACE for the initial treatment of unresectable hepatocellular carcinoma beyond up-to-seven criteria: A single-center propensity score matching analysis | 2022 | May | Clin Res Hepatol Gastroenterol | 46 | 5 | 101893 | 10.1016/j.clinre.2022.101893 | Wrong patient population |
| 736 | Shi, Q.; Zhang, X.; Wu, M.; Xia, Y.; Pan, Y.; Weng, J.; Li, N.; Zan, X.; Xia, J. | Emulsifying Lipiodol with pH-sensitive DOX@HmA nanoparticles for hepatocellular carcinoma TACE treatment eliminate metastasis | 2023 | Dec | Mater Today Bio | 23 |  | 100873 | 10.1016/j.mtbio.2023.100873 |  |
| 737 | Shi, X. J.; Jin, X.; Wang, M. Q.; Wei, L. X.; Ye, H. Y.; Liang, Y. R.; Luo, Y.; Dong, J. H. | Effect of resection following downstaging of unresectable hepatocelluar carcinoma by transcatheter arterial chemoembolization | 2012 | Jan | Chin Med J (Engl) | 125 | 2 | 197-202 |  |  |
| 738 | Shi, X.; Wang, Y.; Ren, J.; Han, X.; Bi, Y. | A retrospective pilot study of transarterial chemoembolisation using camrelizumab-eluting Callisphere beads for unresectable hepatocellular carcinoma | 2023 | 24-Nov | BMC Cancer | 23 | 1 | 1144 | 10.1186/s12885-023-11668-7 |  |
| 739 | Shi, Z.; Wang, D.; Kang, T.; Yi, R.; Cui, L.; Jiang, H. | Comparison of CalliSpheres(®) microspheres drug-eluting beads and conventional transarterial chemoembolization in hepatocellular carcinoma patients: a randomized controlled trial | 2023 | 1-Mar | Radiol Oncol | 57 | 1 | 70-79 | 10.2478/raon-2023-0001 | Wrong patient population |
| 740 | Shimose, S.; Iwamoto, H.; Shirono, T.; Tanaka, M.; Niizeki, T.; Kajiwara, M.; Itano, S.; Yano, Y.; Matsugaki, S.; Moriyama, E.; Noda, Y.; Nakano, M.; Kuromatsu, R.; Koga, H.; Kawaguchi, T. | The impact of curative conversion therapy aimed at a cancer-free state in patients with hepatocellular carcinoma treated with atezolizumab plus bevacizumab | 2023 | Jun | Cancer Med | 12 | 11 | 12325-12335 | 10.1002/cam4.5931 |  |
| 741 | Shimose, Shigeo; Tanaka, Masatoshi; Iwamoto, Hideki; Niizeki, Takashi; Shirono, Tomotake; Aino, Hajime; Noda, Yu; Kamachi, Naoki; Okamura, Shusuke; Nakano, Masahito | Prognostic impact of transcatheter arterial chemoembolization (TACE) combined with radiofrequency ablation in patients with unresectable hepatocellular carcinoma: Comparison with TACE alone using decision-tree analysis after propensity score matching | 2019 |  | Hepatology Research | 49 | 8 | 919-928 |  | Wrong patient population |
| 742 | Shindoh, J.; Kawamura, Y.; Kobayashi, Y.; Akuta, N.; Kobayashi, M.; Suzuki, Y.; Ikeda, K.; Hashimoto, M. | Time-to-Interventional Failure as a New Surrogate Measure for Survival Outcomes after Resection of Hepatocellular Carcinoma | 2020 | Jan | J Gastrointest Surg | 24 | 1 | 50-57 | 10.1007/s11605-019-04277-y |  |
| 743 | Shirono, T.; Koga, H.; Niizeki, T.; Nagamatsu, H.; Iwamoto, H.; Shimose, S.; Nakano, M.; Okamura, S.; Noda, Y.; Kamachi, N.; Kuromatsu, R.; Ogo, E.; Torimura, T. | Usefulness of a novel transarterial chemoinfusion plus external-beam radiation therapy for advanced hepatocellular carcinoma with tumor thrombi in the inferior vena cava and right atrium: Case study | 2021 | 24-Aug | Cancer Rep (Hoboken) | |  | e1539 | 10.1002/cnr2.1539 |  |
| 744 | Shirono, T.; Koga, H.; Niizeki, T.; Nagamatsu, H.; Iwamoto, H.; Shimose, S.; Nakano, M.; Okamura, S.; Noda, Y.; Kamachi, N.; Kuromatsu, R.; Ogo, E.; Torimura, T. | Usefulness of a novel transarterial chemoinfusion plus external-beam radiation therapy for advanced hepatocellular carcinoma with tumor thrombi in the inferior vena cava and right atrium: Case study | 2022 | Aug | Cancer Rep (Hoboken) | 5 | 8 | e1539 | 10.1002/cnr2.1539 |  |
| 745 | Sho, T.; Morikawa, K.; Kubo, A.; Tokuchi, Y.; Kitagataya, T.; Yamada, R.; Shigesawa, T.; Kimura, M.; Nakai, M.; Suda, G.; Natsuizaka, M.; Ogawa, K.; Sakamoto, N. | Prospect of lenvatinib for unresectable hepatocellular carcinoma in the new era of systemic chemotherapy | 2021 | 15-Dec | World J Gastrointest Oncol | 13 | 12 | 2076-2087 | 10.4251/wjgo.v13.i12.2076 |  |
| 746 | Shui, Y.; Yu, W.; Ren, X.; Guo, Y.; Xu, J.; Ma, T.; Zhang, B.; Wu, J.; Li, Q.; Hu, Q.; Shen, L.; Bai, X.; Liang, T.; Wei, Q. | Stereotactic body radiotherapy based treatment for hepatocellular carcinoma with extensive portal vein tumor thrombosis | 2018 | 25-Sep | Radiat Oncol | 13 | 1 | 188 | 10.1186/s13014-018-1136-5 |  |
| 747 | Si, T.; Huang, Z.; Khorsandi, S. E.; Ma, Y.; Heaton, N. | Hepatic arterial infusion chemotherapy versus transarterial chemoembolization for unresectable hepatocellular carcinoma: A systematic review with meta-analysis | 2022 |  | Front Bioeng Biotechnol | 10 |  | 1010824 | 10.3389/fbioe.2022.1010824 | Wrong study design |
| 748 | Si, Y.; Hu, X.; Du, H.; Lou, W.; Zhang, H.; Cao, F.; Yu, W.; Wang, W.; Jin, K. | Transarterial chemoembolization for patients with unresectable hepatocellular carcinoma: a retrospective study of a 5-year experience in a single institution | 2013 | Sep | Hepatogastroenterology | 60 | 126 | 1405-8 | 10.5754/hge121310 |  |
| 749 | Siraj, T. H.; Tameez Ud Din, A.; Chaudhary, F. M. D.; Ahmad, S.; Siddiqui, K. H. | Rapid Intrahepatic Progression of Hepatocellular Carcinoma after Transarterial Chemoembolization: A Case Report | 2019 | 2-Aug | Cureus | 11 | 8 | e5305 | 10.7759/cureus.5305 |  |
| 750 | Siriapisith, T.; Siwasattayanon, P.; Tongdee, T. | Radiofrequency ablation alone versus radiofrequency ablation combined with chemoembolization in unresectable hepatocellular carcinoma | 2012 | Mar | J Med Assoc Thai | 95 | 3 | 430-6 |  |  |
| 751 | Siriwardana, R. C.; Lo, C. M.; Chan, S. C.; Fan, S. T. | Role of portal vein embolization in hepatocellular carcinoma management and its effect on recurrence: a case-control study | 2012 | Jul | World J Surg | 36 | 7 | 1640-6 | 10.1007/s00268-012-1522-3 |  |
| 752 | Siriwardana, R. C.; Niriella, M. A.; Dassanayake, A. S.; Liyanage, C. A.; Upasena, A.; Sirigampala, C.; de Silva, H. J. | Factors affecting post-embolization fever and liver failure after trans-arterial chemo-embolization in a cohort without background infective hepatitis- a prospective analysis | 2015 | 4-Aug | BMC Gastroenterol | 15 |  | 96 | 10.1186/s12876-015-0329-8 |  |
| 753 | Somma, F.; Stoia, V.; Serra, N.; D'Angelo, R.; Gatta, G.; Fiore, F. | Yttrium-90 trans-arterial radioembolization in advanced-stage HCC: The impact of portal vein thrombosis on survival | 2019 |  | PLoS One | 14 | 5 | e0216935 | 10.1371/journal.pone.0216935 |  |
| 754 | Song, D. S.; Nam, S. W.; Bae, S. H.; Kim, J. D.; Jang, J. W.; Song, M. J.; Lee, S. W.; Kim, H. Y.; Lee, Y. J.; Chun, H. J.; You, Y. K.; Choi, J. Y.; Yoon, S. K. | Outcome of transarterial chemoembolization-based multi-modal treatment in patients with unresectable hepatocellular carcinoma | 2015 | 28-Feb | World J Gastroenterol | 21 | 8 | 2395-404 | 10.3748/wjg.v21.i8.2395 | Wrong patient population |
| 755 | Song, P.; Hai, Y.; Ma, W.; Zhao, L.; Wang, X.; Xie, Q.; Li, Y.; Wu, Z.; Li, Y.; Li, H. | Arsenic trioxide combined with transarterial chemoembolization for unresectable primary hepatic carcinoma: A systematic review and meta-analysis | 2018 | May | Medicine (Baltimore) | 97 | 18 | e0613 | 10.1097/md.0000000000010613 | Wrong patient population |
| 756 | Song, W.; Chen, Q.; Guo, D.; Jiang, C. | Preoperative estimation of the survival of patients with unresectable hepatocellular carcinoma achieving complete response after conventional transcatheter arterial chemoembolization: assessments of clinical and LI-RADS MR features | 2022 | Sep | Radiol Med | 127 | 9 | 939-949 | 10.1007/s11547-022-01517-1 |  |
| 757 | Song, Y.; Xing, H.; Zhou, L.; Zhang, N.; Yang, M. | LncRNA H19 modulated by miR-146b-3p/miR-1539-mediated allelic regulation in transarterial chemoembolization of hepatocellular carcinoma | 2021 | Sep | Arch Toxicol | 95 | 9 | 3063-3070 | 10.1007/s00204-021-03119-8 |  |
| 758 | Sottani, C.; Poggi, G.; Melchiorre, F.; Montagna, B.; Minoia, C. | Simultaneous measurement of doxorubicin and reduced metabolite doxorubicinol by UHPLC-MS/MS in human plasma of HCC patients treated with TACE | 2013 | 1-Feb | J Chromatogr B Analyt Technol Biomed Life Sci | 915-916 |  | 71-8 | 10.1016/j.jchromb.2012.12.012 |  |
| 759 | Stroehl, Y. W.; Letzen, B. S.; van Breugel, J. M.; Geschwind, J. F.; Chapiro, J. | Intra-arterial therapies for liver cancer: assessing tumor response | 2017 | Feb | Expert Rev Anticancer Ther | 17 | 2 | 119-127 | 10.1080/14737140.2017.1273775 |  |
| 760 | Su, M.; Chen, S.; Li, S.; Xu, F.; Zhao, G.; Qu, J.; Zhou, J. | Gelatin sponge microparticles for transarterial chemoembolization combined with regorafenib in hepatocellular carcinoma: a single-center retrospective study | 2022 | Dec | J Gastrointest Oncol | 13 | 6 | 3183-3192 | 10.21037/jgo-22-1170 |  |
| 761 | Su, T. S.; Lu, H. Z.; Cheng, T.; Zhou, Y.; Huang, Y.; Gao, Y. C.; Tang, M. Y.; Jiang, H. Y.; Lian, Z. P.; Hou, E. C.; Liang, P. | Long-term survival analysis in combined transarterial embolization and stereotactic body radiation therapy versus stereotactic body radiation monotherapy for unresectable hepatocellular carcinoma >5 cm | 2016 | 3-Nov | BMC Cancer | 16 | 1 | 834 | 10.1186/s12885-016-2894-9 | Wrong patient population |
| 762 | Suh, Y. G.; Kim, D. Y.; Han, K. H.; Seong, J. | Effective biliary drainage and proper treatment improve outcomes of hepatocellular carcinoma with obstructive jaundice | 2014 | Sep | Gut Liver | 8 | 5 | 526-35 | 10.5009/gnl13370 |  |
| 763 | Suk Oh, J.; Jong Chun, H.; Gil Choi, B.; Giu Lee, H. | Transarterial chemoembolization with drug-eluting beads in hepatocellular carcinoma: usefulness of contrast saturation features on cone-beam computed tomography imaging for predicting short-term tumor response | 2013 | Apr | J Vasc Interv Radiol | 24 | 4 | 483-9 | 10.1016/j.jvir.2013.01.001 | Wrong patient population |
| 764 | Sun, B.; Zhang, L.; Sun, T.; Ren, Y.; Cao, Y.; Zhang, W.; Zhu, L.; Guo, Y.; Gui, Y.; Liu, F.; Chen, L.; Xiong, F.; Zheng, C. | Safety and efficacy of lenvatinib combined with camrelizumab plus transcatheter arterial chemoembolization for unresectable hepatocellular carcinoma: A two-center retrospective study | 2022 |  | Front Oncol | 12 |  | 982948 | 10.3389/fonc.2022.982948 |  |
| 765 | Sun, B.; Zhang, L.; Xiang, D.; Li, Q.; Ren, Y.; Cao, Y.; Sun, T.; Zhang, W.; Wu, L.; Zhu, L.; Chen, L.; Zhao, H.; Zheng, C. | The Effect of Alcohol Consumption in Unresectable Hepatocellular Carcinoma with Transarterial Chemoembolization | 2022 |  | J Oncol | 2022 |  | 7062105 | 10.1155/2022/7062105 |  |
| 766 | Sun, H. C.; Zhu, X. D. | Downstaging Conversion Therapy in Patients With Initially Unresectable Advanced Hepatocellular Carcinoma: An Overview | 2021 |  | Front Oncol | 11 |  | 772195 | 10.3389/fonc.2021.772195 |  |
| 767 | Sun, H.; Zhang, M.; Liu, R.; Liu, Y.; Hou, Y.; Wu, C. | Endovascular implantation of (125)I seed combined with transcatheter arterial chemoembolization for unresectable hepatocellular carcinoma | 2018 | May | Future Oncol | 14 | 12 | 1165-1176 | 10.2217/fon-2017-0354 |  |
| 768 | Sun, J. H.; Zhou, G. H.; Zhang, Y. L.; Nie, C. H.; Zhou, T. Y.; Ai, J.; Zhu, T. Y.; Wang, W. L.; Zheng, S. S. | Chemoembolization of liver cancer with drug-loading microsphere 50-100µm | 2017 | 17-Jan | Oncotarget | 8 | 3 | 5392-5399 | 10.18632/oncotarget.14281 | Wrong patient population |
| 769 | Sun, J.; Shi, J.; Huang, B.; Cheng, F.; Guo, W.; Lau, W. Y.; Cheng, S. | The degree of hepatic arterial blood supply of portal vein tumor thrombus in patients with hepatocellular carcinoma and its impact on overall survival after transarterial chemoembolization | 2017 | 3-Oct | Oncotarget | 8 | 45 | 79816-79824 | 10.18632/oncotarget.19767 |  |
| 770 | Sun, L.; Xu, X.; Meng, F.; Liu, Q.; Wang, H.; Li, X.; Li, G.; Chen, F. | Lenvatinib plus transarterial chemoembolization with or without immune checkpoint inhibitors for unresectable hepatocellular carcinoma: A review | 2022 |  | Front Oncol | 12 |  | 980214 | 10.3389/fonc.2022.980214 |  |
| 771 | Sun, Q.; Ma, W.; Gao, Y.; Zheng, W.; Zhang, B.; Peng, Y. | Meta-analysis: therapeutic effect of transcatheter arterial chemoembolization combined with compound kushen injection in hepatocellular carcinoma | 2012 |  | Afr J Tradit Complement Altern Med | 9 | 2 | 178-88 | 10.4314/ajtcam.v9i2.1 | Wrong patient population |
| 772 | Sun, T.; Zhang, W.; Chen, L.; Ren, Y.; Liu, Y.; Zheng, C. | A comparative study of efficacy and safety of transarterial chemoembolization with CalliSpheres and conventional transarterial chemoembolization in treating unresectable intrahepatic cholangiocarcinoma patients | 2022 |  | J Cancer | 13 | 4 | 1282-1288 | 10.7150/jca.67523 |  |
| 773 | Sun, T.; Zhang, W.; Chen, L.; Ren, Y.; Liu, Y.; Zheng, C. | A comparative study of efficacy and safety of transarterial chemoembolization with CalliSpheres and conventional transarterial chemoembolization in treating unresectable intrahepatic cholangiocarcinoma patients | 2022 |  | J Cancer | 13 | 4 | 1282-1288 | 10.7150/jca.67523 |  |
| 774 | Sun, Y.; Bai, H.; Xia, W.; Wang, D.; Zhou, B.; Zhao, X.; Yang, G.; Xu, L.; Zhang, W.; Liu, P.; Xu, J.; Meng, S.; Liu, R.; Gao, X. | Predicting the Outcome of Transcatheter Arterial Embolization Therapy for Unresectable Hepatocellular Carcinoma Based on Radiomics of Preoperative Multiparameter MRI | 2020 | Oct | J Magn Reson Imaging | 52 | 4 | 1083-1090 | 10.1002/jmri.27143 |  |
| 775 | Tachiiri, T.; Nishiofuku, H.; Maeda, S.; Sato, T.; Toyoda, S.; Matsumoto, T.; Chanoki, Y.; Minamiguchi, K.; Taiji, R.; Kunichika, H.; Yamauchi, S.; Ito, T.; Marugami, N.; Tanaka, T. | Vascular Normalization Caused by Short-Term Lenvatinib Could Enhance Transarterial Chemoembolization in Hepatocellular Carcinoma | 2023 | 5-May | Curr Oncol | 30 | 5 | 4779-4786 | 10.3390/curroncol30050360 |  |
| 776 | Takano, M.; Kokudo, T.; Miyazaki, Y.; Kageyama, Y.; Takahashi, A.; Amikura, K.; Sakamoto, H. | Complete response with sorafenib and transcatheter arterial chemoembolization in unresectable hepatocellular carcinoma | 2016 | 14-Nov | World J Gastroenterol | 22 | 42 | 9445-9450 | 10.3748/wjg.v22.i42.9445 |  |
| 777 | Takayasu, K. | Transcatheter arterial chemoembolization for unresectable hepatocellular carcinoma: recent progression and perspective | 2013 |  | Oncology | 84 Suppl 1 |  | 28-33 | 10.1159/000345886 | Wrong patient population |
| 778 | Takayasu, K. | Transarterial chemoembolization for hepatocellular carcinoma over three decades: current progress and perspective | 2012 | Apr | Jpn J Clin Oncol | 42 | 4 | 247-55 | 10.1093/jjco/hys020 |  |
| 779 | Takeda, K.; Tsurumaru, Y.; Yamamoto, Y.; Araki, K.; Kogure, Y.; Mori, K.; Nakagawa, K.; Shimizu, T.; Matsuda, G.; Niino, H.; Sekido, H.; Kobayashi, S.; Morimoto, M.; Kunisaki, C.; Endo, I. | Treatment of hepatocellular carcinoma with hepatic vein tumor thrombosis protruding into the inferior vena cava by conversion surgery following chemotherapy with regorafenib: a case report | 2020 | Jun | Clin J Gastroenterol | 13 | 3 | 428-433 | 10.1007/s12328-019-01077-4 |  |
| 780 | Taketomi, A. | Clinical trials of antiangiogenic therapy for hepatocellular carcinoma | 2016 | Apr | International Journal of Clinical Oncology | 21 | 2 | 213-218 | 10.1007/s10147-016-0966-0 |  |
| 781 | Tamai, T.; Kumagai, K.; Sakae, H.; Onishi, H.; Tabu, K.; Tabu, E.; Muromachi, K.; Saishoji, A.; Oda, K.; Mawatari, S.; Moriuchi, A.; Sakurai, K.; Ido, A. | Early sorafenib induction after transarterial chemoembolization for unresectable hepatocellular carcinoma: Can sorafenib after TACE improve loco-regional control? | 2017 | Dec | Mol Clin Oncol | 7 | 6 | 1135-1141 | 10.3892/mco.2017.1434 | Wrong patient population |
| 782 | Tan, J.; Tang, T.; Zhao, W.; Zhang, Z. S.; Xiao, Y. D. | Initial Incomplete Thermal Ablation Is Associated With a High Risk of Tumor Progression in Patients With Hepatocellular Carcinoma | 2021 |  | Front Oncol | 11 |  | 760173 | 10.3389/fonc.2021.760173 | Wrong patient population |
| 783 | Tanaka, H.; Okamoto, K.; Sato, Y.; Tanaka, T.; Tomonari, T.; Nakamura, F.; Fujino, Y.; Mitsui, Y.; Miyamoto, H.; Muguruma, N.; Morita, A.; Ikushima, H.; Takayama, T. | Synergistic anti-tumor activity of miriplatin and radiation through PUMA-mediated apoptosis in hepatocellular carcinoma | 2020 | Nov | J Gastroenterol | 55 | 11 | 1072-1086 | 10.1007/s00535-020-01705-8 |  |
| 784 | Tang, C. W.; Zhu, M.; Feng, W. M.; Bao, Y.; Zheng, Y. Y. | Chinese herbal medicine, Jianpi Ligan decoction, improves prognosis of unresectable hepatocellular carcinoma after transarterial chemoembolization: a retrospective study | 2016 |  | Drug Des Devel Ther | 10 |  | 2461-6 | 10.2147/dddt.S113295 |  |
| 785 | Tang, C.; Shen, J.; Feng, W.; Bao, Y.; Dong, X.; Dai, Y.; Zheng, Y.; Zhang, J. | Combination Therapy of Radiofrequency Ablation and Transarterial Chemoembolization for Unresectable Hepatocellular Carcinoma: A Retrospective Study | 2016 | May | Medicine (Baltimore) | 95 | 20 | e3754 | 10.1097/md.0000000000003754 | Wrong patient population |
| 786 | Tang, X.; He, X.; Jiang, H. | Efficacy and safety of HIFU in combination with TACE in unresectable pediatric HB: A randomized, controlled, single-center clinical trial | 2022 | 2-Dec | Medicine (Baltimore) | 101 | 48 | e32022 | 10.1097/md.0000000000032022 |  |
| 787 | Tanguturi, S. K.; Wo, J. Y.; Zhu, A. X.; Dawson, L. A.; Hong, T. S. | Radiation therapy for liver tumors: ready for inclusion in guidelines? | 2014 | Aug | Oncologist | 19 | 8 | 868-79 | 10.1634/theoncologist.2014-0097 |  |
| 788 | Tao, R.; Li, X.; Ran, R.; Xiao, Z.; Zhang, H.; Kong, H.; Song, Q.; Huang, Y.; Wang, L.; Huang, J. | A mixed analysis comparing nine minimally invasive surgeries for unresectable hepatocellular carcinoma patients | 2017 | 17-Jan | Oncotarget | 8 | 3 | 5460-5473 | 10.18632/oncotarget.12348 | Wrong patient population |
| 789 | Tao, Z.; Ruan, Y.; Peng, Z.; Zhang, K.; Gao, Y. | Transarterial Chemoembolization Combined With Endoscopic Therapy Is Beneficial for Unresectable Hepatocellular Carcinoma With Esophagogastric Varices | 2021 |  | Front Oncol | 11 |  | 783574 | 10.3389/fonc.2021.783574 | Wrong patient population |
| 790 | Tashrifwala, F. A. A.; Karmani, V. K.; Haider, I.; Syeda, A. Z.; Noorani, A.; Mustafa, M. S.; Dave, T.; Hafeez, H. | Efficacy of Transarterial Chemoembolization Combined With Camrelizumab in the Treatment of Hepatocellular Carcinoma: A Systematic Review and Meta-Analysis | 2023 | Nov | Cureus | 15 | 11 | e48673 | 10.7759/cureus.48673 |  |
| 791 | Tavernier, J.; Fagnoni, P.; Chabrot, P.; Guiu, B.; Vadot, L.; Aho, S.; Boyer, L.; Abergel, A.; Hillon, P.; Sautou, V.; Boulin, M. | Comparison of two transarterial chemoembolization strategies for hepatocellular carcinoma | 2014 | Dec | Anticancer Res | 34 | 12 | 7247-53 |  |  |
| 792 | Tawada, A.; Chiba, T.; Ooka, Y.; Kanogawa, N.; Saito, T.; Motoyama, T.; Ogasawara, S.; Suzuki, E.; Kanai, F.; Yoshikawa, M.; Yokosuka, O. | Transarterial chemoembolization with miriplatin plus epirubicin in patients with hepatocellular carcinoma | 2015 | Jan | Anticancer Res | 35 | 1 | 549-54 |  | Wrong patient population |
| 793 | Teng, Y.; Ding, X.; Li, W.; Sun, W.; Chen, J. | A Retrospective Study on Therapeutic Efficacy of Transarterial Chemoembolization Combined With Immune Checkpoint Inhibitors Plus Lenvatinib in Patients With Unresectable Hepatocellular Carcinoma | 2022 | Jan-Dec | Technol Cancer Res Treat | 21 |  | 1.53E+16 | 10.1177/15330338221075174 | Wrong patient population |
| 794 | Teng, Y.; Ding, X.; Li, W.; Sun, W.; Chen, J. | A Retrospective Study on Therapeutic Efficacy of Transarterial Chemoembolization Combined With Immune Checkpoint Inhibitors Plus Lenvatinib in Patients With Unresectable Hepatocellular Carcinoma | 2022 | Jan-Dec | Technol Cancer Res Treat | 21 |  | 1.53E+16 | 10.1177/15330338221075174 |  |
| 795 | Terzi, Eleonora; Piscaglia, Fabio; Forlani, Ludovica; Mosconi, Cristina; Renzulli, Matteo; Bolondi, Luigi; Golfieri, Rita | TACE performed in patients with a single nodule of hepatocellular carcinoma | 2014 |  | BMC Cancer | 14 | 1 | 14-Jan |  | Wrong patient population |
| 796 | Thorat, A.; Lee, C. F.; Wu, T. H.; Chan, K. M.; Chou, H. S.; Lee, W. C. | Safety of transarterial chemoembolization as bridging therapy in HCC patients with hyperbilirubinemia on the waiting list for liver transplantation: a centre experience | 2013 | Nov-Dec | Hepatogastroenterology | 60 | 128 | 2076-9 |  |  |
| 797 | Thuluvath, P. J.; To, C.; Amjad, W. | Role of Locoregional Therapies in Patients With Hepatocellular Cancer Awaiting Liver Transplantation | 2021 | 1-Jan | Am J Gastroenterol | 116 | 1 | 57-67 | 10.14309/ajg.0000000000000999 |  |
| 798 | Tian, M.; Zhang, X.; Huang, G.; Fan, W.; Li, J.; Zhang, Y. | Alpha-fetoprotein assessment for hepatocellular carcinoma after transarterial chemoembolization | 2019 | Oct | Abdom Radiol (NY) | 44 | 10 | 3304-3311 | 10.1007/s00261-019-02116-x |  |
| 799 | Tian, Z.; Hou, X.; Liu, W.; Shao, C.; Gao, L.; Jiang, J.; Zhang, L.; Han, Z.; Wei, L. | Targeted blocking of CCR2 and CXCR2 improves the efficacy of transarterial chemoembolization of hepatocarcinoma | 2022 | 19-Nov | Cancer Cell Int | 22 | 1 | 362 | 10.1186/s12935-022-02771-z |  |
| 800 | Tomonari, T.; Tanaka, H.; Tanaka, T.; Taniguchi, T.; Sogabe, M.; Kawano, Y.; Okamoto, K.; Miyamoto, H.; Sato, Y.; Takayama, T. | A case of complete response with rechallenge-lenvatinib plus transcatheter arterial chemoembolization for unresectable hepatocellular carcinoma refractory to multiple molecular-targeted agent treatments | 2023 | Jun | Clin J Gastroenterol | 16 | 3 | 438-443 | 10.1007/s12328-023-01777-y |  |
| 801 | Tong, H.; Li, X.; Wei, B.; Tang, C. | Combinative treatment of transarterial chemoembolization, celecoxib and lanreotide in unresectable hepatocellular carcinoma | 2015 | Oct | Clin Res Hepatol Gastroenterol | 39 | 5 | e65-6 | 10.1016/j.clinre.2015.01.008 | Wrong publication type |
| 802 | Tong, H.; Wei, B.; Chen, S.; Xie, Y. M.; Zhang, M. G.; Zhang, L. H.; Huang, Z. Y.; Tang, C. W. | Adjuvant celecoxib and lanreotide following transarterial chemoembolisation for unresectable hepatocellular carcinoma: a randomized pilot study | 2017 | 18-Jul | Oncotarget | 8 | 29 | 48303-48312 | 10.18632/oncotarget.15684 |  |
| 803 | Toprak, N. U.; Sayin, E.; Akilli, F. M.; Gundogdu, A. | Sepsis caused by Anaerococcus nagyae after transarterial-chemoembolization for hepatocellular carcinoma: Case report and literature review | 2021 | Dec | Anaerobe | 72 |  | 102464 | 10.1016/j.anaerobe.2021.102464 |  |
| 804 | Toyama, T.; Nitta, N.; Ohta, S.; Tanaka, T.; Nagatani, Y.; Takahashi, M.; Murata, K.; Shiomi, H.; Naka, S.; Kurumi, Y.; Tani, T.; Tabata, Y. | Clinical trial of cisplatin-conjugated gelatin microspheres for patients with hepatocellular carcinoma | 2012 | Jan | Jpn J Radiol | 30 | 1 | 62-8 | 10.1007/s11604-011-0010-2 |  |
| 805 | Tsai, W. L.; Lai, K. H.; Liang, H. L.; Hsu, P. I.; Chan, H. H.; Chen, W. C.; Yu, H. C.; Tsay, F. W.; Wang, H. M.; Tsai, H. C.; Cheng, J. S. | Hepatic arterial infusion chemotherapy for patients with huge unresectable hepatocellular carcinoma | 2014 |  | PLoS One | 9 | 5 | e92784 | 10.1371/journal.pone.0092784 |  |
| 806 | Tsai, W. L.; Sun, W. C.; Chen, W. C.; Chiang, C. L.; Lin, H. S.; Liang, H. L.; Cheng, J. S. | Hepatic arterial infusion chemotherapy vs transcatheter arterial embolization for patients with huge unresectable hepatocellular carcinoma | 2020 | 7-Aug | Medicine (Baltimore) | 99 | 32 | e21489 | 10.1097/md.0000000000021489 |  |
| 807 | Tsilimigras, D. I.; Pawlik, T. M. | Sorafenib plus transarterial chemoembolization for unresectable hepatocellular carcinoma: Acknowledgments | 2020 | Dec | Ann Transl Med | 8 | 23 | 1557 | 10.21037/atm-20-7228 |  |
| 808 | Tsochatzis, E. A.; Fatourou, E. M.; Triantos, C. K.; Burroughs, A. K. | Transarterial therapies for hepatocellular carcinoma | 2013 |  | Recent Results Cancer Res | 190 |  | 195-206 | 10.1007/978-3-642-16037-0_13 |  |
| 809 | Tsujita, Y.; Sofue, K.; Ueshima, E.; Ueno, Y.; Hori, M.; Tsurusaki, M.; Murakami, T. | Evaluation and Prediction of Treatment Response for Hepatocellular Carcinoma | 2023 | 1-Apr | Magn Reson Med Sci | 22 | 2 | 209-220 | 10.2463/mrms.rev.2022-0118 |  |
| 810 | Turpin, A.; de Baere, T.; Heurgué, A.; Le Malicot, K.; Ollivier-Hourmand, I.; Lecomte, T.; Perrier, H.; Vergniol, J.; Sefrioui, D.; Rinaldi, Y.; Edeline, J.; Jouve, J. L.; Silvain, C.; Becouarn, Y.; Dauvois, B.; Baconnier, M.; Debette-Gratien, M.; Deplanque, G.; Dharancy, S.; Lepage, C.; Hebbar, M. | Liver transarterial chemoembolization and sunitinib for unresectable hepatocellular carcinoma: Results of the PRODIGE 16 study | 2021 | Mar | Clin Res Hepatol Gastroenterol | 45 | 2 | 101464 | 10.1016/j.clinre.2020.05.012 | Wrong patient population |
| 811 | Tzeng, W. S.; Teng, W. L.; Huang, P. H.; Yen, F. L.; Shiue, Y. L. | Anti-cancer activity and cellular uptake of 7,3',4'- and 7,8,4'-trihydroxyisoflavone in HepG2 cells under hypoxic conditions | 2024 | Dec | J Enzyme Inhib Med Chem | 39 | 1 | 2288806 | 10.1080/14756366.2023.2288806 |  |
| 812 | Ueda, S.; Hori, S.; Hori, A.; Makitani, K.; Wan, K.; Sonomura, T. | Retrospective Study of the Efficacy and Safety of Chemoembolization with Drug-Eluting Microspheres Combined with Intra-Arterial Infusion of Bevacizumab for Unresectable Hepatocellular Carcinoma | 2022 |  | J Hepatocell Carcinoma | 9 |  | 973-985 | 10.2147/jhc.S380439 |  |
| 813 | Ueda, T.; Murata, S.; Yasui, D.; Mine, T.; Kumita, S. | Comparison of the antitumor efficacy of transcatheter arterial chemoembolization with a miriplatin-iodized oil suspension and a cisplatin-iodized oil suspension for hepatocellular carcinoma | 2013 | Oct | Hepatol Res | 43 | 10 | 1071-7 | 10.1111/hepr.12212 | Wrong patient population |
| 814 | Uhlig, J.; Lukovic, J.; Dawson, L. A.; Patel, R. A.; Cavnar, M. J.; Kim, H. S. | Locoregional Therapies for Colorectal Cancer Liver Metastases: Options Beyond Resection | 2021 | Mar | Am Soc Clin Oncol Educ Book | 41 |  | 133-146 | 10.1200/edbk_320519 |  |
| 815 | Ursino, S.; Greco, C.; Cartei, F.; Colosimo, C.; Stefanelli, A.; Cacopardo, B.; Berretta, M.; Fiorica, F. | Radiotherapy and hepatocellular carcinoma: update and review of the literature | 2012 | Oct | Eur Rev Med Pharmacol Sci | 16 | 11 | 1599-604 |  |  |
| 816 | Vadot, L.; Boulin, M.; Malbranche, C.; Guiu, B.; Aho, S.; Musat, A.; Pernot, C.; Guignard, M. H.; Hillon, P.; Fagnoni, P. | Result and cost of hepatic chemoembolisation with drug eluting beads in 21 patients | 2013 | Jan | Diagn Interv Imaging | 94 | 1 | 53-9 | 10.1016/j.diii.2012.05.001 | Wrong patient population |
| 817 | Vardar, B. U.; Meram, E.; Karaoglu, K.; Liang, M.; Yu, M.; Laeseke, P.; Ozkan, O. S. | Radioembolization Followed by Transarterial Chemoembolization in Hepatocellular Carcinoma | 2022 | Apr | Cureus | 14 | 4 | e23783 | 10.7759/cureus.23783 |  |
| 818 | Ventura, Y.; Carr, B. I.; Kori, I.; Guerra, V.; Shibolet, O. | Analysis of aggressiveness factors in hepatocellular carcinoma patients undergoing transarterial chemoembolization | 2018 | 21-Apr | World J Gastroenterol | 24 | 15 | 1641-1649 | 10.3748/wjg.v24.i15.1641 |  |
| 819 | Verbus, E. A.; Rossi, A. J.; Teke, M.; Nugent, F. W.; Hernandez, J. M. | Stereotactic Body Radiation Therapy (SBRT) Versus Transarterial Chemoembolization (TACE) as a Bridge to Transplant in Unresectable Hepatocellular Carcinoma | 2022 | Jan | Ann Surg Oncol | 29 | 1 | 33-34 | 10.1245/s10434-021-10278-6 | Wrong study design |
| 820 | Verbus, E. A.; Rossi, A. J.; Teke, M.; Nugent, F. W.; Hernandez, J. M. | Stereotactic Body Radiation Therapy (SBRT) Versus Transarterial Chemoembolization (TACE) as a Bridge to Transplant in Unresectable Hepatocellular Carcinoma | 2022 | Jan | Ann Surg Oncol | 29 | 1 | 33-34 | 10.1245/s10434-021-10278-6 |  |
| 821 | Vogel, A.; Rimassa, L.; Sun, H. C.; Abou-Alfa, G. K.; El-Khoueiry, A.; Pinato, D. J.; Sanchez Alvarez, J.; Daigl, M.; Orfanos, P.; Leibfried, M.; Blanchet Zumofen, M. H.; Gaillard, V. E.; Merle, P. | Comparative Efficacy of Atezolizumab plus Bevacizumab and Other Treatment Options for Patients with Unresectable Hepatocellular Carcinoma: A Network Meta-Analysis | 2021 | Jun | Liver Cancer | 10 | 3 | 240-248 | 10.1159/000515302 |  |
| 822 | Wacker, F.; Dewald, C. | [Local and locoregional treatment of intrahepatic cholangiocarcinoma] | 2022 | Mar | Radiologe | 62 | 3 | 247-252 | 10.1007/s00117-021-00946-9 |  |
| 823 | Wan, X.; Zhai, X.; Yan, Z.; Yang, P.; Li, J.; Wu, D.; Wang, K.; Xia, Y.; Shen, F. | Retrospective analysis of transarterial chemoembolization and sorafenib in Chinese patients with unresectable and recurrent hepatocellular carcinoma | 2016 | 13-Dec | Oncotarget | 7 | 50 | 83806-83816 | 10.18632/oncotarget.11514 | Wrong patient population |
| 824 | Wang, C.; Zhang, L.; Yang, Z.; Zhao, D.; Deng, Z.; Xu, J.; Wu, Y.; Hao, Y.; Dong, Z.; Feng, L.; Liu, Z. | Self-fueling ferroptosis-inducing microreactors based on pH-responsive Lipiodol Pickering emulsions enable transarterial ferro-embolization therapy | 2024 | Jan | Natl Sci Rev | 11 | 1 | nwad257 | 10.1093/nsr/nwad257 |  |
| 825 | Wang, D.; Gaba, R. C.; Jin, B.; Lewandowski, R. J.; Riaz, A.; Memon, K.; Ryu, R. K.; Sato, K. T.; Kulik, L. M.; Mulcahy, M. F.; Larson, A. C.; Salem, R.; Omary, R. A. | Perfusion reduction at transcatheter intraarterial perfusion MR imaging: a promising intraprocedural biomarker to predict transplant-free survival during chemoembolization of hepatocellular carcinoma | 2014 | Aug | Radiology | 272 | 2 | 587-97 | 10.1148/radiol.14131311 |  |
| 826 | Wang, D.; Liu, J.; Li, T.; Wang, Y.; Liu, X.; Bai, Y.; Wang, C.; Ju, S.; Huang, S.; Yang, C.; Zhou, C.; Zhang, Y.; Xiong, B. | A VEGFR targeting peptide-drug conjugate (PDC) suppresses tumor angiogenesis in a TACE model for hepatocellular carcinoma therapy | 2022 | 6-Oct | Cell Death Discov | 8 | 1 | 411 | 10.1038/s41420-022-01198-9 |  |
| 827 | Wang, D.; Rao, W. | Bench-to-bedside development of multifunctional flexible embolic agents | 2023 |  | Theranostics | 13 | 7 | 2114-2139 | 10.7150/thno.80213 |  |
| 828 | Wang, E. A.; Stein, J. P.; Bellavia, R. J.; Broadwell, S. R. | Treatment options for unresectable HCC with a focus on SIRT with Yttrium-90 resin microspheres | 2017 | Nov | Int J Clin Pract | 71 | 11 |  | 10.1111/ijcp.12972 |  |
| 829 | Wang, F. Y.; Meng, W.; Li, Y.; Li, T.; Qin, C. Y. | Comparison of overall survival in patients with unresectable hepatic metastases with or without transarterial chemoembolization: A Propensity Score Matching Study | 2016 | 13-Oct | Sci Rep | 6 |  | 35336 | 10.1038/srep35336 |  |
| 830 | Wang, H.; Cao, C.; Wei, X.; Shen, K.; Shu, Y.; Wan, X.; Sun, J.; Ren, X.; Dong, Y.; Liu, Y.; Zhai, B. | A comparison between drug-eluting bead-transarterial chemoembolization and conventional transarterial chemoembolization in patients with hepatocellular carcinoma: A meta-analysis of six randomized controlled trials | 2020 |  | J Cancer Res Ther | 16 | 2 | 243-249 | 10.4103/jcrt.JCRT_504_19 |  |
| 831 | Wang, H.; Zhu, X.; Zhao, Y.; Dong, D.; Li, L.; Cai, Y.; Li, Y.; Wang, W. | Phase 1 trial of apatinib combined with intensity-modulated radiotherapy in unresectable hepatocellular carcinoma | 2022 | 15-Jul | BMC Cancer | 22 | 1 | 771 | 10.1186/s12885-022-09819-3 |  |
| 832 | Wang, J.; Li, J.; Tang, G.; Tian, Y.; Su, S.; Li, Y. | Clinical outcomes and influencing factors of PD-1/PD-L1 in hepatocellular carcinoma | 2021 | Apr | Oncol Lett | 21 | 4 | 279 | 10.3892/ol.2021.12540 |  |
| 833 | Wang, J.; Zhao, M.; Han, G.; Han, X.; Shi, J.; Mi, L.; Li, N.; Yin, X.; Duan, X.; Hou, J.; Yin, F. | Transarterial Chemoembolization Combined With PD-1 Inhibitors Plus Lenvatinib Showed Improved Efficacy for Treatment of Unresectable Hepatocellular Carcinoma Compared With PD-1 Inhibitors Plus Lenvatinib | 2023 | Jan-Dec | Technol Cancer Res Treat | 22 |  | 1.53E+16 | 10.1177/15330338231166765 |  |
| 834 | Wang, K.; Yu, H. M.; Xiang, Y. J.; Cheng, Y. Q.; Ni, Q. Z.; Guo, W. X.; Shi, J.; Feng, S.; Zhai, J.; Cheng, S. Q. | Transcatheter arterial chemoembolization plus atezolizumab and bevacizumab for unresectable hepatocellular carcinoma: a single-arm, phase II trial | 2022 | Sep | Future Oncol | 18 | 30 | 3367-3375 | 10.2217/fon-2022-0188 |  |
| 835 | Wang, L.; Ke, Q.; Lin, N.; Huang, Q.; Zeng, Y.; Liu, J. | The efficacy of transarterial chemoembolization combined with microwave ablation for unresectable hepatocellular carcinoma: a systematic review and meta-analysis | 2019 |  | Int J Hyperthermia | 36 | 1 | 1288-1296 | 10.1080/02656736.2019.1692148 |  |
| 836 | Wang, M.; Sun, L.; Han, X.; Ren, J.; Li, H.; Wang, W.; Xu, W.; Liang, C.; Duan, X. | The addition of camrelizumab is effective and safe among unresectable hepatocellular carcinoma patients who progress after drug-eluting bead transarterial chemoembolization plus apatinib therapy | 2023 | Jan | Clin Res Hepatol Gastroenterol | 47 | 1 | 102060 | 10.1016/j.clinre.2022.102060 |  |
| 837 | Wang, P.; Sheng, L.; Wang, G.; Wang, H.; Huang, X.; Yan, X.; Yang, X.; Pei, R. | Association of transarterial chemoembolization with survival in patients with unresectable hepatocellular carcinoma | 2014 | Mar | Mol Clin Oncol | 2 | 2 | 203-206 | 10.3892/mco.2014.239 |  |
| 838 | Wang, Q.; Xia, D.; Bai, W.; Wang, E.; Sun, J.; Huang, M.; Mu, W.; Yin, G.; Li, H.; Zhao, H.; Li, J.; Zhang, C.; Zhu, X.; Wu, J.; Li, J.; Gong, W.; Li, Z.; Lin, Z.; Pan, X.; Shi, H.; Shao, G.; Liu, J.; Yang, S.; Zheng, Y.; Xu, J.; Song, J.; Wang, W.; Wang, Z.; Zhang, Y.; Ding, R.; Zhang, H.; Yu, H.; Zheng, L.; Gu, W.; You, N.; Wang, G.; Zhang, S.; Feng, L.; Liu, L.; Zhang, P.; Li, X.; Chen, J.; Xu, T.; Zhou, W.; Zeng, H.; Zhang, Y.; Huang, W.; Jiang, W.; Zhang, W.; Shao, W.; Li, L.; Niu, J.; Yuan, J.; Li, X.; Lv, Y.; Li, K.; Yin, Z.; Xia, J.; Fan, D.; Han, G. | Development of a prognostic score for recommended TACE candidates with hepatocellular carcinoma: A multicentre observational study | 2019 | May | J Hepatol | 70 | 5 | 893-903 | 10.1016/j.jhep.2019.01.013 |  |
| 839 | Wang, T.; Du, Y. N.; Sun, J.; Song, H.; Jiang, Y.; Liu, F.; Lv, X. | Drug-eluting bead transarterial chemoembolization could improve the hepatic hemodynamics of patients with unresectable hepatocellular carcinoma: a retrospective cohort study | 2023 | 28-Feb | J Gastrointest Oncol | 14 | 1 | 302-311 | 10.21037/jgo-23-76 |  |
| 840 | Wang, W. J.; Liu, Z. H.; Wang, K.; Yu, H. M.; Cheng, Y. Q.; Xiang, Y. J.; Feng, J. K.; Zhou, L. P.; Zhou, H. K.; Pan, W. W.; Guo, W. X.; Shi, J.; Cheng, S. Q. | Efficacy and safety of TACE combined with lenvatinib and PD-1 inhibitors for unresectable recurrent HCC: A multicenter, retrospective study | 2023 | May | Cancer Med | 12 | 10 | 11513-11524 | 10.1002/cam4.5880 | Wrong patient population |
| 841 | Wang, W.; Bai, W.; Wang, E.; Zhao, Y.; Liu, L.; Yang, M.; Cai, H.; Xia, D.; Zhang, L.; Niu, J.; Yin, Z.; Zhang, Z.; Fan, D.; Xia, J.; Han, G. | mRECIST response combined with sorafenib-related adverse events is superior to either criterion alone in predicting survival in HCC patients treated with TACE plus sorafenib | 2017 | 15-Jan | Int J Cancer | 140 | 2 | 390-399 | 10.1002/ijc.30451 |  |
| 842 | Wang, X.; Yarmohammadi, H.; Cao, G.; Ji, X.; Hu, J.; Yarmohammadi, H.; Chen, H.; Zhu, X.; Yang, R.; Solomon, S. B. | Dual phase cone-beam computed tomography in detecting <3 cm hepatocellular carcinomas during transarterial chemoembolization | 2017 | Jan-Mar | J Cancer Res Ther | 13 | 1 | 38-43 | 10.4103/0973-1482.206242 |  |
| 843 | Wang, Y. Y.; Yang, X.; Wang, Y. C.; Long, J. Y.; Sun, H. S.; Li, Y. R.; Xun, Z. Y.; Zhang, N.; Xue, J. N.; Ning, C.; Zhang, J. W.; Zhu, C. P.; Zhang, L. H.; Yang, X. B.; Zhao, H. T. | Clinical outcomes of lenvatinib plus transarterial chemoembolization with or without programmed death receptor-1 inhibitors in unresectable hepatocellular carcinoma | 2023 | 14-Mar | World J Gastroenterol | 29 | 10 | 1614-1626 | 10.3748/wjg.v29.i10.1614 |  |
| 844 | Wang, Y.; Lin, W.; Huang, G.; Nie, S.; Yu, Q.; Hou, F.; Zong, S. | The therapeutic principle of combined clearing heat and resolving toxin plus TACE on primary liver cancer: A systematic review and meta-analysis | 2024 | 30-Jan | J Ethnopharmacol | 319 | Pt 1 | 117072 | 10.1016/j.jep.2023.117072 |  |
| 845 | Wang, Z. X.; Wang, E. X.; Bai, W.; Xia, D. D.; Mu, W.; Li, J.; Yang, Q. Y.; Huang, M.; Xu, G. H.; Sun, J. H.; Li, H. L.; Zhao, H.; Wu, J. B.; Yang, S. F.; Li, J. P.; Li, Z. X.; Zhang, C. Q.; Zhu, X. L.; Zheng, Y. B.; Wang, Q. H.; Li, J.; Yuan, J.; Li, X. M.; Niu, J.; Yin, Z. X.; Xia, J. L.; Fan, D. M.; Han, G. H.; On Behalf Of China Hcc-Tace Study, Group | Validation and evaluation of clinical prediction systems for first and repeated transarterial chemoembolization in unresectable hepatocellular carcinoma: A Chinese multicenter retrospective study | 2020 | 14-Feb | World J Gastroenterol | 26 | 6 | 657-669 | 10.3748/wjg.v26.i6.657 |  |
| 846 | Wang, Z. Y.; Xie, C. F.; Feng, K. L.; Xiong, C. M.; Huang, J. H.; Chen, Q. L.; Zhong, C.; Zhou, Z. W. | Drug-eluting beads versus conventional transarterial chemoembolization for the treatment of unresectable hepatocellular carcinoma: A meta-analysis | 2023 | 25-Aug | Medicine (Baltimore) | 102 | 34 | e34527 | 10.1097/md.0000000000034527 | Wrong study design |
| 847 | Wang, Z.; Chapiro, J.; Schernthaner, R.; Duran, R.; Chen, R.; Geschwind, J. F.; Lin, M. | Multimodality 3D Tumor Segmentation in HCC Patients Treated with TACE | 2015 | Jul | Acad Radiol | 22 | 7 | 840-5 | 10.1016/j.acra.2015.03.001 |  |
| 848 | Wang, Z.; Hansis, E.; Chen, R.; Duran, R.; Chapiro, J.; Sheu, Y. R.; Kobeiter, H.; Grass, M.; Geschwind, J. F.; Lin, M. | Automatic bone removal for 3D TACE planning with C-arm CBCT: Evaluation of technical feasibility | 2016 |  | Minim Invasive Ther Allied Technol | 25 | 3 | 162-70 | 10.3109/13645706.2015.1129970 |  |
| 849 | Wang, Z.; Lin, M.; Lesage, D.; Chen, R.; Chapiro, J.; Gu, T.; Tacher, V.; Duran, R.; Geschwind, J. F. | Three-dimensional evaluation of lipiodol retention in HCC after chemoembolization: a quantitative comparison between CBCT and MDCT | 2014 | Mar | Acad Radiol | 21 | 3 | 393-9 | 10.1016/j.acra.2013.11.006 |  |
| 850 | Wang, Z.; Peng, Y.; Hu, J.; Wang, X.; Sun, H.; Sun, J.; Shi, Y.; Xiao, Y.; Ding, Z.; Yang, X.; Tang, M.; Tang, Z.; Wang, J.; Lau, W. Y.; Fan, J.; Zhou, J. | Associating Liver Partition and Portal Vein Ligation for Staged Hepatectomy for Unresectable Hepatitis B Virus-related Hepatocellular Carcinoma: A Single Center Study of 45 Patients | 2020 | Mar | Ann Surg | 271 | 3 | 534-541 | 10.1097/sla.0000000000002942 |  |
| 851 | Wang, Z.; Wang, E.; Bai, W.; Xia, D.; Ding, R.; Li, J.; Wang, Q.; Liu, L.; Sun, J.; Mu, W.; Zhao, H.; Pan, X.; Shao, G.; Zhu, X.; Yin, G.; Shi, H.; Wu, J.; Lin, Z.; Yang, S.; Liu, J.; Wang, W.; Zhu, X.; Lv, Y.; Li, J.; Chen, H.; Wang, W.; Li, K.; Yuan, X.; Yu, T.; Yuan, J.; Li, X.; Niu, J.; Yin, Z.; Xia, J.; Fan, D.; Han, G. | Exploratory Analysis to Identify Candidates Benefitting from Combination Therapy of Transarterial Chemoembolization and Sorafenib for First-Line Treatment of Unresectable Hepatocellular Carcinoma: A Multicenter Retrospective Observational Study | 2020 | Jun | Liver Cancer | 9 | 3 | 308-325 | 10.1159/000505692 | Wrong patient population |
| 852 | Wei, J.; Cui, W.; Fan, W.; Wang, Y.; Li, J. | Unresectable Hepatocellular Carcinoma: Transcatheter Arterial Chemoembolization Combined With Microwave Ablation vs. Combined With Cryoablation | 2020 |  | Front Oncol | 10 |  | 1285 | 10.3389/fonc.2020.01285 |  |
| 853 | Wei, Y. G.; Su, H.; Lv, Z. L.; Liao, X. W.; Zeng, Z. M.; Jia, Y. X.; Huang, H. S.; Shen, X. Q.; Zhu, G. Z.; Han, C. Y.; Ye, X. P.; Peng, T. | Case Report: A case of hepatocellular carcinoma with aberrant right hepatic artery treated with transarterial chemoembolization and infusion chemotherapy separately to bilobar lesion combining with systemic therapies and sequential hepatectomy | 2023 |  | Front Oncol | 13 |  | 1165538 | 10.3389/fonc.2023.1165538 |  |
| 854 | Wei, Y.; Liu, J.; Yan, M.; Zhao, S.; Long, Y.; Zhang, W. | Effectiveness and Safety of Combination Therapy of Transarterial Chemoembolization and Apatinib for Unresectable Hepatocellular Carcinoma in the Chinese Population: A Meta-Analysis | 2019 |  | Chemotherapy | 64 | 2 | 94-104 | 10.1159/000502510 |  |
| 855 | Weintraub, J. L.; Salem, R. | Treatment of hepatocellular carcinoma combining sorafenib and transarterial locoregional therapy: state of the science | 2013 | Aug | J Vasc Interv Radiol | 24 | 8 | 1123-34 | 10.1016/j.jvir.2013.01.494 |  |
| 856 | Weiss, K. E.; Sze, D. Y.; Rangaswami, A. A.; Esquivel, C. O.; Concepcion, W.; Lebowitz, E. A.; Kothary, N.; Lungren, M. P. | Transarterial chemoembolization in children to treat unresectable hepatocellular carcinoma | 2018 | Jun | Pediatr Transplant | 22 | 4 | e13187 | 10.1111/petr.13187 |  |
| 857 | White, J. A.; Gray, S. H.; Li, P.; Simpson, H. N.; McGuire, B. M.; Eckhoff, D. E.; Abdel Aal, A. M. K.; Saddekni, S.; Dubay, D. A. | Current guidelines for chemoembolization for hepatocellular carcinoma: Room for improvement? | 2017 | Jun | Hepatol Commun | 1 | 4 | 338-346 | 10.1002/hep4.1046 |  |
| 858 | Wholey, M.; Palacios Iii, R.; Wholey, D.; Mendez, A. | Safety and Long-Term Survival Outcome in Patients With Unresectable Barcelona Clinic Liver Cancer (BCLC) Stages C and D Advanced Hepatocellular Carcinoma Treated With 40 µm Drug-Eluting Bead Transcatheter Arterial Chemoembolization | 2022 | Apr | Cureus | 14 | 4 | e24047 | 10.7759/cureus.24047 |  |
| 859 | Willatt, J.; Hannawa, K. K.; Ruma, J. A.; Frankel, T. L.; Owen, D.; Barman, P. M. | Image-guided therapies in the treatment of hepatocellular carcinoma: A multidisciplinary perspective | 2015 | 27-Feb | World J Hepatol | 7 | 2 | 235-44 | 10.4254/wjh.v7.i2.235 |  |
| 860 | Wo, J. Y.; Dawson, L. A.; Zhu, A. X.; Hong, T. S. | An emerging role for radiation therapy in the treatment of hepatocellular carcinoma and intrahepatic cholangiocarcinoma | 2014 | Apr | Surg Oncol Clin N Am | 23 | 2 | 353-68 | 10.1016/j.soc.2013.10.007 |  |
| 861 | Wong, T. C.; Chiang, C. L.; Lee, A. S.; Lee, V. H.; Yeung, C. S.; Ho, C. H.; Cheung, T. T.; Ng, K. K.; Chok, S. H.; Chan, A. C.; Dai, W. C.; Wong, F. C.; Luk, M. Y.; Leung, T. W.; Lo, C. M. | Better survival after stereotactic body radiation therapy following transarterial chemoembolization in nonresectable hepatocellular carcinoma: A propensity score matched analysis | 2019 | Mar | Surg Oncol | 28 |  | 228-235 | 10.1016/j.suronc.2019.01.006 | Wrong patient population |
| 862 | Wu, G. C.; Chan, E. D.; Chou, Y. C.; Yu, C. Y.; Hsieh, T. Y.; Hsieh, C. B.; Chian, C. F.; Ke, F. C.; Dai, Y. L.; Su, W. L. | Risk factors for the development of pulmonary oil embolism after transcatheter arterial chemoembolization of hepatic tumors | 2014 | Sep | Anticancer Drugs | 25 | 8 | 976-81 | 10.1097/cad.0000000000000113 |  |
| 863 | Wu, J. L.; Luo, J. Y.; Jiang, Z. B.; Huang, S. B.; Chen, G. R.; Ran, H. Y.; Liang, Q. Y.; Huang, M. S.; Lai, L. S.; Chen, J. W. | Inflammation-related nomogram for predicting survival of patients with unresectable hepatocellular carcinoma received conversion therapy | 2023 | 28-May | World J Gastroenterol | 29 | 20 | 3168-3184 | 10.3748/wjg.v29.i20.3168 |  |
| 864 | Wu, J. Y.; Wu, J. Y.; Li, Y. N.; Qiu, F. N.; Zhou, S. Q.; Yin, Z. Y.; Chen, Y. F.; Li, B.; Zhou, J. Y.; Yan, M. L. | Lenvatinib combined with anti-PD-1 antibodies plus transcatheter arterial chemoembolization for neoadjuvant treatment of resectable hepatocellular carcinoma with high risk of recurrence: A multicenter retrospective study | 2022 |  | Front Oncol | 12 |  | 985380 | 10.3389/fonc.2022.985380 |  |
| 865 | Wu, J. Y.; Yin, Z. Y.; Bai, Y. N.; Chen, Y. F.; Zhou, S. Q.; Wang, S. J.; Zhou, J. Y.; Li, Y. N.; Qiu, F. N.; Li, B.; Yan, M. L. | Lenvatinib Combined with Anti-PD-1 Antibodies Plus Transcatheter Arterial Chemoembolization for Unresectable Hepatocellular Carcinoma: A Multicenter Retrospective Study | 2021 |  | J Hepatocell Carcinoma | 8 |  | 1233-1240 | 10.2147/jhc.S332420 |  |
| 866 | Wu, J. Y.; Zhang, Z. B.; Zhou, J. Y.; Ke, J. P.; Bai, Y. N.; Chen, Y. F.; Wu, J. Y.; Zhou, S. Q.; Wang, S. J.; Zeng, Z. X.; Li, Y. N.; Qiu, F. N.; Li, B.; Yan, M. L. | Outcomes of Salvage Surgery for Initially Unresectable Hepatocellular Carcinoma Converted by Transcatheter Arterial Chemoembolization Combined with Lenvatinib plus Anti-PD-1 Antibodies: A Multicenter Retrospective Study | 2023 | Aug | Liver Cancer | 12 | 3 | 229-237 | 10.1159/000528356 |  |
| 867 | Wu, J.; Ng, J.; Christos, P. J.; Goldenberg, A. S.; Sparano, J.; Sung, M. W.; Hochster, H. S.; Muggia, F. M. | Chronic thalidomide and chemoembolization for hepatocellular carcinoma | 2014 | Dec | Oncologist | 19 | 12 | 1229-30 | 10.1634/theoncologist.2014-0283 |  |
| 868 | Wu, J.; Song, L.; Zhao, D. Y.; Guo, B.; Liu, J. | Chemotherapy for transarterial chemoembolization in patients with unresectable hepatocellular carcinoma | 2014 | 21-Aug | World J Gastroenterol | 20 | 31 | 10960-8 | 10.3748/wjg.v20.i31.10960 |  |
| 869 | Wu, Ke-Tong; Wang, Cun-Chuan; Lu, Li-Gong; Zhang, Wei-Dong; Zhang, Fu-Jun; Shi, Feng; Li, Chuan-Xing | Hepatocellular carcinoma: clinical study of long-term survival and choice of treatment modalities | 2013 |  | World journal of gastroenterology: WJG | 19 | 23 | 3649 |  | Wrong patient population |
| 870 | Wu, L. F.; Rao, S. X.; Xu, P. J.; Yang, L.; Chen, C. Z.; Liu, H.; Huang, J. F.; Fu, C. X.; Halim, A.; Zeng, M. S. | Pre-TACE kurtosis of ADC(total) derived from histogram analysis for diffusion-weighted imaging is the best independent predictor of prognosis in hepatocellular carcinoma | 2019 | Jan | Eur Radiol | 29 | 1 | 213-223 | 10.1007/s00330-018-5482-3 |  |
| 871 | Wu, L.; Xu, P.; Rao, S.; Yang, L.; Chen, C.; Liu, H.; Fu, C.; Zeng, M. | ADC(total) ratio and D ratio derived from intravoxel incoherent motion early after TACE are independent predictors for survival in hepatocellular carcinoma | 2017 | Sep | J Magn Reson Imaging | 46 | 3 | 820-830 | 10.1002/jmri.25617 | Wrong patient population |
| 872 | Wu, M.; Gao, S.; Song, H.; Zhang, Z.; Wang, J.; Liu, R.; Wang, X.; Cheng, J.; Luo, J.; Liu, Q.; Chen, Y.; Yan, Z.; Liu, L. | Percutaneous thermal ablation combined with simultaneous transarterial chemoembolization for hepatocellular carcinoma ≤ 5 cm | 2019 |  | J Cancer Res Ther | 15 | 4 | 766-772 | 10.4103/jcrt.JCRT_250_19 |  |
| 873 | Wu, S. J.; Ruan, D. D.; Wu, Q. Y.; Tang, Y.; Zhang, J. H.; Cai, S. L.; Zhou, Y. F.; Luo, J. W.; Fang, Z. T. | Safety and Efficacy of Drug-Eluting Bead Transarterial Chemoembolization Combined with Lenvatinib and Anti-PD-1 Antibodies for Unresectable Hepatocellular Carcinoma: A Retrospective Analysis | 2023 |  | J Hepatocell Carcinoma | 10 |  | 807-820 | 10.2147/jhc.S408819 |  |
| 874 | Wu, S.; Fan, K.; Yang, Q.; Chen, Z.; Hou, Y.; Zou, Y.; Cai, W.; Kang, L. | Smart nanoparticles and microbeads for interventional embolization therapy of liver cancer: state of the art | 2023 | 6-Feb | J Nanobiotechnology | 21 | 1 | 42 | 10.1186/s12951-023-01804-7 |  |
| 875 | Wu, X.; Chapiro, J.; Malhotra, A.; Kothary, N. | Comparison of Drug-Eluting Embolics versus Conventional Transarterial Chemoembolization for the Treatment of Patients with Unresectable Hepatocellular Carcinoma: A Cost-Effectiveness Analysis | 2021 | Jan | J Vasc Interv Radiol | 32 | 1 | 2-12.e1 | 10.1016/j.jvir.2020.09.022 |  |
| 876 | Wu, Y.; Qi, H.; Cao, F.; Shen, L.; Chen, S.; Xie, L.; Huang, T.; Song, Z.; Zhou, D.; Fan, W. | TACE-Sorafenib With Thermal Ablation Has Survival Benefits in Patients With Huge Unresectable Hepatocellular Carcinoma | 2020 |  | Front Pharmacol | 11 |  | 1130 | 10.3389/fphar.2020.01130 | Wrong patient population |
| 877 | Xia, D.; Bai, W.; Wang, E.; Li, J.; Chen, X.; Wang, Z.; Huang, M.; Huang, M.; Sun, J.; Yang, W.; Lin, Z.; Wu, J.; Li, Z.; Yang, S.; Zhu, X.; Chen, Z.; Zhang, Y.; Fan, W.; Mai, Q.; Ding, R.; Nie, C.; Feng, L.; Li, X.; Huang, W.; Sun, J.; Wang, Q.; Lv, Y.; Li, X.; Luo, B.; Wang, Z.; Yuan, J.; Guo, W.; Li, K.; Li, B.; Li, R.; Yin, Z.; Xia, J.; Han, G. | Lenvatinib with or without Concurrent Drug-Eluting Beads Transarterial Chemoembolization in Patients with Unresectable, Advanced Hepatocellular Carcinoma: A Real-World, Multicenter, Retrospective Study | 2022 | Jul | Liver Cancer | 11 | 4 | 368-382 | 10.1159/000523849 |  |
| 878 | Xia, D.; Wang, Q.; Bai, W.; Wang, E.; Wang, Z.; Mu, W.; Sun, J.; Huang, M.; Yin, G.; Li, H.; Zhao, H.; Zhang, C.; Li, J.; Wu, J.; Zhu, X.; Yang, S.; Pan, X.; Li, J.; Li, Z.; Xu, G.; Shi, H.; Zhang, H.; Zhang, Y.; Ding, R.; Yu, H.; Zheng, L.; Yang, X.; Wang, G.; You, N.; Feng, L.; Zhang, S.; Huang, W.; Xu, T.; Fan, W.; Li, X.; Yang, X.; Zhou, W.; Wang, W.; Li, X.; Wang, Z.; Luo, B.; Niu, J.; Yuan, J.; Lv, Y.; Li, K.; Guo, W.; Yin, Z.; Fan, D.; Xia, J.; Han, G. | Optimal time point of response assessment for predicting survival is associated with tumor burden in hepatocellular carcinoma receiving repeated transarterial chemoembolization | 2022 | Sep | Eur Radiol | 32 | 9 | 5799-5810 | 10.1007/s00330-022-08716-4 | Wrong patient population |
| 879 | Xia, W. L.; Xu, S. J.; Guo, Y.; Zhao, X. H.; Hu, H. T.; Zhao, Y.; Yao, Q. J.; Zheng, L.; Zhang, D. Y.; Guo, C. Y.; Fan, W. J.; Li, H. L. | Plasma arginase-1 as a predictive marker for early transarterial chemoembolization refractoriness in unresectable hepatocellular carcinoma | 2022 |  | Front Oncol | 12 |  | 1014653 | 10.3389/fonc.2022.1014653 |  |
| 880 | Xia, W. L.; Zhao, X. H.; Guo, Y.; Cao, G. S.; Wu, G.; Fan, W. J.; Yao, Q. J.; Xu, S. J.; Guo, C. Y.; Hu, H. T.; Li, H. L. | Transarterial chemoembolization combined with apatinib with or without PD-1 inhibitors in BCLC stage C hepatocellular carcinoma: A multicenter retrospective study | 2022 |  | Front Oncol | 12 |  | 961394 | 10.3389/fonc.2022.961394 |  |
| 881 | Xia, Z.; Zhao, W.; Liu, J.; Zhang, J.; Pan, J.; Chen, K.; Wang, L.; Zhao, H.; Chen, X. | A Three-Gene Signature for Predicting the Prognosis of Patients Treated with Transarterial Chemoembolization (TACE) and Identification of PD-184352 as a Potential Drug to Reverse Nonresponse to TACE | 2022 |  | J Oncol | 2022 |  | 2704862 | 10.1155/2022/2704862 |  |
| 882 | Xiang, Z.; Li, G.; Mu, L.; Wang, H.; Zhou, C.; Yan, H.; Huang, M. | TACE Combined with Lenvatinib and Camrelizumab for Unresectable Multiple Nodular and Large Hepatocellular Carcinoma (>5 cm) | 2023 | Jan-Dec | Technol Cancer Res Treat | 22 |  | 1.53E+16 | 10.1177/15330338231200320 |  |
| 883 | Xiao, Y. D.; Ma, C.; Zhang, Z. S.; Liu, J. | Safety and efficacy assessment of transarterial chemoembolization using drug-eluting beads in patients with hepatocellular carcinoma and arterioportal shunt: a single-center experience | 2019 |  | Cancer Manag Res | 11 |  | 1551-1557 | 10.2147/cmar.S193948 | Wrong patient population |
| 884 | Xie, D. Y.; Zhu, K.; Ren, Z. G.; Zhou, J.; Fan, J.; Gao, Q. | A review of 2022 Chinese clinical guidelines on the management of hepatocellular carcinoma: updates and insights | 2023 | 10-Apr | Hepatobiliary Surg Nutr | 12 | 2 | 216-228 | 10.21037/hbsn-22-469 |  |
| 885 | Xie, D.; Sun, Q.; Wang, X.; Zhou, J.; Fan, J.; Ren, Z.; Gao, Q. | Immune checkpoint inhibitor plus tyrosine kinase inhibitor for unresectable hepatocellular carcinoma in the real world | 2021 | Apr | Ann Transl Med | 9 | 8 | 652 | 10.21037/atm-20-7037 |  |
| 886 | Xie, F.; Zang, J.; Guo, X.; Xu, F.; Shen, R.; Yan, L.; Yang, J.; He, J. | Comparison of transcatheter arterial chemoembolization and microsphere embolization for treatment of unresectable hepatocellular carcinoma: a meta-analysis | 2012 | Mar | J Cancer Res Clin Oncol | 138 | 3 | 455-62 | 10.1007/s00432-011-1117-7 | Wrong patient population |
| 887 | Xie, H.; Yu, H.; Tian, S.; Yang, X.; Wang, X.; Yang, Z.; Wang, H.; Guo, Z. | What is the best combination treatment with transarterial chemoembolization of unresectable hepatocellular carcinoma? a systematic review and network meta-analysis | 2017 | 21-Nov | Oncotarget | 8 | 59 | 100508-100523 | 10.18632/oncotarget.20119 | Wrong patient population |
| 888 | Xie, L. L.; Sun, C. J.; Li, X. D.; Wang, Y. H.; Wang, C. E. | Arterial embolization of massive hepatocellular carcinoma with lipiodol and gelatin sponge | 2015 | Feb | Indian J Cancer | 51 Suppl 2 |  | e49-51 | 10.4103/0019-509x.151990 |  |
| 889 | Xie, Zhi-Bo; Wang, Xiao-Bo; Peng, Yu-Chong; Zhu, Shao-Liang; Ma, Liang; Xiang, Bang-De; Gong, Wen-Feng; Chen, Jie; You, Xue-Mei; Jiang, Jing-Hang | Systematic review comparing the safety and efficacy of conventional and drug-eluting bead transarterial chemoembolization for inoperable hepatocellular carcinoma | 2015 |  | Hepatology Research | 45 | 2 | 190-200 |  | Wrong patient population |
| 890 | Xin, Y.; Zhang, X.; Liu, N.; Peng, G.; Huang, X.; Cao, X.; Zhou, X.; Li, X. | Efficacy and safety of lenvatinib plus PD-1 inhibitor with or without transarterial chemoembolization in unresectable hepatocellular carcinoma | 2023 | Jun | Hepatol Int | 17 | 3 | 753-764 | 10.1007/s12072-023-10502-3 |  |
| 891 | Xin'an, L.; Jianying, Z.; Lizhi, N.; Fei, Y.; Xiaohua, W.; Jibing, C.; Jialiang, L.; Kecheng, X. | Alleviating the pain of unresectable hepatic tumors by percutaneous cryoablation: experience in 73 patients | 2013 | Dec | Cryobiology | 67 | 3 | 369-73 |  |  |
| 892 | Xing, M.; Kokabi, N.; Camacho, J. C.; Kooby, D. A.; El-Rayes, B. F.; Kim, H. S. | 90Y radioembolization versus chemoembolization in the treatment of hepatocellular carcinoma: an analysis of comparative effectiveness | 2013 | Jul | J Comp Eff Res | 2 | 4 | 435-44 | 10.2217/cer.13.37 | Wrong patient population |
| 893 | Xing, M.; Kokabi, N.; Prajapati, H. J.; Close, O.; Ludwig, J. M.; Kim, H. S. | Survival in unresectable AJCC stage I and II HCC and the effect of DEB-TACE: SEER versus tertiary cancer center cohort study | 2016 | Mar | J Comp Eff Res | 5 | 2 | 141-54 | 10.2217/cer.15.54 | Wrong outcomes |
| 894 | Xing, M.; Webber, G.; Prajapati, H. J.; Chen, Z.; El-Rayes, B.; Spivey, J. R.; Pillai, A. A.; Kim, H. S. | Preservation of quality of life with doxorubicin drug-eluting bead transarterial chemoembolization for unresectable hepatocellular carcinoma: Longitudinal prospective study | 2015 | Jul | J Gastroenterol Hepatol | 30 | 7 | 1167-74 | 10.1111/jgh.12920 |  |
| 895 | Xiong, J.; He, D.; Hu, W.; Liu, X. | Retroperitoneal and intrahepatic metastasis from primary clear cell carcinoma of the liver: A case report and review of the literature | 2017 | Mar | Medicine (Baltimore) | 96 | 12 | e6452 | 10.1097/md.0000000000006452 |  |
| 896 | Xu, H.; Deng, Y.; Zhou, Z.; Huang, Y. | Chinese Herbal Medicine (Chaihu-Huaji Decoction) Alleviates Postembolization Syndrome following Transcatheter Arterial Chemoembolization and Improves Survival in Unresectable Hepatocellular Cancer: A Retrospective Study | 2019 |  | Evid Based Complement Alternat Med | 2019 |  | 6269518 | 10.1155/2019/6269518 |  |
| 897 | Xu, L. F.; Sun, H. L.; Chen, Y. T.; Ni, J. Y.; Chen, D.; Luo, J. H.; Zhou, J. X.; Hu, R. M.; Tan, Q. Y. | Large primary hepatocellular carcinoma: transarterial chemoembolization monotherapy versus combined transarterial chemoembolization-percutaneous microwave coagulation therapy | 2013 | Mar | J Gastroenterol Hepatol | 28 | 3 | 456-63 | 10.1111/jgh.12088 | Wrong patient population |
| 898 | Xu, L.; Peng, Z. W.; Chen, M. S.; Shi, M.; Zhang, Y. J.; Guo, R. P.; Lin, X. J.; Lau, W. Y. | Prognostic nomogram for patients with unresectable hepatocellular carcinoma after transcatheter arterial chemoembolization | 2015 | Jul | J Hepatol | 63 | 1 | 122-30 | 10.1016/j.jhep.2015.02.034 |  |
| 899 | Xu, L.; Wang, S.; Wang, S.; Wang, Y.; Li, W.; Lin, G.; Yuan, Z. | Baseline apparent diffusion coefficients: Validation study of new predictor of survival in patients with unresectable hepatocellular carcinoma following chemoembolization | 2021 |  | J Xray Sci Technol | 29 | 3 | 507-516 | 10.3233/xst-200827 |  |
| 900 | Xu, L.; Zhao, D.; Tian, P.; Ding, J.; Jiang, Z.; Ni, G.; Hou, Z.; Ni, C. | Development and Validation of a Prognostic Model for Transarterial Chemoembolization in Unresectable Hepatocellular Carcinoma Based on Preoperative Serum Prealbumin | 2023 |  | J Hepatocell Carcinoma | 10 |  | 2239-2250 | 10.2147/jhc.S433245 |  |
| 901 | Xu, Q.; Huang, Y.; Shi, H.; Song, Q.; Xu, Y. | Sunitinib versus sorafenib plus transarterial chemoembolization for inoperable hepatocellular carcinoma patients | 2018 | Jan-Feb | J buon | 23 | 1 | 193-199 |  | Wrong patient population |
| 902 | Xu, X.; Gao, D.; Yuan, X.; Liu, L. I.; Zhang, X.; Liang, X.; Chen, S.; Ai, M.; Chen, B. O.; Shi, D.; Yang, Z.; Hoffman, R. M.; Xu, J. | β-Catenin Expression Correlates With Prognosis in Hepatocellular Carcinoma Patients Treated With Transcatheter Arterial Chemoembolization | 2019 | Mar | Anticancer Res | 39 | 3 | 1129-1134 | 10.21873/anticanres.13221 | Wrong patient population |
| 903 | Xu, Y. J.; Lai, Z. C.; He, M. K.; Bu, X. Y.; Chen, H. W.; Zhou, Y. M.; Xu, L.; Wei, W.; Zhang, Y. J.; Chen, M. S.; Guo, R. P.; Shi, M.; Li, Q. J. | Toripalimab Combined With Hepatic Arterial Infusion Chemotherapy Versus Lenvatinib for Advanced Hepatocellular Carcinoma | 2021 | Jan-Dec | Technol Cancer Res Treat | 20 |  | 1.53303E+16 | 10.1177/15330338211063848 |  |
| 904 | Xu, Y.; Wang, T.; Zeng, J.; Wang, B.; Zhou, L.; Yang, M.; Zhang, L.; Zhang, N. | Integrative Functional Genomics Implicated the Key T-/B-Cell Deficiency Regulator RAG1 in Transarterial Chemoembolization of Hepatocellular Carcinoma | 2021 |  | Front Cell Dev Biol | 9 |  | 720791 | 10.3389/fcell.2021.720791 |  |
| 905 | Xu, Z.; Yu, C.; Wang, S.; Xu, G. | Transcatheter arterial chemoembolization as an examination method for hepatocellular carcinoma undetected by B-mode ultrasound, computed tomography and digital subtratcion angiography: A case report | 2015 | Sep | Oncol Lett | 10 | 3 | 1759-1762 | 10.3892/ol.2015.3446 |  |
| 906 | Xue, J.; Ni, H.; Wang, F.; Xu, K.; Niu, M. | Advances in locoregional therapy for hepatocellular carcinoma combined with immunotherapy and targeted therapy | 2021 | Aug | J Interv Med | 4 | 3 | 105-113 | 10.1016/j.jimed.2021.05.002 |  |
| 907 | Xue, T.; Feng, W.; Yu, H.; Zhu, M.; Fei, M.; Bao, Y.; Wang, X.; Ma, W.; Lv, G.; Guan, J.; Chen, S. | Metastasis-Associated Protein 1 Is Involved in Angiogenesis after Transarterial Chemoembolization Treatment | 2017 |  | Biomed Res Int | 2017 |  | 6757898 | 10.1155/2017/6757898 |  |
| 908 | Yamada, R.; Bassaco, B.; Bracewell, S.; Gillen, K.; Kocher, M.; Collins, H.; Anderson, M. B.; Guimaraes, M. | Long-term follow-up after conventional transarterial chemoembolization (c-TACE) with mitomycin for hepatocellular carcinoma (HCC) | 2019 | Apr | J Gastrointest Oncol | 10 | 2 | 348-353 | 10.21037/jgo.2019.01.01 | Wrong patient population |
| 909 | Yamada, R.; Bassaco, B.; Bracewell, S.; Volin, S.; Collins, H.; Hannegan, C.; Guimarares, M. | Combined conventional transarterial chemoembolization with Mitomycin and percutaneous ablation for unresectable hepatocellular carcinoma | 2020 | Apr | J Gastrointest Oncol | 11 | 2 | 298-303 | 10.21037/jgo.2019.01.07 | Wrong patient population |
| 910 | Yan, H.; Wang, X.; Zhou, D.; Wang, P.; Yang, Z. | Dynamic Nomogram for Predicting Macrovascular Invasion of Patients with Unresectable Hepatocellular Carcinoma after Transarterial Chemoembolization | 2022 |  | J Cancer | 13 | 6 | 1914-1922 | 10.7150/jca.69548 |  |
| 911 | Yan, L.; Chen, L.; Qian, K.; Kan, X.; Zhang, H.; Liang, B.; Zheng, C. | Caudate Lobe Hepatocellular Carcinoma Treated with Sequential Transarterial Chemoembolization and Iodine 125 Seeds Implantation: A Single-Center Retrospective Study | 2021 |  | Cancer Manag Res | 13 |  | 3901-3912 | 10.2147/cmar.S309310 | Wrong intervention |
| 912 | Yan, L.; Ren, Y.; Qian, K.; Kan, X.; Zhang, H.; Chen, L.; Liang, B.; Zheng, C. | Sequential transarterial chemoembolization and early radiofrequency ablation improves clinical outcomes for early-intermediate hepatocellular carcinoma in a 10-year single-center comparative study | 2021 | 20-Apr | BMC Gastroenterol | 21 | 1 | 182 | 10.1186/s12876-021-01765-x | Wrong patient population |
| 913 | Yan, L.; Ren, Y.; Qian, K.; Kan, X.; Zhang, H.; Chen, L.; Liang, B.; Zheng, C. | Superselective Transarterial Chemoembolization for Unresectable or "Ablation Unsuitable" Hepatocellular Carcinoma in the Caudate Lobe: A Real World, Single-Center Retrospective Study | 2021 |  | Front Oncol | 11 |  | 678847 | 10.3389/fonc.2021.678847 | Wrong patient population |
| 914 | Yang, B.; Jie, L.; Yang, T.; Chen, M.; Gao, Y.; Zhang, T.; Zhang, Y.; Wu, H.; Liao, Z. | TACE Plus Lenvatinib Versus TACE Plus Sorafenib for Unresectable Hepatocellular Carcinoma With Portal Vein Tumor Thrombus: A Prospective Cohort Study | 2021 |  | Front Oncol | 11 |  | 821599 | 10.3389/fonc.2021.821599 | Wrong patient population |
| 915 | Yang, B.; Liang, J.; Qu, Z.; Yang, F.; Liao, Z.; Gou, H. | Transarterial strategies for the treatment of unresectable hepatocellular carcinoma: A systematic review | 2020 |  | PLoS One | 15 | 2 | e0227475 | 10.1371/journal.pone.0227475 | Wrong patient population |
| 916 | Yang, F.; Xu, G. L.; Huang, J. T.; Yin, Y.; Xiang, W.; Zhong, B. Y.; Li, W. C.; Shen, J.; Zhang, S.; Yang, J.; Sun, H. P.; Wang, W. S.; Zhu, X. L. | Transarterial Chemoembolization Combined With Immune Checkpoint Inhibitors and Tyrosine Kinase Inhibitors for Unresectable Hepatocellular Carcinoma: Efficacy and Systemic Immune Response | 2022 |  | Front Immunol | 13 |  | 847601 | 10.3389/fimmu.2022.847601 |  |
| 917 | Yang, F.; Xu, G. L.; Huang, J. T.; Yin, Y.; Xiang, W.; Zhong, B. Y.; Li, W. C.; Shen, J.; Zhang, S.; Yang, J.; Sun, H. P.; Wang, W. S.; Zhu, X. L. | Transarterial Chemoembolization Combined With Immune Checkpoint Inhibitors and Tyrosine Kinase Inhibitors for Unresectable Hepatocellular Carcinoma: Efficacy and Systemic Immune Response | 2022 |  | Front Immunol | 13 |  | 847601 | 10.3389/fimmu.2022.847601 |  |
| 918 | Yang, F.; Yang, J.; Xiang, W.; Zhong, B. Y.; Li, W. C.; Shen, J.; Zhang, S.; Yin, Y.; Sun, H. P.; Wang, W. S.; Zhu, X. L. | Safety and Efficacy of Transarterial Chemoembolization Combined With Immune Checkpoint Inhibitors and Tyrosine Kinase Inhibitors for Hepatocellular Carcinoma | 2021 |  | Front Oncol | 11 |  | 657512 | 10.3389/fonc.2021.657512 | Wrong patient population |
| 919 | Yang, F.; Yang, J.; Xiang, W.; Zhong, B. Y.; Li, W. C.; Shen, J.; Zhang, S.; Yin, Y.; Sun, H. P.; Wang, W. S.; Zhu, X. L. | Safety and Efficacy of Transarterial Chemoembolization Combined With Immune Checkpoint Inhibitors and Tyrosine Kinase Inhibitors for Hepatocellular Carcinoma | 2021 |  | Front Oncol | 11 |  | 657512 | 10.3389/fonc.2021.657512 |  |
| 920 | Yang, H.; Yang, T.; Qiu, G.; Liu, J. | Efficacy and Safety of TACE Combined with Lenvatinib and PD-(L)1 Inhibitor in the Treatment of Unresectable Hepatocellular Carcinoma: A Retrospective Study | 2023 |  | J Hepatocell Carcinoma | 10 |  | 1435-1443 | 10.2147/jhc.S423684 |  |
| 921 | Yang, Man; Yuan, Jin-Qiu; Bai, Ming; Han, Guo-Hong | Transarterial chemoembolization combined with sorafenib for unresectable hepatocellular carcinoma: a systematic review and meta-analysis | 2014 |  | Molecular biology reports | 41 | 10 | 6575-6582 |  | Wrong patient population |
| 922 | Yang, P.; Zeng, Z. C.; Wang, B. L.; Zhang, J. Y.; Fan, J.; Zhou, J.; Hu, Y. | The Degree of Lipiodol Accumulation Can Be an Indicator of Successful Treatment for Unresectable Hepatocellular Carcinoma (HCC) Patients - in the Case of Transcatheter Arterial Chemoembolization (TACE) and External Beam Radiotherapy (EBRT) | 2016 |  | J Cancer | 7 | 11 | 1413-20 | 10.7150/jca.15405 |  |
| 923 | Yang, X. G.; Sun, Y. Y.; Li, D. S.; Xu, G. H.; Huang, X. Q. | Efficacy and Safety of Drug-Eluting Beads Transarterial Chemoembolization Combining Immune Checkpoint Inhibitors in Unresectable Intrahepatic Cholangiocarcinoma: A Propensity Score Matching Analysis | 2022 |  | Front Immunol | 13 |  | 940009 | 10.3389/fimmu.2022.940009 |  |
| 924 | Yang, X.; Xu, H.; Zuo, B.; Yang, X.; Bian, J.; Long, J.; Wang, D.; Zhang, J.; Ning, C.; Wang, Y.; Xun, Z.; Wang, Y.; Lu, X.; Mao, Y.; Sang, X.; Zhao, H. | Downstaging and resection of hepatocellular carcinoma in patients with extrahepatic metastases after stereotactic therapy | 2021 | Aug | Hepatobiliary Surg Nutr | 10 | 4 | 434-442 | 10.21037/hbsn-21-188 |  |
| 925 | Yang, Y.; Du, N.; Ma, J.; Peng, Z.; Zhou, B.; Yu, J.; Zhou, X.; Zhang, W.; Yan, Z. | Efficacy and Safety of Transarterial Chemoembolization with a Three-Stage Mixed Chemoembolic Regimen for Large Unresectable Hepatocellular Carcinoma | 2023 |  | J Hepatocell Carcinoma | 10 |  | 1897-1910 | 10.2147/jhc.S433409 |  |
| 926 | Yang, Z. W.; He, W.; Zheng, Y.; Zou, R. H.; Liu, W. W.; Zhang, Y. P.; Wang, C. W.; Wang, Y. J.; Yuan, Y. C.; Li, B. K.; Yuan, Y. F. | The efficacy and safety of long- versus short-interval transarterial chemoembolization in unresectable hepatocellular carcinoma | 2018 |  | J Cancer | 9 | 21 | 4000-4008 | 10.7150/jca.24250 |  |
| 927 | Yang, Z.; Hu, Z.; Fu, Y.; Hu, D.; Zhou, Z.; Chen, M.; Pan, Y.; Zhang, Y. | Laparoscopic Hepatectomy versus Open Hepatectomy After Conversion Therapy Using Transarterial Chemoembolization or Hepatic Arterial Infusion Chemotherapy for Patients with Initially Unresectable Hepatocellular Carcinoma | 2023 |  | J Hepatocell Carcinoma | 10 |  | 1157-1167 | 10.2147/jhc.S417739 |  |
| 928 | Yao, M.; Cheng, S.; Zhai, X.; Zhao, H.; Hong, J.; Li, X.; Meng, Y.; Chen, W. | Prognostic Comparison between cTACE and H101-TACE in Unresectable Hepatocellular Carcinoma (HCC): A Propensity-Score Matching Analysis | 2022 |  | Appl Bionics Biomech | 2022 |  | 9084852 | 10.1155/2022/9084852 | Wrong publication type |
| 929 | Yao, X.; Yan, D.; Liu, D.; Zeng, H.; Li, H. | Efficacy and adverse events of transcatheter arterial chemoembolization in combination with sorafenib in the treatment of unresectable hepatocellular carcinoma | 2015 | Jul | Mol Clin Oncol | 3 | 4 | 929-935 | 10.3892/mco.2015.554 | Wrong patient population |
| 930 | Yao, X.; Yan, D.; Zeng, H.; Liu, D.; Li, H. | Concurrent sorafenib therapy extends the interval to subsequent TACE for patients with unresectable hepatocellular carcinoma | 2016 | May | J Surg Oncol | 113 | 6 | 672-7 | 10.1002/jso.24215 | Wrong patient population |
| 931 | Yao, Y.; Chen, J.; Jiao, D.; Li, Y.; Zhou, X.; Han, X. | Elemene injection combined with transcatheter arterial chemoembolization for unresectable hepatocellular carcinoma: A meta-analysis | 2019 | Nov | Medicine (Baltimore) | 98 | 44 | e17813 | 10.1097/md.0000000000017813 | Wrong patient population |
| 932 | Yasui, D.; Yamane, A.; Itoh, H.; Kobayashi, M.; Kumita, S. I. | In vivo evaluation of a monodisperse solid-in-oil-in-water miriplatin/ lipiodol emulsion in transcatheter arterial chemoembolization using a rabbit VX2 tumor model | 2020 |  | PLoS One | 15 | 8 | e0222553 | 10.1371/journal.pone.0222553 |  |
| 933 | Ye, T.; Shao, S. H.; Ji, K.; Yao, S. L. | Evaluation of short-term effects of drug-loaded microspheres and traditional transcatheter arterial chemoembolization in the treatment of advanced liver cancer | 2022 | 15-Dec | World J Gastrointest Oncol | 14 | 12 | 2367-2379 | 10.4251/wjgo.v14.i12.2367 |  |
| 934 | Yeo, K. F.; Ker, A.; Kao, P. E.; Wang, C. C. | Hypothetical hypoxia-driven rapid disease progression in hepatocellular carcinoma post transarterial chemoembolization: A case report | 2023 | 6-Jul | World J Clin Cases | 11 | 19 | 4664-4669 | 10.12998/wjcc.v11.i19.4664 |  |
| 935 | Yeung, C. S. Y.; Chiang, C. L.; Wong, N. S. M.; Ha, S. K.; Tsang, K. S.; Ho, C. H. M.; Wang, B.; Lee, V. W. Y.; Chan, M. K. H.; Lee, F. A. S. | Palliative Liver Radiotherapy (RT) for Symptomatic Hepatocellular Carcinoma (HCC) | 2020 | 27-Jan | Sci Rep | 10 | 1 | 1254 | 10.1038/s41598-020-58108-1 |  |
| 936 | Yildiz, I.; Deniz, S.; Ozer, A.; Caliskan, K. | Trans-Arterial Chemoembolization with 50 µm Degradable Starch Microspheres Versus 300-500 µm Drug Eluting Beads in Hepatocellular Carcinoma: A Comparative Analysis of Initial Treatment Outcomes | 2022 |  | J Belg Soc Radiol | 106 | 1 | 10 | 10.5334/jbsr.2594 | Wrong outcomes |
| 937 | Yim, S. Y.; Chun, H. S.; Lee, J. S.; Lim, J. H.; Kim, T. H.; Kim, B. K.; Kim, S. U.; Park, J. Y.; Ahn, S. H.; Kim, G. M.; Won, J. Y.; Seo, Y. S.; Kim, Y. H.; Um, S. H.; Kim, D. Y. | Transarterial Radioembolization for Unresectable Hepatocellular Carcinoma: Real-Life Efficacy and Safety Analysis of Korean Patients | 2022 | 13-Jan | Cancers (Basel) | 14 | 2 |  | 10.3390/cancers14020385 |  |
| 938 | Yim, S. Y.; Chun, H. S.; Lee, J. S.; Lim, J. H.; Kim, T. H.; Kim, B. K.; Kim, S. U.; Park, J. Y.; Ahn, S. H.; Kim, G. M.; Won, J. Y.; Seo, Y. S.; Kim, Y. H.; Um, S. H.; Kim, D. Y. | Transarterial Radioembolization for Unresectable Hepatocellular Carcinoma: Real-Life Efficacy and Safety Analysis of Korean Patients | 2022 | 13-Jan | Cancers (Basel) | 14 | 2 |  | 10.3390/cancers14020385 |  |
| 939 | Yin, C.; Armstrong, S.; Shin, R.; Geng, X.; Wang, H.; Satoskar, R. S.; Fishbein, T.; Smith, C.; Banovac, F.; Kim, A. Y.; He, A. R. | Bridging and downstaging with TACE in early and intermediate stage hepatocellular carcinoma: Predictors of receiving a liver transplant | 2023 | Mar | Ann Gastroenterol Surg | 7 | 2 | 295-305 | 10.1002/ags3.12622 |  |
| 940 | Yin, L.; Liu, K. C.; Lv, W. F.; Lu, D.; Tan, Y. L.; Wang, G. X.; Dai, J. Y.; Zhu, X. H.; Jiang, B. | Comparing the effectiveness and safety of Sorafenib plus TACE with Apatinib plus TACE for treating patients with unresectable hepatocellular carcinoma: a multicentre propensity score matching study | 2023 | 30-May | Cancer Imaging | 23 | 1 | 52 | 10.1186/s40644-023-00574-7 |  |
| 941 | Yoshimaru, Y.; Nagaoka, K.; Tanaka, K.; Narahara, S.; Inada, H.; Kurano, S.; Tokunaga, T.; Iio, E.; Watanabe, T.; Setoyama, H.; Tateyama, M.; Yoshida, K.; Tsunoda, T.; Nakamura, Y.; Tanaka, M.; Sasaki, Y.; Tanaka, Y. | Randomized phase I/II study of vascular endothelial growth factor receptor peptide vaccines for patients with hepatocellular carcinoma | 2023 | 29-Nov | Hepatol Res |  |  |  | 10.1111/hepr.13995 |  |
| 942 | Yoshimitsu, K. | Transarterial chemoembolization using iodized oil for unresectable hepatocellular carcinoma: perspective from multistep hepatocarcinogenesis | 2014 |  | Hepat Med | 6 |  | 89-94 | 10.2147/hmer.S31440 |  |
| 943 | You, R.; Jiang, H.; Xu, Q.; Yin, G. | Preintervention MCP-1 serum levels as an early predictive marker of tumor response in patients with hepatocellular carcinoma undergoing transarterial chemoembolization | 2021 | Feb | Transl Cancer Res | 10 | 2 | 966-976 | 10.21037/tcr-20-2791 | Wrong patient population |
| 944 | Younes, E. H.; Zahra, H. F.; Soumaya, B. M.; Maria, L.; Nada, L.; Hakima, A.; Samira, E. F.; Meriem, H.; Badreddine, A.; Youssef, H.; Imane, K.; Meryem, B.; Noureddine, A.; Adil, I. S.; Mustapha, M.; Youssef, A. L. M. | Study of predictive factors of complete response after chemoembolization for unresectable hepatocellular carcinoma in 162 patients | 2020 | Dec | Clin Exp Hepatol | 6 | 4 | 313-320 | 10.5114/ceh.2020.102169 | Wrong comparator |
| 945 | Young, Shamar; Sanghvi, Tina; Sharma, Sandeep; Richardson, Cameron; Rubin, Nathan; Richards, Masters; D'Souza, Donna; Flanagan, Siobhan; Golzarian, Jafar | Local recurrence following complete radiologic response in patients treated with transarterial chemoembolization for hepatocellular carcinoma | 2022 |  | Diagnostic and Interventional Imaging | 103 | 3 | 143-149 |  | Wrong patient population |
| 946 | Yu, B.; Zhang, N.; Feng, Y.; Zhang, Y.; Zhang, T.; Wang, L. | Tyrosine Kinase Inhibitors Plus Anti-PD-1 Antibodies with Hepatic Arterial Infusion Chemotherapy or Transarterial Chemoembolization for Unresectable Hepatocellular Carcinoma | 2023 |  | J Hepatocell Carcinoma | 10 |  | 1735-1748 | 10.2147/jhc.S431917 |  |
| 947 | Yu, J. I.; Park, H. C. | Considerations for radiation therapy in hepatocellular carcinoma: the radiation oncologists' perspective | 2014 |  | Dig Dis | 32 | 6 | 755-63 | 10.1159/000368018 |  |
| 948 | Yu, J. I.; Park, H. C.; Lim, D. H.; Kim, C. J.; Oh, D.; Yoo, B. C.; Paik, S. W.; Kho, K. C.; Lee, J. H. | Scheduled interval trans-catheter arterial chemoembolization followed by radiation therapy in patients with unresectable hepatocellular carcinoma | 2012 | Jul | J Korean Med Sci | 27 | 7 | 736-43 | 10.3346/jkms.2012.27.7.736 | Wrong patient population |
| 949 | Yu, J. I.; Park, H. C.; Lim, D. H.; Paik, S. W. | Do Biliary Complications after Hypofractionated Radiation Therapy in Hepatocellular Carcinoma Matter? | 2016 | Apr | Cancer Res Treat | 48 | 2 | 574-82 | 10.4143/crt.2015.076 |  |
| 950 | Yu, M. Q.; An, T. Z.; Li, J. X.; Chang, D. H.; Zhang, Z. S.; Xiao, Y. D. | Integrated Liver Inflammatory Score Predicts the Therapeutic Outcome of Patients with Hepatocellular Carcinoma after Transarterial Chemoembolization | 2021 | Aug | J Vasc Interv Radiol | 32 | 8 | 1194-1202 | 10.1016/j.jvir.2021.03.540 |  |
| 951 | Yu, Q.; Thapa, N.; Karani, K.; Navuluri, R.; Ahmed, O.; Van Ha, T. | Transarterial Radioembolization versus Transarterial Chemoembolization Plus Percutaneous Ablation for Unresectable, Solitary Hepatocellular Carcinoma of ≥ 3 cm: A Propensity Score-Matched Study | 2022 | Dec | J Vasc Interv Radiol | 33 | 12 | 1570-1577.e1 | 10.1016/j.jvir.2022.09.005 |  |
| 952 | Yu, S. C.; Hui, J. W.; Hui, E. P.; Chan, S. L.; Lee, K. F.; Mo, F.; Wong, J.; Ma, B.; Lai, P.; Mok, T.; Yeo, W. | Unresectable hepatocellular carcinoma: randomized controlled trial of transarterial ethanol ablation versus transcatheter arterial chemoembolization | 2014 | Feb | Radiology | 270 | 2 | 607-20 | 10.1148/radiol.13130498 | Wrong patient population |
| 953 | Yu, S. C.; Lau, T. W.; Tang, P.; Chan, S. K.; Chu, C. C.; Hui, J. W.; Lee, K. F.; Chan, A. | Mechanism and Natural Course of Tumor Involution in Hepatocellular Carcinoma Following Transarterial Ethanol Ablation | 2016 | Aug | Cardiovasc Intervent Radiol | 39 | 8 | 1136-43 | 10.1007/s00270-016-1360-z |  |
| 954 | Yu, S. J.; Lee, Y. B.; Cho, E. J.; Lee, J. H.; Kim, H. C.; Chung, J. W.; Yoon, J. H.; Kim, Y. J. | Use of doxorubicin-eluting bead transarterial chemoembolization for unresectable hepatocellular carcinoma with portal vein invasion: a prospective study | 2023 | Mar | J Liver Cancer | 23 | 1 | 166-176 | 10.17998/jlc.2023.02.08 | Wrong outcomes |
| 955 | Yu, X.; Ge, N.; Guo, X.; Shen, S.; Liang, J.; Huang, X.; Wan, S.; Xing, J.; Huang, Q.; Yang, Y. | Genetic variants in the EPCAM gene is associated with the prognosis of transarterial chemoembolization treated hepatocellular carcinoma with portal vein tumor thrombus | 2014 |  | PLoS One | 9 | 4 | e93416 | 10.1371/journal.pone.0093416 |  |
| 956 | Yuan, G.; Liu, Z.; Wang, W.; Liu, M.; Xu, Y.; Hu, W.; Fan, Y.; Zhang, X.; Liu, Y.; Si, G. | Multifunctional nanoplatforms application in the transcatheter chemoembolization against hepatocellular carcinoma | 2023 | 27-Feb | J Nanobiotechnology | 21 | 1 | 68 | 10.1186/s12951-023-01820-7 |  |
| 957 | Yuan, H.; Lan, Y.; Li, X.; Tang, J.; Liu, F. | Large hepatocellular carcinoma with local remnants after transarterial chemoembolization: treatment by sorafenib combined with radiofrequency ablation or sorafenib alone | 2019 |  | Am J Cancer Res | 9 | 4 | 791-799 |  |  |
| 958 | Yuan, P.; Song, J.; Wang, F.; Zhu, G.; Chen, B. | Combination of TACE and Lenvatinib as a promising option for downstaging to surgery of initially unresectable intrahepatic cholangiocarcinoma | 2022 | Oct | Invest New Drugs | 40 | 5 | 1125-1132 | 10.1007/s10637-022-01257-z |  |
| 959 | Yuan, P.; Wang, S.; Zhou, F.; Wan, S.; Yang, Y.; Huang, X.; Zhang, Z.; Zhu, Y.; Zhang, H.; Xing, J. | Functional polymorphisms in the NPAS2 gene are associated with overall survival in transcatheter arterial chemoembolization-treated hepatocellular carcinoma patients | 2014 | Jul | Cancer Sci | 105 | 7 | 825-32 | 10.1111/cas.12428 |  |
| 960 | Zarisfi, M.; Kasaeian, A.; Wen, A.; Liapi, E. | Systematic Review and Pharmacokinetic Meta-analysis of Doxorubicin Exposure in Transcatheter Arterial Chemoembolization and Doxorubicin-Eluted Beads Chemoembolization for Treatment of Unresectable Hepatocellular Carcinoma | 2022 | Jul | Eur J Drug Metab Pharmacokinet | 47 | 4 | 449-466 | 10.1007/s13318-022-00762-z | Wrong study design |
| 961 | Zeng, J. Y.; Piao, X. H.; Zou, Z. Y.; Yang, Q. F.; Qin, Z. L.; Chen, J. B.; Zhou, L.; Niu, L. Z.; Liu, J. G. | Cryoablation with drug-loaded bead embolization in the treatment of unresectable hepatocellular carcinoma: safety and efficacy analysis | 2018 | 26-Jan | Oncotarget | 9 | 7 | 7557-7566 | 10.18632/oncotarget.24029 |  |
| 962 | Zeng, J.; Han, L.; Wang, T.; Huang, L.; Zheng, Y.; Zhang, N.; Li, Z.; Yang, M. | The Allelic Expression of RNA Editing Gene ADARB1 in Hepatocellular Carcinoma Treated with Transarterial Chemoembolization | 2023 |  | Pharmgenomics Pers Med | 16 |  | 229-238 | 10.2147/pgpm.S402115 |  |
| 963 | Zeng, Z. X.; Wu, J. Y.; Wu, J. Y.; Li, Y. N.; Fu, Y. K.; Zhang, Z. B.; Liu, D. Y.; Li, H.; Ou, X. Y.; Zhuang, S. W.; Yan, M. L. | The TAE score predicts prognosis of unresectable HCC patients treated with TACE plus lenvatinib with PD-1 inhibitors | 2023 | 1-Dec | Hepatol Int |  |  |  | 10.1007/s12072-023-10613-x |  |
| 964 | Zhang, H.; Guo, X.; Dai, J.; Wu, Y.; Ge, N.; Yang, Y.; Ji, J.; Zhang, H. | Genetic variations in IDH gene as prognosis predictors in TACE-treated hepatocellular carcinoma patients | 2014 | Nov | Med Oncol | 31 | 11 | 278 | 10.1007/s12032-014-0278-z |  |
| 965 | Zhang, H.; Han, C.; Zheng, X.; Zhao, W.; Liu, Y.; Ye, X. | Significant response to transarterial chemoembolization combined with PD-1 inhibitor and apatinib for advanced intrahepatic cholangiocarcinoma: A case report and literature review | 2023 | Aug | J Cancer Res Ther | 19 | 4 | 1055-1060 | 10.4103/jcrt.jcrt_1697_22 |  |
| 966 | Zhang, H.; Zeng, X.; Peng, Y.; Tan, C.; Wan, X. | Cost-Effectiveness Analysis of Hepatic Arterial Infusion Chemotherapy of Infusional Fluorouracil, Leucovorin, and Oxaliplatin Versus Transarterial Chemoembolization in Patients With Large Unresectable Hepatocellular Carcinoma | 2022 |  | Front Pharmacol | 13 |  | 849189 | 10.3389/fphar.2022.849189 |  |
| 967 | Zhang, H.; Zhao, X.; Yu, W. | Factors Associated with Recurrence of Hepatocellular Carcinoma in 197 Patients Following Transarterial Chemoembolization: A Retrospective Study from a Single Center | 2021 | 17-Sep | Med Sci Monit | 27 |  | e929879 | 10.12659/msm.929879 |  |
| 968 | Zhang, J. X.; Chen, P.; Liu, S.; Zu, Q. Q.; Shi, H. B.; Zhou, C. G. | Safety and Efficacy of Transarterial Chemoembolization and Immune Checkpoint Inhibition with Camrelizumab for Treatment of Unresectable Hepatocellular Carcinoma | 2022 |  | J Hepatocell Carcinoma | 9 |  | 265-272 | 10.2147/jhc.S358658 |  |
| 969 | Zhang, J. X.; Chen, Y. X.; Zhou, C. G.; Liu, J.; Liu, S.; Shi, H. B.; Zu, Q. Q. | Transarterial chemoembolization combined with lenvatinib versus transarterial chemoembolization combined with sorafenib for unresectable hepatocellular carcinoma: A comparative retrospective study | 2022 | Sep | Hepatol Res | 52 | 9 | 794-803 | 10.1111/hepr.13801 |  |
| 970 | Zhang, J. X.; Hua, H. J.; Cheng, Y.; Liu, S.; Shi, H. B.; Zu, Q. Q. | Role of Transarterial Chemoembolization in the Era of Tyrosine Kinase Inhibitor and Immune Checkpoint Inhibitor Combination Therapy for Unresectable Hepatocellular Carcinoma: A Retrospective Propensity Score Matched Analysis | 2023 | 27-Sep | Acad Radiol |  |  |  | 10.1016/j.acra.2023.09.001 |  |
| 971 | Zhang, J.; Feng, G. A.; Li, Y.; Wang, W. | Drug-eluting bead transarterial chemoembolization with medium-sized versus small-sized CalliSpheres microspheres in unresectable primary liver cancer | 2022 | Aug | Asia Pac J Clin Oncol | 18 | 4 | 388-393 | 10.1111/ajco.13660 | Wrong outcomes |
| 972 | Zhang, J.; Feng, G. A.; Li, Y.; Wang, W. | Drug-eluting bead transarterial chemoembolization with medium-sized versus small-sized CalliSpheres microspheres in unresectable primary liver cancer | 2021 | 27-Oct | Asia Pac J Clin Oncol | |  |  | 10.1111/ajco.13660 |  |
| 973 | Zhang, J.; Huang, H.; Bian, J.; Sang, X.; Xu, Y.; Lu, X.; Zhao, H. | Safety, feasibility, and efficacy of associating liver partition and portal vein ligation for staged hepatectomy in treating hepatocellular carcinoma: a systematic review | 2020 | Oct | Ann Transl Med | 8 | 19 | 1246 | 10.21037/atm-20-2214 |  |
| 974 | Zhang, K.; Sun, X.; Xie, F.; Jian, W.; Li, C. | Effectiveness and the strategy to treat the side effects of sorafenib administration after transarterial chemoembolization in advanced hepatocellular carcinoma patients | 2018 | Jan | J Cancer Res Ther | 14 | 1 | 196-200 | 10.4103/jcrt.JCRT_1175_16 |  |
| 975 | Zhang, L.; Xia, W.; Yan, Z. P.; Sun, J. H.; Zhong, B. Y.; Hou, Z. H.; Yang, M. J.; Zhou, G. H.; Wang, W. S.; Zhao, X. Y.; Jian, J. M.; Huang, P.; Zhang, R.; Zhang, S.; Zhang, J. Y.; Li, Z.; Zhu, X. L.; Gao, X.; Ni, C. F. | Deep Learning Predicts Overall Survival of Patients With Unresectable Hepatocellular Carcinoma Treated by Transarterial Chemoembolization Plus Sorafenib | 2020 |  | Front Oncol | 10 |  | 593292 | 10.3389/fonc.2020.593292 |  |
| 976 | Zhang, L.; Yan, Z. P.; Hou, Z. H.; Huang, P.; Yang, M. J.; Zhang, S.; Zhang, S.; Zhang, S. H.; Zhu, X. L.; Ni, C. F.; Li, Q. | Neutrophil-to-Lymphocyte and Platelet-to-Lymphocyte Ratios as Predictors of Outcomes in Patients With Unresectable Hepatocellular Carcinoma Undergoing Transarterial Chemoembolization Plus Sorafenib | 2021 |  | Front Mol Biosci | 8 |  | 624366 | 10.3389/fmolb.2021.624366 | Wrong patient population |
| 977 | Zhang, L.; Yan, Z. P.; Hou, Z. H.; Huang, P.; Yang, M. J.; Zhang, S.; Zhang, S.; Zhang, S. H.; Zhu, X. L.; Ni, C. F.; Li, Q. | Corrigendum: Neutrophil-to-Lymphocyte and Platelet-to-Lymphocyte Ratios as Predictors of Outcomes in Patients With Unresectable Hepatocellular Carcinoma Undergoing Transarterial Chemoembolization Plus Sorafenib | 2021 |  | Front Mol Biosci | 8 |  | 727969 | 10.3389/fmolb.2021.727969 |  |
| 978 | Zhang, L.; Yin, X.; Gan, Y. H.; Zhang, B. H.; Zhang, J. B.; Chen, Y.; Xie, X. Y.; Ge, N. L.; Wang, Y. H.; Ye, S. L.; Ren, Z. G. | Radiofrequency ablation following first-line transarterial chemoembolization for patients with unresectable hepatocellular carcinoma beyond the Milan criteria | 2014 | 10-Jan | BMC Gastroenterol | 14 |  | 11 | 10.1186/1471-230x-14-11 | Wrong patient population |
| 979 | Zhang, N.; Yu, B. R.; Wang, Y. X.; Zhao, Y. M.; Zhou, J. M.; Wang, M.; Wang, L. R.; Lin, Z. H.; Zhang, T.; Wang, L. | Clinical outcomes of hepatic arterial infusion chemotherapy combined with tyrosine kinase inhibitors and anti-PD-1 immunotherapy for unresectable intrahepatic cholangiocarcinoma | 2022 | Aug | J Dig Dis | 23 | 9-Aug | 535-545 | 10.1111/1751-2980.13127 |  |
| 980 | Zhang, T. Q.; Huang, Z. M.; Shen, J. X.; Chen, G. Q.; Shen, L. J.; Ai, F.; Gu, Y. K.; Yao, W.; Zhang, Y. Y.; Guo, R. P.; Chen, M. S.; Huang, J. H. | Safety and effectiveness of multi-antenna microwave ablation-oriented combined therapy for large hepatocellular carcinoma | 2019 |  | Therap Adv Gastroenterol | 12 |  | 1.76E+15 | 10.1177/1756284819862966 | Wrong patient population |
| 981 | Zhang, T.; Zeng, Y.; Huang, J.; Liao, M.; Wu, H. | Combined resection with radiofrequency ablation for bilobar hepatocellular carcinoma: a single-center experience | 2014 | Oct | J Surg Res | 191 | 2 | 370-8 | 10.1016/j.jss.2014.03.048 |  |
| 982 | Zhang, T.; Zhao, Y. T.; Wang, Z.; Li, C. R.; Jin, J.; Jia, A. Y.; Wang, S. L.; Song, Y. W.; Liu, Y. P.; Ren, H.; Fang, H.; Bao, H.; Liu, X. F.; Yu, Z. H.; Li, Y. X.; Wang, W. H. | Efficacy and Safety of Intensity-Modulated Radiotherapy Following Transarterial Chemoembolization in Patients With Unresectable Hepatocellular Carcinoma | 2016 | May | Medicine (Baltimore) | 95 | 21 | e3789 | 10.1097/md.0000000000003789 | Wrong outcomes |
| 983 | Zhang, W.; Chen, L.; Cao, Y.; Sun, B.; Ren, Y.; Sun, T.; Zheng, C. | Efficacy of Drug-Eluting Beads Transarterial Chemoembolization Plus Apatinib Compared with Conventional Transarterial Chemoembolization Plus Apatinib in the Treatment of Unresectable Hepatocellular Carcinoma | 2021 |  | Cancer Manag Res | 13 |  | 5391-5402 | 10.2147/cmar.S314762 | Wrong patient population |
| 984 | Zhang, W.; Zhang, Z. W.; Zhang, B. X.; Huang, Z. Y.; Zhang, W. G.; Liang, H. F.; Chen, X. P. | Outcomes and Prognostic Factors of Spontaneously Ruptured Hepatocellular Carcinoma | 2019 | Sep | J Gastrointest Surg | 23 | 9 | 1788-1800 | 10.1007/s11605-018-3930-7 |  |
| 985 | Zhang, Y. Q.; Jiang, L. J.; Wen, J.; Liu, D. M.; Huang, G. H.; Wang, Y.; Fan, W. Z.; Li, J. P. | Comparison of α-Fetoprotein Criteria and Modified Response Evaluation Criteria in Solid Tumors for the Prediction of Overall Survival of Patients with Hepatocellular Carcinoma after Transarterial Chemoembolization | 2018 | Dec | J Vasc Interv Radiol | 29 | 12 | 1654-1661 | 10.1016/j.jvir.2018.07.031 | Wrong patient population |
| 986 | Zhang, Y.; Fan, W.; Zhu, K.; Lu, L.; Fu, S.; Huang, J.; Wang, Y.; Yang, J.; Huang, Y.; Yao, W.; Li, J. | Sorafenib continuation or discontinuation in patients with unresectable hepatocellular carcinoma after a complete response | 2015 | 15-Sep | Oncotarget | 6 | 27 | 24550-9 | 10.18632/oncotarget.4076 | Wrong patient population |
| 987 | Zhang, Y.; Huang, G.; Wang, Y.; Liang, L.; Peng, B.; Fan, W.; Yang, J.; Huang, Y.; Yao, W.; Li, J. | Is Salvage Liver Resection Necessary for Initially Unresectable Hepatocellular Carcinoma Patients Downstaged by Transarterial Chemoembolization? Ten Years of Experience | 2016 | Dec | Oncologist | 21 | 12 | 1442-1449 | 10.1634/theoncologist.2016-0094 |  |
| 988 | Zhang, Y.; Li, Y.; Ji, H.; Zhao, X.; Lu, H. | Transarterial Y90 radioembolization versus chemoembolization for patients with hepatocellular carcinoma: A meta-analysis | 2015 | Oct | Biosci Trends | 9 | 5 | 289-98 | 10.5582/bst.2015.01089 |  |
| 989 | Zhang, Z. H.; Liu, Q. X.; Zhang, W.; Ma, J. Q.; Wang, J. H.; Luo, J. J.; Liu, L. X.; Yan, Z. P. | Combined endovascular brachytherapy, sorafenib, and transarterial chemobolization therapy for hepatocellular carcinoma patients with portal vein tumor thrombus | 2017 | 21-Nov | World J Gastroenterol | 23 | 43 | 7735-7745 | 10.3748/wjg.v23.i43.7735 |  |
| 990 | Zhang, Z. H.; Zhang, W.; Gu, J. Y.; Liu, Q. X.; Ma, J. Q.; Liu, L. X.; Wang, J. H.; Luo, J. J.; Yan, Z. P. | Treatment of Hepatocellular Carcinoma with Tumor Thrombus with the Use of Iodine-125 Seed Strand Implantation and Transarterial Chemoembolization: A Propensity-Score Analysis | 2018 | Aug | J Vasc Interv Radiol | 29 | 8 | 1085-1093 | 10.1016/j.jvir.2018.02.013 | Wrong patient population |
| 991 | Zhang, Z. M.; Lai, E. C.; Zhang, C.; Yu, H. W.; Liu, Z.; Wan, B. J.; Liu, L. M.; Tian, Z. H.; Deng, H.; Sun, Q. H.; Chen, X. P. | The strategies for treating primary hepatocellular carcinoma with portal vein tumor thrombus | 2015 | Aug | Int J Surg | 20 |  | 16-Aug | 10.1016/j.ijsu.2015.05.009 |  |
| 992 | Zhang, Z.; Jiao, T.; Li, J.; Hu, B.; Zhang, W.; Wang, Z.; Wan, T.; Wang, Y.; Lu, S. | Efficacy of treatment based on TKIs in combination with PD-1 inhibitors for unresectable recurrent hepatocellular carcinoma | 2023 | 18-Feb | World J Surg Oncol | 21 | 1 | 53 | 10.1186/s12957-023-02939-5 |  |
| 993 | Zhang, Z.; Wu, Y.; Zheng, T.; Chen, X.; Chen, G.; Chen, H.; Guo, X.; Zheng, S.; Xie, X.; Zhang, B. | Efficacy of Transarterial Chemoembolization Combined with Molecular Targeted Agents for Unresectable Hepatocellular Carcinoma: A Network Meta-Analysis | 2022 | 29-Jul | Cancers (Basel) | 14 | 15 |  | 10.3390/cancers14153710 |  |
| 994 | Zhao, C.; Fan, L.; Qi, F.; Ou, S.; Yu, L.; Yi, X.; Ni, B.; Zheng, Z.; Lu, J.; Zhang, C.; Chen, C.; Lu, X.; Cheng, L.; Hu, T.; Ma, Y. | Raltitrexed plus oxaliplatin-based transarterial chemoembolization in patients with unresectable hepatocellular carcinoma | 2016 | Aug | Anticancer Drugs | 27 | 7 | 689-94 | 10.1097/cad.0000000000000371 | Wrong patient population |
| 995 | Zhao, C.; Wang, X. J.; Wang, S.; Feng, W. H.; Shi, L.; Yu, C. P. | Lobaplatin combined floxuridine/pirarubicin-based transcatheter hepatic arterial chemoembolization for unresectable primary hepatocellular carcinoma | 2014 |  | Asian Pac J Cancer Prev | 15 | 5 | 2057-60 | 10.7314/apjcp.2014.15.5.2057 | Wrong patient population |
| 996 | Zhao, C.; Xiang, Z.; Li, M.; Wang, H.; Liu, H.; Yan, H.; Huang, M. | Transarterial Chemoembolization Combined with Atezolizumab Plus Bevacizumab or Lenvatinib for Unresectable Hepatocellular Carcinoma: A Propensity Score Matched Study | 2023 |  | J Hepatocell Carcinoma | 10 |  | 1195-1206 | 10.2147/jhc.S418256 |  |
| 997 | Zhao, D.; Xu, W.; Zhan, Y.; Xu, L.; Ding, W.; Xu, A.; Hou, Z.; Ni, C. | Development and Validation of Nomograms to Predict the Prognosis of Patients With Unresectable Hepatocellular Carcinoma Receiving Transarterial Chemoembolization | 2023 |  | Clin Med Insights Oncol | 17 |  | 1.18E+16 | 10.1177/11795549231178178 |  |
| 998 | Zhao, G. S.; Liu, S.; Liu, Y.; Li, C.; Wang, R. Y.; Bian, J.; Zhang, Y. W.; Zhou, J.; Lin, Y. J.; Wu, J. | Assessment of efficacy and prognostic factors by Gelfoam for DEB-TACE in unresectable large hepatocellular carcinoma with portal vein tumor thrombus: a multi-center retrospective study | 2022 | Jul | Expert Rev Gastroenterol Hepatol | 16 | 7 | 673-680 | 10.1080/17474124.2022.2091545 | Wrong patient population |
| 999 | Zhao, G. S.; Song, Y. X.; Sun, J. B.; Liu, S.; Xu, F.; Ma, J.; Li, C.; Gao, F.; Zhou, J.; Wang, R. Y.; Liang, S. N. | Efficacy and safety of CalliSpheres® microspheres drug-eluting beads transarterial chemoembolization in GCLM combined trans-arterial infusion therapy for treating primary focus of gastric cancer: a multi-center retrospective study | 2023 | Jul-Dec | Expert Rev Anticancer Ther | 23 | 9 | 1009-1016 | 10.1080/14737140.2023.2239496 |  |
| 1000 | Zhao, G.; Liu, S.; Chen, S.; Ren, Z.; Li, C.; Bian, J.; Wu, J.; Zhou, J.; Zhang, Y. | Assessment of efficacy and safety by CalliSpheres versus HepaSpheres for drug-eluting bead transarterial chemoembolization in unresectable large hepatocellular carcinoma patients | 2021 | Dec | Drug Deliv | 28 | 1 | 1356-1362 | 10.1080/10717544.2021.1943057 | Wrong patient population |
| 1001 | Zhao, G.; Liu, S.; Zhang, Y.; Zhao, T.; Wang, R.; Bian, J.; Wu, J.; Zhou, J. | Irinotecan eluting beads-transarterial chemoembolization using Callispheres® microspheres is an effective and safe approach in treating unresectable colorectal cancer liver metastases | 2021 | 15-Jul | Ir J Med Sci |  |  |  | 10.1007/s11845-021-02629-9 |  |
| 1002 | Zhao, G.; Liu, S.; Zhang, Y.; Zhao, T.; Wang, R.; Bian, J.; Wu, J.; Zhou, J. | Irinotecan eluting beads-transarterial chemoembolization using Callispheres® microspheres is an effective and safe approach in treating unresectable colorectal cancer liver metastases | 2022 | Jun | Ir J Med Sci | 191 | 3 | 1139-1145 | 10.1007/s11845-021-02629-9 |  |
| 1003 | Zhao, H.; Tsauo, J.; Zhang, X.; Ma, H.; Weng, N.; Yang, Z.; Li, X. | Prevalence and prognostic impact of hepatopulmonary syndrome in patients with unresectable hepatocellular carcinoma undergoing transarterial chemoembolization: a prospective cohort study | 2022 | 5-Sep | Chin Med J (Engl) | 135 | 17 | 2043-2048 | 10.1097/cm9.0000000000002034 |  |
| 1004 | Zhao, H.; Zhai, X.; Chen, Z.; Wan, X.; Chen, L.; Shen, F.; Ling, C. | Transarterial chemoembolization combined with Jie-du granule preparation improves the survival outcomes of patients with unresectable hepatocellular carcinoma | 2017 | 11-Jul | Oncotarget | 8 | 28 | 45234-45241 | 10.18632/oncotarget.16804 | Wrong patient population |
| 1005 | Zhao, J. J.; Tan, E.; Sultana, R.; Syn, N. L.; Da Zhuang, K.; Leong, S.; Tai, D. W. M.; Too, C. W. | Intra-Arterial Therapy for Unresectable Colorectal Liver Metastases: A Meta-Analysis | 2021 | Nov | J Vasc Interv Radiol | 32 | 11 | 1536-1545.e38 | 10.1016/j.jvir.2021.05.032 |  |
| 1006 | Zhao, J.; Wu, J.; He, M.; Cao, M.; Lei, J.; Luo, H.; Yi, F.; Ding, J.; Wei, Y.; Zhang, W. | Comparison of transcatheter arterial chemoembolization combined with radiofrequency ablation or microwave ablation for the treatment of unresectable hepatocellular carcinoma: a systemic review and meta-analysis | 2020 |  | Int J Hyperthermia | 37 | 1 | 624-633 | 10.1080/02656736.2020.1774667 | Wrong patient population |
| 1007 | Zhao, J.; Zeng, L.; Wu, Q.; Wang, L.; Lei, J.; Luo, H.; Yi, F.; Wei, Y.; Yu, J.; Zhang, W. | Stereotactic Body Radiotherapy Combined with Transcatheter Arterial Chemoembolization versus Stereotactic Body Radiotherapy Alone as the First-Line Treatment for Unresectable Hepatocellular Carcinoma: A Meta-Analysis and Systematic Review | 2019 |  | Chemotherapy | 64 | 6-May | 248-258 | 10.1159/000505739 |  |
| 1008 | Zhao, J.; Zhang, H.; Wei, L.; Xie, S.; Suo, Z. | Comparing the long-term efficacy of standard and combined minimally invasive procedures for unresectable HCC: a mixed treatment comparison | 2017 | 28-Feb | Oncotarget | 8 | 9 | 15101-15113 | 10.18632/oncotarget.13145 | Wrong patient population |
| 1009 | Zhao, M.; Wang, J. P.; Pan, C. C.; Li, W.; Huang, Z. L.; Zhang, L.; Fang, W. J.; Jiang, Y.; Li, X. S.; Wu, P. H. | CT-guided radiofrequency ablation after with transarterial chemoembolization in treating unresectable hepatocellular carcinoma with long overall survival improvement | 2012 | Oct | Eur J Radiol | 81 | 10 | 2717-25 | 10.1016/j.ejrad.2011.10.023 |  |
| 1010 | Zhao, P.; Zhao, J.; Deng, Y.; Zeng, G.; Jiang, Y.; Liao, L.; Zhang, S.; Tao, Q.; Liu, Z.; Tang, X.; Tu, X.; Jiang, L.; Zhang, H.; Zheng, Y. | Application of iron/barium ferrite/carbon-coated iron nanocrystal composites in transcatheter arterial chemoembolization of hepatocellular carcinoma | 2021 | Nov | J Colloid Interface Sci | 601 |  | 30-41 | 10.1016/j.jcis.2021.05.102 |  |
| 1011 | Zhao, R. C.; Zhou, J.; Wei, Y. G.; Liu, F.; Chen, K. F.; Li, Q.; Li, B. | Cost-effectiveness analysis of transcatheter arterial chemoembolization with or without sorafenib for the treatment of unresectable hepatocellular carcinoma | 2017 | 15-Oct | Hepatobiliary Pancreat Dis Int | 16 | 5 | 493-498 | 10.1016/s1499-3872(17)60009-2 |  |
| 1012 | Zhao, S.; Dou, W.; Fan, Q.; Hu, J.; Li, H.; Zhang, X.; Zhang, Q.; Liu, L. | Identifying optimal candidates of transarterial chemoembolization (TACE) vs. sorafenib in patients with unresectable hepatocellular carcinoma | 2020 | May | Ann Transl Med | 8 | 9 | 587 | 10.21037/atm.2020.02.123 |  |
| 1013 | Zhao, S.; Zhang, T.; Dou, W.; Wang, E.; Wang, M.; Wang, C.; Du, X.; Liu, L. | A comparison of transcatheter arterial chemoembolization used with and without apatinib for intermediate- to advanced-stage hepatocellular carcinoma: a systematic review and meta-analysis | 2020 | Apr | Ann Transl Med | 8 | 8 | 542 | 10.21037/atm.2020.02.125 |  |
| 1014 | Zhao, S.; Zhang, X.; Wang, M.; Tan, K.; Dou, W.; Fan, Q.; Li, H.; Du, X.; Liu, L. | Identifying optimal candidates for liver resection or transarterial chemoembolisation in patients with unresectable hepatocellular carcinoma | 2020 | May | Ann Transl Med | 8 | 9 | 586 | 10.21037/atm.2020.02.83 |  |
| 1015 | Zhao, S.; Zhou, M.; Wang, P.; Yang, J.; Zhang, D.; Yin, F.; Song, P. | Sorafenib, Lenvatinib, or Lenvatinib Combining PD-1 Inhibitors Plus TACE in Unresectable Hepatocellular Carcinoma: A Retrospective Analysis | 2022 | Jan-Dec | Technol Cancer Res Treat | 21 |  | 1.53E+16 | 10.1177/15330338221133640 |  |
| 1016 | Zheng, X.; Qian, K. | Neutrophil-to-lymphocyte ratio predicts therapy outcomes of transarterial chemoembolization combined with tyrosine kinase inhibitors plus programmed cell death ligand 1 antibody for unresectable hepatocellular carcinoma | 2023 | 1-Jul | Anticancer Drugs | 34 | 6 | 775-782 | 10.1097/cad.0000000000001458 |  |
| 1017 | Zheng, Y. B.; Huang, J. W.; Zhan, M. X.; Zhao, W.; Liu, B.; He, X.; Li, Y.; Hu, B. S.; Lu, L. G. | Genetic variants in the KDR gene is associated with the prognosis of transarterial chemoembolization treated hepatocellular carcinoma | 2014 | Nov | Tumour Biol | 35 | 11 | 11473-81 | 10.1007/s13277-014-2478-8 |  |
| 1018 | Zheng, Y. B.; Meng, Q. W.; Zhao, W.; Liu, B.; Huang, J. W.; He, X.; Li, Y.; Hu, B. S.; Lu, L. G. | Prognostic value of serum vascular endothelial growth factor receptor 2 response in patients with hepatocellular carcinoma undergoing transarterial chemoembolization | 2014 | Mar | Med Oncol | 31 | 3 | 843 | 10.1007/s12032-014-0843-5 |  |
| 1019 | Zhi, W.; Hou, J.; Fan, S.; Han, Y. | Delayed biopsy following completion of transarterial chemoembolization in patients with hepatocellular carcinoma: Effects on pathological outcomes and its advantages | 2022 | Sep | J Cancer Res Ther | 18 | 5 | 1346-1351 | 10.4103/jcrt.jcrt_732_22 | Wrong outcomes |
| 1020 | Zhong, N. B.; Lv, G. M.; Chen, Z. H. | Stereotactic body radiotherapy combined with transarterial chemoembolization for huge (≥10 cm) hepatocellular carcinomas: A clinical study | 2014 | Sep | Mol Clin Oncol | 2 | 5 | 839-844 | 10.3892/mco.2014.304 | Wrong patient population |
| 1021 | Zhou, C.; Wang, R.; Ding, Y.; Du, L.; Hou, C.; Lu, D.; Hao, L.; Lv, W. | Prognostic factors for acute kidney injury following transarterial chemoembolization in patients with hepatocellular carcinoma | 2014 |  | Int J Clin Exp Pathol | 7 | 5 | 2579-86 |  |  |
| 1022 | Zhou, D. S.; Xu, L.; Luo, Y. L.; He, F. Y.; Huang, J. T.; Zhang, Y. J.; Chen, M. S. | Inflammation scores predict survival for hepatitis B virus-related hepatocellular carcinoma patients after transarterial chemoembolization | 2015 | 14-May | World J Gastroenterol | 21 | 18 | 5582-90 | 10.3748/wjg.v21.i18.5582 |  |
| 1023 | Zhou, D.; Liang, J.; Xu, L. I.; He, F.; Zhou, Z.; Zhang, Y.; Chen, M. | Derived neutrophil to lymphocyte ratio predicts prognosis for patients with HBV-associated hepatocellular carcinoma following transarterial chemoembolization | 2016 | May | Oncol Lett | 11 | 5 | 2987-2994 | 10.3892/ol.2016.4359 |  |
| 1024 | Zhou, H.; Song, T. | Conversion therapy and maintenance therapy for primary hepatocellular carcinoma | 2021 | 6-Jul | Biosci Trends | 15 | 3 | 155-160 | 10.5582/bst.2021.01091 |  |
| 1025 | Zhou, J.; Feng, J.; Wu, Y.; Dai, H. Q.; Zhu, G. Z.; Chen, P. H.; Wang, L. M.; Lu, G.; Liao, X. W.; Lu, P. Z.; Su, W. J.; Hooi, S. C.; Ye, X. P.; Shen, H. M.; Peng, T.; Lu, G. D. | Simultaneous treatment with sorafenib and glucose restriction inhibits hepatocellular carcinoma in vitro and in vivo by impairing SIAH1-mediated mitophagy | 2022 | Nov | Exp Mol Med | 54 | 11 | 2007-2021 | 10.1038/s12276-022-00878-x |  |
| 1026 | Zhou, Luke; Zhang, Mao; Chen, Siyu | Comparison of surgical resection and transcatheter arterial chemoembolization for large hepatocellular carcinoma: a systematic review and meta-analysis | 2023 |  | Annals of Hepatology | 28 | 2 | 100890 |  | Wrong study design |
| 1027 | Zhou, W. Z.; Shi, H. B.; Liu, S.; Yang, Z. Q.; Zhou, C. G.; Xia, J. G.; Zhao, L. B.; Li, L. S. | Arterioportal shunts in patients with hepatocellular carcinoma treated using ethanol-soaked gelatin sponge: therapeutic effects and prognostic factors | 2015 | Feb | J Vasc Interv Radiol | 26 | 2 | 223-30 | 10.1016/j.jvir.2014.11.002 | Wrong patient population |
| 1028 | Zhou, Z. G.; Chen, J. B.; Qiu, H. B.; Wang, R. J.; Chen, J. C.; Xu, L.; Chen, M. S.; Zhang, Y. J. | Parecoxib prevents complications in hepatocellular carcinoma patients receiving hepatic transarterial chemoembolization: a prospective score-matched cohort study | 2016 | 10-May | Oncotarget | 7 | 19 | 27938-45 | 10.18632/oncotarget.8560 |  |
| 1029 | Zhu, D.; Ma, K.; Yang, W.; Zhou, H. F.; Shi, Q.; Ren, J. W.; Xie, Y. G.; Liu, S.; Shi, H. B.; Zhou, W. Z. | Transarterial chemoembolization plus apatinib with or without camrelizumab for unresected hepatocellular carcinoma: A two-center propensity score matching study | 2022 |  | Front Oncol | 12 |  | 1057560 | 10.3389/fonc.2022.1057560 |  |
| 1030 | Zhu, H. D.; Li, X.; Ji, J. S.; Huang, M.; Shao, G. L.; Lu, J.; Zhao, X. Y.; Li, H. L.; Yang, Z. Q.; Tu, J. F.; Zhou, J. M.; Zeng, C. H.; Teng, G. J. | TACE with dicycloplatin in patients with unresectable hepatocellular carcinoma: a multicenter randomized phase II trial | 2022 | Nov | Eur Radiol | 32 | 11 | 7335-7343 | 10.1007/s00330-022-08848-7 |  |
| 1031 | Zhu, W.; Qiu, Z.; Yan, H.; Zhong, Z.; Jiang, W.; Zhang, F.; Gao, F. | Retrograde venous coil embolization prior to transarterial chemoembolization in hepatocellular carcinoma with arterio-hepatic venous shunts | 2022 | Nov | Diagnostic and Interventional Radiology | 28 | 6 | 616-620 | 10.5152/dir.2022.211114 |  |
| 1032 | Zhu, Y.; Sun, P.; Wang, K.; Xiao, S.; Cheng, Y.; Li, X.; Wang, B.; Li, J.; Yu, W.; Cheng, Y. | Efficacy and safety of lenvatinib monotreatment and lenvatinib-based combination therapy for patients with unresectable hepatocellular carcinoma: a retrospective, real-world study in China | 2021 | 18-Sep | Cancer Cell Int | 21 | 1 | 503 | 10.1186/s12935-021-02200-7 |  |
| 1033 | Zhu, Y.; Wang, E.; Zhao, S.; Han, D.; Zhao, Y.; Chen, H.; Zhu, J.; Han, T.; Bai, Y.; Lou, Y.; Zhang, Y.; Yang, M.; Zuo, L.; Fan, J.; Chen, X.; Jia, J.; Wu, W.; Ren, W.; Bai, T.; Ma, S.; Xu, F.; Tang, Y.; Han, Y.; Zhao, J.; Qi, X.; Li, J.; Du, X.; Chen, D.; Liu, L. | Identify optimal HAP series scores for unresectable HCC patients undergoing TACE plus sorafenib: A Chinese multicenter observational study | 2022 |  | Front Oncol | 12 |  | 983554 | 10.3389/fonc.2022.983554 |  |
| 1034 | Zhu, Z. Y.; Yuan, M.; Yang, P. P.; Xie, B.; Wei, J. Z.; Qin, Z. Q.; Qian, Z.; Wang, Z. Y.; Fan, L. F.; Qian, J. Y.; Tan, Y. L. | Single medium-sized hepatocellular carcinoma treated with sequential conventional transarterial chemoembolization (cTACE) and microwave ablation at 4 weeks versus cTACE alone: a propensity score | 2022 | 10-Jun | World J Surg Oncol | 20 | 1 | 192 | 10.1186/s12957-022-02643-w | Wrong patient population |
| 1035 | Zou, Jing Huai; Zhang, Lan; Ren, Zheng Gang; Ye, Sheng Long | Efficacy and safety of cTACE versus DEB-TACE in patients with hepatocellular carcinoma: a meta-analysis | 2016 |  |  | 17 | 8 | 510-517 |  | Wrong patient population |
| 1036 | Zou, X.; Fan, W.; Xue, M.; Li, J. | Evaluation of the Benefits of TACE Combined with Sorafenib for Hepatocellular Carcinoma Based on Untreatable TACE (unTACEable) Progression | 2021 |  | Cancer Manag Res | 13 |  | 4013-4029 | 10.2147/cmar.S304591 |  |
| 1037 | Zou, Z. M.; An, T. Z.; Li, J. X.; Zhang, Z. S.; Xiao, Y. D.; Liu, J. | Predicting early refractoriness of transarterial chemoembolization in patients with hepatocellular carcinoma using a random forest algorithm: A pilot study | 2021 |  | J Cancer | 12 | 23 | 7079-7087 | 10.7150/jca.63370 |  |
| 1038 |  | [Expert consensus on transarterial chemoembolization refractoriness and subsequent therapies in hepatocellular carcinoma] | 2022 | 1-Aug | Zhonghua Nei Ke Za Zhi | 61 | 8 | 860-866 | 10.3760/cma.j.cn112138-20220615-00452 |  |
| 1039 |  | [Chinese clinical practice guidelines for transarterial chemoembolization of hepatocellular carcinoma (2023 edition)] | 2023 | 12-Sep | Zhonghua Yi Xue Za Zhi | 103 | 34 | 2674-2694 | 10.3760/cma.j.cn112137-20230630-01114 |  |

# 
