# Supplementary material for: Clinical and safety outcomes in unresectable, very early and early-stage hepatocellular carcinoma following Irreversible Electroporation (IRE) and Transarterial Chemoembolization (TACE): A systematic literature review and meta-analysis
Source: PLoS One. 2025 Apr 29;20(4):e0322113. doi: 10.1371/journal.pone.0322113 (PMC12083900; doi:10.1371/journal.pone.0322113)
Supplement: S2 Fig — (PDF) [file pone.0322113.s021.pdf]

S2 Fig. Forest Plot, IRE PD Results at 1 Month

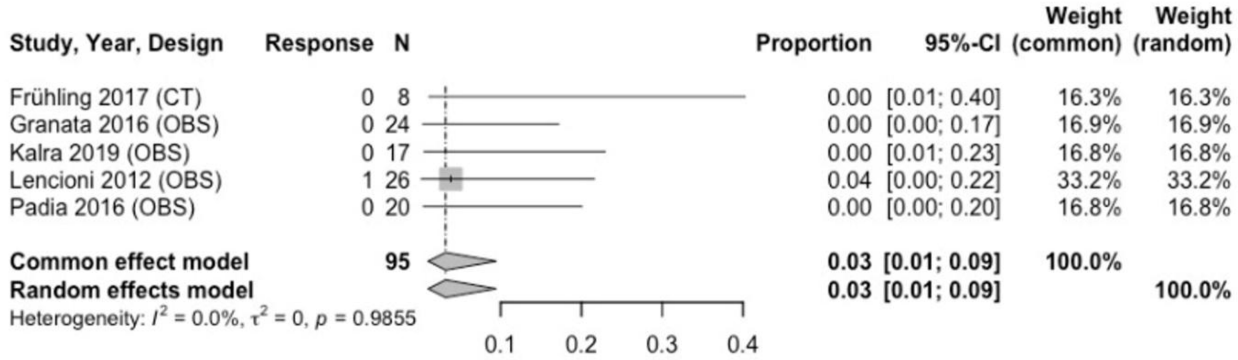

Abbreviations: CT, clinical trial; OBS, observational study; PD, progressive disease; CI, confidence interval
