# Supplementary material for: Clinical and safety outcomes in unresectable, very early and early-stage hepatocellular carcinoma following Irreversible Electroporation (IRE) and Transarterial Chemoembolization (TACE): A systematic literature review and meta-analysis
Source: PLoS One. 2025 Apr 29;20(4):e0322113. doi: 10.1371/journal.pone.0322113 (PMC12083900; doi:10.1371/journal.pone.0322113)
Supplement: S7 Fig — (PDF) [file pone.0322113.s026.pdf]

S7 Fig. Forest Plot, TACE SD Results, 0 to < 3 Months

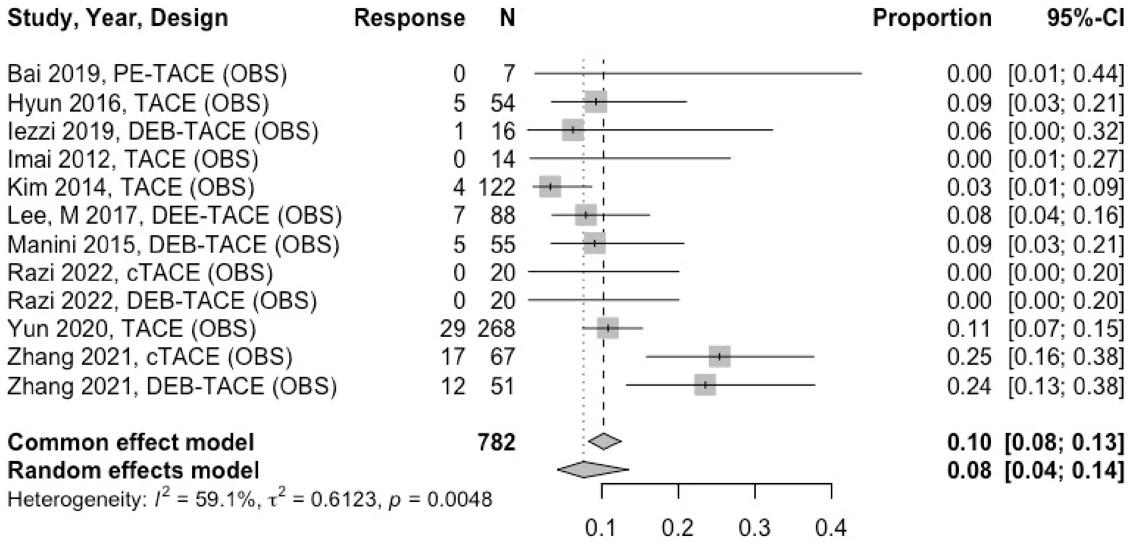

Abbreviations: OBS, observational study; SD, stable disease; CI, confidence interval; TACE, transarterial chemoembolization; cTACE, conventional transarterial chemoembolization; DEB-TACE, drug-eluting bead transarterial chemoembolization; DEE-TACE, drug-eluting embolic transarterial chemoembolization; PE-TACE; pirarubin-eluting transarterial chemoembolization
