# Supplementary material for: Clinical and safety outcomes in unresectable, very early and early-stage hepatocellular carcinoma following Irreversible Electroporation (IRE) and Transarterial Chemoembolization (TACE): A systematic literature review and meta-analysis
Source: PLoS One. 2025 Apr 29;20(4):e0322113. doi: 10.1371/journal.pone.0322113 (PMC12083900; doi:10.1371/journal.pone.0322113)
Supplement: S9 Fig — (PDF) [file pone.0322113.s028.pdf]

S9 Fig. Forest Plot, TACE PD Results, 3 to < 6 Months

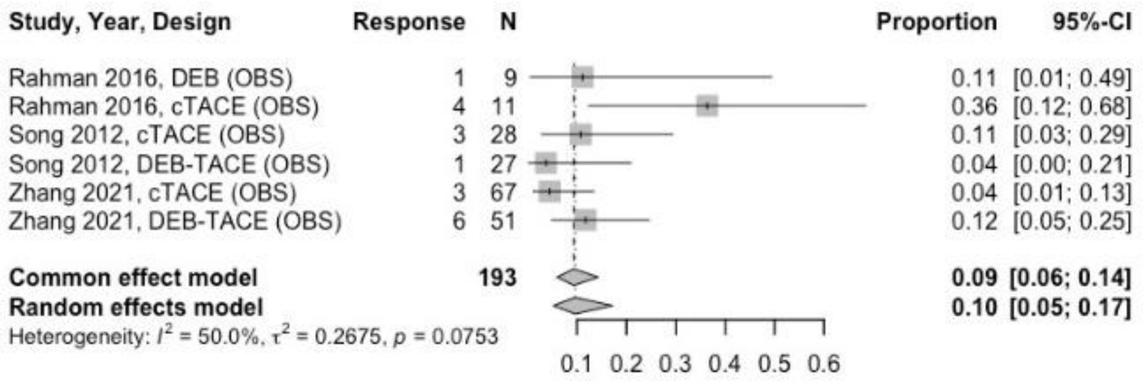

Abbreviations: OBS, observational study; PD, progressive disease; CI, confidence interval; cTACE, conventional transarterial chemoembolization; DEB-TACE, drug-eluting bead transarterial chemoembolization
